# GbPP2C80 Interacts with GbWAKL14 to Negatively Co‐Regulate Resistance to *Fusarium* and *Verticillium wilt* via MPK3 and ROS Signaling in Sea Island Cotton

**DOI:** 10.1002/advs.202309785

**Published:** 2024-06-18

**Authors:** Nan Zhao, Anhui Guo, Weiran Wang, Bin Li, Meng Wang, Zixin Zhou, Kaiyun Jiang, Alifu Aierxi, Baoliang Wang, Daniel Adjibolosoo, Zhanghao Xia, Huijing Li, Yanan Cui, Jie Kong, Jinping Hua

**Affiliations:** ^1^ Joint Laboratory for International Cooperation in Crop Molecular Breeding Ministry of Education College of Agronomy and Biotechnology China Agricultural University Beijing 100193 China; ^2^ Institute of Economic Crops Xinjiang Academy of Agricultural Sciences Urumqi Xinjiang 830091 China

**Keywords:** Fusarium wilt, protein phosphatase 2C80, reactive oxygen species, Sea Island cotton, Verticillium wilt, wall‐associated receptor kinase‐like 14

## Abstract

*Fusarium wilt* (*FW*) is widespread in global cotton production, but the mechanism underlying *FW* resistance in superior‐fiber‐quality Sea Island cotton is unclear. This study reveals that *FW* resistance has been the target of genetic improvement of Sea Island cotton in China since the 2010s. The key nonsynonymous single nucleotide polymorphism (SNP, T/C) of gene *Gbar_D03G001670* encoding protein phosphatase 2C 80 (PP2C80) results in an amino acid shift (L/S), which is significantly associated with *FW* resistance of Sea Island cotton. Silencing *GbPP2C80* increases *FW* resistance in Sea Island cotton, whereas overexpressing *GbPP2C80* reduces *FW* resistance in *Arabidopsis*. *GbPP2C80* and *GbWAKL14* exist synergistically in Sea Island cotton accessions with haplotype forms “susceptible–susceptible” (TA) and “resistant–resistant” (CC), and interact with each other. CRISPR/Cas9‐mediated knockout of *GbWAKL14* enhances *FW* and *Verticillium wilt* (*VW*) resistance in upland cotton and overexpression of *GbWAKL14* and *GbPP2C80* weakens *FW* and *VW* resistance in *Arabidopsis*. *GbPP2C80* and *GbWAKL14* respond to *FW* and *VW* by modulating reactive oxygen species (ROS) content via affecting *MPK3* expression. In summary, two tandem genes on chromosome D03, *GbPP2C80*, and *GbWAKL14*, functions as cooperative negative regulators in cotton wilt disease defense, providing novel genetic resources and molecular markers for the development of resistant cotton cultivars.

## Graphical abstract

Wilt diseases have caused significant losses in cotton production, however, the resistance genes and molecular mechanisms are unclear. Here, the genes *GbPP2C80* and *GbWAKL14* are identified to synergistically negatively regulate *FW* and *VW* resistance by modulating MPK3 and ROS signaling. The resistance conferred by *GbPP2C80* and *GbWAKL14* comes from the introduction and improvement of *G. barbadense*.

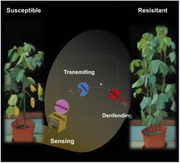

## 1 Introduction

Cotton is one of the most important industrial crops. Sea Island cotton is a cultivated cotton species with excellent fibers that are necessary raw materials for high‐end fabrics. *Fusarium wilt* is the main disease that threatens the yield and quality of Sea Island cotton.^[^ ^1^ ^]^ Xinjiang, one of the four major Sea Island cotton‐producing areas in the world, and in particular the only one in China, has seen the disease incidence of *Fusarium wilt* rise to 70%, resulting in production cuts of more than 30%. Therefore, improving the *Fusarium wilt* resistance of Sea Island cotton is an urgent issue to be addressed in the production of high‐end cotton.

*Fusarium wilt* (*FW*) disease is caused by the soil‐borne fungus *Fusarium oxysporum* f. sp. *vasinfectum* (*Fov*), which can infect over 100 plants, including cotton.^[^ ^2^ ^]^ Depending on pathogenicity, *Fusarium oxysporum* can be classified into races 1 to 8, of which *Fov* race 7 is the most widespread and virulent.^[^ ^3^ ^]^ The pathogenicity of *Fov* race 7 varies from region to region, with that from Wulumuqi (Xinjiang, China) being the most pathogenic and causing *Fusarium wilt* symptoms in cotton 25 to 30 days post inoculation (dpi).^[^ ^4^ ^]^ Typical symptoms include leaf chlorosis and necrosis, seedling wilting, accompanied by vascular discoloration, plant growth retardation, and eventually the death of the entire plant.^[^ ^5^ ^]^ *Fusarium oxysporum* enters the xylem by infecting the roots of cotton, disrupting water conduction, secreting many pathogenic factors such as small peptides, phytotoxins, etc., causing the catheter to turn brown and eventually causing chlorisis and wilting of the above‐ground leaves.^[^ ^3^ ^]^ Additionally, *Fusarium oxysporum* produces chlamydospores that can lie dormant in the soil for up to 10 years in the absence of a host plant, and the use of pesticides to control the disease has limited effectiveness and can cause soil and water pollution.^[^ ^6^ ^]^ Therefore, it is crucial to uncover the *FW* resistance genes and dissect the molecular mechanisms of disease resistance in cotton.

Some *FW* resistance genes have been identified in different cotton species. In the diploid species *G. arboreum*, the gene *GaGSTF9* (*Ga11G2353*) on chromosome 11 was associated with *Fov* (race Ag149) resistance through GWAS of 215 *G. arboreum* accessions.^[^ ^7^ ^]^ The gene (*Gh_D03G0209*), encoding a GLUTAMATE RECEPTOR‐LIKE (GLR) protein, was identified to affect *FW* resistance by GWAS of 290 diverse upland cotton accessions.^[^ ^3^ ^]^ In addition, several other resistance factors have been identified,^[^ ^8^ ^]^ including wall‐associated kinase,^[^ ^9^ ^]^ receptor(‐like) proteins,^[^ ^3^, ^10^ ^]^ protein phosphatase,^[^ ^11^ ^]^ mitogen‐activated protein kinase signaling cascades,^[^ ^12^ ^]^ lignin synthesis,^[^ ^13^ ^]^ fatty acid formation,^[^ ^14^ ^]^ and other defense‐associated proteins.^[^ ^1^ ^]^

However, relatively few studies have been performed on the *FW* resistance of Sea Island cotton. The QTL for *Fov*1 resistance in Pima S‐7, a Sea Island cotton‐resistant cultivar, was located on chromosome 16.^[^ ^15^ ^]^ Three QTLs for *Fov*4 resistance were mapped on chromosomes c17, c24, and c25 of Sea Island cotton‐resistant cultivar Pima S‐6.^[^ ^16^ ^]^ A QTL (*qFov race 7‐D03‐1*) for *Fov*7 resistance was mapped on chromosome D03 of Sea Island cotton‐resistant cultivar 06–146, and a calmodulin‐like protein coding gene *GB_D03G0217* was identified to increase the severity of the disease by VIGS (virus‐induced gene silencing).^[^ ^17^ ^]^Additionally, transcriptomic analysis unveiled some differentially expressed genes associated with *FW* resistance,^[^ ^18^ ^]^ such as chalcone isomerase gene *GbCHI01* involved in phenylalanine metabolism,^[^ ^19^ ^]^ glucosyltransferase gene *GbUGT73C1*,^[^ ^20^ ^]^ and glutathione transferase gene *GbGSTU7*.^[^ ^21^ ^]^ Given the limited number of cloned resistance genes, it is urgent to identify and clone more *FW* resistance genes in Sea Island cotton, which will help to further understand the molecular mechanisms of disease resistance in cotton.

The resistance genes and mechanisms of other plants, such as the first barrier and sensor, wall‐associated receptor kinase‐like (WAKL), can also provide references for the study on *FW* resistance of Sea Island cotton. In sweet orange, *CsWAKL08* was shown to confer resistance to citrus bacterial canker through ROS control and JA signaling.^[^ ^22^ ^]^ In rice, OsWAKL21.2 activated rice immune responses through its kinase activity and *Arabidopsis* immune responses through its guanylate cyclase activity.^[^ ^23^ ^]^ In upland cotton, GhWAK7A interacted directly with GhLYK5 and GhCERK1, promoting the formation of a chitin‐induced GhLYK5‐GhCERK1 dimer and phosphorylating GhLYK5, ultimately modulating cotton responses to *Verticillium wilt* and *Fusarium wilt*.^[^ ^9^ ^]^ Knockout of *GhWAKL* (*Gh_D04G1868*) increased the susceptibility of upland cotton to *Verticillium wilt*, and overexpression of *GhWAKL* increased the resistance of *Arabidopsis thaliana* to *Verticillium wilt*; GhWAKL interacted with GhDNAJ1 on the membrane, but GhWAKL with phosphorylation site Ser^628^ mutation reduced the interaction with GhDNAJ1 and weakened plant resistance to *Verticillium wilt*.^[^ ^24^ ^]^ Whether *WAK(L)* genes play a role in *FW* resistance of Sea Island cotton by phosphorylating downstream genes through its kinase activity or by influencing ROS metabolism is worth further exploring.

Another potential *FW* resistance factor may be type 2C protein phosphatase (PP2C). PP2C is a key negative regulator of plant immunity.^[^ ^25^ ^]^ In *Arabidopsis*, type 2C protein phosphates PLL4 and PLL5 dephosphorylated receptor kinases FLS2 and EFR, ultimately affecting pattern‐triggered immunity (PTI).^[^ ^26^ ^]^ In tomatoes, PP2C is a candidate regulator of either a single or a multi‐level immune signaling network.^[^ ^27^ ^]^ For instance, type 2C protein phosphatase Pic1 acted as a negative regulator of PTI signaling by dephosphorylating receptor‐like cytoplasmic kinase Pti1b, reducing ROS production and thus disrupting its ability to activate plant immune response.^[^ ^28^ ^]^ In wheat, the key hub protein phosphatase 2C 70 interacted with several resistance proteins such as RLP37, RPP13, and RPS2 analogs, and PP2C isoform X1 was mapped to the genetic fragment of powdery mildew resistance gene *PmAS846* at a distance of 4.8 Mb.^[^ ^29^ ^]^ In upland cotton, protein phosphatase GhAP2C1 and GhMPK4 synergistically regulated the immune response to *Fusarium wilt*; after silencing and overexpressing of *GhAP2C1*, resistance to *Fusarium wilt* was enhanced and attenuated, respectively.^[^ ^11^ ^]^

Additionally, a few genes could simultaneously mediate responses to fungal wilt pathogens *Fov and Verticillium dahliae* (*Vd*), which cause devastating wilt diseases in cotton, *Fusarium wilt*, and *Verticillium wilt*, respectively. A wall‐associated kinase, GhWAK7A, phosphorylated lysin‐motif‐containing receptor‐like kinases GhLYK5 and promoted chitin‐induced dimerization of GhLYK5‐GhCERK1, further activating cytoplasmic signaling events including ROS production, MAPK activation, and the expression of defense‐related genes to fend off *Fov* and *Vd* infections.^[^ ^9^ ^]^ The germin‐like proteins GhGLP2 and GhABP19 played important roles in the regulation of resistance to *Fusarium* and *Verticillium wilt* pathogens through the SOD activity.^[^ ^1^, ^30^ ^]^ GhBsr‐k1 acted as a negative regulator during cotton resistance against *Vd* and *Fov* by regulating the transcription of lignin deposition genes (*GhPAL2* and *GhPAL5*).^[^ ^31^ ^]^ The above results provide important clues for rapid screening and efficient improvement of wilt‐resistant cotton varieties by exploiting genes that co‐regulate *FW* and *VW* resistance.

In our previous work, we screened out an *FW*‐resistance candidate gene, *GbDP2*, which encoded a putative wall‐associated receptor kinase‐like 14 (WAKL14); the expression of this gene in the sensitive variety was higher than in the resistant variety, and the *FW* resistance was improved by silencing this gene in Sea Island cotton.^[^ ^32^ ^]^ Here, we performed GWAS to identify another *FW*‐resistant gene, *GbPP2C80*, which encodes a protein phosphatase and is located upstream of *GbWAKL14* on chromosome D03. The regulatory role of *GbWAKL14* in *FW* resistance has been validated by VIGS in Sea Island cotton and by overexpression in *Arabidopsis*. The relationship between GbPP2C80 and GbWAKL14 was explored using haplotype analysis, subcellular localization, protein interaction, and expression analysis. The roles of *GbWAKL14* in *FW* and *VW* resistance have been elucidated using CRISPR/Cas9 editing in upland cotton and overexpression in *Arabidopsis*. Furthermore, the downstream pathways and molecular mechanisms have also been explored. This study has important implications for the exploration of novel wilt‐resistant genes and broadening disease resistance mechanisms in cotton.

## 2 Results

### 2.1 *FW* Resistance is the Target of Genetic Improvement in Sea Island Cotton

To accurately analyze the genetic resistance of the various cotton genotypes, we evaluated the disease percentage of *FW* in the field. Phenotypic data of 336 Sea Island cotton accessions gathered worldwide^[^ ^32^ ^]^ were investigated in a natural disease nursery in Korla (Xinjiang, China) in the years 2015, 2016, 2018, and 2019. The *FW* disease percentage (DP, %) varied greatly, ranging from 0 to 98.78% at the seedling stage (3‐4 leaf stage)(Table S1, Supporting Information). The DP correlation coefficient was higher between 2018 and 2019, followed by that between 2015 and 2016 (Figure S1a, Supporting Information). According to the average DP value of different years, all cotton accessions were divided into five groups, including g1 (0.00 ≤ DP < 1.00%), g2 (1.00% ≤ DP < 25.00%), g3 (25.00% ≤ DP < 50.00%), g4 (50.00% ≤ DP < 75.00%), and g5 (75.00% ≤ DP < 100.00%) (Figure S1, Supporting Information). These results showed that the disease percentage of *FW* in Sea Island cotton had a large phenotypic variation and is suitable for GWAS.

**Figure 1:**
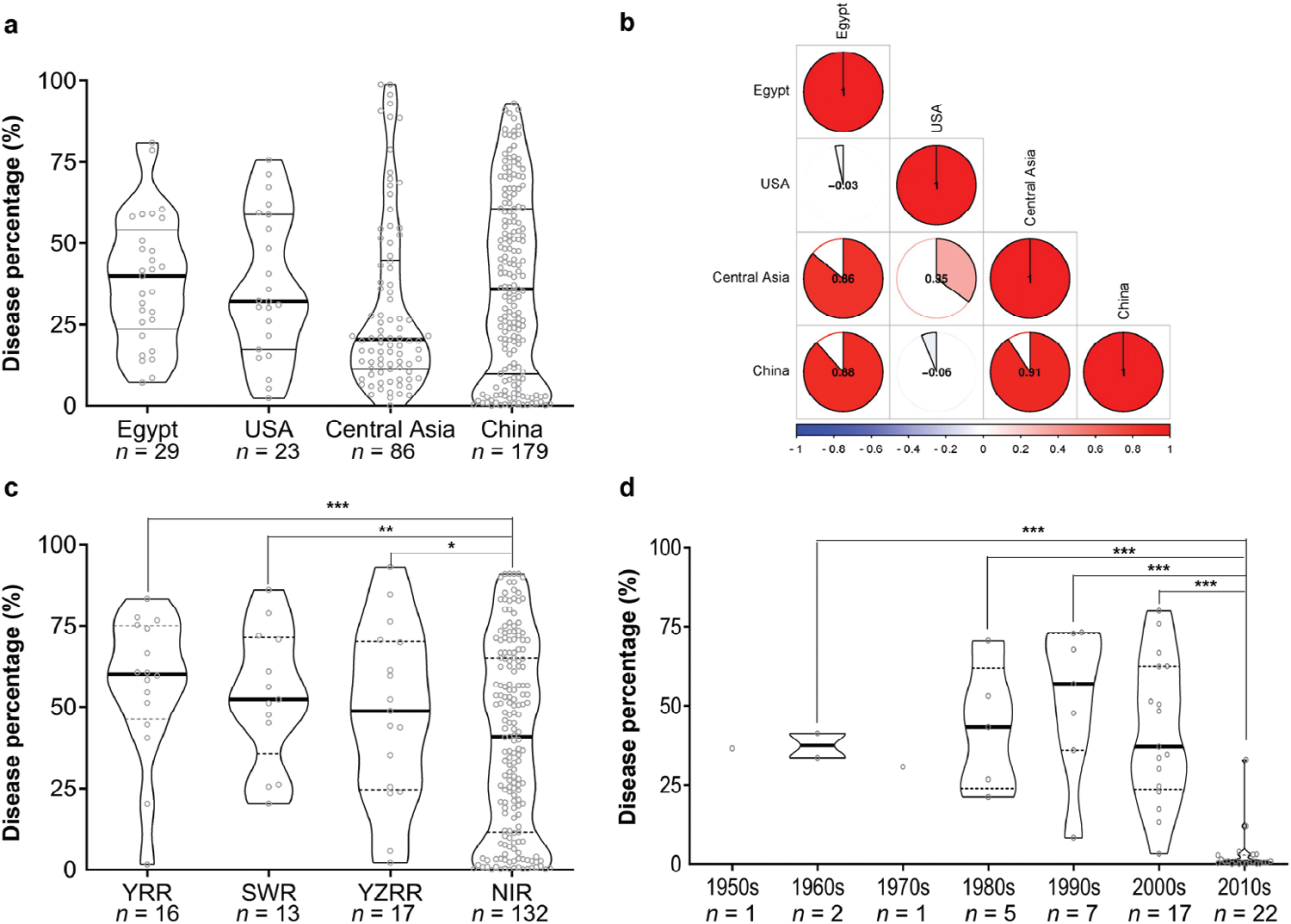
Most Sea Island cotton bred in the 2010s in the Northwest Inland cotton region of China were *FW‐*resistant varieties. a) *FW* disease percentage in Sea Island cotton from four cotton production regions in the world. b) Correlation analysis of *FW* disease percentage in Sea Island cotton from four cotton production regions in the world. c) *FW* disease percentage in Sea Island cotton from four cotton production regions in China. SWR, Southwest region; YRR, Yellow River region; YZRR, Yangtze River region; NIR, Northwestern Inland region. d) *FW* disease percentage in Sea Island cotton varieties bred in Xinjiang (China) at different breeding stages.

To explore the trend of genetic breeding for disease resistance in Sea Island cotton, the differences in disease percentage of *FW* were analyzed in terms of geographic location and breeding period. Due to the wide range of DP values, there was no significant difference in DP values between the four main Sea Island cotton‐producing regions; however, compared with the USA and Egypt, the number of low *FW*‐resistant accessions in China and Central Asia was much higher (**Figure** **1**a), suggesting that *FW* resistance was gradually paid attention to after the introduction of Sea Island cotton accessions into China. Additionally, the *FW*‐DP of Sea Island cotton accessions in China had the highest correlation with that in Central Asia, followed by that in Egypt (Figure 1b), demonstrating that Chinese Sea Island cotton varieties were more closely related to Central Asia. In China, the *FW* disease percentage of Sea Island cotton accessions in the Northwestern Inland region (NIR) was significantly lower than that in other regions, including the Yangtze River region (YZRR), Southwest region (SWR) and Yellow River region (YRR) (Figure 1c). This is strictly restricted by suitable thermal and light resources in the southern areas of Xinjiang (NIR), which is the most dominating growing region for Sea Island cotton in China. Years of cultivation have exacerbated the occurrence of soil‐borne diseases such as *FW* and *VW*, forcing breeders to breed disease‐resistant Sea Island cotton varieties to meet the demands of cotton production. In southern areas of Xinjiang, China, relatively resistant varieties began to appear in the 1990s. After this, the number of resistant varieties and the degree of disease resistance increased somewhat in the 2000s; Until the 2010s, almost all selected varieties were extremely resistant to *FW* (Figure 1d).

### 2.2 A key Non‐Synonymous SNP in GbPP2C80 is Significantly Associated with FW Resistance in Sea Island Cotton

**Figure 2:**
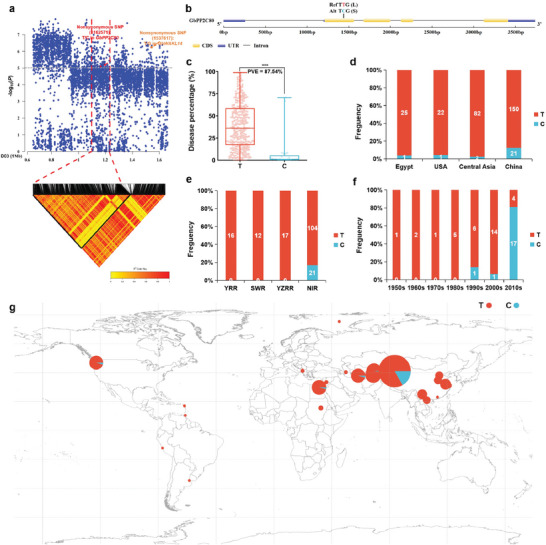
Location, effect, frequency, and geographical distribution of the key non‐synonymous SNP (1 134 219) of *GbPP2C80*, an *FW*‐resistance‐related gene. a) Local Manhattan plot (top) and LD heatmap (bottom) surrounding the key non‐synonymous SNP on D03. The dashed line indicated the significance threshold (−log_10_ *P* = 5). We performed a statistical analysis using the *F*‐test. The red dot and red arrow indicated the position of the non‐synonymous SNP (1 134 219) in *GbPP2C80*. The red dotted line showed the candidate LD region. The orange dot and orange arrow indicated the position of the non‐synonymous SNP (1 537 617) in *GbWAKL14*. b) Structure of gene *GbPP2C80* containing the key non‐synonymous SNP in the second exon. Blue and yellow rectangles mark the UTR and CDS, respectively. The reference type of key nonsynonymous SNP in the reference genome of Sea Island cotton 3–79 was “T” marked in red, and the alternative type was “C” labeled in blue. “TTG” and “TCG” were the genetic codons in which the key non‐synonymous SNP residue. The “L” and “S” in parentheses are abbreviations for amino acids, leucine, and serine, which were caused by key nonsynonymous SNP, T and C. c) Box plot for DP based on the haplotypes of the non‐synonymous SNP. In a box plot, the centerline indicated the median, the box limits were the upper and lower quartiles, and the whiskers marked the range of the data. PEV represented phenotypes explained by variation. We performed a significance analysis using a two‐tailed *t*‐test. *** indicated an extremely significant difference (*P *< 0.001). d) The frequencies of Sea Island cotton accessions with different haplotypes in four worldwide cotton‐production regions. f) The frequencies of Sea Island cotton accessions with different haplotypes from four cotton‐production regions in China. SWR, Southwest region; YRR, Yellow River region; YZRR, Yangtze River region; NIR, Northwestern Inland region. e) The frequencies of Sea Island cotton accessions with different haplotypes in different breeding stages in Xinjiang, China. g) Geographical distribution of Sea Island cotton accessions with different haplotypes on a world map.

Taking *P *< 10^−6^ as the significance threshold, 1513, 2129, 170, 242, 209, and 263 significant SNPs were detected by GWAS in datasets of 2015, 2016, 2018, 2019, Mean and BLUP, respectively (Table S2, Supporting Information). Among a total of 2398 SNPs, 22, 23, 103, 103, 1411, and 736 significant SNPs were repeatedly detected in 6, 5, 4, 3, 2, and 1 datasets, respectively, with *P*‐values ranging from 6.00 to 13.12 (Table S3, Supporting Information). There were significantly associated SNP signals on chromosomes A02, A07, A11, A13, D03, D05, D06, and D12 (Table S4, Supporting Information). There was a repeatedly detectable peak on chromosome D03 in all 6 datasets, indicating that it was a significantly associated SNP cluster, harboring 99.42% (2384/2398) of the associated SNPs (Table S4, Supporting Information). Based on the *P* values and dataset repeats, we screened a set of core SNPs located in a 1.16 Mb (1, 068, 053‐2, 224, 222 bp) genomic region on D03 (Table S5, Supporting Information). We then searched for the gene that was closest to these core SNPs and contained key non‐synonymous SNP affecting *FW* resistance. Finally, we found a gene *Gbar_D03G001670* (hereafter referred to *GbPP2C80*), in which a T/C nonsynonymous SNP (1 162 571) was located in the second exon (**Figure** **2**a) and caused a leucine (L)/Serine (S) shift (Figure 2b). *FW* disease percentage was significantly lower in Sea Island cotton accessions carrying the C haplotype than those carrying T, accounting for 87.54% of phenotypic variation (Figure 2c).

To explore the effect of the key non‐synonymous SNP (1 162 571) of the *GbPP2C80* gene on *FW* disease percentage, we further analyzed the frequency distribution of this SNP (1 162 571) in Sea Island cotton accessions from different geographic regions and breeding stages. There were 1 (3.85%), 1 (4.35%), and 2 (2.38%) Sea Island cotton accessions with resistant C type in the USA, Egypt, and Central Asia, respectively, whereas there were 21 (12.28%) in China (Figure 2d,g), consistent with the relatively large number of Sea Island cotton accessions with lower DP in China (Figure 1a). In China, the resistant haplotype C was found only in Sea Island cotton accessions from the Northwestern Inland region (NIR) (Figure 2e,g), which was consistent with the lowest DP of Sea Island cotton growing in NIR regions, China (Figure 1c).

The varieties with relatively lower DP appeared in the 1990s, and the C haplotype also occurred in the 1990s and remained unchanged in the 2000s, accounting for 80.95% in the 2010s, when the majority of newly bred varieties in China are *FW* resistant (Figure 2f and Figure 1d). Given the above, it is likely that the non‐synonymous SNP in *GbPP2C80* is responsible for the resistance of Sea Island cotton to *FW*.

### 2.3 *GbPP2C80* Negatively Modulates FW Resistance in *G. barbadense* and *A. thaliana*

**Figure 3:**
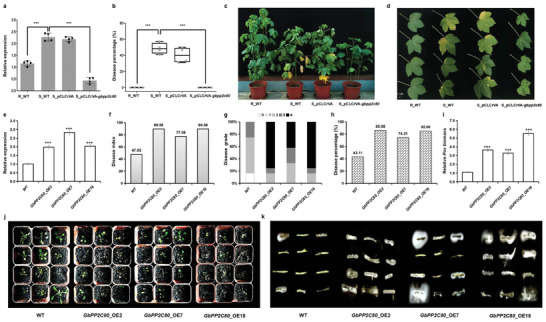
*GbPP2C80* negatively modulates the *FW* resistance of Sea Island cotton and Arabidopsis. a) qRT‐PCR analysis of the *GbPP2C80* gene in the wild type and silenced Sea Island cotton varieties. R: *FW*‐resistant variety, T10‐280. S: *FW*‐susceptible variety, II15‐3464. WT: wild type. S_pCLCrVA: *FW*‐susceptible variety containing pCLCrVA empty vector. S_pCLCrVA‐*gbpp2c80*: *GbPP2C80*‐silenced *FW*‐susceptible lines. *n *= 4 individuals. b) Disease percentage for *GbPP2C80*‐silenced Sea Island cotton individuals. *n *= 4 individuals. c,d), Phenotype of *GbPP2C80*‐silenced Sea Island cotton individuals and leaves. *n *= 4 individuals. e) Expression levels of *GbPP2C80* in wild type and overexpressed *Arabidopsis*. *n*, three biological repeats. f) Disease index of *GbPP2C80*‐overexpressed T_3_ *Arabidopsis* individuals at 10 dpi. *n *= 12 individuals. g) Disease grades of *GbPP2C80*‐overexpressed T_3_ *Arabidopsis* individuals at 10 dpi. *n *= 12 individuals. h) Disease percentage of *GbPP2C80*‐overexpressed T_3_ *Arabidopsis* individuals at 10 dpi. *n *= 12 individuals. i) Relative *Fov* biomass in wild type and *GbPP2C80*‐overexpressed *Arabidopsis* lines at 10 dpi. *n *= 12 individuals. j) Disease phenotype of *GbPP2C80*‐overexpressed T_3_ *Arabidopsis* individuals at 10 dpi. *n *= 12 individuals. k) Fungal recovery experiments. Stem segments of *GbPP2C80*‐overexpressed T_3_ *Arabidopsis* lines and WT control at 7 dpi were cut and placed on potato dextrose agar (PDA) plates and incubated at 28 °C. The photographs were taken 3 days later.

The expression differences of *GbPP2C80* between resistant variety (R, T10‐280) and susceptible variety (S, II15‐3464) containing corresponding haplotypes were analyzed. We discovered that the expression of *GbPP2C80* was higher in the susceptible variety (S_WT) than in the resistant varieties (R_WT) (**Figure** **3**a). Therefore, the *FW* susceptible Sea Island cotton variety was used as the VIGS receptor to validate the role of *GbPP2C80* on the *FW* resistance of Sea Island cotton, with S_pCLCrVA as the empty vector control and R_WT as the resistance control. Furthermore, Sea Island cotton seedlings infiltrated with different constructs were sampled for RNA extraction and qRT‐PCR analysis. The expression of *GbPP2C80* was significantly reduced in the silenced plants (S_pCLCrVA‐*gbpp2c80*) compared with S_WT and S_pCLCrVA controls (*P *< 0.001) (Figure 3a). We found that the disease percentage of S_WT control plants was ≈48.75% at 25 days post *Fov race 7* inoculation, whereas that of the silenced plants (S_pCLCrVA‐*gbpp2c80*) was 0.00, achieving the resistant level of the resistant control (R_WT) (Figure 3b). After inoculation with *Fov race 7*, the cotton seedlings of wild‐type (S_WT) and empty‐vector (S_pCLCrVA) controls exhibited more wilting and etiolated leaves than the silenced plants (S_pCLCrVA‐*gbpp2c80*) (Figure 3c,d). These findings suggested that silencing *GbPP2C80* enhanced the *FW* resistance of Sea Island cotton. Additionally, compared with the wild‐type (S_WT) and empty‐vector (S_pCLCrVA) controls, the plant height of the silenced plants (S_pCLCrVA‐*gbpp2c80*) decreased slightly but not significantly (Figure S2a, Supporting Information), while the leaf number of the silenced plants (S_pCLCrVA‐*gbpp2c80*) diminished significantly (Figure S2b, Supporting Information), implying a balance between disease resistance and plant growth.

Subsequently, we generated transgenic *Arabidopsis* lines with heterozygous expression of *GbPP2C80*. Four T_3_ transgenic lines overexpressing *GbPP2C80* were used to inoculate with *Fov* race 7 (Figure 3e). After four weeks of growth under normal conditions, the transgenic and WT plants were inoculated with *Fov7*. The disease index of WT plants reached 43.11% at 10 dpi (days post inoculation); however, that of the *GbPP2C80* overexpression lines ranged from 74.31 to 85.88% (Figure 3f). Correspondingly, the disease grade of WT plants was dominated by the second grade, while the *GbPP2C80* overexpression lines were concentrated in the fourth grade (Figure 3g). Similar to the disease index, the disease percentage in *GbPP2C80* over‐expressed lines was significantly higher than that in WT (Figure 3h). Fungal biomass analysis confirmed that more fungal biomass significantly accumulated in *Arabidopsis* overexpressing *GbPP2C80* compared to WT (Figure 3i). Notably, a more susceptible phenotype was observed in *GbPP2C80*‐overexpressed lines, with more wilting, chlorosis, early senescence, and necrosis (Figure 3j). This result was further confirmed by fungal recovery experiments (Figure 3k). Taken together, *GbPP2C80* negatively modulates the resistance to *Fov7*.

### 2.4 *GbPP2C80* and *GbWAKL14* Coexist Synergistically in Sea Island Cotton and Interact with Each Other

**Figure 4:**
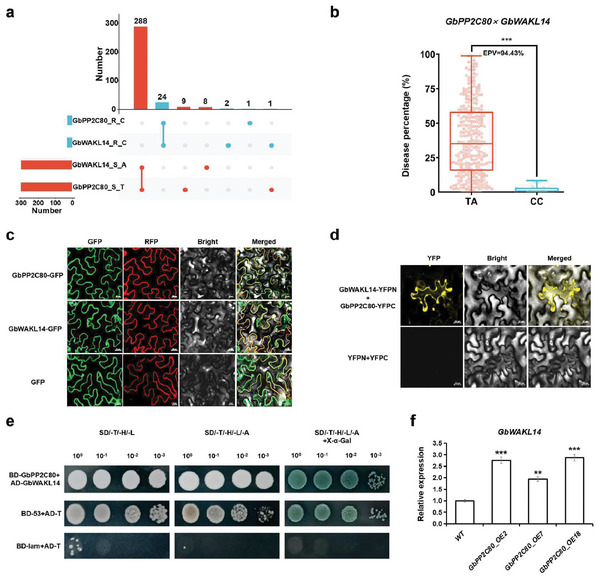
*GbPP2C80* and *GbWAKL14* coexist synergistically in Sea Island cotton and interact in *vivo*. a) The coexistence combinations of key nonsynonymous SNPs of *GbPP2C80* and *GbWAKL14* in Sea Island cotton accessions. R: resistant: S: susceptible. For *GbPP2C80*, the resistant (R) and the susceptible (S) haplotypes were C and T, respectively. For *GbWAKL14*, the resistant (R) and the susceptible (S) haplotype were C and A, respectively. b) Effect of haplotype interactions between *GbPP2C80* and *GbWAKL14* on *FW* disease percentage of Sea Island cotton. TA: susceptible haplotype combination; CC: resistant haplotype combination. c) The subcellular location of GbPP2C80 and GbWAKL14. d) BiFC validated the interaction between GbPP2C80 and GbWAKL14. e) Y2H validated the interaction between GbPP2C80 and GbWAKL14. f) The expression of *GbWAKL14* in *GbPP2C80*‐overexpressed *Arabidopsis*. *n = *6.

In our previous research, we identified a *FW*‐related gene, *GbWAKL14*,^[^ ^32^ ^]^ which is located downstream of the *GbPP2C80* gene, with a distance of 375 kb between two non‐synonymous SNPs of *GbPP2C80* and *GbWAKL14* (Figure 2a). Therefore, we would like to query if *GbPP2C80* and *GbWAKL14* can synergistically regulate *FW* resistance in Sea Island cotton. First, we analyzed the coexistence of the *GbPP2C80* and *GbWAKL14* haplotypes in Sea Island cotton accessions. Among 336 Sea Island cotton accessions, 288 (85.71%) contained both *GbPP2C80* (T) and *GbWAKL14* (A) susceptible haplotypes, and 24 (7.14%) harbored both *GbPP2C80* (C) and *GbWAKL14* (C) resistant haplotypes, and the proportion of two genes alone was less than 3% (**Figure** **4**a). We further analyzed the effects of the *GbPP2C80* and *GbWAKL14* haplotypes' interactions on the *FW* resistance. Compared with the TA type, the CC type could reduce the *FW* disease percentage by 94.43% (Figure 4b), improving the *FW* resistance by 6.89% than *GbPP2C80* alone (87.54%, Figure 2c).

Subcellular localization was performed for GbPP2C80 and GbWAKL14 using the GFP‐fused vector and tobacco transient transformation system. Both GbPP2C80 and GbWAKL14 were located on the cell membrane (Figure 4c), which is consistent with the roles of PP2C protein in dephosphorylating the membranous or cytoplasmic kinase, and WAKL14 protein in sensing and transmitting extracellular pathogen‐associated signals, respectively. Further, we validated their interaction on the membrane by bimolecular fluorescence complementation (BiFC) (Figure 4d). Yeast two‐hybrid (Y2H) also demonstrated that GbPP2C80 interacted with GbWAKL14 in vivo (Figure 4e). Additionally, we discovered the elevated expression of *GbWAKL14* in *GbPP2C80*‐overexpressed *Arabidopsis* lines (Figure 4f). In conclusion, *GbPP2C80* and *GbWAKL14* might work synergistically to defend against *Fov7* infection.

### 2.5 *GbWAKL14* Plays a Negative Role in Modulating the *FW* Resistance of Upland Cotton and *A. thaliana*

According to the gene symbols WAK and WAKL, we detected 91, 92, 69, and 69 *WAK*(*L*) genes in *G. barbadense* (Gb, HAU), *G. hirsutum* (Gh, HAU), *G. raimondii* (Gr, JGI), and *G. arboreum* (Ga, CRI) in cottonFGD (https://cottonfgd.net/). Based on 26 *WAK*(*L*) genes (*WAK1‐WAK5*, *WAKL1‐WAKL22*, no *WAKL19*) in *Arabidopsis*, we divided the *WAK*(*L*) genes in *G. barbadense* (Gb, 91), *G. hirsutum* (Gh, 92), *G. raimondii* (Gr, 69), and *G. arboreum* (Ga, 69) into 26 categories. These genes were renamed as “species abbreviation (i.e., Gb) + gene symbol (i.e., WAKL14) + chromosome number (i.e., D03) or + copy number (i.e., −3)”. Unlike other types of genes, WAKL14‐type genes were relatively conserved and clustered together in the phylogenetic tree (Figure S3, Supporting Information). *FW* resistance gene *GbWAKL14*_*D03* (in *G. barbadense*) and its homology *GhWAKL14_D03* (in *G. hirsutum*) had the closest relationship, and both originated from *GrWAKL14_3* (in *G. raimondii*). Furthermore, we aligned the CDS and protein sequences of *GbWAKL14*_*D03* and *GhWAKL14_D03* (Figure S4, Supporting Information), and found extremely high identity (99.71% for CDS, 99.28% for proteins). Six SNPs caused changes of five amino acids (AA), including the 605 amino acid (S/R) that corresponded to the key SNP (1 537 617, A/C). In *G. barbadence* (3‐79), the key SNP is A (susceptible haplotype) that corresponds to amino acid S (serine); while in *G. hirsutum* (TM‐1), the key SNP is C (resistant haplotype) that corresponds to amino acid R (arginine).

**Figure 5:**
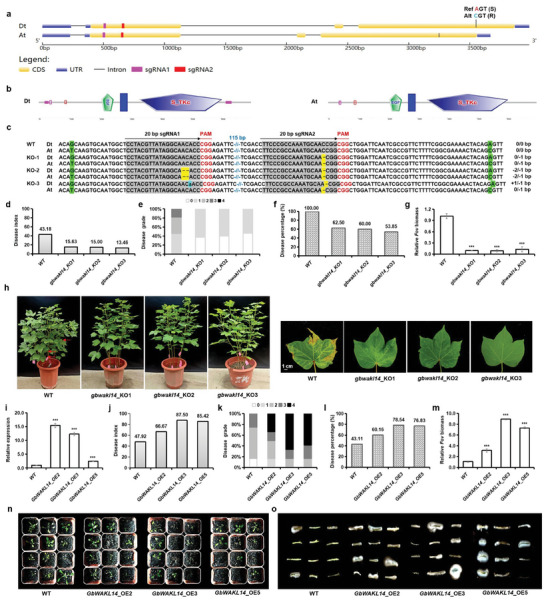
*GbWAKL14* played a negative role in modulating the *FW* resistance. a) The locations of two sgRNAs in gene *GbWAKL14*. The non‐synonymous SNP (A/C) in the third exon was marked by a black vertical line. b) The locations of two sgRNAs in protein GbWAKL14. c) The editing types of three *GbWAKL14‐*knockout (KO) lines. WT: Jin668. d) Disease index of WT and *GbWAKL14*‐knockout Jin668 lines at 35 dpi. *n *= 11, 8, 5, 14 for WT, *gbwakl14*_KO1, *gbwakl14*_KO2, *gbwakl14*_KO3, respectively. e) Disease grade of WT and *GbWAKL14*‐knockout Jin668 lines at 35 dpi. f) Disease percentage of WT and *GbWAKL14*‐knockout Jin668 lines at 35 dpi. g) Relative *Fov* biomass in WT and *GbWAKL14*‐knockout Jin668 lines at 35 dpi. h) Phenotype of *GbWAKL14*‐knockout Jin668 individuals and leaves. i) Expression level of *GbWAKL14* in WT and *GbWAKL14‐*overexpressed *Arabidopsis* individuals. j) Disease index of WT and *GbWAKL14*‐overexpressed T_3_ *Arabidopsis* lines at 10 dpi. *n *= 12 individuals. k) Disease grade of WT and *GbWAKL14*‐overexpressed T_3_ *Arabidopsis* lines at 10 dpi. *n *= 12 individuals. l) Disease percentage of WT and *GbWAKL14*‐overexpressed T_3_ *Arabidopsis* lines at 10 dpi. *n *= 12 individuals. m) Relative *Fov* biomass in WT and *GbWAKL14‐*overexpressed T_3_ *Arabidopsis* lines at 10 dpi. *n *= 12 individuals. n) Disease phenotype of WT and *GbWAKL14*‐overexpressed T_3_ *Arabidopsis* lines at 10 dpi. *n *= 12 individuals. o) Fungal recovery experiments. Stem segments of *GbWAKL14*‐overexpressed T_3_ *Arabidopsis* lines and WT control at 7 dpi were cut and placed on potato dextrose agar plates and incubated at 28 °C. The photographs were taken 3 days later.

To further verify the role of *GbWAKL14* in *FW* resistance, we used the CRISPR/Cas9 gene editing strategy to knock out the *GbWAKL14* gene in Jin668, an upland cotton variety resistant to *FW*. Since *GbWAKL14* was highly similar to its homolog in At subgenome, we selected two 20‐nt sequences in the first exon that could simultaneously target *GbWAKL14* in Dt and its homolog in At (**Figure** **5**a; Figure S5a, Supporting Information). The locations of two sgRNAs corresponded to protein sequences before all functional domains and the 605 amino acid (S/R) affected by key SNP (1 537 617, A/C; Figure 5b; Figures S4b, S5b, Supporting Information). Several transgenic lines were generated in the background of Jin668 and the mutations were identified using Hi‐TOM.^[^ ^33^ ^]^ Three knockout lines (*gbwakl14*_KO1, *gbwakl14*_KO2, and *gbwakl14*_KO3) were selected for *FW* resistance validation (Figure 5c). In *gbwakl14*_KO1, only a single‐base (C) deletion was detected in sgRNA2 of gene *GbWAKL14*. In *gbwakl14*_KO2, a two‐base deletion (AC) and a single‐base (C) deletion were observed in sgRNA1 and sgRNA2 of gene *GbWAKL14*, respectively. In *gbwakl14*_KO3, a single‐base insertion (a) and a single‐base (C) deletion were observed in sgRNA1 and sgRNA2 of gene *GbWAKL14*, respectively. Although the mutations in the above KO lines differed, they all led to frame‐shift mutations and premature termination of protein translation before all functional domains and the 605 amino acid (S/R) affected by key SNP (1 537 617, A/C; Figure S5c, Supporting Information), resulting in a complete loss of inherent function. Thus, knocking out *GbWAKL14* homolog in upland cotton has essentially the same effect as knocking out *GbWAKL14*, at least in KO lines with the editing types in this study. The three KO lines were highly resistant to *Fov7*, with almost no brown discoloration and no fungal DNA detected. In contrast, wild‐type plants showed wilt symptoms with brown, shriveled rims of leaves, and moderate disease index, disease grade, disease percentage, and a certain level of fungal DNA (Figure 5d–h). These results indicated that *GbWAKL14* played a negative role in modulating the *FW* resistance in upland cotton.

At the same time, we generated *GbWAKL14*‐overexpressed transgenic *Arabidopsis* lines. Four T_3_ *GbWAKL14*‐overexpressed lines were chosen for *FW* treatment (Figure 5i). All transgenic and WT plants were first grown in normal conditions for four weeks and then inoculated with *Fov7*. A more susceptible phenotype was observed in *GbWAKL14‐*overexpressed lines at 10 dpi, with a higher disease index, disease grade, and disease percentage (Figure 5j–l). The accumulation of fungal biomass in *GbWAKL14‐*overexpressed lines was significantly higher than in WT (Figure 5m). In terms of overall symptoms, more *GbWAKL14*‐overexpressed lines showed more wilting, chlorosis, early senescence, necrosis, and more fungi (Figure 5n,o). In summary, *GbWAKL14* negatively modulated *FW* resistance in *A. thaliana*.

### 2.6 *GbPP2C80* and *GbWAKL14* Interact with MPK3 to Regulate *GbRbohD* Expression and ROS Scavenging Enzymes Activities

**Figure 6:**
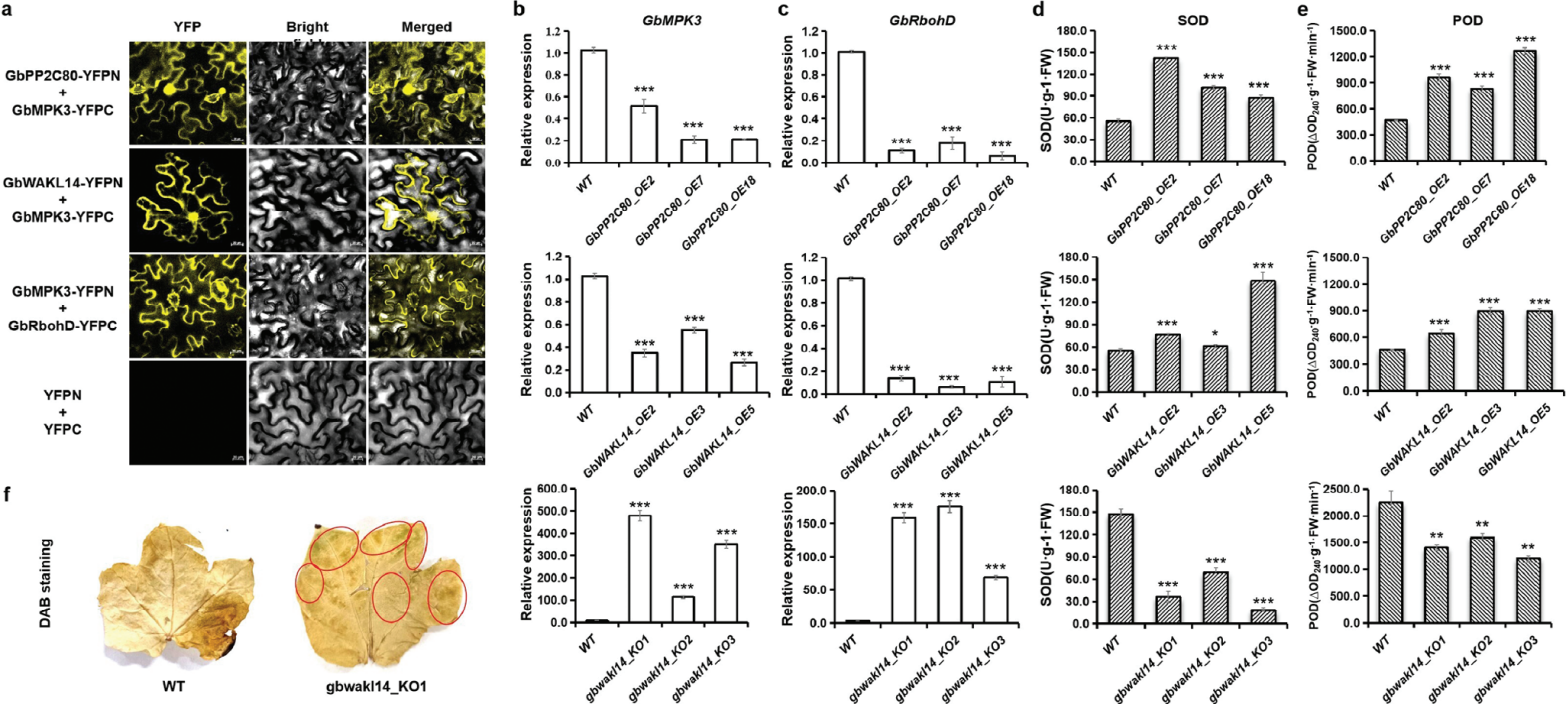
GbPP2C80 and GbWAKL14 regulated ROS production via MAPK signaling. a) BiFC validated the interactions of GbPP2C80 with GbMPK3, GbWAKL14 with GbMPK3, and GbMPK3 with GbRbohD. b) The expression level of *GbMPK3* in *GbPP2C80* (the upper panel) and *GbWAKL14* (the middle panel) overexpressed *Arabidopsis* lines, and *GbWAKL14* (the lower panel) knockout upland cotton lines. *n *= 3 individuals. c) The expression level of *GbRbohD* in *GbPP2C80* (the upper panel) and *GbWAKL14* (the middle panel) overexpressed *Arabidopsis* lines, and *GbWAKL14* (the lower panel) knockout upland cotton lines. *n *= 3. d) The activity of SOD in *GbPP2C80* (the upper panel) and *GbWAKL14* (the middle panel) overexpressed *Arabidopsis* lines, and *GbWAKL14*(the lower panel) knockout upland cotton lines after infecting with *Fov7*. *n *= 3. e) The activity of POD in *GbPP2C80* (the upper panel) and *GbWAKL14* (the middle panel) overexpressed *Arabidopsis* lines, and *GbWAKL14* (the lower panel) knockout upland cotton lines after infecting with *Fov7*. *n *= 3. f) DAB staining showing ROS content of wild type and *GbWAKL14*‐knockout upland cotton. The darker parts of the WT leaves were not caused by DAB staining but by contact with a hot pan. Regions containing brown spots in the leaves of *GbWAKL14*‐knockout upland cotton were marked with red circles.

To dissect the regulatory mechanism of GbPP2C80 and GbWAKL14 in *FW* resistance, we analyzed their relationship to MAPK and ROS‐related genes commonly involved in disease defense. First, we validated the interaction of GbPP2C80/GbWAKL14 and GbMPK3 on the nuclear and membrane, and the interaction of *GbMPK3* and *GbRbohD* on the membrane by BiFC (**Figure** **6**a). Second, we detected the expression of *GbMPK3* and *GbRbohD* in *GbPP2C80* and *GbWAKL14* transgenic lines and found that the expression levels of *GbMPK3* and *GbRbohD* were lower in *GbPP2C80/GbWAKL14* overexpressed lines than those in WT (Figure 6b), and the opposite was true in *GbWAKL14* knockout lines (Figure 6c). Third, we detected the activities of two major ROS scavenging enzymes, superoxide dismutase (SOD) and peroxidase (POD). The results showed a significant increase in *GbPP2C80/GbWAKL14* overexpression lines but an evident decrease in *GbWAKL14* knockout lines (Figure 6d,e). Finally, using DAB staining, we discovered more brown spots in *GbWAKL14* knockout cotton lines than brown spots in wild type, illuminating that ROS content and *FW* resistance of *GbWAKL14* knockout cotton lines were increased (Figure 6f). Therefore, we concluded that *GbPP2C80* and *GbWAKL14* might defend against *FW* by tuning the ROS content via the MAPK pathway.

### 2.7 *GbWAKL14* and *GbPP2C80* also Negatively Orchestrate *VW* Resistance via ROS Metabolism in Upland Cotton and *Arabidopsis*

**Figure 7:**
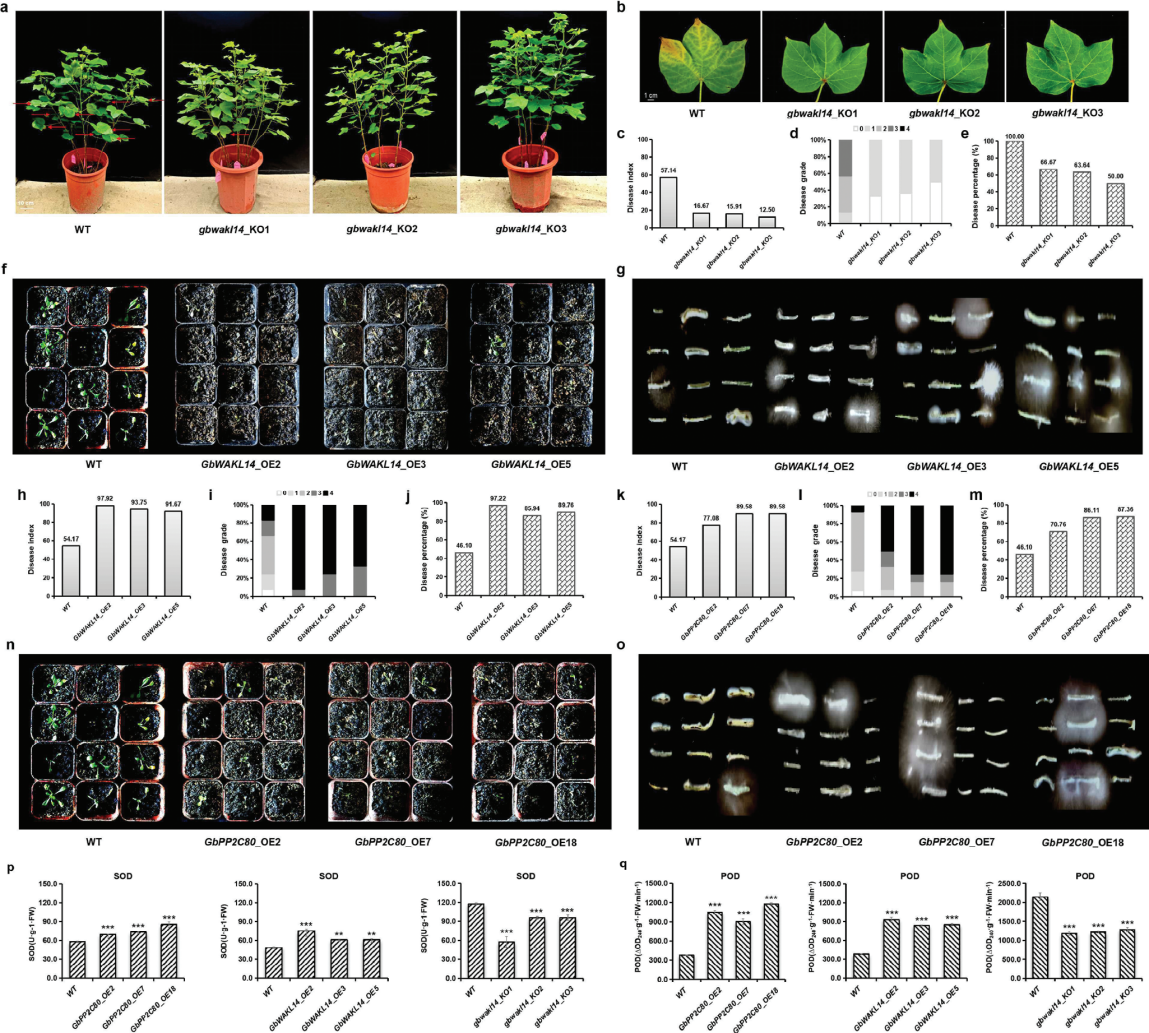
*GbWAKL14* negatively regulated *VW* resistance in upland cotton and *Arabidopsis*. a,b) Phenotypes of *GbWAKL14*‐knockout Jin668 individuals and leaves at 35 dpi. *n *= 7, 3, 22, 6 for WT, *gbwakl14*_KO1, *gbwakl14*_KO2, *gbwakl14*_KO3, respectively. c) Disease index of WT and *GbWAKL14*‐knockout Jin668 lines at 35 dpi. d) Disease grade of WT and *GbWAKL14*‐knockout Jin668 lines at 35 dpi. e) Disease percentage of WT and *GbWAKL14*‐knockout Jin668 lines at 35 dpi. f) Disease phenotype of WT and *GbWAKL14*‐overexpressed T_3_ *Arabidopsis* lines at 10 dpi. *n *= 12 individuals. g) Fungal recovery experiments of *GbWAKL14*‐overexpressed T_3_ *Arabidopsis* lines and the WT. h) Disease index of WT and *GbWAKL14*‐overexpressed T_3_ *Arabidopsis* lines at 10 dpi. *n *= 12 individuals. i) Disease grade of WT and *GbWAKL14*‐overexpressed T_3_ *Arabidopsis* lines at 10 dpi. *n *= 12 individuals. j) Disease percentage of WT and *GbWAKL14*‐overexpressed T_3_ *Arabidopsis* lines at 10 dpi. *n *= 12 individuals. k) Disease index of *GbPP2C80*‐overexpressed T_3_ *Arabidopsis* individuals at 10 dpi. *n *= 12 individuals. l) Disease grade of *GbPP2C80*‐overexpressed T_3_ *Arabidopsis* individuals at 10 dpi. *n *= 12 individuals. m) Disease percentage of *GbPP2C80*‐overexpressed T_3_ *Arabidopsis* individuals at 10 dpi. *n *= 12 individuals. n) Disease phenotype of *GbPP2C80*‐overexpressed T_3_ *Arabidopsis* individuals at 10 dpi. *n *= 12 individuals. o) Fungal recovery experiments of *GbPP2C80*‐overexpressed T_3_ *Arabidopsis* lines and the WT. p) The activity of SOD in *GbPP2C80* (the left panel) and *GbWAKL14* (the middle panel) over‐expressed *Arabidopsis* lines, and *GbWAKL14* (the right panel) knockout upland cotton lines after infection with *Vd991*. *n *= 3. q) The activity of POD in *GbPP2C80* (the left panel) and *GbWAKL14* (the middle panel) over‐expressed *Arabidopsis* lines, and *GbWAKL14* (the right panel) knockout upland cotton lines at 10 dpi. *n *= 3.

In addition, we wondered if *GbWAKL14* could also modulate the resistance of cotton to *VW*. Therefore, we infected wild‐type and CRISPR/Cas9‐edited *GbWAKL14* upland cotton (Jin668) individuals with *Verticillium dahliae* race *Vd991* and found that the *VW* resistance was greatly increased after *GbWAKL14* was knocked out (**Figure** **7**a,b). The *VW* disease index of wild type was 57.14%, while the *VW* disease index of three *GbWAKL14*‐knockout cotton lines were 16.67%, 15.91%, and 12.50%, respectively, showing a significant reduction trend (Figure 7c). The *VW* disease grades of wild type were mainly grade 2 and grade 3, while the *VW* disease grades of the *GbWAKL14*‐knockout line were mainly grade 0 and grade 1 (Figure 7d). Similar to the *VW* disease index, the *VW* disease percentage of the *GbWAKL14*‐knockout line (66.67%, 63.64%, 50.00%) was also lower than that of the wild type (100%, Figure 7e). In addition, wild‐type and *GbWAKL14*‐knockout upland cotton lines were grown in natural disease nurseries with *FW* and *VW* mixed pathogens in Kolar, Xinjiang. Significant symptoms of wilt disease were observed in the wild type and a resistant phenotype was observed in the *GbWAKL14*‐knockout cotton lines (Figure S6, Supporting Information). Additionally, we observed more susceptible phenotypes in *GbWAKL14‐*overexpressed *Arabidopsis* lines (Figure 7f–j) and *GbPP2C80‐*overexpressed *Arabidopsis* lines (Figure 7k–o), when infected with *Vd991* at 10 dpi. Furthermore, we found that the activities of two key ROS scavenging enzymes, SOD and POD, were relatively higher in *GbWAKL14‐* and *GbPP2C80‐*overexpressed *Arabidopsis* lines, but significantly lower in *GbWAKL14*‐knockout cotton lines (Figure 7p,q). To sum up, we concluded that *GbWAKL14* and *GbPP2C80* contributed to *FW* and VW resistance by regulating ROS metabolism in cotton and *Arabidopsis*.

### 2.8 Haplotype Dynamics Reveal Evolution and Improvement of the *FW* Resistance Genes *GbPP2C80* and *GbWAKL14* in Cotton

**Figure 8:**
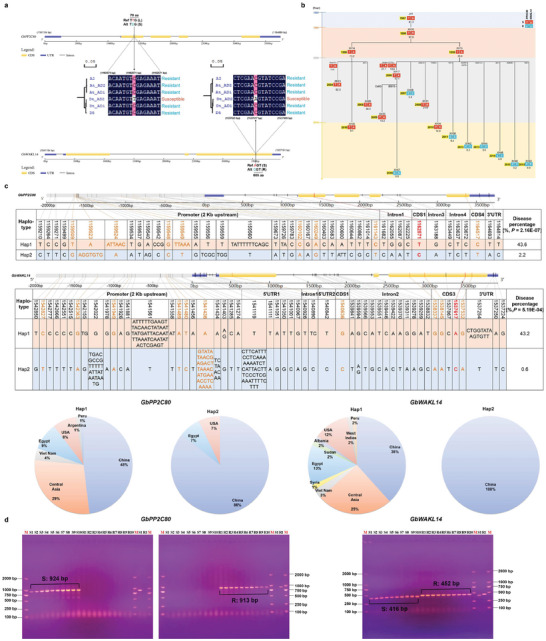
Resistant SNPs in *GbPP2C80* and *GbWAKL14* were obtained during the stages of introduction and genetic improvement of Sea Island cotton. a) The key non‐synonymous SNPs of *GbPP2C80* and *GbWAKL14* in diploid progenitors, A_2_ and D_5_, and in two tetraploid cultivated cotton species, AD_1_ and AD_2_. b) The resistant haplotype combination of *GbPP2C80* and *GbWAKL14* was selected primarily after the 2010s in the pedigree improvement of Sea Island cotton. R: the resistant haplotype C for both *GbPP2C80* (the former) and *GbWAKL14* (the latter), shown in blue. S: the susceptible haplotype T for *GbPP2C80* (the former) and A for *GbWAKL14* (the latter), shown in orange. The number in the yellow box in front of the haplotype was the year in which the variety was bred. The timeline on the left and the modules in different colors covered the varieties cultivated in the corresponding years. c) Resistant and susceptible haplotype blocks formed by SNPs and InDels in gene regions and 2 Kb promoter regions, and the geographical distribution of *GbPP2C80* and *GbWAKL14*. Variations marked with yellow in the promoter region could cause changes in the promoter elements. Variations marked with yellow in the gene region were those in the CDS regions. Variations marked with red in CDS were the key non‐synonymous SNPs (consistent with Figure 2). The difference in disease percentage between the two major haplotype blocks was extremely significant (*P *< 0.001). d) PCR distinguishing resistant and susceptible Sea Island varieties using SNP and InDel variations of *GbPP2C80* (left) and *GbWAKL14* (right). For *GbPP2C80*, we designed susceptible (Tm, 60.0 °C) and resistant (Tm, 62.0 °C) specific primers based on the deletion of 11 bp in the promoter region of resistant varieties. For *GbWAKL14*, susceptible and resistant varieties were distinguished based on the insertion of 36 bp in the promoter region of resistant varieties using only one pair of primers (Tm, 57.0 °C). The sequences of these two sets of primers are listed in Table S6 (Supporting Information). S1–S10, 10 extremely susceptible Sea Island cotton varieties; R1–R10, 10 extremely resistant Sea Island cotton varieties (Table S7, Supporting Information).

To understand the evolutionary origin of the *FW* resistance genes *GbPP2C80* and *GbWAKL14*, a phylogenetic tree was constructed using homologous CDS sequences from A_2_, D genome diploid, and AD genome tetraploid cotton species. *GbPP2C80* and *GbWAKL14* in *G. barbadense* (AD_2_) were most closely related to those in tetraploid *G. darwinii* (AD_5_), and diploid D genome cotton species (Figure S7, Supporting Information). Furthermore, to explore the evolution origin of susceptible haplotypes (nonsynonymous SNPs) in *GbPP2C80* and *GbWAKL14*, we extracted the simplified tree containing homologous CDS sequences of A_2_, At_AD_2_, At_AD_1_, Dt_AD_2_ (candidate genes), Dt_AD_1_, and D_5_ (Figures S8,S9, Supporting Information). Notably, the susceptible SNPs that caused the shift of amino acids were only present in Dt_AD_2_, suggesting the susceptible haplotypes for *GbPP2C80* and *GbWAKL14* were not derived from the diploid progenitors, A_2_ and D_5_, but were acquired after the divergence of tetraploid cultivated cotton species, AD_1_ and AD_2_ (**Figure** **8**a). Presumably, the key susceptible SNPs of the genes *GbPP2C80* and *GbWAKL14* were generated before the introduction and were selectively swept during the improvement of Sea Island cotton. JH1 was the first Sea Island cotton variety to be independently cultivated in China in 1953. Using it as a backbone parent, more than 70 Sea Island cotton varieties of the Xinhai series were gradually bred. Here a pedigree composed of Xinjiang (China)‐self‐bred Sea Island cotton varieties was chosen to analyze the dynamics of key susceptible/resistant SNPs in *GbPP2C80* and *GbWAKL14*. The results showed that the key SNPs of *GbPP2C80* and *GbWAKL14* existed in fixed combinations, susceptible–susceptible (TA) or resistant–resistant (CC), in Sea Island cotton pedigree varieties (Figure 8b). In addition, the resistant–resistant (CC) combination first emerged in the 2000s and dominated in the 2010s, consistent with a declining trend of disease percentage (Figure 1d).

In addition, we further detected all SNPs and InDels in the 2 Kb promoter and gene regions of *GbPP2C8*0 and *GbWAKL14*. For *GbPP2C8*0, there were 20 SNPs and 11 InDels that influenced regulatory elements in the promoter region, 8 SNPs in the intron, 2 SNPs in exon, and 2 SNPs in 3′ untranslated region (UTR), respectively (Figure 8c, the upper panel). These variations formed two major haplotypes, susceptible Hap1 (mean disease percentage 43.6%) and resistant Hap2 (mean disease percentage 2.2%), which coincided with the key nonsynonymous SNPs, T and C. Hap1 existed in Sea Island cotton production regions from all over the world while Hap2 was present only in USA, Egypt, and China (Figure 8c, the lower panel), implying that the resistant haplotype was introduced into China from USA and Egypt. For *GbWAKL14*, 16 SNPs and 7 InDels occurred in the promoter region, 6 SNPs and 1InDel happened in 5′ UTR, 12 SNPs and 2 InDel arose in introns, 6 SNPs turned up in exons, and 2 SNPs and 1 InDel came out in 3′UTR (Figure 8c, the middle panel), resulting in two major haplotypes, Hap1 and Hap2, with disease percentage averaging 43.2% and 0.6%, respectively, which were consistent with the category based on the key nonsynonymous SNPs, susceptible A and resistant C. Interestingly, the resistant haplotype of GbWAKL14 was unique to Chinese Sea Island cotton varieties (Figure 8c, the lower panel), suggesting that it was generated during the improvement of Sea Island cotton in China. Additionally, the variations in the promoter and gene regions (UTR, exon, and intron) of *GbPP2C80* and *GbWAKL14* demonstrated that they might affect disease resistance at the DNA, mRNA, and protein levels. In this study, VIGS and overexpression assays verified their roles at the mRNA level, and CRISPR editing confirmed their functions at the DNA and protein levels. In addition, we have developed molecular markers based on the InDel and SNP variations of *GbPP2C80* and *GbWAKL14* to distinguish resistant and susceptible Sea Island cotton varieties, facilitating effective and efficient molecular markers‐assisted selection of resistant Sea Island cotton varieties (Figure 8d).

## 3 Discussion

### 3.1 Protein Phosphatases and Wall Associated Receptor‐Like Kinases Regulate Wilt Resistance by Modulating MAPK Signaling and ROS Metabolism

Cotton responses to wilt disease by four kinds of genes: 1) genes encoding receptor‐like proteins/kinases that recognize and bind to extracellular pathogens and then transmit signals downstream; 2) genes involved in signal transduction, primarily mitogen‐activated protein kinase (MAPK) cascades that sort and amplify external signals into intracellular signals; 3) transcription factors that interact with RNA polymerase to influence the transcription initiation of the resistant genes; 4) defense‐related genes that either strengthen the cell wall or accumulate ROS levels.^[^ ^34^ ^]^

*GbWAKL14* belongs to the first class of genes that encode receptor‐like kinases, so we hypothesized that GbWAKL14 was capable of recognizing and binding to extracellular pathogens and then transmitting the signal downstream. To explore the role of *GbWAKL14*, we predicted the domains of GbWAKL14 and found that it contained extracellular epidermal growth factor motif and transmembrane region (Figure 5b); in addition, GFP‐fused vector containing *GbWAKL14* showed its location on the membrane (Figure 4d). Furthermore, the CRISPR/Cas9 knockout of *GbWAKL14* validated its negative role in cotton resistance to *FW* and *VW* (Figure 5 and Figure 7). As for the underlying molecular mechanism, *CsWAKL08* conferred resistance to bacterial canker by regulating ROS in citrus;^[^ ^22^ ^]^ OsWAKL21.2 affected rice resistance using its kinase domain;^[^ ^23^ ^]^ GhWAK7A orchestrated the resistance of upland cotton to *VW* and *FW* by interacting with and phosphorylating a lysin‐motif receptor kinase, GhLYK5;^[^ ^9^ ^]^ GhWAKL with a mutation in the phosphorylation site Ser^628^ reduced cotton resistance to *VW*.^[^ ^24^ ^]^ Based on these studies, we hypothesized that GbWAKL14 regulated cotton resistance to *FW* and *VW* by controlling ROS and its kinase domain, with the involvement of phosphatases. Here, we validated that GbWAKL14 could affect ROS content by interacting with GbMAPK3 (Figure 6), and it did interact with a protein phosphatase type 2C, GbPP2C80 (Figure 4e,f).

Protein phosphatase type 2C (PP2C) acts as a key negative regulator of plant immunity mainly by dephosphorylating receptor‐like proteins/kinases. Type 2C protein phosphates PLL4 and PLL5 affected *Arabidopsis* immunity by dephosphorylating receptor kinases FLS2 and EFR.^[^ ^26^ ^]^ Type 2C protein phosphatase Pic1 activates tomato immune by dephosphorylating the receptor‐like cytoplasmic kinase Pti1 and reducing ROS.^[^ ^28^ ^]^ Protein phosphatase 2C 70interacted with receptor‐like proteins RLP37, RPP13, and RPS2 to induce powdery mildew resistance in wheat.^[^ ^29^ ^]^ These demonstrated the authentic interaction of WAK(L)s and PP2Cs in the resistance pathway. In addition, protein phosphatase GhAP2C1 and GhMPK4 synergistically regulated the resistance of upland cotton to *FW*.^[^ ^11^ ^]^ Similarly, we verified that GbPP2C80 regulated *FW* and *VW* resistance by interacting with GbMPK3 and regulating the expression of *GbMPK3* and *GbRbohD* (Figure 6 and Figure 7).

**Figure 9:**
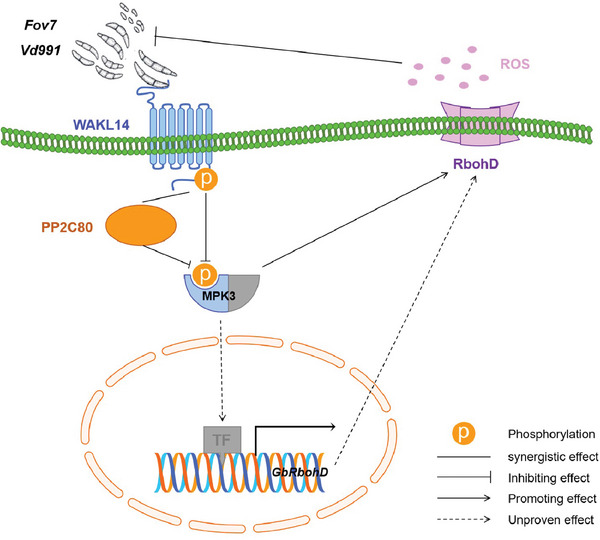
Model diagram shows the molecular mechanism by which *GbPP2C80* and *GbWAKL14* co‐regulate the *FW* and *VW* resistance. The protein GbWAKL14 senses *Fov7* and *Vd991* signals on the cell membrane and transfers them into the cell by regulating the expression of the gene *GbMPK3*. Both GbWAKL14 and GbMPK3 are affected by the protein phosphatase GbPP2C80. GbMPK3 further modulates the expression of *GbRbohD* by directly interacting with GbRbohD or by activating transcription factor (TF, i.e., WRKY40) to affect *GbRbohD* expression, increasing ROS content to resist invasion and transmission of *FW* and *VW* pathogens. GbPP2C80 is synergistic with GbWAKL14 (connected by a straight line) and antagonistic with GbMPK3 (connected by a flat‐headed arrow). GbMPK3 is synergistic with GbRbohD, but their relationship needs further exploration (connected by a pointed arrow).

In summary, we drew a functional schematic diagram to illustrate the *FW* and *VW* resistance mechanisms underlying *GbPP2C80* and *GbWAKL14* (**Figure** **9**). GbWAKL14 on the cell membrane senses the signals of *FW* and *VW* pathogens (*Fov7* and *Vd991*) and transmits them into the cell by the downstream MAPK pathway (in this study, mainly *GbMPK3*); in addition, GbWAKL14 and GbMPK3 are affected by the protein phosphatase GbPP2C80. Subsequently, GbMPK3 regulates the expression of *GbRbohD* by directly interacting with GbRbohD (in this study) or activating transcription factors (TF, i.e., WRKY40,^[^ ^12a^ ^]^) to indirectly influence the expression of *GbRbohD*, resulting in accumulation of ROS content to defense against *FW* and *VW* pathogens. Based on haplotype analysis and transgenetic validation, we found that GbPP2C80 and GbWAKL14 had a synergistic effect on *FW* and *VW* resistance. The expression levels of *GbMPK3* and *GbRbohD* were lower in *GbPP2C80* and *GbWAKL14* overexpressed plants, but higher in *GbWAKL14* knockout plants, suggesting that GbPP2C80 and GbWAKL14 were antagonistic to GbMPK3 and GbRbohD in response to *FW* and *VW*.

### 3.2 A Gene Cluster on Chromosome D03 is Responsible for *FW* Resistance in Cotton

Although there have been few studies of *FW*‐resistant QTLs/genes in Sea Island cotton, candidate QTLs/genes on chromosome D03 have been repeatedly reported. The first report *Fov 1*‐resistance QTL was located on chromosome 16 (D03) of the resistant Sea Island cotton variety Pima S‐7.^[^ ^15^, ^35^ ^]^ The *Fov 4* gene appeared to be located near a genome region on chromosome 14, and more than one gene and gene interactions across six linkage groups/chromosomes (3, 6, 8, 14, 17, and 25) were involved in the inheritance of FOV race 4 resistance;^[^ ^36^ ^]^ additionally, three *Fov4*‐targetedQTLs were mapped on chromosomes c17, c24, and c25 of resistant Sea Island cotton cultivar Pima S‐6;^[^ ^16^ ^]^ Recently, 24 FOV4 resistance QTLs were detected on A01, A02, A03, A04, A05, A06, A07, and A11, including 3 major QTLs on chromosomes A04, A06, and A11, by GWAS of 246 *G. arboreum* accessions.^[^ ^37^ ^]^ Meanwhile, a QTL targeting *Fov7* resistance (*qFOV7‐D03‐1*) was mapped on chromosome D03 of resistant Sea Island cotton cultivar 06–146, from which a gene *GB_D03G0217* (*GbCML*) encoding calmodulin‐like protein was identified, and VIGS silencing increased the severity of the disease.^[^ ^17^ ^]^ In our previous study, we also identified two functional genes, *GbDP1* (also called *GbZHD6*) and *GbDP2* (also called *GbWAKL14*), which were related to *FW* resistance on D03 chromosome;^[^ ^32^ ^]^ in this study, we identified a novel *FW* resistance gene, *GbPP2C80*, on chromosome D03, which interacted with GbWAKL14 and negatively regulated *FW* resistance like *GbWAKL14*. Interestingly, we found 99.42% (2384/2398) of *FW*‐associated SNPs, ranging from 220, 809 bp to 2, 368, 579 bp, were located on chromosome D03 (Table S3, Supporting Information). According to the 388 kb LD region of our Sea Island cotton population,^[^ ^32^ ^]^ the QTL interval was usually located 388 kb upstream and downstream of the SNP, a continuous large QTL (0 – 2, 756, 579 bp, *qFW‐D03*) was formed on D03 due to the overlap of QTLs (Figure S10a, Supporting Information). Based on the *P* value and dataset repeats that SNP is detected, we screened for a set of core SNPs and then defined the genes closest to the core SNPs and containing non‐synonymous mutations that significantly affect the *FW* phenotype as candidate genes. Combined with transgenetic validation, we obtained 3 functional genes, *GbDP1*, *GbDP2* (*GbWAKL14*), and *GbPP2C80*, all of which negatively regulated *FW* resistance. The *GbCML* gene in *qFOV7‐D03‐1* identified by 110 *G. barbadense* lines obtained by crossing susceptible Xinhai 14 with resistant 06–146 was also located in our QTL (*qFW‐D03*) region, but unfortunately, the haplotype shift of *GbCML* did not cause the significant change in *FW* disease percentage (Figure S10b, Supporting Information). These results indicated the presence of multiple *FW* resistance genes in different Sea Island cotton varieties. From this, we speculated that there was a functional module on D03 consisting of multiple genes that work together to synergistically regulate FW resistance.

In our previous study, the *FW* resistance gene *GbWAKL14* was identified by GWAS on 336 Sea Island cotton accessions, and its negative effect on the *FW* resistance of Sea Island cotton was validated by VIGS.^[^ ^32^ ^]^ In light of this, we would like to further elucidate the regulatory mechanism responsible for the resistance of Sea Island cotton to *FW*. First, we used the online software STITCH (http://stitch.embl.de/) to predict the interacting protein of GbWAKL14 using its homology in *Arabidopsis*. Almost all interacting proteins of GbWAKL14 were found to be type 2C protein phosphatases or contain PP2C domains (Figure S11a, Supporting Information), suggesting that GbWAKL14 might function by interacting with PP2C proteins through phosphorylation or dephosphorylation pathways. Similarly, another study reported that in the absence of ligands (PAMPs), signaling by plant receptors (leucine‐rich repeat‐receptor kinases FLS, EFR, and BAK1) for pathogen‐associated molecular patterns (PAMPs) in immunity would be inhibited by association with specific type2C protein phosphatases (PLL4 and PLL5); upon activation, the PAMP receptors phosphorylated different cytosolic kinases (BIK1and PBL1), which in turn phosphorylated the phosphatases, thereby promoting receptor signaling.^[^ ^26^ ^]^ We expected that a member of the PP2C family should work together with *GbWAKL14* to confer *FW* resistance to cotton. We therefore re‐analyzed the GWAS data to mine for PP2C genes that were significantly associated with *FW* resistance. Fortunately, we discovered the gene *GbPP2C80*, whose exon had a nonsynonymous SNP (T/C) that caused the change in one amino acid (Figure 2b), which is significantly associated with *FW* disease percentage (Figure 2c). The *GbPP2C80* gene was located 372 kb upstream of the *GbWAKL14* gene (Figure S10, Supporting Information), and the PP2C80 protein was a core interacting protein of WAKL14 (Figure S11a, Supporting Information). During the breeding and improvement of Sea Island cotton, the resistant haplotype of *GbPP2C80* was always accompanied by the resistant haplotype of *GbWAKL14*. Given the above, we decided to further validate the role of *GbPP2C80* in *FW* resistance and its interaction with GbWAKL14 to elucidate the regulatory network of plant resistance to wilt diseases.

For *GbZHD6*, another *FW* resistance gene identified in our previous study, we also predicted its interacting proteins (Figure S11b, Supporting Information). In the next step, we will consider mining homologous proteins significantly associated with *FW* resistance in the QTL (*qFW‐D03*) region of Sea Island cotton. Additionally, soybean zinc finger homeodomain proteins GmZF‐HD1 and GmZF‐HD2 could bind to two repeats of ATTA homeodomain binding site in the promoter of calmodulin isoform 4 (GmCaM4) under pathogen induction.^[^ ^38^ ^]^ Therefore, zinc‐finger homeodomain protein 6 (GbZHD6) may bind to the promoter of calmodulin‐like protein *GbCML* (*qFOV7‐D03‐1*) in response to Sea Island cotton to *Fov7* invasion. In the future, further analysis and functional studies of these genes should be conducted in this module.

### 3.3 Upland Cotton and Sea Island Cotton have Some Similar and Different *FW* Resistance Genes

*Fusarium wilt* has a huge dampening effect on cotton production. To date, a number of *FW* resistance genes have been screened in upland cotton and Sea Island cotton, and there were a few types of genes, such as receptor‐like kinase genes and protein phosphatase genes, were known to play a role in both cotton species. For receptor‐like kinase genes, *GhWAK7A* positively regulated upland cotton (*G. hirsutum*, AD_1_) resistance to *FW* by interacting with another receptor‐like kinase GhLYK5 and GhCERK1 and affecting the expression of *GhMPK3*.^[^ ^9^ ^]^ In our study, *GbWAKL14* negatively regulated the *FW* resistance of Sea Island cotton, and we found that it did not interact with GbLYK5 and GbCERK1 through STRING prediction but interact with GbMPK3 validated by BiFC), implying the opposite regulatory patterns but similar downstream MAPK signaling pathways between the *FW*‐resistance mechanisms in upland cotton and in Sea Island cotton. For protein phosphatase genes, *GhAP2C1* negatively regulated the immune response of upland cotton to *FW*.^[^ ^11^ ^]^ In this study, *GbPP2C80* also showed a negative regulation pattern in the defense of Sea Island cotton against *FW*, consistent with its conservative role as negative regulators in the immune responses of *Arabidopsis*,^[^ ^26^ ^]^ tomato,^[^ ^27^, ^28^ ^]^ and wheat.^[^ ^29^ ^]^ Additionally, GhAP2C1 interacted with GhMPK4, but GbPP2C80 did not interact with GbMPK4, but with GbMPK3, demonstrating that the PP2C genes transmitted signals to different MAPK genes in upland cotton and Sea Island cotton.

While similar resistance genes exist in different cotton species, most of the resistance genes identified so far are specific to one cotton species. More *FW* resistance genes were found in upland cotton compared to other cotton species. In addition to the receptor‐like kinases, a number of receptor‐like proteins have also been reported to be associated with *FW* resistance in upland cotton, such as GhRLPGSO1‐like and GhRLP44, GhRLP6, GhRLP34 and GhRLP20, and GhGLR4.8.^[^ ^3^, ^10^ ^]^ In addition, there are many MAPK cascades‐related genes, such as *GhMKK4* and *GhMPK20*,^[^ ^12a^ ^]^ *GhMKK6* and *GhMPK4*,^[^ ^12b^ ^]^ and other defense‐related proteins, including GhPLP2,^[^ ^39^ ^]^ GhGLP2,^[^ ^1^ ^]^ GhECR,^[^ ^14^ ^]^ and caffeic acid 3‐Omethyltransferase and peroxidase2.^[^ ^40^ ^]^ In Sea Island cotton, there are also a few other kinds of *FW* resistance genes, including calmodulin‐like protein gene *GB_D03G0217*,^[^ ^17^ ^]^ chalcone isomerase gene GbCHI01,^[^ ^19^ ^]^ glucosyltransferase gene *GbUGT73C1* ^[^ ^20^ ^]^ and *GbGSTU7*.^[^ ^21^ ^]^ From the above, we can see that there were relatively fewer *FW* resistance genes in Sea Island cotton than in upland cotton, which limited the understanding of the similarities and differences in the *FW* resistance genes and mechanisms in these two allotetraploid cotton species and their breeding applications. Therefore, more work needs to be done in future studies on the *FW* resistance of Sea Island cotton.

### 3.4 Similarities and Differences Exist in the Genes and Mechanisms of Cotton Resistance to *FW* and *VW*

*Fusarium* and *Verticillium wilt* are both induced by soil‐borne fungi, so it is possible that there are some identical or similar loci, genes, and mechanisms that influence both types of wilt diseases in cotton. MQTL containing both *FW* and *VW* hotspots were found on chromosomes At03, At06, At07, At08, At12, Dt02, Dt03, Dt04, Dt05, Dt08, Dt09, Dt11, and Dt13.^[^ ^41^ ^]^ In this study, two *FW* and *VW* genes, *GbPP2C80* and *GbWAKL14*, were mapped on D03. The wall‐associated kinase GhWAK7A mediated the responses of upland cotton to *Fov* and *Vd* by complexing with the chitin sensory receptors GhLYK5 and GhCERK1.^[^ ^9^ ^]^ In the present study, *GbWAKL14*, encoding the wall‐associated receptor kinase‐like 14, regulated the resistance of Sea Island cotton to *Fov* and *Vd*, but we predicted no interaction between WAKL14 and LYK5/CERK1 (Figure S12a, Supporting Information). These different mechanisms might partly explain the discrepancy between GhWAK7A being a positive regulator but GbWAKL14 being a negative factor. The germin‐like protein GhABP19 positively modulated the resistance of upland cotton to *Fov* and *Vd* through its SOD activity and JA pathway.^[^ ^30^ ^]^ In the present study, the resistance mechanisms of *GbPP2C80* and *GbWAKL14* were also associated with SOD activity. In addition, *GhBsr‐k1* (broad‐spectrum resistance Kitaake‐1) negatively regulated the resistance of upland cotton to *Fov* and *Vd* by up‐regulating the expression of lignin synthesis and signal response‐related genes (*GhPAL2* and *GhPAL5*) through phenylpropanoid metabolism and further increasing xylem development and locally lignin accumulation.^[^ ^31^ ^]^ WRKY transcription factor GhWRKY40 was a negative factor for *FW* resistance of upland cotton,^[^ ^12a^ ^]^ and GbWRKY1 negatively regulated *VW* resistance in Sea Island cotton.^[^ ^42^ ^]^ Glutathione transferases GbGSTU7 positively modulated *FW* resistance in Sea Island cotton,^[^ ^21^, ^43^ ^]^ GaGSTF9 positively regulated *FW* resistance in *G. arboreum* and *VW* resistance in *A. thaliana*.^[^ ^44^ ^]^

Moreover, even genes from the same family may have different functions or mechanisms in cotton resistance to *FW* and *VW*. Mitogen‐activated protein kinase cascade GhMKK4‐GhMPK20 played negative roles in the resistance of upland cotton to *FW*,^[^ ^12a^ ^]^ while GhMKK4, GhMKK6, and GhMKK9 played positive roles in the resistance of upland cotton to *VW*.^[^ ^45^ ^]^ The protein phosphatase GhAP2C1 negatively modulated the response of upland cotton to *FW* by antagonistically interacting with GhMPK4.^[^ ^11^ ^]^ Here, we found that MPK3, but not MPK4, was the direct downstream of the protein phosphatase PP2C80 and the wall‐associated receptor kinase WAKL14 (Figure S12b, Supporting Information), and we validated the antagonistic interaction between PP2C80/WAKL14 and MPK3 (Figure 6). However, another protein phosphatase, GhPP2C52, might be a positive regulator of *VW* resistance.^[^ ^46^ ^]^

Most of the identified genes were specific for *VW* resistance in cotton, and their mechanisms involved MAPK signaling pathways, ROS, transcription factors, hormones, and cell wall components. The cytochrome P450 gene *GbCYP72A1* positively affected *VW* resistance through plant hormone signal transduction, plant‐pathogen interaction, and mitogen‐activated protein kinase (MAPK) signaling pathways.^[^ ^47^ ^]^ Myo‐inositol oxygenase GbMIOX5^[^ ^48^ ^]^ and Hen egg white lysozyme HEWL^[^ ^49^ ^]^ positively regulated the resistance of Sea Island cotton to *VW* through ROS metabolism. Transcription factors GbVIP1^[^ ^50^ ^]^ and GbNAC1^[^ ^51^ ^]^ negatively modulated *VW* resistance in Sea Island cotton, while GbbHLH171^[^ ^52^ ^]^ was a positive factor.GhHB12^[^ ^53^ ^]^ and GhBLH7‐D06^[^ ^54^ ^]^ had negative effects on *VW* resistance in upland cotton, while GhMYB108,^[^ ^55^ ^]^ GhWRKY53,^[^ ^56^ ^]^ andRVE2^[^ ^57^ ^]^ played positive roles. Through the salicylic acid (SA) signaling pathway, ribosomal protein GaRPL18^[^ ^58^ ^]^ and isochorismate synthase GhICS2A^[^ ^59^ ^]^ positively regulated *VW* resistance, while Ca^2+^‐independent calmodulin‐binding protein GhIQM1 showed a negative effect.^[^ ^60^ ^]^ By regulating the jasmonate acid (JA) signaling pathway, cyclin‐dependent kinase GhCDKE exposed positive and negative effects on *VW* resistance,^[^ ^61^ ^]^ while calcium‐dependent protein kinase GhCPK33 and GhBIN2 played negative roles on *VW* resistance^[^ ^48^, ^62^ ^]^ The strigolactone biosynthesis genes *GbCCD7* and *GbCCD8b* positively regulated *VW* resistance by crosstalk with JA and ABA signaling pathways and inducing ROS accumulation.^[^ ^63^ ^]^ *Gh4CL3*, a gene involved in lignin and flavonoid biosynthesis, was a positive regulator of *VW* resistance by promoting JA signaling‐mediated enhancement of cell wall lignification.^[^ ^64^ ^]^ The multicopper oxidases GbAO and GbSKS participated in *VW* resistance by regulating cell wall components including pectin and lignin.^[^ ^65^ ^]^

There have been relatively few studies of *FW* resistance in cotton, mainly related to phenylpropanoids and flavonoids. Phenylpropanoid biosynthesis and phenylalanine metabolism were crucial for *FW* resistance in upland cotton, and caffeic acid 3‐O‐methyltransferase, peroxidase2, and two transcription factors (MYB46 and MYB86) affected lignin accumulation and synthesis.^[^ ^40^ ^]^ Correspondingly, the phenylpropanoid metabolism of Sea Island cotton was enhanced, and the phenylalanine ammonia‐lyase 2 (PAL2) and pleiotropic drug resistance 12 (PDR12) transporter were up‐regulated.^[^ ^66^ ^]^ Sulfotransferase GBSOT4 enhanced the resistance of Sea Island cotton to *FW* by regulating flavonoid content.^[^ ^67^ ^]^ The chalconeisomerase genes *GbCHI01*, *GbCHI05*, *GbCHI06*, and *GbCHI09* regulated flavonoid homeostasis via SA and MeJA, synergistically acting on *FW* resistance in Sea Island cotton.^[^ ^68^ ^]^ Flavonoid 3′‐hydroxylase had a synergistic effect with *GbCHI* and *GbDFR* genes to enhance the resistance of Sea Island cotton to *FW*.^[^ ^69^ ^]^ Cinnamate‐4‐hydroxylase GbC4H mediated MeJA and SA signaling pathways, regulated downstream genes to accumulate flavonoids, and ultimately inhibited the occurrence of *FW* in Sea Island cotton.^[^ ^70^ ^]^ Additionally, the anthocyanidin reductase Gb_ANR‐47 enhanced the *FW* resistance of Sea Island cotton by modulating the content of proanthocyanidins.^[^ ^71^ ^]^ As a result, the genes and mechanisms of *FW* resistance are very limited and therefore deserve more exploration.

## 4 Conclusion

Wilt diseases threaten cotton production around the world. Sea Island cotton is known for its superior fiber but is less resistant to *FW*. It is important to search for elite resistance genes and dissect the resistance mechanisms in the selection and breeding of resistant cotton varieties. In this study, a pair of genes, *GbPP2C80* and *GbWAKL14*, have been identified by GWAS on chromosome D03 to negatively co‐regulate *FW* resistance in Sea Island cotton. Transgenetic experiments have been performed on Sea Island cotton, *Arabidopsis*, and upland cotton to validate their roles in wilt disease resistance through silencing, overexpression, and knockout. *GbPP2C80* interacted with *GbWAKL14* to regulate *FW* and *VW* resistance by antagonistically affecting MPK3 and ROS signaling. *GbPP2C80* and *GbWAKL14* originated from the D genome, and the resistant haplotype combination (CC) were obtained during the introduction and improvement of Sea Island cotton. Haplotype blocks consisting of SNPs and InDels in *GbPP2C80* and *GbWAKL14* were used to develop two sets of molecular markers for rapid and efficient screening of resistant cotton varieties. This study provides new *FW* and *VW* resistance genes to help better understand cotton breeding improvement.

## 5 Experimental Section

### Plant Materials

336 Sea Island cotton (*Gossypium barbadense*) accessions derived from major global cotton‐growing countries were planted in a natural disease nursery in Korla, Xinjiang province, for DP phenotyping, and preserved at China Agricultural University, Beijing, China. Wild typehighlysusceptible (S) Sea Island accession II15‐3464 and highly resistant (*R*) Sea Island accession T10‐280 were planted in large pots with nutritive soil and vermiculite (V: V = 2: 1) in Beijing, for VIGS assay of *GbPP2C80*. Wild type and *GbWAKL14*‐knockout cotton generated from upland cotton (*G. hirsutum*) accession Jin668 were planted in a natural disease nursery in Korla (Xinjiang), in large pots in Beijing for DP investigation, and in a field in Heijian (Hebei) for propagation. Cotton plants were sown in mid‐to‐late April and harvested in mid‐to‐late October in Xinjiang, Beijing, and Hejian. Columbia *Arabidopsis thaliana* was used as transformation receptors of the *GbPP2C80 and GbWAKL14* overexpression vectors. Wild type (Col‐0), *GbPP2C80‐* and *GbWAKL14*‐overexpressed *Arabidopsis* were grown in 1/2 MS medium and transferred into small pots with nutritive soil and vermiculite (V: V = 2: 1) when the roots and leaves grow out, at 20 °C underlong‐day conditions (16 h/8 h light/dark and 60% humidity) in growth chamber of green house, in Beijing. The tobacco (*Nicotiana benthamiana*) was used for subcellular localization and interaction validation. The seeds of cotton were grown in small pots with nutritive soil and vermiculite (V: V = 2: 1), and the seedlings were grown in a greenhouse under long‐day conditions (16 h/8 h light/dark, 25–28 °C, and 60% humidity).

### Pathogen Cultivation, Plant Inoculation and Disease Assay

The *Fusarium oxysporum*f. sp. *vasinfectum*race 7 (*Fov race 7*) (Guiliang Jian, Institute of Plant Protection, Chinese Academy of Agricultural Sciences) and *Verticillium dahliae* Kleb *Vd991* (Ping Liu, China Agricultural University) were grown on potato dextrose agar plates (PDA, Difco) for 4 days at room temperature (25 °C) for *Vd991*, and 28 °C for *Fov7*. The hyphae of *Fov7* and *Vd991* were inoculated in potato dextrose broth (PDB, AoBoXing Bio‐Tech Co., Ltd, Beijing, China) and incubated in a shaker (120 rpm) at 28 °C and 25 °C for 5 days. After centrifugation at 4000 rpm for 15 min, the spores were resuspended with sterile water and adjusted to the final concentration. The inoculum used for *Fov7* inoculation was 1 × 10^7^ spores mL^−1^ and 1 × 10^6^ spores mL^−1^ for *Vd991*. 5‐week‐old cotton seedlings were inoculated with *Fov race 7* suspension using the root‐irrigation method. 3‐week‐old Arabidopsis seedlings were washed with water to remove soil and dried on paper towels before being immersed in the *Fov7* or *Vd991* spore suspension for 3 min and then planted in pots with pre‐wet soil. After inoculation with *Fov7* and *Vd991*, the plants were returned to the same growth conditions as before the infection. For the fungi recovery assay, 1–2 cm stem sections from the base were surface sterilized in 70% ethanol and rinsed with sterile water after the incubation of *Fov7* and *Vd991* for 7 days. The stem segments were placed on the potato dextrose agar (PDA, AoBoXing Bio‐Tech Co., Ltd, Beijing, China) medium supplemented with kanamycin (50 mg L^−1^) and cultured for 3 days at 28 °C for *Fov 7* and at 25 °C for *Vd 991* and then photographed. The disease percentage and disease index was calculated as previously described by Wang et al.^[^ ^9^ ^]^ For *Arabidopsis*, the disease percentage and disease index were evaluated at 10 dpi, while for cotton, they were assessed at 25 dpi.

### qRT‐PCR

The total RNA (≈2 µg) of cotton and Arabidopsis leaves was extracted and was then reverse‐transcribed in a 20 µl reaction mixture with PrimeScript RT reagent Kit with gDNA Eraser (Perfect Real Time) (Cat # RR047A, Takara). Sample aliquots (1 µl) were used as templates for qRT‐PCR on a 7500 Real‐Time PCR system. Three technical replicates per sample and three biological replicates were analyzed for each experiment. *UBQ7* was used as the internal control for the qRT‐PCR data analysis. The primers used are listed in Table S6 (Supporting Information).

### VIGS Assays

Sea Island cotton (*G. barbadense*) highly susceptible (S) accession II15‐3464 and highly resistant (*R*) accession T10‐280 were used for VIGS transformation. For virus‐induced gene silencing (VIGS), 508 bp fragment from *GbPP2C80* was cloned into the *Pac*I and *Spe*I sites of the pCLCrV‐VA vector (the primers used are listed in Table S6, Supporting Information). This vector was introduced into *A. tumefaciens* strain GV3101. *Agrobacterium* cultures were adjusted to OD_600 _= 0.8 and *A. tumefaciens* samples with pCLCrV‐VA vector were mixed with pCLCrV‐VB vector in equal volumes, which were agro‐infiltrated into cotton cotyledons by vacuum infiltration as previously described.^[^ ^72^ ^]^ The primers used are listed in Table S6 (Supporting Information).

### Overexpression Vector Construction and Transformation

The full‐length open reading frames of the *GbPP2C80* and *GbWAKL14* were amplified through PCR using cDNAs synthesized from RNA that was isolated from seedlings of susceptible variety II15‐3464. The amplified products were further cloned into the p2301 vector driven by the cauliflower mosaic virus (CaMV) 35S promoter. The full‐length *GbPP2C80* sequence was inserted into the restriction endonuclease restriction sites *Xba*I and *Kpn*I of the p2301vector. The full‐length *GbWAKL14* was inserted into the restriction endonuclease restriction sites *Kpn* I and *Spe* I of the p2301vector. The resulting constructs were further transformed into *A. thalian* *a* by *Agrobacterium tumefaciens* GV3101 and selected with kanamycin.^[^ ^73^ ^]^ The primers used are listed in Table S6 (Supporting Information).

### CRISPR/Cas9 Gene‐Editing Vector Construction, Transformation and Mutation Identification

Two sgRNA targets were designed using the tool CRISPR‐P^[^ ^74^ ^]^ in the exon of *GbWAKL14*, namely sgRNA1 and sgRNA2 (Figure 5a; Figure S5a, Supporting Information). Two sgRNAs were 20 bp in length and 130 bp apart, followed by a 5′‐NGG PAM sequence in the forward strand (Figure 5c; Figure S5a, Supporting Information). Two pairs of DNA oligonucleotide primers for sgRNA1 and sgRNA2 were synthesized and annealed to generate dimers using overlapping extension PCR, followed by the acquisition of the plasmid vector containing both the targeted sgRNA cassettes and Cas9. The recombinant plasmids were inserted into the multiple cloning sites of the CRISPR/Cas9 vector pRGEB32‐GhU6.9‐NPT2.^[^ ^75^ ^]^ The primers used for the construction of the CRISPR/Cas9 recombinant vectors are listed in Table S8 (Supporting Information). The CRISPR/Cas9 recombinant plasmids were individually transformed into *Agrobacterium tumefaciens* GV3101 by the freeze‐thaw method. The hypocotyl of the upland cotton cultivar Jin668 was used as an explant for tissue culture and transformation according to the reports.^[^ ^64^, ^76^ ^]^ To detect mutations in the target regions, the fragments containing sgRNA sequences were amplified from the genome DNA by PCR using specific primers. PCR products were added with adaptors used for High‐throughput Tracking Of Mutations (Hi‐TOM) sequencing.^[^ ^33^ ^]^ The primers used for mutation detection are listed in Table S6 (Supporting Information).

### Subcellular Localization

The full‐length CDS sequences of *GbPP2C80* and *GbWAKL14* were inserted into the p35S::GFP vector to construct the recombinant vectors p35S::GbPP2C80‐GFP and p35S::GbWAKL14‐GFP. The primers used are listed in Table S6 (Supporting Information). The 35S‐GFP empty vector was used as a control. The pGD‐mCherry vector containing a red fluorescent protein (RFP) was used as a membrane marker. The four vectors were separately introduced into *Agrobacterium tumefaciens* strain GV3101, and then three kinds of *Agrobacterium* solution (OD_600_ = 0.5) containing GFP vectors were mixed up with the *Agrobacterium* solution (OD_600_ = 0.5) containing RFP vector in a ratio of 1:1, which were separately infiltrated into the leaves of *Nicotiana benthamiana*. After 24 h of dark treatment, the tobacco individuals injected with *Agrobacterium* solution were exposed to light for 48 h. The injected leaves were cut to observe the fluorescence of GFP signals using a Laser Scanning Confocal Microscope (Zeiss LSM 900, USA).

### BiFC Assays

BiFC experiments were used to verify the protein interactions. The full‐length CDS without termination codons of *GbPP2C80*, *GbWAKL14*, and *GbMPK3* were inserted into the pCambia 1300‐YFPN vector, respectively. Meanwhile, the full‐length CDS without termination codons of *GbPP2C80*, *GbMPK3*, and *GbRbohD* were inserted into the pCambia 1300‐YFPC vector, respectively. The primers used are listed in Table S6 (Supporting Information). The empty pCambia 1300‐YFPN vector and the empty pCambia 1300‐YFPC vector were used as negative controls for the BiFC assays. The vectors were individually transformed into *Agrobacterium tumefaciens*GV3101. *Agrobacterium* solutions containing GbWAKL14‐YFPN and GbPP2C80‐YFPC, GbPP2C80‐YFPN and GbMPK3‐YFPC, GbWAKL14‐YFPN and GbMPK3‐YFPC, GbMPK3‐YFPN and GbRbohD‐YFPC, YFPN and YFPC were mixed in 1:1 ratio, respectively. Four sets of mixed solutions were injected into *N. benthamiana* individuals using 1 mL syringes without needles. After dark for 24 h and light for 48 h, the YFP fluorescence signals in leaf epidermal cells were detected using a Laser Scanning Confocal Microscope (Zeiss LSM 880, USA).

### Y2H Assay

To confirm the interaction between GbPP2C80 and GbWAKL14, the protein codon sequence of *GbPP2C80* was inserted into pGBKT7 and introduced into the yeast strain AH109 to generate BD‐GbPP2C80 bait. Meanwhile, the full‐length CDS sequence of *GbWAKL14* was inserted into pGADT7 to generate GbWAKL14‐AD and introduced into yeast strain AH109. The positive clones of the bait and prey were mixed and mated in a 30 °C shaker for 24 h, followed by cultured on SD‐Leu‐Trp, SD‐Leu‐Trp‐His (with X‐*α*‐Gal, Coolaber, Beijing, China), and SD‐Leu‐Trp‐His‐Ade (with X‐*α*‐Gal) medium. pGADT7‐T/pGBKT7‐53 (AD‐T/BD‐53) plasmids were used as a positive control, and pGADT7/pGBKT7 (AD/BD) plasmids were used as a negative control. The primers used are listed in Table S6 (Supporting Information).

### Enzyme Activity Determination

To explore whether the wilt disease resistance conferred by *GbPP2C80* and *GbWAKL14* was related to ROS metabolism, the activities of two vital ROS scavenging enzymes, SOD and POD, were determined. Fresh samples (0.5 g; leaves for cotton, and seedlings for Arabidopsis) were added into 2 mL 0.05 mol L^−1^ pH7.8 phosphate buffer and were ground on the ice. The homogenate was poured into a 10 mL centrifuge tube. The mortar was rinsed with 1 mL 0.05 mol L^−1^ pH7.8 phosphate buffer three times (a total of 5 mL buffer). The homogenate was centrifuged at 10 000 revolutions per minute (rmp) for 20 min (4 °C). The supernatant was taken as a crude enzyme extract solution, which was stored at 0–4 °C and used to determine the activity of SOD and POD. To examine the activity of SOD, 3.3 mL reaction mixture was prepared as follows: 0.05 mol L^−1^ phosphoric acid buffer, 1.5 mL; 130 mmol L^−1^ methionine (Met), 0.3 mL; 750 µmol L^−1^ nitroblue tetrazolium (NBT), 0.3 mL; 100 µmol L^−1^ EDTA‐Na_2_, 0.3 mL; 20 µmol L^−1^ riboflavin (FD), 0.3 mL; enzyme extraction solution, 0.1 mL; H_2_O, 0.5 mL. Another 4 test tubes were used as the control and the buffer solution was used instead of the enzyme extraction solution. After mixing, 2 tubes were placed in the dark and the remaining tubes were subjected to a reaction under 4000 Lx sunlight for 20 min. After the reaction, the OD_560_ values of the tubes were determined separately by using the tubes without light as the blank. The total activity of SOD was calculated as the formula: SOD activity (U g^−1^ FW) = (*ACK *− *AE*) × *V*/(*ACK* × 0.5 × *W *× *Vt*). *ACK* was the absorbance of the illumination control tube. *AE* is the absorbance of the sample tube. *V* is the total volume of sample liquid (mL). *Vt* is the sample amount (mL) determined; *W* is the sample fresh weight (g). To examine the activity of POD, 0.005 mL enzyme extract solution was added into 3 mL reaction solution (0.1 mol L^−1^ pH6.0 phosphoric acid buffer, 100 mL; guaiacol, 0.5 mL; 30% H_2_O_2_, 1 mL) and shaken quickly in a test tube, and then poured into a cuvette. The reaction solution without enzyme extraction solution was used as the blank. The OD_470_ values were read every 15 s for a total of 1 minute (If there is a time scan, the start and end time interval of a line with a larger slope and its corresponding two absorption values can be directly recorded. The activity of POD was calculated as the formula: POD activity (∆*OD*_240_ g^−1^ FW min^−1^) = (∆*OD*_470 _×* V*/*Vt*)/(*W* × *T*). ∆*OD*_470_ was difference of two absorbance values. *V* is the total volume of enzyme extract solution (mL). *Vt* is the sample amount (mL) of enzyme extract solution determined. *W* is the sample fresh weight (g). *T* is the reaction time interval (min).

### DAB Staining

To further verify that *GbPP2C80* and *GbWAKL14* regulate wilt disease resistance via ROS content, the method of DAB staining was used. First, DAB dye solution was prepared as follows: 100 mg 3, 3′‐Diaminobenzidine tetrahydrochloride (DAB·4HCl, Sigma, USA, D5637) was dissolved in pH7.0 phosphoric acid buffer, and then 100 µL 0.01% Triton X‐100 was added, the total volume should be 100 mL by filling pH7.0 phosphoric acid buffer. Second, the sampled leaves were fully immersed in a DAB dye solution and vacuumed for 30 min. Third, they were incubated overnight at room temperature. Finally, the photos of the leaves were taken after decolorization with 95% ethanol in an 80 °C water bath.

### Molecular Markers Development

To assist molecular breeding, molecular markers based on *GbPP2C80* and *GbWAKL14* were developed to effectively distinguish susceptible and resistant Sea Island cotton varieties at any stage of development. First, all variations, including SNPs and InDels in the 2 kb promoter regions and gene regions of *GbPP2C80* and *GbWAKL14*, were retrieved from the files SNP.vcf and InDel.vcf of the 336 Sea Island cotton population, using software “vcftools”. Second, promoter regions containing larger InDels were exploited for a primer design using Primer Premier 5. Third, 10 extremely susceptible Sea Island cotton varieties (S1–S10) and 10 extremely resistant Sea Island cotton varieties (R1–R10) were planted in the pots with nutritive soil and vermiculite (V: V = 2: 1), and grown in a greenhouse under 16 h light/8 h dark, 28 °C. The information about 10 extremely susceptible and 10 extremely resistant Sea Island cotton varieties were detailed in Table S7 (Supporting Information). After the cotyledons were flattened, the seedlings were sampled for DNA extraction using the modified CTAB method. Fourth, PCR with different annealing temperatures and agarose gel concentrations were examined to find the optimum conditions. Finally, the prime primers and PCR conditions were determined as follows: for *GbPP2C80*, the susceptible‐specific (S marker, Tm 60.0 °C, product length 924 bp) and resistant‐specific (R marker, Tm 62.0 °C, product length 913 bp) primers based on 11 bp InDel in promoter region, respectively were chosen; for *GbWAKL14*, only one pair of primers (S/R marker) was confirmed to distinguish the susceptible (product length 416 bp) and resistant varieties (product length 452 bp) based on 36 bp InDels in promoter region (both Tm, 57.0 °C). The sequences of these two sets of primers are listed in Table S6 (Supporting Information). Although 5% agarose gel was originally used to distinguish between bands of different sizes, 1% agarose gel was later found to be sufficient to distinguish between bands amplified from susceptible and resistant varieties. For *GbPP2C80*, it was the difference of both the band size (11 bp) and the presence/absence variation. For *GbWAKL14*, a size discrepancy of 36 bp could be obviously identified on a 1% agarose gel, leading to significant savings in experimental costs.

## Conflict of Interest

The authors declare no conflict of interest.

## Supporting information

Supporting Information

## Data Availability

The data that support the findings of this study are openly available in the NCBI sequence read archive (SRA) under BioProject ID: PRJNA720818 at 10.1111/pbi.13747, reference number 32.
