# Supplementary material for: GbPP2C80 Interacts with GbWAKL14 to Negatively Co‐Regulate Resistance to Fusarium and Verticillium wilt via MPK3 and ROS Signaling in Sea Island Cotton
Source: Adv Sci (Weinh). 2024 Jun 18;11(30):2309785. doi: 10.1002/advs.202309785 (PMC11321686; doi:10.1002/advs.202309785)
Supplement: Supplementary file 1 — Supporting Information [file ADVS-11-2309785-s001.docx]

Supporting Information

GbPP2C80 Interacts with GbWAKL14 to Negatively Co-regulate Resistance to *Fusarium* and *Verticillium wilt* via MPK3 and ROS Signaling in Sea Island Cotton

Nan Zhao, Anhui Guo, Weiran Wang, Bin Li, Meng Wang, Zixin Zhou, Kaiyun Jiang, Alifu·Aierxi, Baoliang Wang, Daniel Adjibolosoo, Zhanghao Xia, Huijing Li, Yanan Cui, Jie Kong* and Jinping Hua*

**Table S1.** Information of *FW* disease percentage of 336 Sea Island cotton accessions.

| No. | Accession | Origin ^a)^ | DP [%]^b)^ | No. | Accession | Origin^a)^ | DP [%]^b)^ |
| --- | --- | --- | --- | --- | --- | --- | --- |
| 1 | II15-3491 | Tajikistan, CA | 0.00 | 36 | K-335 | Xinjiang, CHN | 3.46±1.61 |
| 2 | T10-280 | Xinjiang, CHN | 0.00±0.00 | 37 | SuB8915 | Tajikistan, CA | 3.47 |
| 3 | XH42 | Xinjiang, CHN | 0.18±0.36 | 38 | L3404 | Xinjiang, CHN | 3.53 |
| 4 | XH49 | Xinjiang, CHN | 0.18±0.37 | 39 | TJK1 | Tajikistan, CA | 3.65±1.22 |
| 5 | XH51 | Xinjiang, CHN | 0.19±0.38 | 40 | XH44 | Xinjiang, CHN | 3.95±3.66 |
| 6 | XH37 | Xinjiang, CHN | 0.21±0.42 | 41 | 9122И | Turkmenistan, CA | 4.17±0.92 |
| 7 | XH43 | Xinjiang, CHN | 0.28±0.57 | 42 | XH60 | Xinjiang, CHN | 4.71±1.61 |
| 8 | XH38 | Xinjiang, CHN | 0.36±0.71 | 43 | 5230B | Tajikistan, CA | 5.15±0.75 |
| 9 | XH56 | Xinjiang, CHN | 0.39±0.78 | 44 | K-128 | Xinjiang, CHN | 5.19±0.56 |
| 10 | XH53 | Xinjiang, CHN | 0.51±1.03 | 45 | PIMA | USA | 5.38±0.90 |
| 11 | XH46 | Xinjiang, CHN | 0.54±1.09 | 46 | C352 | Shanghai, CHN | 5.75±4.64 |
| 12 | XH48 | Xinjiang, CHN | 0.59±1.17 | 47 | II15-3428 | Tajikistan, CA | 5.90 |
| 13 | XH57 | Xinjiang, CHN | 0.77±1.54 | 48 | SuB155 | Tajikistan, CA | 6.04 |
| 14 | XH54 | Xinjiang, CHN | 0.81±1.61 | 49 | Su90200 | Uzbekistan, CA | 6.33±4.39 |
| 15 | XH47 | Xinjiang, CHN | 0.85±1.71 | 50 | BZ47-65 | Xinjiang, CHN | 6.37±3.86 |
| 16 | XH52 | Xinjiang, CHN | 0.91±1.20 | 51 | 5476И | Turkmenistan, CA | 6.57±0.23 |
| 17 | XH55 | Xinjiang, CHN | 0.92±1.84 | 52 | SuB8916 | Tajikistan, CA | 7.09 |
| 18 | Naire | Xinjiang, CHN | 1.15±1.63 | 53 | Xiao8528 | Xinjiang, CHN | 7.09 |
| 19 | XH58 | Xinjiang, CHN | 1.50±3.00 | 54 | Giza77 | Egypt | 7.24±7.62 |
| 20 | Hai-cw | Henan, CHN | 1.67±0.59 | 55 | Su4294 | Uzbekistan, CA | 7.40±3.98 |
| 21 | DIMD67 | Jiangsu, CHN | 2.22 | 56 | XK90006 | Xinjiang, CHN | 7.68±4.66 |
| 22 | 572Q | USA | 2.30±1.53 | 57 | 700Q | USA | 7.97±1.40 |
| 23 | K-132 | Xinjiang, CHN | 2.38±1.45 | 58 | XK399-8 | Xinjiang, CHN | 8.04±7.30 |
| 24 | K-136 | Xinjiang, CHN | 2.38±1.76 | 59 | Su90198 | Uzbekistan, CA | 8.09±6.02 |
| 25 | II15-3506 | Tajikistan, CA | 2.44 | 60 | II15-3506-1 | Tajikistan, CA | 8.12 |
| 26 | 08AW009 | Xinjiang, CHN | 2.68±1.47 | 61 | Su9943 | Turkmenistan, CA | 8.14±6.19 |
| 27 | BZ3410 | Xinjiang, CHN | 2.71±0.82 | 62 | XH11 | Xinjiang, CHN | 8.37±7.24 |
| 28 | XH39 | Xinjiang, CHN | 2.76±2.49 | 63 | 9140И | Turkmenistan, CA | 8.46±1.17 |
| 29 | K-103 | Xinjiang, CHN | 2.95±3.00 | 64 | Giza68 | Egypt | 8.59±3.18 |
| 30 | XH41 | Xinjiang, CHN | 3.00±4.43 | 65 | BZ4159 | Xinjiang, CHN | 8.69±0.57 |
| 31 | XH36 | Xinjiang, CHN | 3.08±3.99 | 66 | BZ873112 | Xinjiang, CHN | 8.74±5.99 |
| 32 | II15-3469 | Tajikistan, CA | 3.25 | 67 | Su11 | Uzbekistan, CA | 9.57±9.03 |
| 33 | XH28 | Xinjiang, CHN | 3.27±2.52 | 68 | BZ3119 | Xinjiang, CHN | 9.81±0.70 |
| 34 | XkK106 | Xinjiang, CHN | 3.27±3.59 | 69 | Su4314 | Uzbekistan, CA | 10.28±6.56 |
| 35 | L3402 | Xinjiang, CHN | 3.38 | 70 | Su1241 | Uzbekistan, CA | 10.44±4.98 |

^a)^ CA, CHN, and USA represent Central Asia, China, United States of America; ^b)^ DP represents disease percentage (%), the value is mean ± standard deviation.

**Table S1.** Information of *FW* disease percentage of 336 Sea Island cotton accessions. (Continued)

| No. | Accession | Origin ^a)^ | DP [%]^b)^ | No. | Accession | Origin^a)^ | DP [%]^b)^ |
| --- | --- | --- | --- | --- | --- | --- | --- |
| 71 | SOVIN | Xinjiang, CHN | 10.44 | 140 | Giza88 | Egypt | 25.69±1.58 |
| 72 | Su4394 | Uzbekistan, CA | 11.19±6.29 | 141 | BZ266 | Xinjiang, CHN | 25.93±4.11 |
| 73 | Ba2365 | Xinjiang, CHN | 11.25 | 142 | DP353-1 | USA | 25.99±4.89 |
| 74 | SuB56 | Tajikistan, CA | 11.51±7.58 | 143 | Yue51 | Yunnan, CHN | 26.21±2.46 |
| 75 | S03 | Xinjiang, CHN | 11.55±1.23 | 144 | SuB154 | Tajikistan, CA | 26.34±2.86 |
| 76 | Su5931 | Uzbekistan, CA | 11.81±3.43 | 145 | 8704И | Turkmenistan, CA | 26.42±0.97 |
| 77 | XH34 | Xinjiang, CHN | 11.99±7.80 | 146 | LuoSaiNa | Egypt | 26.44±4.88 |
| 78 | XK163-4-1 | Xinjiang, CHN | 12.07±8.54 | 147 | BZ47-65 | Xinjiang, CHN | 26.63±1.60 |
| 79 | YH4 | Viet Nam | 12.29±8.83 | 148 | XH3 | Xinjiang, CHN | 26.78±9.05 |
| 80 | Su90199 | Uzbekistan, CA | 12.79±2.33 | 149 | SuB153 | Tajikistan, CA | 26.80±4.24 |
| 81 | 5904-И | Turkmenistan, CA | 12.92 | 150 | CA275 | Canada | 27.23±15.94 |
| 82 | C-6213 | Uzbekistan, CA | 12.92 | 151 | K-228 | Xinjiang, CHN | 27.56±4.03 |
| 83 | XH33 | Xinjiang, CHN | 13.18±7.86 | 152 | Su2525 | Uzbekistan, CA | 27.73±6.89 |
| 84 | Su3965 | Uzbekistan, CA | 13.49±13.98 | 153 | Su4401 | Uzbekistan, CA | 27.89±12.22 |
| 85 | TJK2 | Tajikistan, CA | 13.62±7.30 | 154 | KakY9-2 | Xinjiang, CHN | 27.90±2.19 |
| 86 | 9763И | Turkmenistan, CA | 13.77±0.48 | 155 | Mo1431 | Xinjiang, CHN | 28.68±8.27 |
| 87 | Giza75 | Egypt | 13.86 | 156 | Giza2 | Egypt | 28.93±6.86 |
| 88 | Giza67 | Egypt | 14.32 | 157 | BZ4932 | Xinjiang, CHN | 28.94±5.17 |
| 89 | SuB9101 | Tajikistan, CA | 14.47±1.41 | 158 | Giza1 | Egypt | 29.45±4.77 |
| 90 | 8763И | Turkmenistan, CA | 14.53±1.03 | 159 | XH29 | Xinjiang, CHN | 30.03±7.85 |
| 91 | Pimacotton | USA | 14.78±8.71 | 160 | DP340 | USA | 30.24±0.77 |
| 92 | FB-20 | USA | 15.42 | 161 | PiB9101 | USA | 30.41±19.94 |
| 93 | Giza30 | Egypt | 15.50±1.04 | 162 | TH2 | Xinjiang, CHN | 30.75±6.95 |
| 94 | XK90103 | Xinjiang, CHN | 15.94±7.21 | 163 | Pima79 | USA | 31.09±4.37 |
| 95 | Su7871 | Turkmenistan, CA | 15.95±3.74 | 164 | Giza80 | Egypt | 31.62±4.73 |
| 96 | Su90197 | Uzbekistan, CA | 16.59±6.80 | 165 | PimaS-3 | USA | 32.12±8.65 |
| 97 | Su1243 | Uzbekistan, CA | 16.61±8.31 | 166 | BZ1248 | Xinjiang, CHN | 32.20±3.44 |
| 98 | Giza69 | Egypt | 16.76 | 167 | PimaS2 | USA | 32.35±4.11 |
| 99 | Su4399 | Uzbekistan, CA | 16.86±1.92 | 168 | 910И | Turkmenistan, CA | 32.77±0.27 |
| 100 | SuB9103 | Tajikistan, CA | 16.95±4.00 | 169 | XH35 | Xinjiang, CHN | 33.02±7.67 |
| 101 | BZ1120 | Xinjiang, CHN | 17.05±5.67 | 170 | TH1 | Xinjiang, CHN | 33.56±3.37 |
| 102 | 9983И | Turkmenistan, CA | 17.22±13.93 | 171 | XH24 | Xinjiang, CHN | 33.59±3.17 |
| 103 | PYE cotton | USA | 17.33±5.14 | 172 | Giza70-2 | Egypt | 34.46±7.24 |
| 104 | XH27 | Xinjiang, CHN | 17.41±9.18 | 173 | XH20 | Xinjiang, CHN | 34.60±6.23 |
| 105 | C6037 | Uzbekistan, CA | 18.51±1.43 | 174 | И24-3386 | Turkmenistan, CA | 34.94±1.05 |
| 106 | Su2515 | Uzbekistan, CA | 18.66±7.62 | 175 | 9363 | Egypt | 35.01 |
| 107 | BZ4021 | Xinjiang, CHN | 19.02±13.27 | 176 | CRM 605 | Jiangsu, CHN | 35.22 |
| 108 | XK90098 | Xinjiang, CHN | 19.03±8.22 | 177 | KakV79 | Xinjiang, CHN | 35.38±3.43 |
| 109 | Su4398 | Uzbekistan, CA | 19.50±5.35 | 178 | Pima5-1 | USA | 35.87±19.42 |
| 110 | BZ4150 | Xinjiang, CHN | 19.77±16.49 | 179 | XH15 | Xinjiang, CHN | 35.94±2.85 |
| 111 | Su4396 | Uzbekistan, CA | 20.02±2.62 | 180 | AShi | Turkmenistan, CA | 36.09±9.75 |
| 112 | SuB51 | Tajikistan, CA | 20.04 | 181 | HM-1 | Xinjiang, CHN | 36.30±5.84 |
| 113 | Su2283 | Uzbekistan, CA | 20.05±10.23 | 182 | Barl | Sudan | 36.41±10.97 |
| 114 | JiB91-47 | Hebei, CHN | 20.21±8.77 | 183 | SL1 | Xinjiang, CHN | 36.50±0.06 |
| 115 | Yue51-2 | Yunnan, CHN | 20.28±1.57 | 184 | AK4154 | Xinjiang, CHN | 36.67±1.18 |
| 116 | XK198-1 | Xinjiang, CHN | 20.68±11.72 | 185 | XH17 | Xinjiang, CHN | 37.11±8.96 |
| 117 | KKH-8660 | Xinjiang, CHN | 20.82±7.83 | 186 | SuB54 | Tajikistan, CA | 37.29±12.75 |
| 118 | 504-И | Tajikistan, CA | 20.87±18.03 | 187 | YH5 | Viet Nam | 37.32±5.12 |
| 119 | SuB67 | Tajikistan, CA | 20.91±10.76 | 188 | Su5803 | Uzbekistan, CA | 37.95±3.83 |
| 120 | C6249 | Uzbekistan, CA | 20.92±10.78 | 189 | BZ5-23 | Xinjiang, CHN | 39.67±5.19 |
| 121 | XH8 | Xinjiang, CHN | 21.13±5.10 | 190 | Giza81 | Egypt | 39.90±5.98 |
| 122 | 9123И | Turkmenistan, CA | 21.17±7.08 | 191 | AK86430 | Xinjiang, CHN | 40.01±4.80 |
| 123 | ShiH219 | Xinjiang, CHN | 21.50±6.29 | 192 | JiB91-41 | Hebei, CHN | 40.55±7.19 |
| 124 | Su1248 | Uzbekistan, CA | 21.53±4.95 | 193 | Pima5-2 | USA | 40.63±9.68 |
| 125 | SuB149 | Tajikistan, CA | 21.57±11.03 | 194 | A6009 | Xinjiang, CHN | 40.75 |
| 126 | Giza45 | Egypt | 21.63±4.80 | 195 | L-3398 | Xinjiang, CHN | 40.94 |
| 127 | Pima6 | USA | 21.65±8.05 | 196 | JH1 | Xinjiang, CHN | 41.33±6.99 |
| 128 | XH30 | Xinjiang, CHN | 22.81±2.68 | 197 | Damdara | Egypt | 41.57±8.47 |
| 129 | SuB24 | Tajikistan, CA | 23.18±20.79 | 198 | Giza76 | Egypt | 41.94±5.29 |
| 130 | C605 | Shanghai, CHN | 23.39±6.63 | 199 | SuB9102 | Tajikistan, CA | 42.33 |
| 131 | SuK-12 | Uzbekistan, CA | 23.88±22.51 | 200 | XK90062 | Xinjiang, CHN | 42.50±15.50 |
| 132 | Mi10 | Shanghai, CHN | 23.88±1.58 | 201 | Bai185 | Egypt | 42.75±4.30 |
| 133 | BZ3410 | Xinjiang, CHN | 24.01±4.32 | 202 | 2И3 | Turkmenistan, CA | 43.15±1.26 |
| 134 | KK86-42 | Xinjiang, CHN | 24.45±9.21 | 203 | XH7 | Xinjiang, CHN | 43.31 |
| 135 | XH31 | Xinjiang, CHN | 24.62±8.06 | 204 | XkK107 | Xinjiang, CHN | 43.47±4.33 |
| 136 | XkK403-8 | Xinjiang, CHN | 25.13±5.97 | 205 | E24-3353 | Jiangsu, CHN | 43.83 |
| 137 | 11/58-1/61 | Jiangsu, CHN | 25.54 | 206 | E24-3360 | Jiangsu, CHN | 44.24 |
| 138 | DaXuan71 | Yunnan, CHN | 25.57±0.80 | 207 | 5476-И | Turkmenistan, CA | 44.40 |
| 139 | ZhongYa | Uzbekistan, CA | 25.65±17.81 | 208 | KangWei2 | Henan, CHN | 44.62±19.86 |

^a)^ CA, CHN, and USA represent Central Asia, China, United States of America; ^b)^ DP represents disease percentage (%), the value is mean ± standard deviation.

**Table S1.** Information of *FW* disease percentage of 336 Sea Island cotton accessions. (Continued)

| No. | Accession | Origin ^a)^ | DP [%]^b)^ | No. | Accession | Origin ^a)^ | DP [%]^b)^ |
| --- | --- | --- | --- | --- | --- | --- | --- |
| 209 | Ashmoni | Egypt | 44.87±10.19 | 254 | 7045 | Xinjiang, CHN | 56.65 |
| 210 | AK3836 | Xinjiang, CHN | 45.16±3.21 | 255 | XH10 | Xinjiang, CHN | 57.00±8.56 |
| 211 | ZaoC1 | Yunnan, CHN | 45.18±1.21 | 256 | Carnac55 | Egypt | 57.76 |
| 212 | West Indies | West Indies | 45.33±9.35 | 257 | Giza45-4 | Egypt | 58.23 |
| 213 | AK4215 | Xinjiang, CHN | 45.93±3.03 | 258 | YH3 | Viet Nam | 58.32±5.23 |
| 214 | SuB89101 | Tajikistan, CA | 46.13±22.36 | 259 | Hai1 | Henan, CHN | 58.42±26.22 |
| 215 | Peru cotton | Peru | 47.12±2.73 | 260 | Pima calbolf | USA | 58.88±10.57 |
| 216 | YunNan3 | Yunnan, CHN | 47.62±8.99 | 261 | Menoufi | Egypt | 58.91 |
| 217 | G1453 | Egypt | 47.67±13.08 | 262 | Giza79 | Egypt | 59.14 |
| 218 | XH13 | Xinjiang, CHN | 47.69±1.67 | 263 | Pima3 | USA | 59.43±8.66 |
| 219 | SYR cotton | Syria | 47.80±14.92 | 264 | ZH261 | Henan, CHN | 59.67 |
| 220 | LuoSaiYa | Albania | 48.16±16.24 | 265 | Hai7124 | Jiangsu, CHN | 59.69±10.26 |
| 221 | YeJiHong | Egypt | 48.24±11.89 | 266 | Northwest | Uzbekistan, CA | 60.43 |
| 222 | XH25 | Xinjiang, CHN | 48.32±7.20 | 267 | Giza85 | Egypt | 60.54±6.58 |
| 223 | HunXuan8 | Xinjiang, CHN | 48.38±0.77 | 268 | Hai92-138 | Henan, CHN | 60.66 |
| 224 | Lushnje | Albania | 48.42±4.03 | 269 | JiB91-49 | Hebei, CHN | 60.73±5.97 |
| 225 | Pima67 | USA | 48.47±9.52 | 270 | HX86225 | Yunnan, CHN | 60.93±17.74 |
| 226 | Yingzi6022 | Jiangsu, CHN | 48.81 | 271 | Jiangsu CRM^c^ | Jiangsu, CHN | 61.36 |
| 227 | SUD2 | Sudan | 49.44±4.16 | 272 | Coibalt | USA | 61.98±7.15 |
| 228 | Tu86-6-17 | Turkmenistan, CA | 50.10 | 273 | XH16 | Xinjiang, CHN | 62.36±1.33 |
| 229 | XH26 | Xinjiang, CHN | 50.46±2.08 | 274 | XH23 | Xinjiang, CHN | 62.46±12.29 |
| 230 | L3598 | Xinjiang, CHN | 50.64 | 275 | XK90062 | Xinjiang, CHN | 64.16 |
| 231 | Giza20 | Egypt | 50.74±12.68 | 276 | XkK4102 | Xinjiang, CHN | 64.81±19.86 |
| 232 | Ba3244 | Xinjiang, CHN | 50.83 | 277 | V9-2 | Turkmenistan, CA | 65.06 |
| 233 | YueJin1 | Yunnan, CHN | 51.17±5.90 | 278 | Ba3021 | Xinjiang, CHN | 65.15 |
| 234 | HaiFu6 | Henan, CHN | 51.32±28.29 | 279 | PingYuan1 | Guangdong, CHN | 66.66±20.34 |
| 235 | XH21 | Xinjiang, CHN | 51.38±5.29 | 280 | JiB91-46 | Hebei, CHN | 66.67 |
| 236 | 8981-И | Turkmenistan, CA | 51.54 | 281 | XH18 | Xinjiang, CHN | 66.79±6.48 |
| 237 | Yunnan8040-2 | Yunnan, CHN | 52.38 | 282 | AK324 | Xinjiang, CHN | 66.80±8.01 |
| 238 | 1500084 | Jiangsu, CHN | 52.38 | 283 | Pima86-6 | USA | 67.16±10.33 |
| 239 | MoShi729 | Azerbaijan, CA | 52.59±17.14 | 284 | II15-3499 | Tajikistan, CA | 67.79 |
| 240 | XH6 | Xinjiang, CHN | 53.07±8.80 | 285 | XH12 | Xinjiang, CHN | 67.84±10.00 |
| 241 | A6009 | Xinjiang, CHN | 53.56±5.62 | 286 | AShi91 | Turkmenistan, CA | 68.72±3.74 |
| 242 | AK44251 | Xinjiang, CHN | 53.86±1.75 | 287 | SUD1 | Sudan | 69.50±11.64 |
| 243 | Antigua | Antigua | 54.24±2.64 | 288 | ZZD178 | Turkmenistan, CA | 69.89 |
| 244 | Pima90-53 | USA | 54.49±10.61 | 289 | 65-3049-6 | Jiangsu, CHN | 69.90±10.26 |
| 245 | SuB8912 | Tajikistan, CA | 54.50 | 290 | XH5 | Xinjiang, CHN | 70.57±1.10 |
| 246 | HaiFu9 | Henan, CHN | 54.55 | 291 | BZ4256 | Xinjiang, CHN | 70.61±19.52 |
| 247 | Moshi0 | Azerbaijan, CA | 54.65 | 292 | 65-3028-5 | Jiangsu, CHN | 70.63±10.91 |
| 248 | YH9 | Viet Nam | 54.69±8.47 | 293 | 7051 | Yunnan, CHN | 70.78 |
| 249 | PS-7 | Xinjiang, CHN | 55.27±5.80 | 294 | BZ843037 | Xinjiang, CHN | 71.24±16.43 |
| 250 | SuB69 | Tajikistan, CA | 55.40 | 295 | DP744 | USA | 71.24±4.06 |
| 251 | YH6 | Viet Nam | 55.71±8.81 | 296 | SuB66 | Tajikistan, CA | 71.79 |
| 252 | YuanMou2 | Yunnan, CHN | 56.26±3.48 | 297 | HX86158 | Yunnan, CHN | 71.94 |
| 253 | ShihzV7-4 | Xinjiang, CHN | 56.63±3.46 | 298 | YH7 | Viet Nam | 72.69 |

^a)^ CA, CHN, and USA represent Central Asia, China, United States of America; ^b)^ DP represents disease percentage (%), the value is mean ± standard deviation.

**Table S1.** Information of *FW* disease percentage of 336 Sea Island cotton accessions. (Continued)

| No. | Accession | Origin ^a)^ | DP [%]^b)^ | No. | Accession | Origin ^a)^ | DP [%]^b)^ |
| --- | --- | --- | --- | --- | --- | --- | --- |
| 299 | XH9 | Xinjiang, CHN | 72.87±4.76 | 318 | Super-okra CRM | Xinjiang, CHN | 83.28 |
| 300 | XH14 | Xinjiang, CHN | 73.19±11.38 | 319 | JiB91-44 | Hebei, CHN | 83.37±4.49 |
| 301 | Okra CRM | Xinjiang, CHN | 73.55 | 320 | Hai66-170 | Xinjiang, CHN | 83.47 |
| 302 | YH10 | Viet Nam | 73.89±7.31 | 321 | E24-3587 | Jiangsu, CHN | 84.66 |
| 303 | JiB91-46 | Hebei, CHN | 74.22±15.23 | 322 | ShihzV4-2 | Xinjiang, CHN | 85.18±10.09 |
| 304 | L3396 | Xinjiang, CHN | 75.00 | 323 | YueLing2 | Yunnan, CHN | 86.04±6.62 |
| 305 | Ji92-113 | Hebei, CHN | 75.31±12.22 | 324 | AK7317 | Xinjiang, CHN | 88.50 |
| 306 | PiB94-1 | USA | 75.66±5.10 | 325 | Ashi91 | Turkmenistan, CA | 88.57 |
| 307 | XH19 | Xinjiang, CHN | 75.99±7.81 | 326 | Nisaifu | Uzbekistan, CA | 88.90 |
| 308 | 7125 | Jiangsu, CHN | 76.33 | 327 | XK2442 | Xinjiang, CHN | 90.01 |
| 309 | JiB91-42 | Hebei, CHN | 76.74 | 328 | Su9943-И | Turkmenistan, CA | 90.69 |
| 310 | Hai92-137 | Henan, CHN | 77.62±3.88 | 329 | XK213 | Xinjiang, CHN | 91.04 |
| 311 | Giza57 | Egypt | 78.69 | 330 | BZ1248 | Xinjiang, CHN | 91.10 |
| 312 | II15-3444 | Tajikistan, CA | 78.88 | 331 | II15-3460 | Tajikistan, CA | 93.01 |
| 313 | Yue51-11 | Yunnan, CHN | 78.90±9.90 | 332 | YJ005499 | Jiangsu, CHN | 93.10 |
| 314 | XH22 | Xinjiang, CHN | 80.04±10.56 | 333 | 3761 | Argentina | 94.29 |
| 315 | 4092 | Egypt | 80.93 | 334 | II15-3493 | Tajikistan, CA | 95.70 |
| 316 | Syrian CRM | Syria | 81.20 | 335 | Su7871-И | Turkmenistan, CA | 98.75 |
| 317 | AK785-3 | Xinjiang, CHN | 82.82±10.44 | 336 | II15-3464 | Tajikistan, CA | 98.78 |

^a)^ CA, CHN, and USA represent Central Asia, China, United States of America; ^b)^ DP represents disease percentage (%), the value is mean ± standard deviation.


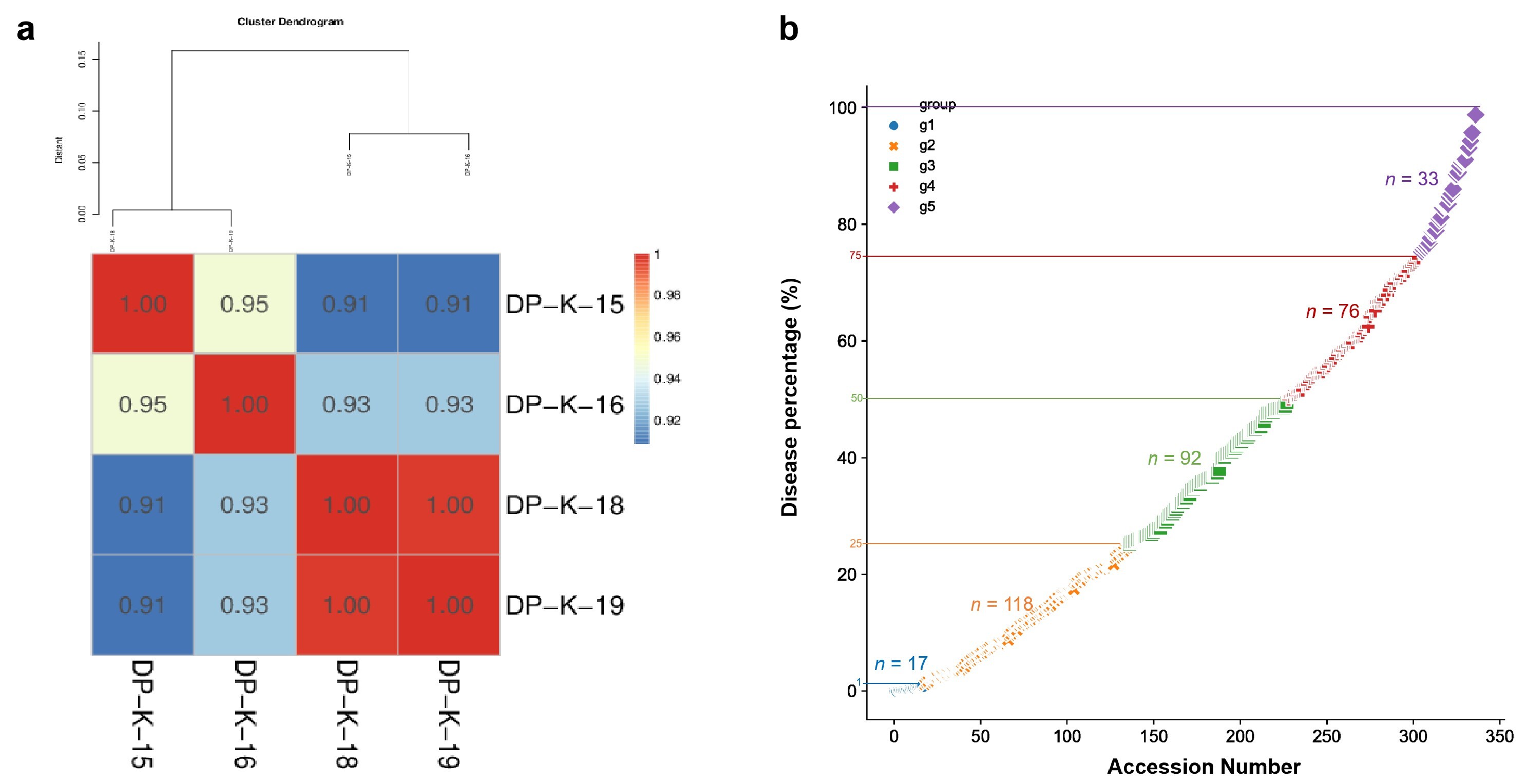


**Figure S1.** Correlation and grouping of *FW* disease percentage at different locations and stages in Sea Island cotton accessions. (a) Correlation of *FW* disease percentage of Sea Island cotton accessions among different years in the same location (Korla, Xinjiang). (b) Five groups divided by *FW* disease percentage of Sea Island cotton accessions. *n* denotes the number of Sea Island cotton accessions in each group.

**Table S2.** List of the associated SNPs identified in different datasets through GWAS for *FW* resistance in Sea Island cotton.

| SNP | Ref/Alt | MAF | -log (p-value) | SNP | Ref/Alt | MAF | -log (p-value) | SNP | Ref/Alt | MAF | -log (p-value) |
| --- | --- | --- | --- | --- | --- | --- | --- | --- | --- | --- | --- |
| **2015** |  |  |  | Gbar_D03_1899807 | G/A | 0.09 | 6.07 | Gbar_D03_239840 | C/T | 0.07 | 6.03 |
| Gbar_D03_1001198 | G/A | 0.08 | 6.79 | Gbar_D03_1910529 | A/G | 0.46 | 6.07 | Gbar_D03_242138 | G/A | 0.08 | 6.06 |
| Gbar_D03_1021128 | T/C | 0.08 | 6.52 | Gbar_D03_1918318 | C/A | 0.08 | 6.99 | Gbar_D03_242166 | T/A | 0.07 | 6.52 |
| Gbar_D03_1033138 | A/G | 0.09 | 6.46 | Gbar_D03_1918338 | A/G | 0.09 | 6.76 | Gbar_D03_243816 | G/T | 0.08 | 6.15 |
| Gbar_D03_1036297 | T/G | 0.14 | 6.48 | Gbar_D03_1918360 | G/A | 0.09 | 6.46 | Gbar_D03_244284 | C/T | 0.08 | 6.36 |
| Gbar_D03_1050696 | G/T | 0.38 | 7.66 | Gbar_D03_1918465 | C/A | 0.09 | 7.13 | Gbar_D03_245629 | A/G | 0.08 | 6.20 |
| Gbar_D03_1050724 | G/T | 0.39 | 7.56 | Gbar_D03_1922237 | A/T | 0.08 | 7.24 | Gbar_D03_245684 | C/A | 0.08 | 6.47 |
| Gbar_D03_1052369 | A/C | 0.37 | 6.10 | Gbar_D03_1922302 | T/C | 0.09 | 6.59 | Gbar_D03_245980 | A/T | 0.08 | 6.26 |
| Gbar_D03_1053536 | A/T | 0.38 | 6.66 | Gbar_D03_1922310 | A/G | 0.08 | 6.56 | Gbar_D03_246396 | G/T | 0.07 | 6.16 |
| Gbar_D03_1057322 | C/T | 0.39 | 7.71 | Gbar_D03_1922363 | G/A | 0.09 | 7.00 | Gbar_D03_246409 | C/A | 0.07 | 6.01 |
| Gbar_D03_1057381 | G/A | 0.39 | 7.88 | Gbar_D03_1922408 | T/C | 0.09 | 7.08 | Gbar_D03_246540 | T/C | 0.08 | 6.19 |
| Gbar_D03_1058771 | T/C | 0.41 | 6.13 | Gbar_D03_1923098 | A/G | 0.08 | 7.06 | Gbar_D03_247958 | A/G | 0.08 | 6.88 |
| Gbar_D03_1058777 | C/A | 0.41 | 6.19 | Gbar_D03_1926752 | T/C | 0.08 | 6.62 | Gbar_D03_248567 | A/G | 0.08 | 6.16 |
| Gbar_D03_1058910 | C/T | 0.40 | 6.73 | Gbar_D03_1926776 | G/A | 0.08 | 6.75 | Gbar_D03_250966 | C/T | 0.08 | 6.16 |
| Gbar_D03_1061196 | T/A | 0.15 | 6.37 | Gbar_D03_1926816 | A/G | 0.09 | 6.07 | Gbar_D03_252667 | G/T | 0.08 | 6.31 |
| Gbar_D03_1062430 | T/C | 0.10 | 6.36 | Gbar_D03_1926884 | G/A | 0.09 | 6.16 | Gbar_D03_254335 | T/A | 0.08 | 6.01 |
| Gbar_D03_1064857 | C/G | 0.09 | 6.53 | Gbar_D03_1928210 | C/G | 0.10 | 6.58 | Gbar_D03_255137 | C/T | 0.08 | 6.05 |
| Gbar_D03_1068053 | A/G | 0.15 | 6.22 | Gbar_D03_1928539 | T/C | 0.09 | 6.24 | Gbar_D03_255247 | C/T | 0.08 | 6.10 |
| Gbar_D03_1075812 | A/G | 0.15 | 6.01 | Gbar_D03_1928830 | G/T | 0.09 | 6.92 | Gbar_D03_256410 | T/C | 0.08 | 6.27 |
| Gbar_D03_1076579 | T/C | 0.15 | 6.72 | Gbar_D03_1941440 | A/T | 0.09 | 6.94 | Gbar_D03_257735 | G/C | 0.08 | 6.10 |
| Gbar_D03_1076588 | G/A | 0.15 | 6.70 | Gbar_D03_1956848 | T/C | 0.09 | 6.45 | Gbar_D03_258830 | C/A | 0.08 | 6.35 |
| Gbar_D03_1087424 | A/C | 0.15 | 6.29 | Gbar_D03_1957237 | T/G | 0.09 | 7.38 | Gbar_D03_263853 | C/T | 0.08 | 6.12 |
| Gbar_D03_1092095 | G/C | 0.16 | 6.30 | Gbar_D03_1960995 | C/T | 0.09 | 6.15 | Gbar_D03_264598 | A/T | 0.07 | 6.13 |
| Gbar_D03_1105006 | A/G | 0.08 | 6.19 | Gbar_D03_1980162 | A/T | 0.12 | 6.69 | Gbar_D03_264615 | A/G | 0.08 | 6.09 |
| Gbar_D03_1105012 | G/T | 0.16 | 6.55 | Gbar_D03_1989223 | C/T | 0.09 | 6.94 | Gbar_D03_265107 | T/A | 0.08 | 6.10 |
| Gbar_D03_1105045 | C/T | 0.08 | 6.02 | Gbar_D03_1989306 | C/T | 0.09 | 6.54 | Gbar_D03_266246 | A/C | 0.08 | 6.92 |
| Gbar_D03_1107259 | A/C | 0.16 | 6.20 | Gbar_D03_1989485 | T/C | 0.18 | 6.45 | Gbar_D03_267782 | T/C | 0.08 | 6.21 |
| Gbar_D03_1110567 | A/G | 0.21 | 6.99 | Gbar_D03_1989856 | T/G | 0.10 | 6.10 | Gbar_D03_267841 | A/T | 0.08 | 6.32 |
| Gbar_D03_1116342 | G/A | 0.16 | 6.53 | Gbar_D03_2004879 | C/T | 0.08 | 6.64 | Gbar_D03_267866 | A/G | 0.08 | 6.44 |
| Gbar_D03_1116402 | A/G | 0.16 | 6.17 | Gbar_D03_2019628 | C/T | 0.09 | 7.05 | Gbar_D03_268910 | T/A | 0.08 | 6.10 |
| Gbar_D03_1117759 | A/G | 0.16 | 6.31 | Gbar_D03_2022615 | T/G | 0.09 | 7.06 | Gbar_D03_276754 | G/A | 0.08 | 6.59 |
| Gbar_D03_1121577 | G/A | 0.16 | 6.07 | Gbar_D03_2023546 | T/C | 0.18 | 6.13 | Gbar_D03_276895 | G/A | 0.08 | 6.10 |
| Gbar_D03_1126238 | T/C | 0.15 | 6.54 | Gbar_D03_2030544 | A/G | 0.09 | 6.22 | Gbar_D03_276918 | A/T | 0.08 | 6.16 |
| Gbar_D03_1131117 | A/G | 0.08 | 6.10 | Gbar_D03_2035736 | G/C | 0.09 | 6.54 | Gbar_D03_276927 | A/T | 0.08 | 6.12 |
| Gbar_D03_1133323 | G/A | 0.08 | 6.20 | Gbar_D03_2035829 | T/G | 0.09 | 6.50 | Gbar_D03_277326 | A/T | 0.08 | 6.18 |
| Gbar_D03_1140499 | C/A | 0.08 | 6.52 | Gbar_D03_2035835 | T/C | 0.09 | 6.72 | Gbar_D03_280479 | T/C | 0.08 | 6.13 |
| Gbar_D03_1141035 | C/T | 0.08 | 6.93 | Gbar_D03_2047669 | T/C | 0.18 | 6.30 | Gbar_D03_280483 | T/A | 0.08 | 6.03 |
| Gbar_D03_1141451 | G/T | 0.08 | 6.56 | Gbar_D03_2056886 | C/A | 0.09 | 6.61 | Gbar_D03_280605 | C/T | 0.08 | 6.20 |
| Gbar_D03_1142341 | A/G | 0.08 | 6.06 | Gbar_D03_2057743 | C/T | 0.09 | 7.54 | Gbar_D03_281099 | C/T | 0.08 | 6.00 |
| Gbar_D03_1159232 | A/G | 0.16 | 6.23 | Gbar_D03_2058409 | T/A | 0.10 | 6.05 | Gbar_D03_281564 | T/G | 0.08 | 6.16 |
| Gbar_D03_1174973 | G/A | 0.08 | 6.15 | Gbar_D03_2068470 | T/A | 0.09 | 6.14 | Gbar_D03_284501 | G/A | 0.08 | 6.17 |
| Gbar_D03_1180020 | G/A | 0.08 | 6.06 | Gbar_D03_2070020 | G/A | 0.09 | 6.23 | Gbar_D03_285695 | T/G | 0.07 | 6.42 |
| Gbar_D03_1187747 | T/C | 0.08 | 6.29 | Gbar_D03_2070146 | C/T | 0.09 | 6.08 | Gbar_D03_285970 | G/A | 0.08 | 6.44 |
| Gbar_D03_1192013 | G/C | 0.08 | 6.49 | Gbar_D03_2081936 | T/G | 0.08 | 7.27 | Gbar_D03_287296 | T/A | 0.08 | 6.33 |
| Gbar_D03_1224465 | A/C | 0.08 | 6.06 | Gbar_D03_2081948 | A/G | 0.08 | 7.10 | Gbar_D03_289432 | C/A | 0.08 | 6.31 |
| Gbar_D03_1269091 | A/T | 0.08 | 6.71 | Gbar_D03_2081955 | G/A | 0.08 | 6.24 | Gbar_D03_290201 | G/A | 0.08 | 6.54 |
| Gbar_D03_1277678 | A/T | 0.08 | 6.60 | Gbar_D03_2112513 | G/A | 0.08 | 6.82 | Gbar_D03_290416 | A/T | 0.08 | 6.82 |
| Gbar_D03_1279352 | C/T | 0.08 | 6.77 | Gbar_D03_2112544 | G/A | 0.08 | 6.35 | Gbar_D03_290586 | T/A | 0.08 | 6.61 |
| Gbar_D03_1279385 | G/A | 0.10 | 6.35 | Gbar_D03_2125937 | T/C | 0.09 | 6.48 | Gbar_D03_291025 | A/T | 0.08 | 6.51 |
| Gbar_D03_1281305 | C/T | 0.07 | 6.37 | Gbar_D03_2129927 | G/A | 0.08 | 6.92 | Gbar_D03_291710 | G/A | 0.08 | 6.32 |
| Gbar_D03_1319515 | C/T | 0.08 | 6.79 | Gbar_D03_2130833 | C/G | 0.08 | 6.66 | Gbar_D03_293205 | A/T | 0.08 | 6.15 |
| Gbar_D03_1329020 | A/G | 0.13 | 6.21 | Gbar_D03_2137861 | A/G | 0.08 | 7.10 | Gbar_D03_293228 | A/G | 0.08 | 6.06 |
| Gbar_D03_1330863 | A/G | 0.13 | 6.33 | Gbar_D03_2179602 | C/T | 0.10 | 6.03 | Gbar_D03_295844 | A/T | 0.08 | 6.03 |
| Gbar_D03_1332091 | T/G | 0.12 | 6.02 | Gbar_D03_2195873 | A/T | 0.46 | 6.08 | Gbar_D03_296209 | G/T | 0.08 | 6.63 |
| Gbar_D03_1336922 | A/G | 0.16 | 6.20 | Gbar_D03_220809 | A/T | 0.08 | 6.22 | Gbar_D03_296278 | A/T | 0.08 | 6.52 |
| Gbar_D03_1345907 | T/C | 0.16 | 6.01 | Gbar_D03_220948 | G/C | 0.08 | 6.07 | Gbar_D03_296456 | G/A | 0.08 | 6.03 |
| Gbar_D03_1348970 | A/C | 0.15 | 6.06 | Gbar_D03_2215669 | A/T | 0.17 | 6.51 | Gbar_D03_297638 | C/T | 0.08 | 6.38 |
| Gbar_D03_1359249 | A/C | 0.08 | 6.02 | Gbar_D03_222160 | T/C | 0.08 | 6.20 | Gbar_D03_297696 | C/G | 0.08 | 6.19 |
| Gbar_D03_1359810 | A/G | 0.09 | 7.68 | Gbar_D03_222186 | T/C | 0.08 | 6.09 | Gbar_D03_297725 | T/C | 0.08 | 6.62 |
| Gbar_D03_1400528 | A/G | 0.08 | 6.15 | Gbar_D03_222231 | A/G | 0.08 | 6.30 | Gbar_D03_297808 | G/C | 0.08 | 6.62 |
| Gbar_D03_1456870 | G/A | 0.08 | 6.50 | Gbar_D03_222232 | A/G | 0.08 | 6.30 | Gbar_D03_298221 | A/T | 0.08 | 6.19 |
| Gbar_D03_1460925 | G/C | 0.08 | 6.23 | Gbar_D03_2222437 | T/C | 0.09 | 6.02 | Gbar_D03_298740 | G/A | 0.08 | 6.14 |
| Gbar_D03_1471416 | G/A | 0.09 | 6.73 | Gbar_D03_222249 | A/G | 0.08 | 6.40 | Gbar_D03_298944 | T/C | 0.08 | 6.09 |
| Gbar_D03_1471482 | C/A | 0.09 | 6.29 | Gbar_D03_2223697 | G/T | 0.09 | 6.25 | Gbar_D03_300361 | G/A | 0.08 | 6.15 |
| Gbar_D03_1501667 | T/C | 0.09 | 6.53 | Gbar_D03_2224946 | A/G | 0.16 | 6.43 | Gbar_D03_302594 | A/G | 0.07 | 6.53 |
| Gbar_D03_1501683 | C/T | 0.08 | 6.34 | Gbar_D03_222591 | C/G | 0.08 | 6.34 | Gbar_D03_305070 | T/G | 0.08 | 6.26 |
| Gbar_D03_1505214 | T/G | 0.09 | 6.07 | Gbar_D03_222616 | A/G | 0.08 | 6.50 | Gbar_D03_305083 | A/G | 0.08 | 6.29 |
| Gbar_D03_1505218 | T/C | 0.09 | 6.06 | Gbar_D03_222655 | G/T | 0.08 | 6.69 | Gbar_D03_307978 | A/G | 0.07 | 6.03 |
| Gbar_D03_1509599 | T/C | 0.08 | 6.10 | Gbar_D03_222693 | T/C | 0.07 | 6.10 | Gbar_D03_307986 | G/A | 0.07 | 6.14 |
| Gbar_D03_1513731 | A/T | 0.08 | 6.32 | Gbar_D03_2228451 | A/G | 0.14 | 6.54 | Gbar_D03_308087 | T/C | 0.08 | 6.65 |
| Gbar_D03_1513733 | C/A | 0.09 | 6.40 | Gbar_D03_222937 | G/A | 0.08 | 6.59 | Gbar_D03_310501 | T/C | 0.07 | 6.22 |
| Gbar_D03_1513734 | C/A | 0.09 | 6.11 | Gbar_D03_222941 | C/A | 0.08 | 6.10 | Gbar_D03_315553 | C/G | 0.08 | 6.91 |
| Gbar_D03_1537617 | T/G | 0.08 | 6.24 | Gbar_D03_224081 | T/C | 0.08 | 6.13 | Gbar_D03_315594 | A/G | 0.08 | 6.51 |
| Gbar_D03_1541480 | A/G | 0.08 | 6.24 | Gbar_D03_224097 | C/T | 0.08 | 6.32 | Gbar_D03_317643 | T/G | 0.08 | 6.61 |
| Gbar_D03_1541489 | T/A | 0.09 | 6.33 | Gbar_D03_224098 | G/T | 0.08 | 6.47 | Gbar_D03_317762 | G/A | 0.08 | 6.11 |
| Gbar_D03_1601122 | C/T | 0.08 | 6.32 | Gbar_D03_224122 | T/C | 0.08 | 6.62 | Gbar_D03_317766 | G/A | 0.08 | 6.04 |
| Gbar_D03_1601160 | C/T | 0.08 | 6.20 | Gbar_D03_2249026 | C/T | 0.09 | 7.37 | Gbar_D03_325034 | C/T | 0.08 | 6.26 |
| Gbar_D03_1620201 | A/C | 0.08 | 6.38 | Gbar_D03_2249706 | T/C | 0.15 | 6.07 | Gbar_D03_326108 | C/T | 0.08 | 6.32 |
| Gbar_D03_1672432 | A/T | 0.08 | 6.18 | Gbar_D03_2255845 | T/G | 0.14 | 6.68 | Gbar_D03_327187 | T/A | 0.07 | 6.08 |
| Gbar_D03_1686833 | A/G | 0.08 | 6.15 | Gbar_D03_2255926 | T/C | 0.14 | 6.17 | Gbar_D03_327895 | A/G | 0.08 | 6.35 |
| Gbar_D03_1699544 | G/A | 0.08 | 6.14 | Gbar_D03_2256558 | T/A | 0.14 | 6.55 | Gbar_D03_335127 | A/C | 0.08 | 6.48 |
| Gbar_D03_1700917 | T/G | 0.08 | 6.47 | Gbar_D03_2256560 | T/A | 0.14 | 7.13 | Gbar_D03_336173 | T/C | 0.08 | 6.26 |
| Gbar_D03_1705967 | A/T | 0.08 | 6.52 | Gbar_D03_2259062 | G/T | 0.08 | 6.16 | Gbar_D03_336255 | A/G | 0.08 | 6.44 |
| Gbar_D03_1714170 | A/G | 0.09 | 6.53 | Gbar_D03_226153 | A/T | 0.08 | 6.15 | Gbar_D03_337300 | A/T | 0.08 | 6.37 |
| Gbar_D03_1763630 | G/T | 0.08 | 6.22 | Gbar_D03_226586 | C/T | 0.07 | 6.42 | Gbar_D03_337548 | T/G | 0.07 | 6.18 |
| Gbar_D03_1805146 | G/A | 0.09 | 6.11 | Gbar_D03_2268413 | A/C | 0.09 | 6.07 | Gbar_D03_337829 | G/T | 0.08 | 6.44 |
| Gbar_D03_1824899 | T/C | 0.18 | 6.51 | Gbar_D03_227406 | C/T | 0.08 | 6.19 | Gbar_D03_338447 | A/T | 0.08 | 6.01 |
| Gbar_D03_1833455 | T/A | 0.09 | 6.38 | Gbar_D03_227599 | A/T | 0.08 | 6.58 | Gbar_D03_338578 | C/T | 0.08 | 6.18 |
| Gbar_D03_1838463 | G/A | 0.09 | 6.05 | Gbar_D03_228548 | G/A | 0.07 | 6.38 | Gbar_D03_339295 | C/T | 0.08 | 6.08 |
| Gbar_D03_1839061 | G/A | 0.09 | 6.22 | Gbar_D03_228570 | A/G | 0.08 | 6.43 | Gbar_D03_339349 | C/A | 0.08 | 6.09 |
| Gbar_D03_1846306 | T/G | 0.18 | 6.07 | Gbar_D03_228596 | A/G | 0.08 | 6.42 | Gbar_D03_341171 | G/A | 0.08 | 6.86 |
| Gbar_D03_1853808 | T/C | 0.09 | 7.28 | Gbar_D03_228815 | T/C | 0.08 | 6.04 | Gbar_D03_341305 | C/A | 0.08 | 6.16 |
| Gbar_D03_1854675 | A/G | 0.09 | 6.13 | Gbar_D03_229831 | C/T | 0.08 | 6.20 | Gbar_D03_342392 | T/A | 0.08 | 6.30 |
| Gbar_D03_1855408 | T/C | 0.09 | 6.13 | Gbar_D03_229844 | T/C | 0.08 | 6.20 | Gbar_D03_342724 | A/G | 0.08 | 6.08 |
| Gbar_D03_1855413 | C/T | 0.09 | 6.71 | Gbar_D03_230553 | C/G | 0.08 | 6.49 | Gbar_D03_342870 | A/G | 0.08 | 6.38 |
| Gbar_D03_1855570 | T/C | 0.09 | 6.55 | Gbar_D03_230585 | A/G | 0.08 | 6.11 | Gbar_D03_343188 | C/G | 0.08 | 6.32 |
| Gbar_D03_1860152 | G/A | 0.09 | 6.19 | Gbar_D03_231767 | C/A | 0.08 | 6.17 | Gbar_D03_343677 | T/C | 0.08 | 6.14 |
| Gbar_D03_1860402 | A/G | 0.08 | 6.23 | Gbar_D03_231982 | A/T | 0.08 | 6.01 | Gbar_D03_344490 | T/C | 0.08 | 6.24 |
| Gbar_D03_1860417 | T/C | 0.08 | 6.33 | Gbar_D03_232520 | C/A | 0.08 | 6.06 | Gbar_D03_345426 | G/A | 0.08 | 6.17 |
| Gbar_D03_1860429 | G/A | 0.08 | 6.88 | Gbar_D03_232810 | G/A | 0.09 | 6.25 | Gbar_D03_346133 | A/T | 0.08 | 6.82 |
| Gbar_D03_1862522 | T/G | 0.09 | 6.35 | Gbar_D03_233018 | T/C | 0.08 | 6.18 | Gbar_D03_346147 | G/T | 0.08 | 7.06 |
| Gbar_D03_1876166 | G/C | 0.09 | 7.07 | Gbar_D03_233431 | G/T | 0.07 | 6.36 | Gbar_D03_346174 | A/T | 0.08 | 7.05 |
| Gbar_D03_1878030 | T/C | 0.08 | 7.75 | Gbar_D03_236618 | C/T | 0.08 | 6.37 | Gbar_D03_347075 | G/A | 0.08 | 6.07 |
| Gbar_D03_1878576 | G/A | 0.10 | 6.39 | Gbar_D03_236653 | A/G | 0.08 | 6.53 | Gbar_D03_347151 | T/C | 0.08 | 6.40 |
| Gbar_D03_1879874 | T/C | 0.09 | 7.43 | Gbar_D03_236777 | T/C | 0.08 | 6.66 | Gbar_D03_349195 | A/G | 0.08 | 6.08 |
| Gbar_D03_1881025 | G/A | 0.09 | 7.19 | Gbar_D03_237064 | G/T | 0.08 | 6.18 | Gbar_D03_351193 | C/G | 0.08 | 6.64 |
| Gbar_D03_1884123 | A/G | 0.08 | 6.11 | Gbar_D03_237071 | C/A | 0.08 | 6.41 | Gbar_D03_351798 | G/A | 0.07 | 6.48 |
| Gbar_D03_1888773 | G/T | 0.10 | 6.13 | Gbar_D03_237378 | G/A | 0.08 | 6.95 | Gbar_D03_351883 | A/G | 0.08 | 6.47 |
| Gbar_D03_1895389 | G/A | 0.09 | 6.17 | Gbar_D03_237550 | T/A | 0.08 | 6.23 | Gbar_D03_351895 | A/G | 0.08 | 6.59 |
| Gbar_D03_1896583 | T/C | 0.09 | 6.02 | Gbar_D03_237715 | C/T | 0.08 | 6.01 | Gbar_D03_351917 | G/A | 0.08 | 6.19 |

**Table S2.** List of the associated SNPs identified in different datasets through GWAS for *FW* resistance in Sea Island cotton. (Continued)

| SNP | Ref/Alt | MAF | -log (p-value) | SNP | Ref/Alt | MAF | -log (p-value) | SNP | Ref/Alt | MAF | -log (p-value) |
| --- | --- | --- | --- | --- | --- | --- | --- | --- | --- | --- | --- |
| **2015** |  |  |  | Gbar_D03_435671 | G/T | 0.08 | 6.77 | Gbar_D03_495334 | T/C | 0.08 | 6.51 |
| Gbar_D03_351927 | A/G | 0.08 | 6.57 | Gbar_D03_437353 | G/C | 0.08 | 6.12 | Gbar_D03_496135 | A/G | 0.07 | 6.22 |
| Gbar_D03_353095 | T/C | 0.08 | 6.91 | Gbar_D03_437356 | C/T | 0.08 | 6.06 | Gbar_D03_496437 | G/T | 0.08 | 6.85 |
| Gbar_D03_353785 | T/C | 0.07 | 6.13 | Gbar_D03_437487 | A/G | 0.08 | 7.07 | Gbar_D03_496666 | G/A | 0.08 | 6.33 |
| Gbar_D03_354247 | A/G | 0.08 | 6.01 | Gbar_D03_437532 | C/T | 0.08 | 6.81 | Gbar_D03_496839 | A/T | 0.08 | 6.10 |
| Gbar_D03_355098 | C/T | 0.07 | 6.59 | Gbar_D03_437877 | C/T | 0.08 | 6.07 | Gbar_D03_497738 | A/C | 0.07 | 6.06 |
| Gbar_D03_357211 | T/C | 0.08 | 6.19 | Gbar_D03_439082 | C/T | 0.08 | 6.32 | Gbar_D03_497951 | G/A | 0.08 | 6.56 |
| Gbar_D03_357800 | G/A | 0.08 | 6.06 | Gbar_D03_439089 | G/A | 0.08 | 6.29 | Gbar_D03_498371 | T/A | 0.08 | 6.31 |
| Gbar_D03_359041 | G/A | 0.08 | 6.29 | Gbar_D03_439090 | T/A | 0.08 | 6.26 | Gbar_D03_500515 | A/G | 0.08 | 6.32 |
| Gbar_D03_361972 | G/A | 0.08 | 6.30 | Gbar_D03_439136 | A/G | 0.08 | 6.09 | Gbar_D03_500815 | C/T | 0.08 | 6.09 |
| Gbar_D03_362303 | C/T | 0.08 | 6.26 | Gbar_D03_439359 | T/C | 0.08 | 6.08 | Gbar_D03_501474 | A/C | 0.08 | 6.46 |
| Gbar_D03_362767 | G/A | 0.08 | 6.32 | Gbar_D03_439612 | G/A | 0.08 | 6.63 | Gbar_D03_501600 | C/T | 0.08 | 6.13 |
| Gbar_D03_363140 | G/T | 0.08 | 6.29 | Gbar_D03_439614 | A/G | 0.08 | 6.56 | Gbar_D03_502048 | C/T | 0.08 | 6.57 |
| Gbar_D03_363238 | G/A | 0.08 | 6.06 | Gbar_D03_439883 | A/G | 0.08 | 6.21 | Gbar_D03_502197 | T/C | 0.08 | 6.24 |
| Gbar_D03_366106 | G/A | 0.08 | 6.22 | Gbar_D03_440489 | T/C | 0.07 | 6.60 | Gbar_D03_502804 | A/G | 0.07 | 6.57 |
| Gbar_D03_367828 | T/C | 0.08 | 6.68 | Gbar_D03_441607 | G/T | 0.08 | 6.56 | Gbar_D03_502823 | C/T | 0.07 | 6.21 |
| Gbar_D03_368207 | T/A | 0.08 | 6.22 | Gbar_D03_442128 | C/T | 0.08 | 6.03 | Gbar_D03_503118 | T/C | 0.08 | 6.34 |
| Gbar_D03_368537 | G/A | 0.08 | 6.08 | Gbar_D03_442279 | G/A | 0.08 | 6.13 | Gbar_D03_503165 | T/C | 0.08 | 6.35 |
| Gbar_D03_368561 | A/G | 0.08 | 6.33 | Gbar_D03_443813 | G/A | 0.08 | 6.51 | Gbar_D03_503175 | T/A | 0.08 | 6.27 |
| Gbar_D03_368579 | T/C | 0.08 | 6.10 | Gbar_D03_443832 | T/C | 0.08 | 6.39 | Gbar_D03_503307 | C/A | 0.08 | 6.11 |
| Gbar_D03_368619 | G/A | 0.08 | 6.57 | Gbar_D03_446603 | C/T | 0.08 | 6.17 | Gbar_D03_503374 | C/T | 0.08 | 6.14 |
| Gbar_D03_369041 | T/C | 0.08 | 6.09 | Gbar_D03_446682 | C/T | 0.08 | 6.51 | Gbar_D03_504141 | A/G | 0.08 | 6.11 |
| Gbar_D03_369830 | T/G | 0.08 | 6.54 | Gbar_D03_446956 | T/G | 0.08 | 6.26 | Gbar_D03_504411 | G/A | 0.08 | 6.22 |
| Gbar_D03_369979 | A/G | 0.08 | 6.53 | Gbar_D03_447239 | G/A | 0.08 | 6.34 | Gbar_D03_504576 | C/T | 0.08 | 6.28 |
| Gbar_D03_371073 | G/A | 0.08 | 6.26 | Gbar_D03_448346 | C/G | 0.08 | 6.85 | Gbar_D03_504805 | T/C | 0.08 | 6.04 |
| Gbar_D03_371111 | C/T | 0.08 | 6.30 | Gbar_D03_449644 | T/G | 0.08 | 6.17 | Gbar_D03_505033 | A/G | 0.08 | 6.41 |
| Gbar_D03_371364 | G/A | 0.08 | 6.24 | Gbar_D03_449901 | G/T | 0.08 | 6.15 | Gbar_D03_505069 | G/A | 0.07 | 6.88 |
| Gbar_D03_371913 | T/C | 0.08 | 6.27 | Gbar_D03_450582 | A/G | 0.08 | 6.31 | Gbar_D03_505971 | C/T | 0.07 | 6.04 |
| Gbar_D03_372618 | C/T | 0.08 | 6.26 | Gbar_D03_450697 | A/T | 0.07 | 6.22 | Gbar_D03_506076 | T/C | 0.08 | 6.32 |
| Gbar_D03_373236 | C/T | 0.08 | 6.04 | Gbar_D03_451008 | T/C | 0.08 | 6.41 | Gbar_D03_506177 | C/T | 0.08 | 6.15 |
| Gbar_D03_377835 | T/C | 0.08 | 6.09 | Gbar_D03_451901 | G/A | 0.08 | 6.13 | Gbar_D03_506188 | A/C | 0.08 | 6.12 |
| Gbar_D03_378142 | G/A | 0.08 | 6.45 | Gbar_D03_452383 | G/A | 0.08 | 6.99 | Gbar_D03_506264 | C/T | 0.07 | 6.71 |
| Gbar_D03_379739 | G/A | 0.08 | 6.44 | Gbar_D03_453697 | C/G | 0.08 | 6.09 | Gbar_D03_506275 | A/T | 0.07 | 6.86 |
| Gbar_D03_380281 | A/C | 0.08 | 6.03 | Gbar_D03_453752 | G/A | 0.07 | 6.54 | Gbar_D03_506428 | A/C | 0.07 | 6.46 |
| Gbar_D03_380949 | C/G | 0.08 | 6.23 | Gbar_D03_453779 | C/T | 0.08 | 6.16 | Gbar_D03_506443 | C/T | 0.07 | 6.14 |
| Gbar_D03_381478 | T/G | 0.08 | 6.27 | Gbar_D03_453781 | T/A | 0.07 | 6.01 | Gbar_D03_506471 | G/A | 0.07 | 6.42 |
| Gbar_D03_382233 | G/A | 0.08 | 6.42 | Gbar_D03_453833 | A/G | 0.08 | 6.39 | Gbar_D03_506491 | C/T | 0.08 | 6.26 |
| Gbar_D03_382256 | G/A | 0.08 | 6.55 | Gbar_D03_453867 | T/C | 0.08 | 6.12 | Gbar_D03_506522 | A/G | 0.07 | 6.40 |
| Gbar_D03_382485 | A/T | 0.08 | 6.33 | Gbar_D03_453869 | G/A | 0.08 | 6.33 | Gbar_D03_506634 | G/A | 0.07 | 6.01 |
| Gbar_D03_382861 | T/C | 0.07 | 6.37 | Gbar_D03_454071 | G/A | 0.07 | 6.03 | Gbar_D03_506792 | A/C | 0.08 | 6.60 |
| Gbar_D03_382874 | T/C | 0.07 | 6.54 | Gbar_D03_454158 | T/C | 0.08 | 6.41 | Gbar_D03_506900 | C/T | 0.07 | 6.10 |
| Gbar_D03_383853 | C/T | 0.08 | 6.09 | Gbar_D03_454523 | G/A | 0.08 | 6.03 | Gbar_D03_507303 | A/G | 0.08 | 6.76 |
| Gbar_D03_383926 | G/A | 0.08 | 6.20 | Gbar_D03_456764 | A/C | 0.08 | 6.48 | Gbar_D03_507341 | G/A | 0.07 | 6.01 |
| Gbar_D03_384657 | A/G | 0.08 | 6.40 | Gbar_D03_457126 | A/G | 0.08 | 6.60 | Gbar_D03_507512 | T/C | 0.08 | 6.07 |
| Gbar_D03_385751 | A/G | 0.08 | 6.66 | Gbar_D03_457147 | A/G | 0.08 | 6.74 | Gbar_D03_507538 | G/A | 0.08 | 6.19 |
| Gbar_D03_385959 | T/G | 0.08 | 6.38 | Gbar_D03_457555 | T/A | 0.08 | 6.11 | Gbar_D03_507601 | T/C | 0.08 | 6.37 |
| Gbar_D03_388400 | T/A | 0.08 | 6.11 | Gbar_D03_457649 | T/G | 0.08 | 6.32 | Gbar_D03_507695 | G/A | 0.08 | 6.26 |
| Gbar_D03_388455 | T/C | 0.08 | 7.15 | Gbar_D03_457921 | T/C | 0.07 | 6.01 | Gbar_D03_508555 | A/G | 0.08 | 7.19 |
| Gbar_D03_392094 | T/A | 0.08 | 6.30 | Gbar_D03_458018 | A/G | 0.08 | 6.68 | Gbar_D03_509339 | T/C | 0.08 | 6.04 |
| Gbar_D03_396757 | G/T | 0.08 | 6.01 | Gbar_D03_458610 | T/G | 0.08 | 6.08 | Gbar_D03_509731 | G/A | 0.08 | 6.20 |
| Gbar_D03_396864 | T/C | 0.08 | 6.04 | Gbar_D03_459639 | G/C | 0.08 | 6.24 | Gbar_D03_510004 | C/T | 0.07 | 6.74 |
| Gbar_D03_401365 | G/A | 0.08 | 6.47 | Gbar_D03_461284 | A/T | 0.07 | 6.60 | Gbar_D03_510058 | C/T | 0.07 | 6.35 |
| Gbar_D03_401592 | T/C | 0.08 | 6.47 | Gbar_D03_462175 | C/A | 0.08 | 6.21 | Gbar_D03_510314 | C/T | 0.08 | 6.03 |
| Gbar_D03_402831 | C/T | 0.08 | 6.27 | Gbar_D03_462575 | G/A | 0.08 | 6.69 | Gbar_D03_510625 | A/G | 0.08 | 6.09 |
| Gbar_D03_402897 | G/A | 0.08 | 6.61 | Gbar_D03_462735 | G/A | 0.08 | 6.46 | Gbar_D03_510669 | G/T | 0.08 | 6.51 |
| Gbar_D03_403327 | T/C | 0.08 | 6.34 | Gbar_D03_462750 | C/T | 0.08 | 6.06 | Gbar_D03_510852 | A/T | 0.08 | 6.42 |
| Gbar_D03_403792 | A/C | 0.08 | 6.13 | Gbar_D03_462782 | A/G | 0.08 | 6.19 | Gbar_D03_510923 | T/G | 0.07 | 6.73 |
| Gbar_D03_405154 | G/T | 0.08 | 6.63 | Gbar_D03_462898 | C/T | 0.08 | 6.63 | Gbar_D03_511182 | A/G | 0.08 | 6.38 |
| Gbar_D03_405198 | G/A | 0.08 | 6.19 | Gbar_D03_462912 | C/G | 0.08 | 6.42 | Gbar_D03_512774 | G/A | 0.08 | 6.32 |
| Gbar_D03_405700 | A/T | 0.08 | 6.39 | Gbar_D03_463030 | G/A | 0.07 | 7.68 | Gbar_D03_516561 | A/G | 0.08 | 6.35 |
| Gbar_D03_405726 | A/G | 0.08 | 6.47 | Gbar_D03_463042 | T/A | 0.07 | 7.39 | Gbar_D03_518070 | G/A | 0.08 | 6.70 |
| Gbar_D03_405730 | A/T | 0.08 | 6.46 | Gbar_D03_463069 | T/G | 0.08 | 6.69 | Gbar_D03_519276 | A/G | 0.08 | 6.28 |
| Gbar_D03_406053 | G/A | 0.08 | 6.51 | Gbar_D03_463234 | A/G | 0.08 | 6.26 | Gbar_D03_519537 | G/T | 0.08 | 6.13 |
| Gbar_D03_406139 | T/C | 0.08 | 6.43 | Gbar_D03_463271 | C/A | 0.08 | 6.26 | Gbar_D03_519680 | C/G | 0.07 | 6.16 |
| Gbar_D03_406564 | C/T | 0.08 | 6.14 | Gbar_D03_463326 | T/C | 0.08 | 6.53 | Gbar_D03_519752 | A/G | 0.08 | 6.59 |
| Gbar_D03_406637 | C/A | 0.08 | 6.55 | Gbar_D03_463364 | A/C | 0.08 | 6.32 | Gbar_D03_519828 | C/T | 0.08 | 6.27 |
| Gbar_D03_409945 | C/T | 0.08 | 6.83 | Gbar_D03_463691 | A/C | 0.08 | 6.71 | Gbar_D03_519894 | T/C | 0.08 | 6.45 |
| Gbar_D03_410928 | G/A | 0.08 | 6.41 | Gbar_D03_464328 | T/G | 0.08 | 6.01 | Gbar_D03_519902 | G/A | 0.08 | 6.51 |
| Gbar_D03_411252 | T/G | 0.08 | 6.23 | Gbar_D03_465332 | C/T | 0.08 | 6.48 | Gbar_D03_519931 | C/T | 0.08 | 6.67 |
| Gbar_D03_411418 | A/G | 0.08 | 6.11 | Gbar_D03_465477 | G/T | 0.08 | 6.13 | Gbar_D03_520101 | C/T | 0.08 | 6.43 |
| Gbar_D03_411439 | T/C | 0.08 | 6.15 | Gbar_D03_466275 | G/A | 0.08 | 6.09 | Gbar_D03_520368 | C/A | 0.08 | 6.05 |
| Gbar_D03_411581 | G/A | 0.08 | 6.23 | Gbar_D03_468281 | T/G | 0.08 | 6.72 | Gbar_D03_520624 | T/A | 0.08 | 6.18 |
| Gbar_D03_411885 | C/T | 0.07 | 6.22 | Gbar_D03_468539 | A/G | 0.07 | 6.17 | Gbar_D03_520660 | C/T | 0.08 | 6.51 |
| Gbar_D03_412682 | C/A | 0.07 | 6.30 | Gbar_D03_469034 | G/C | 0.07 | 6.71 | Gbar_D03_523860 | C/T | 0.08 | 6.57 |
| Gbar_D03_412737 | G/A | 0.08 | 6.61 | Gbar_D03_470216 | T/C | 0.08 | 6.14 | Gbar_D03_523865 | C/T | 0.08 | 6.56 |
| Gbar_D03_412749 | A/C | 0.08 | 6.54 | Gbar_D03_471549 | T/C | 0.08 | 6.16 | Gbar_D03_524047 | C/T | 0.08 | 6.30 |
| Gbar_D03_413017 | T/A | 0.08 | 6.25 | Gbar_D03_471597 | A/G | 0.07 | 6.31 | Gbar_D03_524133 | T/C | 0.08 | 6.55 |
| Gbar_D03_414289 | T/A | 0.08 | 6.30 | Gbar_D03_471649 | A/G | 0.07 | 6.17 | Gbar_D03_525581 | G/A | 0.08 | 6.06 |
| Gbar_D03_416021 | G/A | 0.07 | 6.23 | Gbar_D03_471728 | A/G | 0.08 | 6.48 | Gbar_D03_526472 | A/G | 0.08 | 6.29 |
| Gbar_D03_416025 | C/T | 0.07 | 6.20 | Gbar_D03_471731 | A/G | 0.08 | 6.43 | Gbar_D03_527972 | C/T | 0.08 | 6.01 |
| Gbar_D03_416026 | A/G | 0.07 | 6.26 | Gbar_D03_472231 | C/T | 0.08 | 6.24 | Gbar_D03_528129 | A/C | 0.07 | 6.10 |
| Gbar_D03_417385 | G/C | 0.08 | 6.02 | Gbar_D03_473099 | C/T | 0.08 | 6.23 | Gbar_D03_528807 | A/G | 0.08 | 6.01 |
| Gbar_D03_417844 | G/A | 0.07 | 6.17 | Gbar_D03_474411 | C/T | 0.08 | 6.27 | Gbar_D03_530107 | G/A | 0.08 | 6.24 |
| Gbar_D03_418695 | T/C | 0.08 | 6.13 | Gbar_D03_474425 | T/C | 0.08 | 6.09 | Gbar_D03_530851 | A/G | 0.08 | 6.05 |
| Gbar_D03_418726 | A/G | 0.08 | 6.15 | Gbar_D03_474723 | G/T | 0.08 | 6.05 | Gbar_D03_531756 | G/T | 0.08 | 6.32 |
| Gbar_D03_419056 | G/T | 0.08 | 6.17 | Gbar_D03_475216 | C/T | 0.08 | 6.53 | Gbar_D03_532612 | T/G | 0.08 | 6.50 |
| Gbar_D03_419441 | C/A | 0.08 | 6.26 | Gbar_D03_475398 | G/A | 0.08 | 6.12 | Gbar_D03_533744 | T/C | 0.08 | 6.21 |
| Gbar_D03_423244 | T/G | 0.07 | 6.78 | Gbar_D03_476170 | G/T | 0.08 | 6.41 | Gbar_D03_534462 | G/C | 0.08 | 6.02 |
| Gbar_D03_427960 | G/A | 0.07 | 6.59 | Gbar_D03_477311 | T/A | 0.08 | 6.33 | Gbar_D03_535883 | T/A | 0.08 | 7.48 |
| Gbar_D03_429289 | C/T | 0.07 | 6.32 | Gbar_D03_478976 | T/C | 0.08 | 6.27 | Gbar_D03_536094 | A/G | 0.07 | 6.13 |
| Gbar_D03_429383 | A/G | 0.08 | 6.19 | Gbar_D03_479959 | C/T | 0.07 | 6.00 | Gbar_D03_536096 | G/A | 0.07 | 6.19 |
| Gbar_D03_430264 | A/C | 0.08 | 6.56 | Gbar_D03_479982 | G/A | 0.07 | 6.20 | Gbar_D03_536850 | A/T | 0.08 | 6.29 |
| Gbar_D03_431084 | C/G | 0.08 | 6.48 | Gbar_D03_480663 | C/T | 0.08 | 6.40 | Gbar_D03_537009 | T/C | 0.08 | 6.03 |
| Gbar_D03_431689 | T/C | 0.08 | 6.20 | Gbar_D03_480666 | T/C | 0.08 | 6.14 | Gbar_D03_537091 | G/T | 0.08 | 6.36 |
| Gbar_D03_432032 | T/C | 0.08 | 6.16 | Gbar_D03_481529 | G/A | 0.08 | 6.61 | Gbar_D03_537524 | T/C | 0.08 | 6.05 |
| Gbar_D03_432048 | A/G | 0.08 | 6.32 | Gbar_D03_482747 | A/G | 0.08 | 6.61 | Gbar_D03_538327 | C/T | 0.08 | 6.16 |
| Gbar_D03_432073 | A/G | 0.08 | 6.01 | Gbar_D03_482860 | C/T | 0.08 | 6.22 | Gbar_D03_538413 | C/T | 0.08 | 6.04 |
| Gbar_D03_432198 | C/T | 0.08 | 6.13 | Gbar_D03_482995 | C/T | 0.08 | 6.20 | Gbar_D03_540437 | T/A | 0.07 | 6.13 |
| Gbar_D03_432703 | A/C | 0.08 | 7.11 | Gbar_D03_489394 | T/C | 0.08 | 6.44 | Gbar_D03_540438 | T/C | 0.07 | 6.25 |
| Gbar_D03_433030 | C/T | 0.08 | 6.50 | Gbar_D03_489419 | C/T | 0.08 | 6.11 | Gbar_D03_541604 | C/T | 0.08 | 6.56 |
| Gbar_D03_434058 | T/G | 0.08 | 6.67 | Gbar_D03_489609 | A/G | 0.08 | 6.07 | Gbar_D03_541634 | T/A | 0.08 | 6.40 |
| Gbar_D03_434175 | G/A | 0.08 | 6.11 | Gbar_D03_490683 | G/A | 0.08 | 6.04 | Gbar_D03_541711 | T/C | 0.07 | 6.44 |
| Gbar_D03_434363 | A/G | 0.08 | 6.13 | Gbar_D03_491506 | C/T | 0.08 | 6.30 | Gbar_D03_541848 | T/A | 0.07 | 6.31 |
| Gbar_D03_434386 | G/A | 0.08 | 6.35 | Gbar_D03_491514 | A/T | 0.08 | 6.24 | Gbar_D03_542054 | C/A | 0.07 | 6.20 |
| Gbar_D03_434425 | A/G | 0.08 | 6.36 | Gbar_D03_493428 | G/T | 0.08 | 6.22 | Gbar_D03_542293 | A/G | 0.08 | 6.31 |
| Gbar_D03_434460 | T/C | 0.08 | 6.02 | Gbar_D03_493656 | G/C | 0.08 | 6.39 | Gbar_D03_542441 | A/G | 0.08 | 6.22 |
| Gbar_D03_434671 | C/T | 0.08 | 6.25 | Gbar_D03_493884 | G/A | 0.08 | 6.04 | Gbar_D03_542785 | A/C | 0.08 | 6.09 |
| Gbar_D03_435100 | C/T | 0.07 | 7.17 | Gbar_D03_494172 | A/G | 0.08 | 6.35 | Gbar_D03_542796 | A/C | 0.08 | 6.17 |
| Gbar_D03_435277 | G/C | 0.08 | 6.18 | Gbar_D03_494449 | C/A | 0.08 | 6.33 | Gbar_D03_543292 | A/C | 0.08 | 6.82 |
| Gbar_D03_435301 | A/T | 0.08 | 6.30 | Gbar_D03_494851 | T/C | 0.08 | 6.40 | Gbar_D03_543367 | A/T | 0.08 | 6.04 |

**Table S2.** List of the associated SNPs identified in different datasets through GWAS for *FW* resistance in Sea Island cotton. (Continued)

| SNP | Ref/Alt | MAF | -log (p-value) | SNP | Ref/Alt | MAF | -log (p-value) | SNP | Ref/Alt | MAF | -log (p-value) |
| --- | --- | --- | --- | --- | --- | --- | --- | --- | --- | --- | --- |
| **2015** |  |  |  | Gbar_D03_613067 | T/C | 0.08 | 6.52 | Gbar_D03_666372 | G/A | 0.08 | 6.22 |
| Gbar_D03_545273 | C/T | 0.08 | 6.26 | Gbar_D03_613631 | C/A | 0.08 | 7.08 | Gbar_D03_667281 | C/A | 0.08 | 6.97 |
| Gbar_D03_545286 | A/G | 0.08 | 6.11 | Gbar_D03_613955 | A/G | 0.08 | 6.34 | Gbar_D03_667390 | T/A | 0.08 | 6.85 |
| Gbar_D03_545509 | G/A | 0.08 | 6.19 | Gbar_D03_614536 | T/C | 0.08 | 6.22 | Gbar_D03_667639 | A/C | 0.08 | 6.23 |
| Gbar_D03_545512 | A/T | 0.07 | 6.18 | Gbar_D03_614896 | T/C | 0.08 | 7.00 | Gbar_D03_669501 | G/A | 0.08 | 7.22 |
| Gbar_D03_546783 | A/G | 0.08 | 6.37 | Gbar_D03_614953 | T/C | 0.08 | 7.00 | Gbar_D03_670305 | A/G | 0.08 | 6.14 |
| Gbar_D03_546915 | T/A | 0.08 | 6.50 | Gbar_D03_615180 | A/G | 0.08 | 6.77 | Gbar_D03_670681 | C/A | 0.08 | 6.66 |
| Gbar_D03_547140 | A/G | 0.08 | 6.20 | Gbar_D03_615713 | T/A | 0.08 | 6.61 | Gbar_D03_671090 | T/C | 0.07 | 6.52 |
| Gbar_D03_548405 | T/C | 0.08 | 6.53 | Gbar_D03_615776 | T/C | 0.08 | 6.88 | Gbar_D03_671656 | A/T | 0.08 | 6.24 |
| Gbar_D03_548415 | C/A | 0.09 | 6.48 | Gbar_D03_615790 | A/T | 0.08 | 7.04 | Gbar_D03_671919 | C/T | 0.08 | 6.47 |
| Gbar_D03_548506 | A/G | 0.08 | 6.32 | Gbar_D03_616070 | G/A | 0.08 | 6.65 | Gbar_D03_671942 | G/T | 0.07 | 6.37 |
| Gbar_D03_551060 | C/T | 0.08 | 6.25 | Gbar_D03_616321 | T/C | 0.09 | 6.29 | Gbar_D03_672496 | G/A | 0.08 | 6.32 |
| Gbar_D03_551093 | G/T | 0.08 | 6.47 | Gbar_D03_616638 | T/C | 0.08 | 6.33 | Gbar_D03_673021 | C/T | 0.08 | 6.67 |
| Gbar_D03_552199 | A/T | 0.08 | 6.83 | Gbar_D03_617081 | G/A | 0.08 | 6.60 | Gbar_D03_673181 | A/C | 0.08 | 6.42 |
| Gbar_D03_554990 | G/A | 0.07 | 6.06 | Gbar_D03_617139 | C/T | 0.08 | 7.18 | Gbar_D03_673236 | T/G | 0.08 | 6.50 |
| Gbar_D03_555903 | A/G | 0.07 | 6.25 | Gbar_D03_619689 | G/A | 0.08 | 6.22 | Gbar_D03_673341 | C/A | 0.08 | 6.20 |
| Gbar_D03_556131 | C/T | 0.08 | 6.14 | Gbar_D03_619731 | C/G | 0.08 | 6.10 | Gbar_D03_675228 | T/A | 0.08 | 6.46 |
| Gbar_D03_556309 | T/C | 0.08 | 6.30 | Gbar_D03_620292 | T/C | 0.08 | 6.96 | Gbar_D03_680715 | G/A | 0.07 | 6.05 |
| Gbar_D03_556375 | T/C | 0.08 | 6.58 | Gbar_D03_620407 | T/C | 0.08 | 6.04 | Gbar_D03_680754 | A/C | 0.08 | 6.63 |
| Gbar_D03_556612 | A/G | 0.08 | 6.12 | Gbar_D03_620424 | A/C | 0.08 | 6.15 | Gbar_D03_680769 | T/C | 0.08 | 6.66 |
| Gbar_D03_557193 | G/T | 0.08 | 6.94 | Gbar_D03_620702 | G/C | 0.08 | 6.10 | Gbar_D03_680781 | A/T | 0.08 | 6.23 |
| Gbar_D03_557269 | A/G | 0.07 | 6.59 | Gbar_D03_621175 | G/T | 0.07 | 7.16 | Gbar_D03_682804 | T/C | 0.08 | 7.02 |
| Gbar_D03_558287 | C/T | 0.08 | 6.21 | Gbar_D03_621182 | A/T | 0.07 | 7.07 | Gbar_D03_682989 | C/A | 0.08 | 6.68 |
| Gbar_D03_560351 | G/C | 0.08 | 6.34 | Gbar_D03_621353 | T/G | 0.08 | 6.27 | Gbar_D03_683619 | C/T | 0.08 | 6.72 |
| Gbar_D03_561004 | T/A | 0.08 | 6.39 | Gbar_D03_621810 | G/A | 0.08 | 7.28 | Gbar_D03_684662 | C/A | 0.08 | 6.13 |
| Gbar_D03_561330 | C/T | 0.08 | 6.34 | Gbar_D03_621871 | A/T | 0.08 | 7.09 | Gbar_D03_684963 | C/T | 0.08 | 6.42 |
| Gbar_D03_566189 | T/A | 0.07 | 6.89 | Gbar_D03_621993 | C/T | 0.08 | 6.12 | Gbar_D03_685055 | G/A | 0.08 | 6.43 |
| Gbar_D03_566277 | A/G | 0.07 | 6.47 | Gbar_D03_622680 | A/G | 0.08 | 6.50 | Gbar_D03_685350 | G/A | 0.08 | 6.28 |
| Gbar_D03_567217 | G/A | 0.08 | 6.06 | Gbar_D03_622721 | G/A | 0.08 | 6.17 | Gbar_D03_685401 | G/A | 0.08 | 6.14 |
| Gbar_D03_568757 | T/C | 0.08 | 6.19 | Gbar_D03_622755 | C/T | 0.08 | 6.80 | Gbar_D03_686876 | C/T | 0.07 | 6.08 |
| Gbar_D03_568803 | C/T | 0.08 | 6.18 | Gbar_D03_622781 | C/G | 0.08 | 6.31 | Gbar_D03_687839 | T/C | 0.08 | 6.52 |
| Gbar_D03_569310 | C/T | 0.08 | 6.40 | Gbar_D03_624152 | G/A | 0.08 | 6.72 | Gbar_D03_687857 | G/A | 0.08 | 6.69 |
| Gbar_D03_570360 | C/T | 0.08 | 6.32 | Gbar_D03_624216 | G/C | 0.08 | 6.18 | Gbar_D03_687926 | A/T | 0.08 | 6.47 |
| Gbar_D03_571081 | A/C | 0.08 | 6.45 | Gbar_D03_624387 | A/G | 0.08 | 6.32 | Gbar_D03_688264 | G/A | 0.08 | 6.80 |
| Gbar_D03_571117 | G/A | 0.07 | 6.13 | Gbar_D03_625037 | C/G | 0.08 | 6.54 | Gbar_D03_688289 | A/G | 0.08 | 6.50 |
| Gbar_D03_574319 | T/C | 0.08 | 6.16 | Gbar_D03_625152 | C/T | 0.08 | 6.40 | Gbar_D03_688440 | T/A | 0.08 | 6.64 |
| Gbar_D03_574485 | T/A | 0.08 | 6.59 | Gbar_D03_626683 | A/G | 0.07 | 6.60 | Gbar_D03_688829 | G/A | 0.08 | 6.29 |
| Gbar_D03_574526 | A/G | 0.08 | 6.34 | Gbar_D03_626688 | G/C | 0.07 | 6.41 | Gbar_D03_688914 | T/A | 0.08 | 6.54 |
| Gbar_D03_576234 | G/A | 0.08 | 6.13 | Gbar_D03_627517 | C/T | 0.08 | 6.69 | Gbar_D03_689331 | G/A | 0.08 | 6.14 |
| Gbar_D03_576262 | C/T | 0.08 | 6.19 | Gbar_D03_628525 | C/T | 0.08 | 6.40 | Gbar_D03_689737 | G/C | 0.08 | 6.15 |
| Gbar_D03_577309 | A/G | 0.08 | 6.25 | Gbar_D03_629115 | G/C | 0.08 | 6.54 | Gbar_D03_689840 | T/A | 0.08 | 6.63 |
| Gbar_D03_580269 | A/G | 0.08 | 6.45 | Gbar_D03_630237 | T/C | 0.08 | 7.08 | Gbar_D03_690139 | C/T | 0.08 | 6.76 |
| Gbar_D03_580781 | G/A | 0.07 | 6.02 | Gbar_D03_630241 | C/T | 0.08 | 7.05 | Gbar_D03_690265 | T/C | 0.08 | 7.01 |
| Gbar_D03_582226 | T/C | 0.08 | 6.37 | Gbar_D03_630358 | A/G | 0.08 | 6.24 | Gbar_D03_690328 | T/G | 0.08 | 6.82 |
| Gbar_D03_582513 | A/T | 0.08 | 6.50 | Gbar_D03_630448 | A/C | 0.08 | 7.62 | Gbar_D03_690397 | T/C | 0.08 | 6.61 |
| Gbar_D03_583841 | A/G | 0.08 | 6.06 | Gbar_D03_630508 | A/G | 0.08 | 6.50 | Gbar_D03_690907 | T/C | 0.08 | 6.24 |
| Gbar_D03_586239 | A/G | 0.08 | 6.17 | Gbar_D03_631132 | A/T | 0.08 | 6.44 | Gbar_D03_691097 | T/C | 0.08 | 6.89 |
| Gbar_D03_587662 | G/A | 0.08 | 6.14 | Gbar_D03_632343 | T/C | 0.08 | 6.38 | Gbar_D03_691206 | G/A | 0.08 | 6.76 |
| Gbar_D03_587907 | T/A | 0.07 | 6.09 | Gbar_D03_632717 | C/T | 0.08 | 6.90 | Gbar_D03_691386 | T/C | 0.08 | 6.08 |
| Gbar_D03_587910 | C/T | 0.07 | 6.29 | Gbar_D03_632788 | C/T | 0.08 | 6.78 | Gbar_D03_691411 | C/T | 0.08 | 6.68 |
| Gbar_D03_588355 | G/A | 0.07 | 6.31 | Gbar_D03_633338 | C/G | 0.08 | 6.70 | Gbar_D03_691670 | A/G | 0.08 | 6.84 |
| Gbar_D03_588368 | T/A | 0.08 | 6.25 | Gbar_D03_635739 | G/A | 0.08 | 6.48 | Gbar_D03_691695 | T/A | 0.08 | 6.88 |
| Gbar_D03_588540 | G/A | 0.08 | 6.11 | Gbar_D03_636387 | T/C | 0.08 | 7.02 | Gbar_D03_691815 | G/A | 0.08 | 6.61 |
| Gbar_D03_588734 | A/G | 0.08 | 6.27 | Gbar_D03_638422 | A/T | 0.08 | 6.97 | Gbar_D03_691924 | C/G | 0.08 | 6.99 |
| Gbar_D03_589494 | T/C | 0.08 | 6.30 | Gbar_D03_638844 | G/C | 0.08 | 6.40 | Gbar_D03_692297 | C/T | 0.08 | 6.86 |
| Gbar_D03_589606 | C/T | 0.08 | 6.09 | Gbar_D03_639346 | A/C | 0.08 | 6.60 | Gbar_D03_692350 | A/C | 0.08 | 6.80 |
| Gbar_D03_589777 | G/A | 0.08 | 6.55 | Gbar_D03_640131 | T/A | 0.08 | 6.25 | Gbar_D03_693585 | A/G | 0.08 | 6.73 |
| Gbar_D03_591495 | G/A | 0.08 | 6.14 | Gbar_D03_640851 | C/T | 0.08 | 6.49 | Gbar_D03_693592 | T/C | 0.08 | 6.61 |
| Gbar_D03_591529 | C/T | 0.08 | 6.46 | Gbar_D03_641404 | T/A | 0.08 | 6.85 | Gbar_D03_693634 | A/G | 0.08 | 6.50 |
| Gbar_D03_591662 | G/C | 0.08 | 6.03 | Gbar_D03_641609 | A/G | 0.08 | 6.88 | Gbar_D03_693671 | T/C | 0.08 | 6.63 |
| Gbar_D03_591765 | A/G | 0.08 | 6.09 | Gbar_D03_641827 | T/C | 0.08 | 6.67 | Gbar_D03_693705 | G/A | 0.08 | 6.48 |
| Gbar_D03_593143 | A/T | 0.08 | 6.35 | Gbar_D03_641868 | A/C | 0.08 | 6.39 | Gbar_D03_694384 | T/C | 0.08 | 6.96 |
| Gbar_D03_593281 | A/T | 0.08 | 6.05 | Gbar_D03_642084 | G/A | 0.08 | 6.52 | Gbar_D03_694393 | C/T | 0.08 | 6.17 |
| Gbar_D03_594365 | T/G | 0.08 | 6.43 | Gbar_D03_642090 | A/G | 0.08 | 6.58 | Gbar_D03_694535 | T/C | 0.08 | 6.09 |
| Gbar_D03_594571 | G/A | 0.08 | 6.83 | Gbar_D03_643791 | A/T | 0.08 | 6.53 | Gbar_D03_695003 | A/G | 0.08 | 6.43 |
| Gbar_D03_594606 | G/A | 0.08 | 7.03 | Gbar_D03_643823 | G/C | 0.07 | 6.56 | Gbar_D03_695028 | G/T | 0.09 | 6.85 |
| Gbar_D03_595001 | T/C | 0.08 | 6.59 | Gbar_D03_643845 | A/G | 0.07 | 6.73 | Gbar_D03_697224 | G/C | 0.08 | 6.75 |
| Gbar_D03_595385 | A/G | 0.08 | 6.13 | Gbar_D03_644194 | A/G | 0.08 | 6.49 | Gbar_D03_697559 | A/G | 0.08 | 6.14 |
| Gbar_D03_595520 | T/G | 0.08 | 6.70 | Gbar_D03_644468 | A/T | 0.08 | 6.91 | Gbar_D03_697734 | G/T | 0.08 | 6.65 |
| Gbar_D03_595940 | G/T | 0.08 | 6.48 | Gbar_D03_644961 | G/A | 0.08 | 6.44 | Gbar_D03_699771 | A/G | 0.08 | 6.99 |
| Gbar_D03_596036 | G/A | 0.08 | 6.68 | Gbar_D03_647296 | C/A | 0.08 | 6.01 | Gbar_D03_700031 | A/G | 0.08 | 6.08 |
| Gbar_D03_597218 | T/C | 0.08 | 6.01 | Gbar_D03_648421 | T/C | 0.08 | 6.06 | Gbar_D03_700713 | A/C | 0.08 | 6.61 |
| Gbar_D03_597762 | T/C | 0.08 | 6.28 | Gbar_D03_648646 | A/T | 0.08 | 6.29 | Gbar_D03_700896 | T/C | 0.07 | 6.14 |
| Gbar_D03_598106 | G/A | 0.08 | 6.26 | Gbar_D03_648950 | G/A | 0.08 | 6.52 | Gbar_D03_701142 | A/T | 0.08 | 6.76 |
| Gbar_D03_598463 | T/C | 0.08 | 6.12 | Gbar_D03_650186 | G/T | 0.08 | 6.88 | Gbar_D03_702165 | T/G | 0.08 | 6.32 |
| Gbar_D03_598794 | G/A | 0.08 | 6.61 | Gbar_D03_651982 | C/T | 0.08 | 6.13 | Gbar_D03_703498 | C/T | 0.08 | 7.30 |
| Gbar_D03_599878 | A/C | 0.08 | 6.30 | Gbar_D03_652175 | T/C | 0.08 | 6.32 | Gbar_D03_704291 | T/A | 0.07 | 6.17 |
| Gbar_D03_600088 | T/C | 0.08 | 6.19 | Gbar_D03_652236 | C/T | 0.08 | 6.33 | Gbar_D03_706397 | G/T | 0.08 | 6.52 |
| Gbar_D03_600309 | A/T | 0.08 | 6.16 | Gbar_D03_653249 | C/T | 0.07 | 6.16 | Gbar_D03_706853 | T/C | 0.08 | 6.27 |
| Gbar_D03_600372 | G/A | 0.08 | 6.78 | Gbar_D03_653369 | T/C | 0.08 | 6.98 | Gbar_D03_706901 | A/G | 0.08 | 6.71 |
| Gbar_D03_601270 | G/A | 0.08 | 7.19 | Gbar_D03_653399 | C/A | 0.08 | 6.55 | Gbar_D03_707326 | T/C | 0.08 | 6.94 |
| Gbar_D03_601371 | C/G | 0.08 | 6.24 | Gbar_D03_653541 | G/T | 0.09 | 7.05 | Gbar_D03_708318 | A/C | 0.08 | 6.66 |
| Gbar_D03_601400 | G/A | 0.08 | 6.65 | Gbar_D03_653844 | C/T | 0.08 | 7.34 | Gbar_D03_709055 | T/C | 0.08 | 6.25 |
| Gbar_D03_602149 | G/T | 0.08 | 6.07 | Gbar_D03_653863 | G/A | 0.08 | 6.86 | Gbar_D03_709387 | T/C | 0.08 | 6.34 |
| Gbar_D03_602267 | C/A | 0.08 | 6.31 | Gbar_D03_653900 | A/T | 0.08 | 7.52 | Gbar_D03_709747 | A/T | 0.08 | 6.70 |
| Gbar_D03_603037 | T/C | 0.08 | 6.31 | Gbar_D03_656976 | A/G | 0.08 | 6.78 | Gbar_D03_709909 | C/G | 0.08 | 6.69 |
| Gbar_D03_603254 | G/A | 0.08 | 6.71 | Gbar_D03_656992 | T/C | 0.08 | 6.70 | Gbar_D03_711023 | G/A | 0.08 | 6.08 |
| Gbar_D03_603337 | T/G | 0.08 | 7.22 | Gbar_D03_657002 | A/G | 0.08 | 6.46 | Gbar_D03_711031 | C/T | 0.08 | 6.09 |
| Gbar_D03_604017 | C/T | 0.08 | 6.12 | Gbar_D03_658071 | T/A | 0.08 | 6.46 | Gbar_D03_711056 | A/G | 0.08 | 6.29 |
| Gbar_D03_604273 | G/A | 0.08 | 6.65 | Gbar_D03_658777 | T/C | 0.08 | 7.00 | Gbar_D03_711322 | C/T | 0.08 | 6.19 |
| Gbar_D03_604678 | A/G | 0.08 | 6.63 | Gbar_D03_660586 | A/G | 0.08 | 6.89 | Gbar_D03_711548 | C/T | 0.08 | 6.19 |
| Gbar_D03_605420 | A/C | 0.08 | 6.74 | Gbar_D03_660707 | T/C | 0.08 | 6.53 | Gbar_D03_711741 | T/C | 0.08 | 6.38 |
| Gbar_D03_606155 | A/G | 0.08 | 6.97 | Gbar_D03_661386 | C/A | 0.08 | 6.29 | Gbar_D03_711951 | G/A | 0.08 | 6.16 |
| Gbar_D03_606191 | T/C | 0.08 | 6.09 | Gbar_D03_662294 | A/G | 0.08 | 7.33 | Gbar_D03_711956 | C/T | 0.08 | 6.05 |
| Gbar_D03_606328 | G/C | 0.08 | 6.73 | Gbar_D03_662391 | C/A | 0.08 | 6.24 | Gbar_D03_711964 | T/C | 0.08 | 6.05 |
| Gbar_D03_606418 | C/T | 0.08 | 6.09 | Gbar_D03_662519 | G/A | 0.08 | 6.67 | Gbar_D03_712396 | T/C | 0.08 | 6.05 |
| Gbar_D03_607668 | A/G | 0.08 | 6.74 | Gbar_D03_662524 | C/T | 0.08 | 6.46 | Gbar_D03_712797 | G/A | 0.07 | 6.25 |
| Gbar_D03_607819 | G/T | 0.08 | 6.32 | Gbar_D03_662685 | C/T | 0.08 | 6.85 | Gbar_D03_713258 | C/T | 0.08 | 6.11 |
| Gbar_D03_608224 | A/G | 0.08 | 6.95 | Gbar_D03_662796 | G/A | 0.08 | 6.68 | Gbar_D03_713484 | A/T | 0.08 | 6.94 |
| Gbar_D03_609723 | G/A | 0.08 | 6.10 | Gbar_D03_662920 | A/T | 0.07 | 6.07 | Gbar_D03_713500 | T/C | 0.08 | 6.78 |
| Gbar_D03_609844 | A/G | 0.08 | 6.35 | Gbar_D03_662986 | T/A | 0.08 | 6.87 | Gbar_D03_714281 | A/C | 0.08 | 7.00 |
| Gbar_D03_609904 | C/T | 0.08 | 6.42 | Gbar_D03_663730 | T/C | 0.08 | 6.93 | Gbar_D03_715304 | A/G | 0.08 | 7.57 |
| Gbar_D03_610091 | A/T | 0.08 | 6.36 | Gbar_D03_664095 | G/C | 0.08 | 6.93 | Gbar_D03_715379 | T/A | 0.08 | 6.74 |
| Gbar_D03_611407 | A/T | 0.08 | 6.16 | Gbar_D03_664883 | T/G | 0.08 | 6.33 | Gbar_D03_715785 | C/T | 0.08 | 6.27 |
| Gbar_D03_611581 | C/A | 0.08 | 6.18 | Gbar_D03_665422 | G/A | 0.08 | 6.84 | Gbar_D03_715832 | A/G | 0.08 | 6.45 |
| Gbar_D03_612422 | A/G | 0.08 | 6.09 | Gbar_D03_665945 | C/T | 0.08 | 6.55 | Gbar_D03_716243 | T/C | 0.08 | 6.81 |
| Gbar_D03_612444 | T/C | 0.08 | 6.17 | Gbar_D03_666060 | T/C | 0.07 | 6.06 | Gbar_D03_716250 | G/A | 0.08 | 7.01 |
| Gbar_D03_612619 | A/G | 0.08 | 6.08 | Gbar_D03_666072 | C/T | 0.07 | 6.34 | Gbar_D03_716447 | A/G | 0.08 | 6.59 |
| Gbar_D03_612946 | A/T | 0.08 | 7.05 | Gbar_D03_666219 | T/C | 0.08 | 6.52 | Gbar_D03_716766 | A/G | 0.08 | 6.71 |
| Gbar_D03_613046 | A/G | 0.08 | 6.75 | Gbar_D03_666320 | C/T | 0.08 | 6.89 | Gbar_D03_717015 | A/G | 0.08 | 6.14 |

**Table S2.** List of the associated SNPs identified in different datasets through GWAS for *FW* resistance in Sea Island cotton. (Continued)

| SNP | Ref/Alt | MAF | -log (p-value) | SNP | Ref/Alt | MAF | -log (p-value) | SNP | Ref/Alt | MAF | -log (p-value) |
| --- | --- | --- | --- | --- | --- | --- | --- | --- | --- | --- | --- |
| **2015** |  |  |  | Gbar_D03_766362 | C/T | 0.08 | 6.92 | Gbar_D03_799134 | A/G | 0.08 | 6.63 |
| Gbar_D03_717407 | T/C | 0.08 | 7.19 | Gbar_D03_766470 | G/C | 0.08 | 6.95 | Gbar_D03_800106 | C/T | 0.08 | 7.09 |
| Gbar_D03_717695 | G/A | 0.08 | 6.12 | Gbar_D03_766476 | G/C | 0.08 | 6.49 | Gbar_D03_800241 | A/G | 0.08 | 6.69 |
| Gbar_D03_717742 | G/A | 0.08 | 6.40 | Gbar_D03_766747 | T/A | 0.08 | 6.42 | Gbar_D03_800458 | C/T | 0.08 | 6.87 |
| Gbar_D03_717779 | T/C | 0.09 | 7.42 | Gbar_D03_767268 | A/G | 0.08 | 7.64 | Gbar_D03_802124 | G/A | 0.08 | 6.64 |
| Gbar_D03_717803 | C/G | 0.09 | 6.60 | Gbar_D03_767400 | A/G | 0.08 | 6.57 | Gbar_D03_802954 | C/A | 0.08 | 6.85 |
| Gbar_D03_717823 | C/A | 0.08 | 7.37 | Gbar_D03_768013 | T/C | 0.08 | 6.78 | Gbar_D03_802966 | C/T | 0.08 | 6.64 |
| Gbar_D03_718086 | T/C | 0.08 | 6.27 | Gbar_D03_768014 | C/T | 0.08 | 6.83 | Gbar_D03_803002 | A/T | 0.08 | 6.22 |
| Gbar_D03_718221 | C/A | 0.08 | 6.99 | Gbar_D03_768062 | C/T | 0.08 | 6.19 | Gbar_D03_803219 | C/T | 0.08 | 6.90 |
| Gbar_D03_718281 | A/G | 0.08 | 7.80 | Gbar_D03_768071 | A/G | 0.08 | 6.82 | Gbar_D03_803493 | C/T | 0.08 | 6.44 |
| Gbar_D03_718293 | T/C | 0.08 | 7.64 | Gbar_D03_768195 | G/C | 0.08 | 6.23 | Gbar_D03_803800 | C/G | 0.08 | 6.64 |
| Gbar_D03_718418 | C/T | 0.08 | 6.05 | Gbar_D03_768199 | G/T | 0.08 | 6.23 | Gbar_D03_804931 | A/C | 0.08 | 6.34 |
| Gbar_D03_718489 | C/T | 0.08 | 6.39 | Gbar_D03_769125 | C/A | 0.09 | 6.79 | Gbar_D03_806228 | G/T | 0.08 | 6.73 |
| Gbar_D03_719951 | G/C | 0.08 | 6.73 | Gbar_D03_769406 | A/T | 0.08 | 6.92 | Gbar_D03_806237 | A/G | 0.08 | 6.75 |
| Gbar_D03_721035 | T/C | 0.08 | 6.83 | Gbar_D03_769597 | A/G | 0.08 | 6.38 | Gbar_D03_806328 | G/C | 0.08 | 6.33 |
| Gbar_D03_721848 | T/A | 0.08 | 6.45 | Gbar_D03_769704 | A/G | 0.08 | 7.08 | Gbar_D03_806361 | A/G | 0.08 | 6.16 |
| Gbar_D03_722427 | A/G | 0.07 | 6.08 | Gbar_D03_770296 | T/C | 0.08 | 6.70 | Gbar_D03_806529 | A/G | 0.08 | 6.52 |
| Gbar_D03_722630 | C/T | 0.08 | 6.42 | Gbar_D03_770656 | G/A | 0.08 | 6.90 | Gbar_D03_807754 | T/A | 0.08 | 7.08 |
| Gbar_D03_722745 | T/C | 0.08 | 6.85 | Gbar_D03_771239 | T/C | 0.08 | 6.50 | Gbar_D03_807990 | C/T | 0.08 | 7.08 |
| Gbar_D03_723816 | G/A | 0.08 | 6.87 | Gbar_D03_771322 | T/C | 0.08 | 6.39 | Gbar_D03_808150 | A/C | 0.08 | 6.31 |
| Gbar_D03_723821 | C/T | 0.08 | 6.68 | Gbar_D03_771391 | A/G | 0.08 | 6.29 | Gbar_D03_808265 | C/G | 0.08 | 6.00 |
| Gbar_D03_728310 | T/A | 0.08 | 6.39 | Gbar_D03_771395 | T/C | 0.08 | 6.68 | Gbar_D03_808550 | T/G | 0.08 | 7.15 |
| Gbar_D03_728441 | A/C | 0.08 | 6.22 | Gbar_D03_771406 | A/G | 0.08 | 6.54 | Gbar_D03_809610 | C/T | 0.08 | 6.73 |
| Gbar_D03_728634 | T/A | 0.08 | 6.65 | Gbar_D03_771825 | G/A | 0.08 | 6.48 | Gbar_D03_809907 | T/A | 0.08 | 7.61 |
| Gbar_D03_729685 | C/A | 0.08 | 7.26 | Gbar_D03_772090 | G/A | 0.08 | 6.50 | Gbar_D03_809936 | T/C | 0.08 | 7.29 |
| Gbar_D03_729694 | T/G | 0.08 | 7.27 | Gbar_D03_772091 | G/A | 0.08 | 6.85 | Gbar_D03_810116 | C/T | 0.08 | 6.30 |
| Gbar_D03_730450 | T/C | 0.08 | 7.14 | Gbar_D03_772929 | A/G | 0.08 | 6.37 | Gbar_D03_810150 | G/A | 0.08 | 6.59 |
| Gbar_D03_730643 | G/A | 0.08 | 6.54 | Gbar_D03_773717 | G/A | 0.08 | 6.70 | Gbar_D03_810613 | A/G | 0.08 | 7.41 |
| Gbar_D03_731173 | C/T | 0.08 | 6.15 | Gbar_D03_774109 | G/A | 0.08 | 6.26 | Gbar_D03_810680 | G/A | 0.07 | 6.26 |
| Gbar_D03_731174 | A/G | 0.08 | 6.21 | Gbar_D03_774292 | C/G | 0.08 | 6.09 | Gbar_D03_811064 | A/T | 0.08 | 6.32 |
| Gbar_D03_731506 | A/G | 0.08 | 6.80 | Gbar_D03_774400 | C/T | 0.08 | 6.66 | Gbar_D03_811336 | T/C | 0.08 | 6.78 |
| Gbar_D03_731523 | C/T | 0.08 | 6.64 | Gbar_D03_774415 | C/T | 0.08 | 7.44 | Gbar_D03_811591 | T/C | 0.08 | 6.55 |
| Gbar_D03_731669 | C/A | 0.08 | 6.64 | Gbar_D03_774439 | T/C | 0.08 | 7.08 | Gbar_D03_811659 | C/T | 0.08 | 6.31 |
| Gbar_D03_732313 | A/G | 0.08 | 6.80 | Gbar_D03_774458 | C/T | 0.08 | 7.04 | Gbar_D03_811778 | A/C | 0.08 | 6.91 |
| Gbar_D03_732565 | G/A | 0.08 | 6.61 | Gbar_D03_774516 | A/C | 0.08 | 6.64 | Gbar_D03_812251 | C/G | 0.08 | 7.15 |
| Gbar_D03_733818 | T/C | 0.08 | 6.69 | Gbar_D03_774523 | A/T | 0.08 | 6.50 | Gbar_D03_813482 | C/T | 0.08 | 6.98 |
| Gbar_D03_733937 | C/T | 0.08 | 6.88 | Gbar_D03_775070 | C/T | 0.08 | 6.61 | Gbar_D03_813572 | T/G | 0.08 | 6.54 |
| Gbar_D03_733973 | A/G | 0.08 | 7.61 | Gbar_D03_775094 | T/A | 0.08 | 6.66 | Gbar_D03_813580 | A/C | 0.07 | 6.37 |
| Gbar_D03_734227 | T/C | 0.08 | 6.70 | Gbar_D03_775183 | A/C | 0.08 | 6.73 | Gbar_D03_813730 | T/A | 0.08 | 6.82 |
| Gbar_D03_736371 | G/T | 0.08 | 6.65 | Gbar_D03_775461 | T/G | 0.08 | 6.66 | Gbar_D03_813783 | T/C | 0.08 | 6.49 |
| Gbar_D03_737104 | T/A | 0.08 | 7.37 | Gbar_D03_775562 | A/G | 0.08 | 6.94 | Gbar_D03_815481 | A/G | 0.08 | 6.29 |
| Gbar_D03_737229 | G/A | 0.08 | 6.94 | Gbar_D03_775614 | G/A | 0.08 | 6.78 | Gbar_D03_815732 | C/T | 0.08 | 6.43 |
| Gbar_D03_737284 | T/C | 0.07 | 6.32 | Gbar_D03_776147 | G/A | 0.08 | 6.08 | Gbar_D03_816340 | G/A | 0.08 | 7.02 |
| Gbar_D03_737374 | G/A | 0.08 | 6.57 | Gbar_D03_776247 | A/G | 0.08 | 6.38 | Gbar_D03_817303 | G/A | 0.09 | 6.70 |
| Gbar_D03_737402 | A/G | 0.08 | 6.43 | Gbar_D03_776271 | C/A | 0.08 | 6.36 | Gbar_D03_817476 | G/A | 0.08 | 6.17 |
| Gbar_D03_738020 | G/A | 0.08 | 6.40 | Gbar_D03_776580 | G/C | 0.08 | 6.44 | Gbar_D03_818461 | T/C | 0.08 | 6.67 |
| Gbar_D03_738692 | G/T | 0.08 | 6.56 | Gbar_D03_776590 | A/G | 0.08 | 6.31 | Gbar_D03_818955 | C/A | 0.08 | 6.07 |
| Gbar_D03_738725 | C/T | 0.08 | 6.72 | Gbar_D03_776611 | G/C | 0.08 | 6.47 | Gbar_D03_819316 | C/G | 0.08 | 6.91 |
| Gbar_D03_739176 | T/C | 0.08 | 6.57 | Gbar_D03_776798 | T/C | 0.08 | 6.47 | Gbar_D03_819566 | C/T | 0.08 | 6.33 |
| Gbar_D03_740008 | G/C | 0.08 | 6.80 | Gbar_D03_776847 | C/G | 0.08 | 6.48 | Gbar_D03_820090 | A/C | 0.07 | 6.17 |
| Gbar_D03_740811 | A/G | 0.08 | 6.80 | Gbar_D03_777093 | C/T | 0.08 | 7.10 | Gbar_D03_821496 | T/C | 0.08 | 6.65 |
| Gbar_D03_741159 | C/G | 0.08 | 6.98 | Gbar_D03_777111 | T/G | 0.08 | 6.72 | Gbar_D03_821544 | C/T | 0.08 | 6.49 |
| Gbar_D03_743815 | G/A | 0.08 | 6.77 | Gbar_D03_777135 | T/G | 0.08 | 7.24 | Gbar_D03_822027 | A/T | 0.08 | 7.10 |
| Gbar_D03_743864 | T/C | 0.08 | 6.53 | Gbar_D03_777146 | A/G | 0.08 | 6.84 | Gbar_D03_823477 | G/T | 0.08 | 6.86 |
| Gbar_D03_743905 | T/C | 0.08 | 6.79 | Gbar_D03_777215 | T/A | 0.08 | 7.22 | Gbar_D03_825160 | G/T | 0.08 | 6.91 |
| Gbar_D03_744095 | A/T | 0.08 | 6.50 | Gbar_D03_777669 | C/T | 0.08 | 6.42 | Gbar_D03_825670 | A/G | 0.08 | 6.64 |
| Gbar_D03_744220 | T/G | 0.08 | 6.75 | Gbar_D03_777938 | T/G | 0.08 | 6.30 | Gbar_D03_826341 | T/C | 0.07 | 6.37 |
| Gbar_D03_744859 | G/C | 0.08 | 6.32 | Gbar_D03_778036 | T/C | 0.08 | 6.04 | Gbar_D03_826386 | A/T | 0.07 | 6.35 |
| Gbar_D03_746844 | A/C | 0.08 | 7.12 | Gbar_D03_778610 | G/A | 0.08 | 6.05 | Gbar_D03_826631 | G/A | 0.08 | 7.92 |
| Gbar_D03_746977 | C/G | 0.08 | 7.36 | Gbar_D03_779389 | T/C | 0.08 | 6.75 | Gbar_D03_826777 | C/T | 0.08 | 7.14 |
| Gbar_D03_747092 | A/T | 0.08 | 6.22 | Gbar_D03_779918 | C/T | 0.08 | 6.79 | Gbar_D03_829019 | A/G | 0.08 | 6.60 |
| Gbar_D03_749121 | A/T | 0.08 | 6.74 | Gbar_D03_780704 | A/G | 0.08 | 7.79 | Gbar_D03_829425 | A/T | 0.08 | 6.58 |
| Gbar_D03_749122 | T/A | 0.08 | 6.65 | Gbar_D03_781370 | C/T | 0.08 | 6.35 | Gbar_D03_831772 | A/G | 0.07 | 6.09 |
| Gbar_D03_749369 | C/T | 0.08 | 6.47 | Gbar_D03_786604 | A/T | 0.08 | 6.92 | Gbar_D03_831944 | T/A | 0.08 | 6.80 |
| Gbar_D03_749685 | G/A | 0.08 | 6.45 | Gbar_D03_789050 | C/T | 0.08 | 6.61 | Gbar_D03_832044 | G/A | 0.08 | 6.97 |
| Gbar_D03_749891 | A/T | 0.08 | 6.21 | Gbar_D03_789434 | A/G | 0.08 | 6.59 | Gbar_D03_832128 | T/C | 0.08 | 6.54 |
| Gbar_D03_750408 | C/T | 0.08 | 6.72 | Gbar_D03_789555 | G/A | 0.08 | 6.26 | Gbar_D03_832209 | G/A | 0.08 | 6.81 |
| Gbar_D03_750411 | G/A | 0.08 | 6.81 | Gbar_D03_789594 | A/C | 0.08 | 6.36 | Gbar_D03_832789 | C/G | 0.08 | 7.02 |
| Gbar_D03_750737 | C/T | 0.08 | 6.90 | Gbar_D03_790890 | G/C | 0.08 | 6.55 | Gbar_D03_833775 | T/C | 0.07 | 6.07 |
| Gbar_D03_750908 | G/T | 0.08 | 6.76 | Gbar_D03_791028 | T/G | 0.08 | 6.34 | Gbar_D03_833918 | T/C | 0.08 | 6.46 |
| Gbar_D03_750962 | G/C | 0.08 | 7.23 | Gbar_D03_791052 | T/C | 0.08 | 6.46 | Gbar_D03_834213 | G/A | 0.08 | 6.30 |
| Gbar_D03_751530 | A/G | 0.09 | 6.24 | Gbar_D03_791742 | T/C | 0.08 | 6.81 | Gbar_D03_834357 | G/A | 0.08 | 6.93 |
| Gbar_D03_751937 | C/T | 0.07 | 6.15 | Gbar_D03_791768 | T/A | 0.08 | 6.56 | Gbar_D03_834385 | G/A | 0.08 | 6.35 |
| Gbar_D03_752091 | T/A | 0.08 | 7.07 | Gbar_D03_792002 | A/G | 0.08 | 6.50 | Gbar_D03_834806 | T/C | 0.08 | 7.22 |
| Gbar_D03_752271 | T/C | 0.08 | 6.37 | Gbar_D03_792243 | G/A | 0.08 | 6.14 | Gbar_D03_835978 | A/G | 0.08 | 6.77 |
| Gbar_D03_753046 | C/G | 0.08 | 6.89 | Gbar_D03_792244 | T/C | 0.08 | 6.44 | Gbar_D03_836246 | T/A | 0.08 | 6.75 |
| Gbar_D03_753863 | A/G | 0.08 | 6.23 | Gbar_D03_792267 | A/G | 0.08 | 6.51 | Gbar_D03_836250 | A/G | 0.08 | 6.78 |
| Gbar_D03_754158 | C/T | 0.08 | 7.42 | Gbar_D03_792353 | A/T | 0.08 | 6.12 | Gbar_D03_837245 | G/A | 0.08 | 6.64 |
| Gbar_D03_754171 | A/G | 0.08 | 6.43 | Gbar_D03_792622 | C/T | 0.08 | 6.82 | Gbar_D03_837933 | T/A | 0.08 | 6.48 |
| Gbar_D03_754191 | A/T | 0.08 | 7.31 | Gbar_D03_792702 | T/C | 0.08 | 6.98 | Gbar_D03_837993 | A/C | 0.08 | 6.67 |
| Gbar_D03_754844 | A/G | 0.08 | 6.46 | Gbar_D03_792847 | C/T | 0.08 | 6.35 | Gbar_D03_838032 | G/C | 0.08 | 6.38 |
| Gbar_D03_755431 | T/C | 0.08 | 6.55 | Gbar_D03_793315 | C/T | 0.08 | 6.75 | Gbar_D03_839089 | A/G | 0.08 | 6.71 |
| Gbar_D03_755820 | T/C | 0.08 | 6.75 | Gbar_D03_793333 | C/T | 0.08 | 7.09 | Gbar_D03_839863 | T/C | 0.08 | 6.61 |
| Gbar_D03_756699 | C/A | 0.08 | 6.11 | Gbar_D03_793566 | G/A | 0.08 | 6.89 | Gbar_D03_839876 | T/C | 0.08 | 6.79 |
| Gbar_D03_756879 | A/G | 0.08 | 6.49 | Gbar_D03_793819 | G/A | 0.08 | 6.10 | Gbar_D03_840765 | T/C | 0.08 | 6.24 |
| Gbar_D03_757401 | T/C | 0.08 | 6.30 | Gbar_D03_794186 | T/A | 0.08 | 6.35 | Gbar_D03_841312 | C/G | 0.08 | 6.78 |
| Gbar_D03_757690 | G/A | 0.09 | 6.47 | Gbar_D03_794227 | T/G | 0.08 | 6.66 | Gbar_D03_841994 | A/C | 0.08 | 6.50 |
| Gbar_D03_757878 | C/T | 0.08 | 6.92 | Gbar_D03_794306 | G/A | 0.08 | 6.53 | Gbar_D03_842586 | A/G | 0.08 | 6.63 |
| Gbar_D03_757949 | T/A | 0.08 | 6.75 | Gbar_D03_794426 | G/T | 0.08 | 6.29 | Gbar_D03_843572 | A/C | 0.08 | 6.14 |
| Gbar_D03_757977 | T/A | 0.08 | 6.76 | Gbar_D03_794437 | C/A | 0.08 | 6.55 | Gbar_D03_843648 | A/T | 0.08 | 6.39 |
| Gbar_D03_757988 | A/C | 0.08 | 6.53 | Gbar_D03_794472 | C/T | 0.08 | 6.51 | Gbar_D03_843867 | C/T | 0.08 | 6.78 |
| Gbar_D03_758002 | A/G | 0.08 | 6.78 | Gbar_D03_794550 | G/A | 0.08 | 6.31 | Gbar_D03_845453 | A/G | 0.09 | 6.55 |
| Gbar_D03_758220 | T/A | 0.08 | 6.60 | Gbar_D03_795285 | C/T | 0.08 | 6.27 | Gbar_D03_845600 | G/A | 0.09 | 6.31 |
| Gbar_D03_758463 | T/C | 0.08 | 6.04 | Gbar_D03_795465 | T/A | 0.08 | 6.66 | Gbar_D03_845689 | A/G | 0.08 | 6.89 |
| Gbar_D03_759120 | T/C | 0.08 | 6.62 | Gbar_D03_795516 | T/A | 0.08 | 6.90 | Gbar_D03_845864 | T/G | 0.08 | 6.28 |
| Gbar_D03_760090 | T/A | 0.08 | 6.29 | Gbar_D03_795532 | G/A | 0.08 | 6.35 | Gbar_D03_845887 | G/A | 0.09 | 6.96 |
| Gbar_D03_760482 | T/G | 0.08 | 6.44 | Gbar_D03_795574 | A/G | 0.08 | 6.72 | Gbar_D03_845918 | C/T | 0.08 | 6.92 |
| Gbar_D03_760524 | T/C | 0.08 | 6.40 | Gbar_D03_795798 | G/A | 0.08 | 6.78 | Gbar_D03_845927 | C/A | 0.08 | 6.92 |
| Gbar_D03_761235 | C/A | 0.08 | 6.41 | Gbar_D03_795872 | T/G | 0.08 | 6.15 | Gbar_D03_845978 | C/T | 0.08 | 6.67 |
| Gbar_D03_761532 | T/C | 0.08 | 6.32 | Gbar_D03_796062 | G/T | 0.08 | 6.28 | Gbar_D03_846044 | C/T | 0.08 | 6.95 |
| Gbar_D03_761605 | A/G | 0.08 | 6.82 | Gbar_D03_796100 | T/C | 0.08 | 6.25 | Gbar_D03_846113 | A/G | 0.08 | 7.68 |
| Gbar_D03_761776 | C/T | 0.08 | 6.52 | Gbar_D03_796101 | A/C | 0.08 | 6.53 | Gbar_D03_846244 | C/T | 0.08 | 6.83 |
| Gbar_D03_762044 | G/T | 0.08 | 6.87 | Gbar_D03_796198 | T/C | 0.08 | 6.03 | Gbar_D03_846411 | G/A | 0.08 | 6.88 |
| Gbar_D03_762239 | C/T | 0.08 | 7.22 | Gbar_D03_796307 | A/G | 0.08 | 6.16 | Gbar_D03_846928 | T/C | 0.08 | 6.46 |
| Gbar_D03_762484 | T/C | 0.08 | 6.13 | Gbar_D03_796823 | T/C | 0.08 | 6.40 | Gbar_D03_847147 | G/A | 0.08 | 6.54 |
| Gbar_D03_762656 | G/A | 0.08 | 6.60 | Gbar_D03_796888 | T/C | 0.08 | 6.89 | Gbar_D03_847189 | G/A | 0.08 | 6.42 |
| Gbar_D03_762788 | T/A | 0.08 | 6.28 | Gbar_D03_797066 | G/T | 0.08 | 6.81 | Gbar_D03_847202 | A/T | 0.08 | 6.43 |
| Gbar_D03_764099 | A/G | 0.08 | 6.46 | Gbar_D03_797150 | A/T | 0.08 | 6.30 | Gbar_D03_847223 | A/G | 0.08 | 6.14 |
| Gbar_D03_764935 | C/T | 0.08 | 6.43 | Gbar_D03_797573 | T/A | 0.08 | 6.03 | Gbar_D03_847355 | T/G | 0.08 | 6.91 |
| Gbar_D03_765112 | T/C | 0.08 | 6.54 | Gbar_D03_798962 | C/T | 0.08 | 6.67 | Gbar_D03_847401 | G/A | 0.08 | 6.77 |

**Table S2.** List of the associated SNPs identified in different datasets through GWAS for *FW* resistance in Sea Island cotton. (Continued)

| SNP | Ref/Alt | MAF | -log (p-value) | SNP | Ref/Alt | MAF | -log (p-value) | SNP | Ref/Alt | MAF | -log (p-value) |
| --- | --- | --- | --- | --- | --- | --- | --- | --- | --- | --- | --- |
| **2015** |  |  |  | Gbar_D03_903935 | G/A | 0.08 | 6.93 | Gbar_D03_1117378 | A/G | 0.16 | 6.34 |
| Gbar_D03_847995 | G/A | 0.08 | 6.02 | Gbar_D03_904409 | G/T | 0.08 | 6.40 | Gbar_D03_1117759 | A/G | 0.16 | 6.65 |
| Gbar_D03_848275 | T/C | 0.07 | 6.20 | Gbar_D03_906027 | G/C | 0.08 | 6.76 | Gbar_D03_1118571 | T/C | 0.16 | 6.32 |
| Gbar_D03_848499 | C/T | 0.08 | 6.11 | Gbar_D03_906494 | T/C | 0.08 | 6.79 | Gbar_D03_1121167 | G/A | 0.16 | 6.33 |
| Gbar_D03_848643 | A/C | 0.08 | 7.26 | Gbar_D03_906561 | A/C | 0.08 | 6.34 | Gbar_D03_1121203 | T/C | 0.15 | 6.02 |
| Gbar_D03_848661 | C/T | 0.08 | 7.35 | Gbar_D03_906646 | A/T | 0.08 | 7.13 | Gbar_D03_1121577 | G/A | 0.16 | 6.56 |
| Gbar_D03_849352 | A/G | 0.08 | 6.26 | Gbar_D03_906765 | T/G | 0.08 | 6.89 | Gbar_D03_1126238 | T/C | 0.15 | 6.66 |
| Gbar_D03_849388 | G/A | 0.08 | 6.44 | Gbar_D03_907819 | G/T | 0.08 | 6.68 | Gbar_D03_1131867 | G/A | 0.08 | 6.50 |
| Gbar_D03_849439 | A/G | 0.08 | 6.51 | Gbar_D03_907826 | C/T | 0.08 | 6.60 | Gbar_D03_1133323 | G/A | 0.08 | 6.08 |
| Gbar_D03_849633 | C/T | 0.08 | 6.11 | Gbar_D03_907859 | G/A | 0.08 | 6.53 | Gbar_D03_1140499 | C/A | 0.08 | 6.52 |
| Gbar_D03_850056 | C/A | 0.08 | 6.60 | Gbar_D03_908044 | A/T | 0.08 | 6.49 | Gbar_D03_1140510 | T/G | 0.08 | 6.16 |
| Gbar_D03_850208 | C/T | 0.08 | 6.93 | Gbar_D03_908268 | A/G | 0.08 | 6.75 | Gbar_D03_1141035 | C/T | 0.08 | 6.86 |
| Gbar_D03_850677 | C/A | 0.08 | 6.13 | Gbar_D03_908768 | A/C | 0.08 | 6.12 | Gbar_D03_1141451 | G/T | 0.08 | 7.19 |
| Gbar_D03_851878 | T/C | 0.08 | 6.49 | Gbar_D03_909011 | G/C | 0.08 | 6.31 | Gbar_D03_1142341 | A/G | 0.08 | 6.36 |
| Gbar_D03_852191 | C/T | 0.08 | 6.59 | Gbar_D03_909073 | C/T | 0.08 | 6.85 | Gbar_D03_1145550 | T/C | 0.08 | 6.13 |
| Gbar_D03_855664 | G/A | 0.08 | 6.48 | Gbar_D03_910165 | T/C | 0.08 | 6.59 | Gbar_D03_1159232 | A/G | 0.16 | 6.66 |
| Gbar_D03_856654 | C/A | 0.08 | 6.31 | Gbar_D03_911317 | C/A | 0.08 | 6.47 | Gbar_D03_1173668 | C/T | 0.08 | 6.05 |
| Gbar_D03_857269 | C/T | 0.08 | 6.51 | Gbar_D03_911427 | G/A | 0.08 | 6.64 | Gbar_D03_1174973 | G/A | 0.08 | 6.13 |
| Gbar_D03_857949 | A/G | 0.08 | 6.33 | Gbar_D03_913182 | C/T | 0.08 | 6.82 | Gbar_D03_1180020 | G/A | 0.08 | 6.31 |
| Gbar_D03_858238 | T/C | 0.08 | 6.50 | Gbar_D03_913749 | G/A | 0.08 | 6.08 | Gbar_D03_1187747 | T/C | 0.08 | 6.95 |
| Gbar_D03_858711 | C/T | 0.08 | 6.67 | Gbar_D03_913869 | G/A | 0.08 | 6.17 | Gbar_D03_1192013 | G/C | 0.08 | 6.41 |
| Gbar_D03_859170 | G/A | 0.08 | 7.12 | Gbar_D03_914283 | G/C | 0.08 | 6.10 | Gbar_D03_1226116 | G/A | 0.08 | 6.16 |
| Gbar_D03_860521 | A/G | 0.08 | 6.52 | Gbar_D03_915438 | C/G | 0.08 | 6.63 | Gbar_D03_1266739 | G/A | 0.08 | 6.15 |
| Gbar_D03_860559 | G/A | 0.08 | 6.06 | Gbar_D03_916489 | T/G | 0.08 | 6.07 | Gbar_D03_1269091 | A/T | 0.08 | 7.05 |
| Gbar_D03_860585 | C/T | 0.08 | 6.28 | Gbar_D03_918390 | G/A | 0.08 | 7.03 | Gbar_D03_1274626 | C/G | 0.08 | 6.23 |
| Gbar_D03_861068 | T/C | 0.08 | 6.59 | Gbar_D03_918454 | A/G | 0.08 | 6.98 | Gbar_D03_1277678 | A/T | 0.08 | 6.87 |
| Gbar_D03_861112 | A/G | 0.08 | 6.83 | Gbar_D03_919295 | G/T | 0.08 | 6.21 | Gbar_D03_1279352 | C/T | 0.08 | 6.75 |
| Gbar_D03_861183 | T/C | 0.08 | 6.80 | Gbar_D03_919316 | A/T | 0.08 | 6.33 | Gbar_D03_1279385 | G/A | 0.10 | 6.32 |
| Gbar_D03_861307 | G/A | 0.08 | 6.18 | Gbar_D03_919480 | T/G | 0.08 | 6.21 | Gbar_D03_1281305 | C/T | 0.07 | 6.25 |
| Gbar_D03_861348 | C/T | 0.08 | 6.24 | Gbar_D03_919504 | G/A | 0.08 | 6.30 | Gbar_D03_1319515 | C/T | 0.08 | 7.33 |
| Gbar_D03_861362 | A/G | 0.08 | 6.48 | Gbar_D03_920104 | A/G | 0.08 | 7.30 | Gbar_D03_1321491 | C/T | 0.08 | 6.46 |
| Gbar_D03_861829 | G/T | 0.08 | 6.01 | Gbar_D03_922129 | T/C | 0.08 | 6.43 | Gbar_D03_1323180 | C/A | 0.08 | 6.75 |
| Gbar_D03_861889 | A/G | 0.08 | 6.39 | Gbar_D03_923381 | C/T | 0.08 | 6.27 | Gbar_D03_1329020 | A/G | 0.13 | 6.18 |
| Gbar_D03_863216 | A/G | 0.08 | 6.79 | Gbar_D03_925438 | T/A | 0.08 | 6.25 | Gbar_D03_1330863 | A/G | 0.13 | 6.40 |
| Gbar_D03_863288 | T/A | 0.08 | 6.77 | Gbar_D03_925463 | C/T | 0.08 | 6.51 | Gbar_D03_1332091 | T/G | 0.12 | 7.07 |
| Gbar_D03_863380 | A/G | 0.08 | 6.74 | Gbar_D03_925948 | C/T | 0.08 | 6.20 | Gbar_D03_1359810 | A/G | 0.09 | 7.79 |
| Gbar_D03_864223 | T/C | 0.08 | 6.18 | Gbar_D03_926451 | G/A | 0.08 | 6.11 | Gbar_D03_1456870 | G/A | 0.08 | 6.13 |
| Gbar_D03_864909 | A/G | 0.08 | 6.69 | Gbar_D03_926550 | A/G | 0.08 | 6.49 | Gbar_D03_1460925 | G/C | 0.08 | 6.18 |
| Gbar_D03_865179 | G/A | 0.08 | 6.50 | Gbar_D03_927886 | C/T | 0.08 | 6.05 | Gbar_D03_1471416 | G/A | 0.09 | 6.64 |
| Gbar_D03_865347 | T/C | 0.08 | 6.89 | Gbar_D03_928268 | A/C | 0.08 | 6.15 | Gbar_D03_1471482 | C/A | 0.09 | 6.12 |
| Gbar_D03_866738 | C/A | 0.08 | 6.51 | Gbar_D03_928586 | T/A | 0.08 | 6.54 | Gbar_D03_1501667 | T/C | 0.09 | 6.46 |
| Gbar_D03_866739 | G/T | 0.08 | 6.45 | Gbar_D03_928931 | A/T | 0.08 | 6.39 | Gbar_D03_1501683 | C/T | 0.08 | 6.26 |
| Gbar_D03_867612 | T/A | 0.08 | 6.75 | Gbar_D03_928963 | T/A | 0.08 | 6.88 | Gbar_D03_1509599 | T/C | 0.08 | 6.27 |
| Gbar_D03_868367 | G/A | 0.08 | 6.76 | Gbar_D03_929263 | A/C | 0.08 | 6.44 | Gbar_D03_1513731 | A/T | 0.08 | 6.34 |
| Gbar_D03_868547 | T/C | 0.08 | 6.43 | Gbar_D03_929354 | C/T | 0.08 | 6.19 | Gbar_D03_1513733 | C/A | 0.09 | 6.40 |
| Gbar_D03_869436 | C/A | 0.08 | 6.83 | Gbar_D03_929439 | A/G | 0.08 | 6.85 | Gbar_D03_1513734 | C/A | 0.09 | 6.25 |
| Gbar_D03_870721 | G/T | 0.08 | 6.77 | Gbar_D03_929567 | T/C | 0.08 | 6.33 | Gbar_D03_1531348 | C/T | 0.09 | 6.27 |
| Gbar_D03_872949 | C/T | 0.08 | 6.68 | Gbar_D03_929578 | C/T | 0.08 | 6.16 | Gbar_D03_1534765 | C/T | 0.08 | 6.11 |
| Gbar_D03_875334 | T/A | 0.08 | 6.74 | Gbar_D03_929895 | A/G | 0.08 | 6.59 | Gbar_D03_1537617 | T/G | 0.08 | 6.12 |
| Gbar_D03_876178 | A/G | 0.08 | 6.45 | Gbar_D03_929903 | C/T | 0.08 | 6.61 | Gbar_D03_1541480 | A/G | 0.08 | 6.25 |
| Gbar_D03_876290 | G/T | 0.08 | 6.05 | Gbar_D03_929941 | T/C | 0.08 | 6.57 | Gbar_D03_1541489 | T/A | 0.09 | 6.41 |
| Gbar_D03_877654 | G/A | 0.08 | 6.76 | Gbar_D03_930105 | T/A | 0.08 | 6.72 | Gbar_D03_1601122 | C/T | 0.08 | 6.54 |
| Gbar_D03_880446 | C/G | 0.08 | 6.71 | Gbar_D03_930482 | T/A | 0.08 | 6.33 | Gbar_D03_1601160 | C/T | 0.08 | 6.33 |
| Gbar_D03_881676 | G/A | 0.08 | 6.86 | Gbar_D03_930562 | G/A | 0.08 | 6.33 | Gbar_D03_1620201 | A/C | 0.08 | 6.77 |
| Gbar_D03_882444 | T/A | 0.08 | 6.66 | Gbar_D03_932359 | G/T | 0.08 | 6.99 | Gbar_D03_1629970 | C/T | 0.18 | 6.94 |
| Gbar_D03_882817 | T/A | 0.08 | 6.92 | Gbar_D03_932811 | T/C | 0.08 | 6.80 | Gbar_D03_1638142 | A/C | 0.08 | 6.42 |
| Gbar_D03_883704 | C/G | 0.08 | 6.15 | Gbar_D03_932910 | A/G | 0.08 | 6.36 | Gbar_D03_1663528 | G/A | 0.08 | 6.17 |
| Gbar_D03_884147 | A/T | 0.08 | 6.39 | Gbar_D03_933036 | A/T | 0.08 | 6.43 | Gbar_D03_1672432 | A/T | 0.08 | 6.52 |
| Gbar_D03_884158 | A/T | 0.08 | 6.31 | Gbar_D03_934504 | C/T | 0.08 | 6.12 | Gbar_D03_1672718 | G/A | 0.19 | 6.09 |
| Gbar_D03_884404 | A/C | 0.07 | 6.39 | Gbar_D03_934831 | A/T | 0.08 | 6.31 | Gbar_D03_1686833 | A/G | 0.08 | 6.24 |
| Gbar_D03_884492 | A/G | 0.08 | 6.51 | Gbar_D03_935005 | T/C | 0.08 | 6.31 | Gbar_D03_1699544 | G/A | 0.08 | 6.13 |
| Gbar_D03_884660 | A/C | 0.08 | 6.02 | Gbar_D03_935178 | C/T | 0.08 | 6.04 | Gbar_D03_1700917 | T/G | 0.08 | 6.31 |
| Gbar_D03_885798 | A/T | 0.08 | 6.36 | Gbar_D03_935248 | G/A | 0.08 | 7.21 | Gbar_D03_1703450 | T/A | 0.17 | 6.26 |
| Gbar_D03_885841 | C/A | 0.08 | 6.12 | Gbar_D03_935584 | T/C | 0.08 | 6.76 | Gbar_D03_1705967 | A/T | 0.08 | 6.33 |
| Gbar_D03_886259 | C/T | 0.08 | 6.51 | Gbar_D03_936037 | A/T | 0.08 | 6.10 | Gbar_D03_1714170 | A/G | 0.09 | 6.07 |
| Gbar_D03_886524 | C/A | 0.08 | 6.27 | Gbar_D03_936082 | T/A | 0.08 | 6.20 | Gbar_D03_1824899 | T/C | 0.18 | 6.65 |
| Gbar_D03_886537 | C/T | 0.08 | 6.24 | Gbar_D03_936812 | A/G | 0.08 | 6.67 | Gbar_D03_1839061 | G/A | 0.09 | 6.35 |
| Gbar_D03_887891 | A/G | 0.08 | 6.03 | Gbar_D03_937095 | T/A | 0.08 | 6.13 | Gbar_D03_1843065 | T/G | 0.17 | 6.50 |
| Gbar_D03_888250 | T/C | 0.08 | 7.21 | Gbar_D03_937309 | G/T | 0.07 | 6.48 | Gbar_D03_1853808 | T/C | 0.09 | 7.24 |
| Gbar_D03_888570 | T/C | 0.08 | 6.09 | Gbar_D03_937576 | T/C | 0.08 | 6.20 | Gbar_D03_1855413 | C/T | 0.09 | 6.13 |
| Gbar_D03_889762 | G/A | 0.08 | 6.15 | Gbar_D03_937673 | A/T | 0.08 | 6.93 | Gbar_D03_1860429 | G/A | 0.08 | 6.94 |
| Gbar_D03_889853 | G/A | 0.08 | 6.67 | Gbar_D03_937692 | G/A | 0.08 | 7.00 | Gbar_D03_1876166 | G/C | 0.09 | 6.97 |
| Gbar_D03_890328 | G/A | 0.08 | 6.85 | Gbar_D03_938167 | A/G | 0.08 | 6.04 | Gbar_D03_1878030 | T/C | 0.08 | 7.41 |
| Gbar_D03_891244 | C/G | 0.09 | 6.13 | Gbar_D03_939095 | C/T | 0.08 | 6.86 | Gbar_D03_1879874 | T/C | 0.09 | 7.27 |
| Gbar_D03_891425 | A/T | 0.08 | 6.40 | Gbar_D03_939478 | G/A | 0.08 | 6.70 | Gbar_D03_1881025 | G/A | 0.09 | 7.38 |
| Gbar_D03_891465 | T/C | 0.08 | 6.70 | Gbar_D03_940347 | A/T | 0.08 | 6.48 | Gbar_D03_1918318 | C/A | 0.08 | 6.63 |
| Gbar_D03_891771 | A/C | 0.08 | 6.60 | Gbar_D03_940981 | T/G | 0.08 | 6.19 | Gbar_D03_1918338 | A/G | 0.09 | 6.29 |
| Gbar_D03_891846 | A/G | 0.08 | 6.02 | Gbar_D03_942540 | A/G | 0.08 | 6.89 | Gbar_D03_1918465 | C/A | 0.09 | 6.48 |
| Gbar_D03_892558 | A/G | 0.08 | 6.57 | Gbar_D03_942558 | C/T | 0.08 | 7.09 | Gbar_D03_1922237 | A/T | 0.08 | 7.25 |
| Gbar_D03_892638 | A/G | 0.08 | 6.67 | Gbar_D03_942661 | G/A | 0.08 | 6.52 | Gbar_D03_1922363 | G/A | 0.09 | 6.98 |
| Gbar_D03_892644 | G/A | 0.08 | 6.68 | Gbar_D03_942735 | A/C | 0.08 | 6.70 | Gbar_D03_1922408 | T/C | 0.09 | 7.13 |
| Gbar_D03_892901 | T/C | 0.08 | 6.03 | Gbar_D03_942849 | C/A | 0.08 | 6.76 | Gbar_D03_1923098 | A/G | 0.08 | 7.07 |
| Gbar_D03_893090 | T/C | 0.08 | 6.35 | Gbar_D03_942868 | A/G | 0.08 | 6.02 | Gbar_D03_1926752 | T/C | 0.08 | 6.74 |
| Gbar_D03_893103 | C/T | 0.08 | 6.18 | Gbar_D03_953927 | G/A | 0.08 | 6.95 | Gbar_D03_1926776 | G/A | 0.08 | 6.65 |
| Gbar_D03_893264 | A/G | 0.08 | 6.32 | Gbar_D03_959041 | A/G | 0.08 | 6.46 | Gbar_D03_1926884 | G/A | 0.09 | 6.03 |
| Gbar_D03_893355 | C/G | 0.08 | 6.42 | Gbar_D03_968233 | A/T | 0.08 | 6.58 | Gbar_D03_1928830 | G/T | 0.09 | 6.77 |
| Gbar_D03_893458 | A/G | 0.08 | 6.64 | Gbar_D03_970200 | A/C | 0.08 | 6.09 | Gbar_D03_1941440 | A/T | 0.09 | 6.98 |
| Gbar_D03_893670 | T/A | 0.08 | 6.41 | Gbar_D03_987802 | T/G | 0.09 | 6.15 | Gbar_D03_1956848 | T/C | 0.09 | 6.26 |
| Gbar_D03_895012 | T/A | 0.08 | 6.65 | **2016** |  |  |  | Gbar_D03_1957237 | T/G | 0.09 | 7.42 |
| Gbar_D03_895774 | T/A | 0.08 | 6.28 | Gbar_D03_1001198 | G/A | 0.08 | 6.94 | Gbar_D03_1980162 | A/T | 0.12 | 6.49 |
| Gbar_D03_895885 | C/T | 0.08 | 6.07 | Gbar_D03_1021128 | T/C | 0.08 | 6.50 | Gbar_D03_1989223 | C/T | 0.09 | 6.82 |
| Gbar_D03_895969 | A/G | 0.08 | 6.29 | Gbar_D03_1033138 | A/G | 0.09 | 6.93 | Gbar_D03_1989306 | C/T | 0.09 | 6.12 |
| Gbar_D03_896192 | G/A | 0.08 | 6.57 | Gbar_D03_1041984 | G/A | 0.08 | 6.45 | Gbar_D03_1989485 | T/C | 0.18 | 6.58 |
| Gbar_D03_896279 | C/T | 0.08 | 6.09 | Gbar_D03_1063181 | T/C | 0.15 | 6.09 | Gbar_D03_2004879 | C/T | 0.08 | 6.37 |
| Gbar_D03_896764 | A/T | 0.08 | 6.58 | Gbar_D03_1064857 | C/G | 0.09 | 7.04 | Gbar_D03_2019628 | C/T | 0.09 | 7.06 |
| Gbar_D03_897819 | A/C | 0.08 | 6.83 | Gbar_D03_1068053 | A/G | 0.15 | 6.45 | Gbar_D03_2022615 | T/G | 0.09 | 6.98 |
| Gbar_D03_897899 | G/C | 0.08 | 6.20 | Gbar_D03_1075812 | A/G | 0.15 | 6.34 | Gbar_D03_2022638 | G/A | 0.08 | 6.10 |
| Gbar_D03_897901 | C/T | 0.08 | 6.22 | Gbar_D03_1076579 | T/C | 0.15 | 6.36 | Gbar_D03_2035829 | T/G | 0.09 | 6.04 |
| Gbar_D03_898193 | G/A | 0.08 | 6.62 | Gbar_D03_1076588 | G/A | 0.15 | 6.54 | Gbar_D03_2035835 | T/C | 0.09 | 6.42 |
| Gbar_D03_898245 | G/A | 0.08 | 6.38 | Gbar_D03_1101128 | A/G | 0.16 | 6.13 | Gbar_D03_2047669 | T/C | 0.18 | 6.30 |
| Gbar_D03_898709 | A/G | 0.08 | 6.49 | Gbar_D03_1101164 | G/A | 0.16 | 6.05 | Gbar_D03_2056886 | C/A | 0.09 | 6.78 |
| Gbar_D03_899069 | T/C | 0.08 | 6.40 | Gbar_D03_1105006 | A/G | 0.08 | 6.78 | Gbar_D03_2057743 | C/T | 0.09 | 7.24 |
| Gbar_D03_900147 | C/T | 0.08 | 6.17 | Gbar_D03_1105012 | G/T | 0.16 | 7.70 | Gbar_D03_2081936 | T/G | 0.08 | 7.48 |
| Gbar_D03_900804 | T/C | 0.08 | 6.68 | Gbar_D03_1105045 | C/T | 0.08 | 6.44 | Gbar_D03_2081948 | A/G | 0.08 | 7.71 |
| Gbar_D03_901197 | G/A | 0.07 | 6.30 | Gbar_D03_1107259 | A/C | 0.16 | 6.78 | Gbar_D03_2081955 | G/A | 0.08 | 6.19 |
| Gbar_D03_901272 | G/A | 0.08 | 6.60 | Gbar_D03_1107653 | T/C | 0.16 | 6.08 | Gbar_D03_2112513 | G/A | 0.08 | 6.45 |
| Gbar_D03_901276 | G/T | 0.08 | 6.76 | Gbar_D03_1108433 | A/G | 0.16 | 6.39 | Gbar_D03_2112544 | G/A | 0.08 | 6.10 |
| Gbar_D03_901536 | C/A | 0.08 | 6.87 | Gbar_D03_1110567 | A/G | 0.21 | 8.11 | Gbar_D03_2125937 | T/C | 0.09 | 6.54 |
| Gbar_D03_902288 | G/C | 0.08 | 6.44 | Gbar_D03_1116342 | G/A | 0.16 | 6.75 | Gbar_D03_2129927 | G/A | 0.08 | 6.47 |
| Gbar_D03_903930 | T/A | 0.08 | 7.07 | Gbar_D03_1116402 | A/G | 0.16 | 6.40 | Gbar_D03_2130833 | C/G | 0.08 | 6.47 |

**Table S2.** List of the associated SNPs identified in different datasets through GWAS for *FW* resistance in Sea Island cotton. (Continued)

| SNP | Ref/Alt | MAF | -log (p-value) | SNP | Ref/Alt | MAF | -log (p-value) | SNP | Ref/Alt | MAF | -log (p-value) |
| --- | --- | --- | --- | --- | --- | --- | --- | --- | --- | --- | --- |
| **2016** |  |  |  | Gbar_D03_245980 | A/T | 0.08 | 6.63 | Gbar_D03_315553 | C/G | 0.08 | 7.29 |
| Gbar_D03_2137861 | A/G | 0.08 | 6.95 | Gbar_D03_246179 | C/G | 0.08 | 6.76 | Gbar_D03_315594 | A/G | 0.08 | 7.04 |
| Gbar_D03_220809 | A/T | 0.08 | 6.58 | Gbar_D03_246396 | G/T | 0.07 | 6.42 | Gbar_D03_317643 | T/G | 0.08 | 6.98 |
| Gbar_D03_220931 | A/G | 0.08 | 6.46 | Gbar_D03_246409 | C/A | 0.07 | 6.24 | Gbar_D03_317762 | G/A | 0.08 | 6.29 |
| Gbar_D03_220948 | G/C | 0.08 | 6.62 | Gbar_D03_246540 | T/C | 0.08 | 7.18 | Gbar_D03_317766 | G/A | 0.08 | 6.12 |
| Gbar_D03_221262 | T/C | 0.08 | 6.48 | Gbar_D03_247958 | A/G | 0.08 | 7.09 | Gbar_D03_317770 | A/G | 0.08 | 6.19 |
| Gbar_D03_2214857 | A/G | 0.16 | 6.51 | Gbar_D03_248386 | A/G | 0.08 | 6.40 | Gbar_D03_325034 | C/T | 0.08 | 6.91 |
| Gbar_D03_2215669 | A/T | 0.17 | 6.94 | Gbar_D03_248567 | A/G | 0.08 | 6.73 | Gbar_D03_325159 | A/G | 0.08 | 6.43 |
| Gbar_D03_222160 | T/C | 0.08 | 6.63 | Gbar_D03_248695 | T/C | 0.07 | 6.15 | Gbar_D03_325218 | T/A | 0.08 | 6.32 |
| Gbar_D03_222186 | T/C | 0.08 | 6.90 | Gbar_D03_250966 | C/T | 0.08 | 6.82 | Gbar_D03_326108 | C/T | 0.08 | 6.90 |
| Gbar_D03_222231 | A/G | 0.08 | 7.01 | Gbar_D03_251337 | T/C | 0.08 | 6.40 | Gbar_D03_326859 | G/C | 0.08 | 6.42 |
| Gbar_D03_222232 | A/G | 0.08 | 7.01 | Gbar_D03_251461 | C/G | 0.08 | 6.35 | Gbar_D03_327186 | T/A | 0.08 | 6.22 |
| Gbar_D03_222249 | A/G | 0.08 | 7.04 | Gbar_D03_251470 | C/G | 0.08 | 6.34 | Gbar_D03_327187 | T/A | 0.07 | 6.57 |
| Gbar_D03_2222499 | T/C | 0.16 | 6.43 | Gbar_D03_251539 | C/G | 0.08 | 6.46 | Gbar_D03_327895 | A/G | 0.08 | 6.81 |
| Gbar_D03_2222921 | A/G | 0.17 | 6.48 | Gbar_D03_252667 | G/T | 0.08 | 6.49 | Gbar_D03_328439 | C/T | 0.08 | 6.57 |
| Gbar_D03_2223697 | G/T | 0.09 | 6.34 | Gbar_D03_253369 | G/T | 0.08 | 6.42 | Gbar_D03_328537 | A/T | 0.07 | 6.03 |
| Gbar_D03_2224222 | A/G | 0.20 | 6.30 | Gbar_D03_254335 | T/A | 0.08 | 6.20 | Gbar_D03_335127 | A/C | 0.08 | 6.95 |
| Gbar_D03_2224946 | A/G | 0.16 | 6.82 | Gbar_D03_255137 | C/T | 0.08 | 6.73 | Gbar_D03_335393 | T/A | 0.08 | 6.37 |
| Gbar_D03_222590 | C/G | 0.08 | 6.43 | Gbar_D03_255247 | C/T | 0.08 | 6.69 | Gbar_D03_336173 | T/C | 0.08 | 7.12 |
| Gbar_D03_222591 | C/G | 0.08 | 6.65 | Gbar_D03_256410 | T/C | 0.08 | 6.71 | Gbar_D03_336255 | A/G | 0.08 | 7.20 |
| Gbar_D03_222616 | A/G | 0.08 | 6.58 | Gbar_D03_257735 | G/C | 0.08 | 6.41 | Gbar_D03_336492 | A/G | 0.07 | 6.41 |
| Gbar_D03_222655 | G/T | 0.08 | 7.17 | Gbar_D03_258830 | C/A | 0.08 | 7.12 | Gbar_D03_337013 | A/C | 0.08 | 6.43 |
| Gbar_D03_222693 | T/C | 0.07 | 6.90 | Gbar_D03_259632 | C/G | 0.07 | 6.49 | Gbar_D03_337300 | A/T | 0.08 | 6.86 |
| Gbar_D03_2228451 | A/G | 0.14 | 6.05 | Gbar_D03_261728 | C/T | 0.07 | 6.61 | Gbar_D03_337548 | T/G | 0.07 | 6.24 |
| Gbar_D03_222937 | G/A | 0.08 | 7.00 | Gbar_D03_263048 | T/C | 0.07 | 6.05 | Gbar_D03_337829 | G/T | 0.08 | 7.00 |
| Gbar_D03_222941 | C/A | 0.08 | 6.61 | Gbar_D03_263853 | C/T | 0.08 | 6.59 | Gbar_D03_337979 | C/G | 0.07 | 6.21 |
| Gbar_D03_223181 | C/T | 0.08 | 6.62 | Gbar_D03_263871 | C/G | 0.08 | 6.48 | Gbar_D03_338062 | C/T | 0.08 | 6.44 |
| Gbar_D03_223331 | G/A | 0.08 | 6.31 | Gbar_D03_264339 | A/G | 0.08 | 6.21 | Gbar_D03_338447 | A/T | 0.08 | 6.57 |
| Gbar_D03_223405 | A/G | 0.07 | 6.28 | Gbar_D03_264342 | C/T | 0.07 | 6.03 | Gbar_D03_338578 | C/T | 0.08 | 6.58 |
| Gbar_D03_223567 | C/A | 0.08 | 6.17 | Gbar_D03_264452 | C/T | 0.08 | 6.17 | Gbar_D03_339295 | C/T | 0.08 | 6.19 |
| Gbar_D03_224081 | T/C | 0.08 | 6.54 | Gbar_D03_264598 | A/T | 0.07 | 6.33 | Gbar_D03_339349 | C/A | 0.08 | 6.40 |
| Gbar_D03_224097 | C/T | 0.08 | 6.61 | Gbar_D03_264615 | A/G | 0.08 | 6.42 | Gbar_D03_340016 | A/G | 0.08 | 6.18 |
| Gbar_D03_224098 | G/T | 0.08 | 6.87 | Gbar_D03_265107 | T/A | 0.08 | 6.22 | Gbar_D03_341144 | T/C | 0.08 | 6.15 |
| Gbar_D03_224122 | T/C | 0.08 | 7.08 | Gbar_D03_266246 | A/C | 0.08 | 7.45 | Gbar_D03_341171 | G/A | 0.08 | 7.77 |
| Gbar_D03_224295 | C/G | 0.08 | 6.60 | Gbar_D03_267536 | A/G | 0.07 | 6.37 | Gbar_D03_341305 | C/A | 0.08 | 6.67 |
| Gbar_D03_224785 | C/T | 0.08 | 6.47 | Gbar_D03_267782 | T/C | 0.08 | 6.81 | Gbar_D03_341872 | G/A | 0.08 | 6.36 |
| Gbar_D03_2249026 | C/T | 0.09 | 7.28 | Gbar_D03_267841 | A/T | 0.08 | 6.85 | Gbar_D03_342392 | T/A | 0.08 | 6.49 |
| Gbar_D03_2249706 | T/C | 0.15 | 6.85 | Gbar_D03_267866 | A/G | 0.08 | 6.69 | Gbar_D03_342724 | A/G | 0.08 | 6.60 |
| Gbar_D03_2255845 | T/G | 0.14 | 6.29 | Gbar_D03_268910 | T/A | 0.08 | 6.66 | Gbar_D03_342870 | A/G | 0.08 | 6.86 |
| Gbar_D03_2256558 | T/A | 0.14 | 6.15 | Gbar_D03_269537 | A/T | 0.08 | 6.55 | Gbar_D03_343188 | C/G | 0.08 | 6.72 |
| Gbar_D03_2256560 | T/A | 0.14 | 6.79 | Gbar_D03_269574 | T/A | 0.08 | 6.12 | Gbar_D03_343677 | T/C | 0.08 | 6.56 |
| Gbar_D03_225809 | T/C | 0.08 | 6.02 | Gbar_D03_275717 | C/T | 0.08 | 6.30 | Gbar_D03_344490 | T/C | 0.08 | 6.74 |
| Gbar_D03_226153 | A/T | 0.08 | 6.55 | Gbar_D03_276754 | G/A | 0.08 | 6.87 | Gbar_D03_344918 | T/A | 0.08 | 6.55 |
| Gbar_D03_226586 | C/T | 0.07 | 6.66 | Gbar_D03_276895 | G/A | 0.08 | 6.04 | Gbar_D03_345367 | A/G | 0.07 | 6.15 |
| Gbar_D03_227406 | C/T | 0.08 | 6.98 | Gbar_D03_276918 | A/T | 0.08 | 6.13 | Gbar_D03_345426 | G/A | 0.08 | 6.79 |
| Gbar_D03_227599 | A/T | 0.08 | 7.35 | Gbar_D03_276927 | A/T | 0.08 | 6.01 | Gbar_D03_346133 | A/T | 0.08 | 7.58 |
| Gbar_D03_228487 | G/A | 0.07 | 6.03 | Gbar_D03_277326 | A/T | 0.08 | 6.86 | Gbar_D03_346147 | G/T | 0.08 | 7.79 |
| Gbar_D03_228490 | T/G | 0.07 | 6.06 | Gbar_D03_280479 | T/C | 0.08 | 6.72 | Gbar_D03_346174 | A/T | 0.08 | 7.91 |
| Gbar_D03_228548 | G/A | 0.07 | 6.92 | Gbar_D03_280483 | T/A | 0.08 | 6.80 | Gbar_D03_347075 | G/A | 0.08 | 6.77 |
| Gbar_D03_228570 | A/G | 0.08 | 6.80 | Gbar_D03_280605 | C/T | 0.08 | 6.82 | Gbar_D03_347151 | T/C | 0.08 | 7.04 |
| Gbar_D03_228596 | A/G | 0.08 | 6.73 | Gbar_D03_281099 | C/T | 0.08 | 6.80 | Gbar_D03_348413 | G/A | 0.08 | 6.59 |
| Gbar_D03_228613 | A/G | 0.07 | 6.05 | Gbar_D03_281165 | G/A | 0.08 | 6.66 | Gbar_D03_349195 | A/G | 0.08 | 6.75 |
| Gbar_D03_228651 | A/C | 0.08 | 6.24 | Gbar_D03_281564 | T/G | 0.08 | 6.73 | Gbar_D03_350601 | G/A | 0.08 | 6.75 |
| Gbar_D03_228685 | T/C | 0.07 | 6.29 | Gbar_D03_284501 | G/A | 0.08 | 6.36 | Gbar_D03_350909 | G/A | 0.08 | 6.20 |
| Gbar_D03_228760 | C/T | 0.07 | 6.08 | Gbar_D03_285695 | T/G | 0.07 | 6.72 | Gbar_D03_351193 | C/G | 0.08 | 7.53 |
| Gbar_D03_228776 | C/T | 0.07 | 6.21 | Gbar_D03_285970 | G/A | 0.08 | 7.01 | Gbar_D03_351674 | T/C | 0.07 | 6.18 |
| Gbar_D03_228780 | C/A | 0.07 | 6.17 | Gbar_D03_287296 | T/A | 0.08 | 6.95 | Gbar_D03_351774 | G/A | 0.08 | 6.06 |
| Gbar_D03_228815 | T/C | 0.08 | 6.88 | Gbar_D03_289432 | C/A | 0.08 | 7.07 | Gbar_D03_351798 | G/A | 0.07 | 6.08 |
| Gbar_D03_228992 | T/G | 0.08 | 6.16 | Gbar_D03_290201 | G/A | 0.08 | 7.04 | Gbar_D03_351883 | A/G | 0.08 | 6.89 |
| Gbar_D03_229404 | A/G | 0.07 | 6.09 | Gbar_D03_290416 | A/T | 0.08 | 6.67 | Gbar_D03_351895 | A/G | 0.08 | 6.90 |
| Gbar_D03_229831 | C/T | 0.08 | 6.91 | Gbar_D03_290586 | T/A | 0.08 | 7.07 | Gbar_D03_351917 | G/A | 0.08 | 6.53 |
| Gbar_D03_229844 | T/C | 0.08 | 6.86 | Gbar_D03_291025 | A/T | 0.08 | 6.51 | Gbar_D03_351927 | A/G | 0.08 | 6.80 |
| Gbar_D03_230010 | T/C | 0.08 | 6.81 | Gbar_D03_291710 | G/A | 0.08 | 6.59 | Gbar_D03_352115 | A/G | 0.08 | 6.26 |
| Gbar_D03_230011 | G/C | 0.08 | 6.55 | Gbar_D03_291826 | G/A | 0.08 | 6.14 | Gbar_D03_353095 | T/C | 0.08 | 7.01 |
| Gbar_D03_230025 | C/G | 0.08 | 6.62 | Gbar_D03_292036 | A/T | 0.07 | 6.59 | Gbar_D03_353785 | T/C | 0.07 | 6.41 |
| Gbar_D03_230553 | C/G | 0.08 | 7.16 | Gbar_D03_292283 | T/G | 0.08 | 6.37 | Gbar_D03_353967 | A/G | 0.08 | 6.10 |
| Gbar_D03_230585 | A/G | 0.08 | 6.77 | Gbar_D03_293205 | A/T | 0.08 | 6.12 | Gbar_D03_354247 | A/G | 0.08 | 6.43 |
| Gbar_D03_231449 | T/A | 0.07 | 6.15 | Gbar_D03_293228 | A/G | 0.08 | 6.42 | Gbar_D03_355098 | C/T | 0.07 | 6.75 |
| Gbar_D03_231767 | C/A | 0.08 | 6.74 | Gbar_D03_293274 | C/T | 0.08 | 6.40 | Gbar_D03_357211 | T/C | 0.08 | 6.64 |
| Gbar_D03_231982 | A/T | 0.08 | 6.35 | Gbar_D03_293403 | T/C | 0.08 | 6.16 | Gbar_D03_357800 | G/A | 0.08 | 6.48 |
| Gbar_D03_232520 | C/A | 0.08 | 6.11 | Gbar_D03_293435 | T/G | 0.08 | 6.22 | Gbar_D03_359041 | G/A | 0.08 | 6.87 |
| Gbar_D03_232691 | T/C | 0.08 | 6.40 | Gbar_D03_295398 | T/C | 0.08 | 6.59 | Gbar_D03_359727 | A/C | 0.08 | 6.00 |
| Gbar_D03_232810 | G/A | 0.09 | 6.95 | Gbar_D03_295844 | A/T | 0.08 | 6.33 | Gbar_D03_359854 | A/C | 0.08 | 6.91 |
| Gbar_D03_233018 | T/C | 0.08 | 6.55 | Gbar_D03_295872 | T/A | 0.08 | 6.19 | Gbar_D03_360080 | C/T | 0.07 | 6.16 |
| Gbar_D03_233033 | G/A | 0.08 | 6.43 | Gbar_D03_296209 | G/T | 0.08 | 7.16 | Gbar_D03_361972 | G/A | 0.08 | 6.85 |
| Gbar_D03_233232 | C/A | 0.07 | 6.22 | Gbar_D03_296278 | A/T | 0.08 | 7.07 | Gbar_D03_362303 | C/T | 0.08 | 6.83 |
| Gbar_D03_233239 | A/T | 0.07 | 6.20 | Gbar_D03_296398 | C/A | 0.08 | 6.35 | Gbar_D03_362551 | T/G | 0.08 | 6.73 |
| Gbar_D03_233431 | G/T | 0.07 | 6.55 | Gbar_D03_296456 | G/A | 0.08 | 6.36 | Gbar_D03_362767 | G/A | 0.08 | 6.85 |
| Gbar_D03_233938 | A/T | 0.08 | 6.25 | Gbar_D03_296723 | G/A | 0.09 | 6.22 | Gbar_D03_362782 | G/A | 0.08 | 6.41 |
| Gbar_D03_236618 | C/T | 0.08 | 6.98 | Gbar_D03_296879 | T/G | 0.08 | 6.20 | Gbar_D03_362973 | A/G | 0.08 | 6.18 |
| Gbar_D03_236653 | A/G | 0.08 | 7.34 | Gbar_D03_297638 | C/T | 0.08 | 6.58 | Gbar_D03_363140 | G/T | 0.08 | 6.94 |
| Gbar_D03_236777 | T/C | 0.08 | 6.85 | Gbar_D03_297696 | C/G | 0.08 | 6.87 | Gbar_D03_363238 | G/A | 0.08 | 6.42 |
| Gbar_D03_236894 | C/T | 0.08 | 6.38 | Gbar_D03_297725 | T/C | 0.08 | 7.12 | Gbar_D03_363257 | C/A | 0.08 | 6.40 |
| Gbar_D03_237064 | G/T | 0.08 | 6.52 | Gbar_D03_297808 | G/C | 0.08 | 6.95 | Gbar_D03_363323 | T/A | 0.08 | 6.11 |
| Gbar_D03_237071 | C/A | 0.08 | 6.74 | Gbar_D03_298221 | A/T | 0.08 | 6.54 | Gbar_D03_363877 | A/G | 0.07 | 6.55 |
| Gbar_D03_237378 | G/A | 0.08 | 7.33 | Gbar_D03_298574 | C/A | 0.07 | 6.01 | Gbar_D03_364835 | C/T | 0.08 | 6.40 |
| Gbar_D03_237484 | C/T | 0.08 | 6.13 | Gbar_D03_298740 | G/A | 0.08 | 6.88 | Gbar_D03_364890 | C/T | 0.08 | 6.40 |
| Gbar_D03_237550 | T/A | 0.08 | 6.70 | Gbar_D03_298944 | T/C | 0.08 | 6.43 | Gbar_D03_364949 | C/T | 0.08 | 6.04 |
| Gbar_D03_237715 | C/T | 0.08 | 6.45 | Gbar_D03_300323 | A/T | 0.08 | 6.46 | Gbar_D03_365221 | T/C | 0.08 | 6.32 |
| Gbar_D03_237734 | G/A | 0.08 | 6.48 | Gbar_D03_300361 | G/A | 0.08 | 6.79 | Gbar_D03_366012 | G/A | 0.08 | 6.15 |
| Gbar_D03_237828 | A/G | 0.07 | 6.31 | Gbar_D03_302594 | A/G | 0.07 | 6.60 | Gbar_D03_366106 | G/A | 0.08 | 6.70 |
| Gbar_D03_237923 | G/A | 0.07 | 6.05 | Gbar_D03_305070 | T/G | 0.08 | 6.98 | Gbar_D03_366280 | G/C | 0.08 | 6.45 |
| Gbar_D03_238573 | T/C | 0.07 | 6.05 | Gbar_D03_305083 | A/G | 0.08 | 7.09 | Gbar_D03_367761 | G/A | 0.07 | 6.27 |
| Gbar_D03_238766 | G/T | 0.07 | 6.16 | Gbar_D03_306408 | A/G | 0.08 | 6.01 | Gbar_D03_367828 | T/C | 0.08 | 7.26 |
| Gbar_D03_239840 | C/T | 0.07 | 6.05 | Gbar_D03_306763 | T/C | 0.08 | 6.20 | Gbar_D03_367935 | T/C | 0.07 | 6.63 |
| Gbar_D03_240787 | T/C | 0.08 | 6.37 | Gbar_D03_307265 | C/T | 0.08 | 6.53 | Gbar_D03_368207 | T/A | 0.08 | 6.82 |
| Gbar_D03_241600 | A/G | 0.08 | 6.15 | Gbar_D03_307978 | A/G | 0.07 | 6.53 | Gbar_D03_368537 | G/A | 0.08 | 6.67 |
| Gbar_D03_241686 | G/A | 0.08 | 6.09 | Gbar_D03_307986 | G/A | 0.07 | 6.59 | Gbar_D03_368561 | A/G | 0.08 | 6.87 |
| Gbar_D03_241833 | A/C | 0.07 | 6.13 | Gbar_D03_308036 | C/A | 0.07 | 6.64 | Gbar_D03_368579 | T/C | 0.08 | 6.72 |
| Gbar_D03_241847 | A/G | 0.07 | 6.07 | Gbar_D03_308087 | T/C | 0.08 | 6.85 | Gbar_D03_368619 | G/A | 0.08 | 7.31 |
| Gbar_D03_242138 | G/A | 0.08 | 6.68 | Gbar_D03_309082 | G/T | 0.08 | 6.48 | Gbar_D03_369041 | T/C | 0.08 | 6.96 |
| Gbar_D03_242166 | T/A | 0.07 | 6.83 | Gbar_D03_310128 | T/C | 0.07 | 6.49 | Gbar_D03_369550 | A/G | 0.07 | 6.49 |
| Gbar_D03_242755 | C/T | 0.08 | 6.91 | Gbar_D03_310501 | T/C | 0.07 | 6.06 | Gbar_D03_369830 | T/G | 0.08 | 7.23 |
| Gbar_D03_243816 | G/T | 0.08 | 6.84 | Gbar_D03_310752 | T/C | 0.08 | 6.33 | Gbar_D03_369956 | T/G | 0.07 | 6.15 |
| Gbar_D03_244284 | C/T | 0.08 | 6.62 | Gbar_D03_311216 | T/G | 0.08 | 6.33 | Gbar_D03_369979 | A/G | 0.08 | 6.82 |
| Gbar_D03_244354 | C/T | 0.08 | 6.14 | Gbar_D03_311296 | T/G | 0.08 | 6.64 | Gbar_D03_370056 | G/T | 0.08 | 6.25 |
| Gbar_D03_245344 | C/T | 0.07 | 6.37 | Gbar_D03_311607 | A/T | 0.08 | 6.51 | Gbar_D03_370347 | T/C | 0.07 | 6.04 |
| Gbar_D03_245629 | A/G | 0.08 | 6.37 | Gbar_D03_311657 | T/C | 0.08 | 6.10 | Gbar_D03_370407 | G/A | 0.07 | 6.03 |
| Gbar_D03_245656 | G/A | 0.08 | 6.19 | Gbar_D03_311975 | G/A | 0.08 | 6.11 | Gbar_D03_371073 | G/A | 0.08 | 6.76 |
| Gbar_D03_245684 | C/A | 0.08 | 6.90 | Gbar_D03_314965 | A/G | 0.07 | 6.45 | Gbar_D03_371111 | C/T | 0.08 | 6.87 |

**Table S2.** List of the associated SNPs identified in different datasets through GWAS for *FW* resistance in Sea Island cotton. (Continued)

| SNP | Ref/Alt | MAF | -log (p-value) | SNP | Ref/Alt | MAF | -log (p-value) | SNP | Ref/Alt | MAF | -log (p-value) |
| --- | --- | --- | --- | --- | --- | --- | --- | --- | --- | --- | --- |
| **2016** |  |  |  | Gbar_D03_426537 | G/C | 0.08 | 6.08 | Gbar_D03_457126 | A/G | 0.08 | 7.06 |
| Gbar_D03_371364 | G/A | 0.08 | 6.84 | Gbar_D03_426566 | T/C | 0.08 | 6.17 | Gbar_D03_457147 | A/G | 0.08 | 7.19 |
| Gbar_D03_371913 | T/C | 0.08 | 6.75 | Gbar_D03_426619 | T/A | 0.08 | 6.15 | Gbar_D03_457364 | T/G | 0.08 | 6.36 |
| Gbar_D03_372101 | G/A | 0.07 | 6.03 | Gbar_D03_427960 | G/A | 0.07 | 6.69 | Gbar_D03_457555 | T/A | 0.08 | 6.58 |
| Gbar_D03_372618 | C/T | 0.08 | 6.84 | Gbar_D03_428631 | T/A | 0.08 | 6.61 | Gbar_D03_457649 | T/G | 0.08 | 7.05 |
| Gbar_D03_373236 | C/T | 0.08 | 6.68 | Gbar_D03_428867 | G/C | 0.08 | 6.38 | Gbar_D03_457899 | T/C | 0.08 | 6.64 |
| Gbar_D03_374672 | A/G | 0.08 | 6.14 | Gbar_D03_429255 | T/G | 0.07 | 6.50 | Gbar_D03_457905 | C/T | 0.08 | 6.23 |
| Gbar_D03_377608 | T/C | 0.08 | 6.57 | Gbar_D03_429289 | C/T | 0.07 | 6.68 | Gbar_D03_457921 | T/C | 0.07 | 6.43 |
| Gbar_D03_377835 | T/C | 0.08 | 6.53 | Gbar_D03_429383 | A/G | 0.08 | 6.63 | Gbar_D03_458018 | A/G | 0.08 | 7.24 |
| Gbar_D03_378142 | G/A | 0.08 | 7.21 | Gbar_D03_430264 | A/C | 0.08 | 7.11 | Gbar_D03_458610 | T/G | 0.08 | 6.53 |
| Gbar_D03_378662 | T/C | 0.07 | 6.07 | Gbar_D03_430991 | A/G | 0.07 | 6.14 | Gbar_D03_458775 | G/T | 0.08 | 6.27 |
| Gbar_D03_379122 | T/G | 0.07 | 6.54 | Gbar_D03_431084 | C/G | 0.08 | 6.89 | Gbar_D03_459639 | G/C | 0.08 | 6.99 |
| Gbar_D03_379635 | C/T | 0.08 | 6.11 | Gbar_D03_431163 | T/C | 0.08 | 6.21 | Gbar_D03_459837 | G/A | 0.08 | 6.65 |
| Gbar_D03_379739 | G/A | 0.08 | 6.85 | Gbar_D03_431380 | C/T | 0.08 | 6.57 | Gbar_D03_461284 | A/T | 0.07 | 6.90 |
| Gbar_D03_380046 | C/T | 0.08 | 6.26 | Gbar_D03_431389 | T/C | 0.08 | 6.07 | Gbar_D03_462175 | C/A | 0.08 | 6.70 |
| Gbar_D03_380070 | C/A | 0.08 | 6.29 | Gbar_D03_431689 | T/C | 0.08 | 6.98 | Gbar_D03_462259 | A/G | 0.08 | 6.68 |
| Gbar_D03_380281 | A/C | 0.08 | 6.67 | Gbar_D03_431887 | T/A | 0.07 | 6.07 | Gbar_D03_462396 | T/A | 0.07 | 6.37 |
| Gbar_D03_380335 | T/A | 0.08 | 6.29 | Gbar_D03_431958 | A/G | 0.07 | 6.12 | Gbar_D03_462575 | G/A | 0.08 | 7.28 |
| Gbar_D03_380949 | C/G | 0.08 | 6.50 | Gbar_D03_432032 | T/C | 0.08 | 6.93 | Gbar_D03_462735 | G/A | 0.08 | 6.88 |
| Gbar_D03_381143 | C/T | 0.07 | 6.22 | Gbar_D03_432048 | A/G | 0.08 | 6.86 | Gbar_D03_462750 | C/T | 0.08 | 6.49 |
| Gbar_D03_381277 | G/T | 0.07 | 6.10 | Gbar_D03_432073 | A/G | 0.08 | 6.71 | Gbar_D03_462782 | A/G | 0.08 | 6.83 |
| Gbar_D03_381403 | C/A | 0.08 | 6.33 | Gbar_D03_432198 | C/T | 0.08 | 6.95 | Gbar_D03_462898 | C/T | 0.08 | 7.18 |
| Gbar_D03_381478 | T/G | 0.08 | 6.82 | Gbar_D03_432338 | A/C | 0.08 | 6.45 | Gbar_D03_462912 | C/G | 0.08 | 6.88 |
| Gbar_D03_381665 | T/G | 0.08 | 6.14 | Gbar_D03_432339 | C/G | 0.08 | 6.21 | Gbar_D03_463030 | G/A | 0.07 | 7.81 |
| Gbar_D03_381736 | A/C | 0.08 | 6.07 | Gbar_D03_432703 | A/C | 0.08 | 7.94 | Gbar_D03_463042 | T/A | 0.07 | 7.72 |
| Gbar_D03_382233 | G/A | 0.08 | 6.77 | Gbar_D03_433030 | C/T | 0.08 | 6.53 | Gbar_D03_463069 | T/G | 0.08 | 7.15 |
| Gbar_D03_382256 | G/A | 0.08 | 7.09 | Gbar_D03_433567 | A/T | 0.08 | 6.32 | Gbar_D03_463234 | A/G | 0.08 | 6.94 |
| Gbar_D03_382399 | A/G | 0.08 | 6.14 | Gbar_D03_434058 | T/G | 0.08 | 7.31 | Gbar_D03_463271 | C/A | 0.08 | 6.64 |
| Gbar_D03_382485 | A/T | 0.08 | 6.73 | Gbar_D03_434175 | G/A | 0.08 | 6.21 | Gbar_D03_463326 | T/C | 0.08 | 6.99 |
| Gbar_D03_382861 | T/C | 0.07 | 6.73 | Gbar_D03_434233 | G/A | 0.08 | 6.41 | Gbar_D03_463364 | A/C | 0.08 | 6.88 |
| Gbar_D03_382874 | T/C | 0.07 | 6.91 | Gbar_D03_434363 | A/G | 0.08 | 6.18 | Gbar_D03_463611 | T/C | 0.07 | 6.22 |
| Gbar_D03_383853 | C/T | 0.08 | 6.30 | Gbar_D03_434386 | G/A | 0.08 | 6.32 | Gbar_D03_463649 | C/T | 0.08 | 6.34 |
| Gbar_D03_383926 | G/A | 0.08 | 7.04 | Gbar_D03_434425 | A/G | 0.08 | 6.63 | Gbar_D03_463691 | A/C | 0.08 | 7.36 |
| Gbar_D03_384502 | C/A | 0.08 | 6.24 | Gbar_D03_434460 | T/C | 0.08 | 6.11 | Gbar_D03_463991 | T/C | 0.08 | 6.40 |
| Gbar_D03_384657 | A/G | 0.08 | 6.38 | Gbar_D03_434671 | C/T | 0.08 | 6.63 | Gbar_D03_464328 | T/G | 0.08 | 6.62 |
| Gbar_D03_385646 | A/T | 0.08 | 6.10 | Gbar_D03_434694 | A/T | 0.08 | 6.27 | Gbar_D03_465054 | T/C | 0.07 | 6.09 |
| Gbar_D03_385751 | A/G | 0.08 | 6.80 | Gbar_D03_435100 | C/T | 0.07 | 6.86 | Gbar_D03_465332 | C/T | 0.08 | 7.04 |
| Gbar_D03_385959 | T/G | 0.08 | 7.03 | Gbar_D03_435183 | C/T | 0.08 | 6.07 | Gbar_D03_465413 | C/G | 0.08 | 6.79 |
| Gbar_D03_386126 | T/G | 0.08 | 6.46 | Gbar_D03_435277 | G/C | 0.08 | 6.27 | Gbar_D03_465477 | G/T | 0.08 | 6.58 |
| Gbar_D03_386546 | T/C | 0.08 | 6.71 | Gbar_D03_435301 | A/T | 0.08 | 6.46 | Gbar_D03_466275 | G/A | 0.08 | 6.71 |
| Gbar_D03_388400 | T/A | 0.08 | 6.53 | Gbar_D03_435671 | G/T | 0.08 | 6.69 | Gbar_D03_467959 | A/T | 0.08 | 6.64 |
| Gbar_D03_388455 | T/C | 0.08 | 7.29 | Gbar_D03_437353 | G/C | 0.08 | 6.52 | Gbar_D03_468281 | T/G | 0.08 | 7.19 |
| Gbar_D03_388476 | A/T | 0.08 | 6.03 | Gbar_D03_437356 | C/T | 0.08 | 6.45 | Gbar_D03_468539 | A/G | 0.07 | 6.30 |
| Gbar_D03_389413 | T/C | 0.08 | 6.06 | Gbar_D03_437487 | A/G | 0.08 | 7.72 | Gbar_D03_469034 | G/C | 0.07 | 7.24 |
| Gbar_D03_389479 | A/G | 0.08 | 6.35 | Gbar_D03_437532 | C/T | 0.08 | 7.36 | Gbar_D03_469828 | C/T | 0.08 | 6.66 |
| Gbar_D03_389547 | T/C | 0.07 | 6.32 | Gbar_D03_437857 | C/A | 0.08 | 6.28 | Gbar_D03_470216 | T/C | 0.08 | 6.27 |
| Gbar_D03_389739 | C/G | 0.08 | 6.14 | Gbar_D03_437877 | C/T | 0.08 | 6.89 | Gbar_D03_470365 | A/G | 0.08 | 6.59 |
| Gbar_D03_392094 | T/A | 0.08 | 6.88 | Gbar_D03_439082 | C/T | 0.08 | 6.69 | Gbar_D03_470969 | T/C | 0.08 | 6.11 |
| Gbar_D03_396757 | G/T | 0.08 | 6.31 | Gbar_D03_439089 | G/A | 0.08 | 6.71 | Gbar_D03_471004 | T/C | 0.08 | 6.13 |
| Gbar_D03_396864 | T/C | 0.08 | 6.64 | Gbar_D03_439090 | T/A | 0.08 | 6.63 | Gbar_D03_471549 | T/C | 0.08 | 6.29 |
| Gbar_D03_397540 | T/C | 0.08 | 6.10 | Gbar_D03_439136 | A/G | 0.08 | 6.48 | Gbar_D03_471597 | A/G | 0.07 | 6.06 |
| Gbar_D03_400518 | C/T | 0.08 | 6.30 | Gbar_D03_439359 | T/C | 0.08 | 6.75 | Gbar_D03_471728 | A/G | 0.08 | 6.89 |
| Gbar_D03_401365 | G/A | 0.08 | 6.67 | Gbar_D03_439612 | G/A | 0.08 | 7.12 | Gbar_D03_471731 | A/G | 0.08 | 6.66 |
| Gbar_D03_401374 | A/G | 0.07 | 6.60 | Gbar_D03_439614 | A/G | 0.08 | 7.26 | Gbar_D03_472231 | C/T | 0.08 | 6.81 |
| Gbar_D03_401592 | T/C | 0.08 | 7.05 | Gbar_D03_439744 | A/G | 0.08 | 6.79 | Gbar_D03_473086 | C/T | 0.07 | 6.41 |
| Gbar_D03_401895 | G/A | 0.08 | 6.03 | Gbar_D03_439883 | A/G | 0.08 | 6.60 | Gbar_D03_473099 | C/T | 0.08 | 6.67 |
| Gbar_D03_402831 | C/T | 0.08 | 6.83 | Gbar_D03_440489 | T/C | 0.07 | 6.48 | Gbar_D03_474031 | C/T | 0.07 | 6.05 |
| Gbar_D03_402897 | G/A | 0.08 | 6.89 | Gbar_D03_441607 | G/T | 0.08 | 7.02 | Gbar_D03_474407 | T/C | 0.08 | 6.43 |
| Gbar_D03_403327 | T/C | 0.08 | 6.65 | Gbar_D03_442128 | C/T | 0.08 | 6.16 | Gbar_D03_474411 | C/T | 0.08 | 6.75 |
| Gbar_D03_403389 | G/C | 0.08 | 6.28 | Gbar_D03_442279 | G/A | 0.08 | 6.82 | Gbar_D03_474425 | T/C | 0.08 | 6.49 |
| Gbar_D03_403792 | A/C | 0.08 | 6.84 | Gbar_D03_443612 | T/A | 0.07 | 6.54 | Gbar_D03_474518 | T/A | 0.08 | 6.59 |
| Gbar_D03_405154 | G/T | 0.08 | 6.90 | Gbar_D03_443813 | G/A | 0.08 | 7.05 | Gbar_D03_474723 | G/T | 0.08 | 6.26 |
| Gbar_D03_405198 | G/A | 0.08 | 6.36 | Gbar_D03_443832 | T/C | 0.08 | 6.90 | Gbar_D03_475216 | C/T | 0.08 | 7.28 |
| Gbar_D03_405384 | G/T | 0.08 | 6.29 | Gbar_D03_445097 | C/T | 0.08 | 6.39 | Gbar_D03_475398 | G/A | 0.08 | 6.68 |
| Gbar_D03_405700 | A/T | 0.08 | 6.85 | Gbar_D03_446345 | C/T | 0.08 | 6.56 | Gbar_D03_475454 | C/A | 0.08 | 6.52 |
| Gbar_D03_405726 | A/G | 0.08 | 7.03 | Gbar_D03_446437 | G/A | 0.08 | 6.02 | Gbar_D03_476094 | A/G | 0.07 | 6.46 |
| Gbar_D03_405730 | A/T | 0.08 | 6.99 | Gbar_D03_446603 | C/T | 0.08 | 6.47 | Gbar_D03_476170 | G/T | 0.08 | 6.87 |
| Gbar_D03_406053 | G/A | 0.08 | 7.38 | Gbar_D03_446682 | C/T | 0.08 | 6.96 | Gbar_D03_477311 | T/A | 0.08 | 6.65 |
| Gbar_D03_406139 | T/C | 0.08 | 7.30 | Gbar_D03_446956 | T/G | 0.08 | 6.83 | Gbar_D03_477449 | T/G | 0.07 | 6.40 |
| Gbar_D03_406564 | C/T | 0.08 | 6.68 | Gbar_D03_447043 | G/C | 0.07 | 6.19 | Gbar_D03_477691 | C/A | 0.07 | 6.09 |
| Gbar_D03_406637 | C/A | 0.08 | 7.02 | Gbar_D03_447194 | C/T | 0.08 | 6.07 | Gbar_D03_478387 | C/T | 0.08 | 6.42 |
| Gbar_D03_408929 | G/A | 0.08 | 6.15 | Gbar_D03_447239 | G/A | 0.08 | 7.12 | Gbar_D03_478691 | G/T | 0.08 | 6.61 |
| Gbar_D03_409395 | A/T | 0.08 | 6.60 | Gbar_D03_447613 | T/A | 0.08 | 6.83 | Gbar_D03_478840 | T/C | 0.08 | 6.22 |
| Gbar_D03_409507 | G/A | 0.08 | 6.64 | Gbar_D03_447811 | C/T | 0.07 | 6.58 | Gbar_D03_478976 | T/C | 0.08 | 7.07 |
| Gbar_D03_409648 | T/G | 0.08 | 6.61 | Gbar_D03_447822 | A/G | 0.07 | 6.16 | Gbar_D03_479127 | G/T | 0.07 | 6.03 |
| Gbar_D03_409945 | C/T | 0.08 | 7.33 | Gbar_D03_447988 | T/C | 0.08 | 6.36 | Gbar_D03_479959 | C/T | 0.07 | 6.22 |
| Gbar_D03_410090 | C/T | 0.08 | 6.75 | Gbar_D03_448346 | C/G | 0.08 | 7.19 | Gbar_D03_479982 | G/A | 0.07 | 6.26 |
| Gbar_D03_410594 | G/A | 0.08 | 6.04 | Gbar_D03_449116 | G/A | 0.07 | 6.13 | Gbar_D03_480243 | T/C | 0.08 | 6.19 |
| Gbar_D03_410928 | G/A | 0.08 | 6.97 | Gbar_D03_449644 | T/G | 0.08 | 7.01 | Gbar_D03_480382 | A/G | 0.08 | 6.45 |
| Gbar_D03_411252 | T/G | 0.08 | 6.38 | Gbar_D03_449732 | A/T | 0.08 | 6.04 | Gbar_D03_480663 | C/T | 0.08 | 6.76 |
| Gbar_D03_411418 | A/G | 0.08 | 6.59 | Gbar_D03_449901 | G/T | 0.08 | 6.39 | Gbar_D03_480666 | T/C | 0.08 | 6.52 |
| Gbar_D03_411439 | T/C | 0.08 | 6.66 | Gbar_D03_449954 | A/G | 0.08 | 6.56 | Gbar_D03_481380 | G/A | 0.08 | 6.56 |
| Gbar_D03_411532 | A/G | 0.08 | 6.04 | Gbar_D03_450193 | A/G | 0.07 | 6.44 | Gbar_D03_481529 | G/A | 0.08 | 6.89 |
| Gbar_D03_411581 | G/A | 0.08 | 6.15 | Gbar_D03_450582 | A/G | 0.08 | 6.83 | Gbar_D03_481659 | C/T | 0.08 | 6.19 |
| Gbar_D03_411885 | C/T | 0.07 | 6.43 | Gbar_D03_450697 | A/T | 0.07 | 6.64 | Gbar_D03_481987 | C/T | 0.08 | 6.51 |
| Gbar_D03_412682 | C/A | 0.07 | 6.78 | Gbar_D03_451008 | T/C | 0.08 | 6.92 | Gbar_D03_482747 | A/G | 0.08 | 7.01 |
| Gbar_D03_412737 | G/A | 0.08 | 7.13 | Gbar_D03_451901 | G/A | 0.08 | 6.97 | Gbar_D03_482860 | C/T | 0.08 | 6.87 |
| Gbar_D03_412749 | A/C | 0.08 | 6.69 | Gbar_D03_452383 | G/A | 0.08 | 7.56 | Gbar_D03_482946 | G/A | 0.08 | 6.63 |
| Gbar_D03_413017 | T/A | 0.08 | 7.01 | Gbar_D03_453697 | C/G | 0.08 | 6.92 | Gbar_D03_482995 | C/T | 0.08 | 6.86 |
| Gbar_D03_413379 | T/C | 0.08 | 6.26 | Gbar_D03_453723 | G/T | 0.07 | 6.17 | Gbar_D03_489394 | T/C | 0.08 | 7.21 |
| Gbar_D03_413952 | C/T | 0.07 | 6.18 | Gbar_D03_453752 | G/A | 0.07 | 6.75 | Gbar_D03_489419 | C/T | 0.08 | 6.72 |
| Gbar_D03_414289 | T/A | 0.08 | 6.75 | Gbar_D03_453779 | C/T | 0.08 | 6.42 | Gbar_D03_489609 | A/G | 0.08 | 6.32 |
| Gbar_D03_414431 | T/C | 0.08 | 6.07 | Gbar_D03_453781 | T/A | 0.07 | 6.39 | Gbar_D03_490436 | G/C | 0.08 | 6.35 |
| Gbar_D03_416021 | G/A | 0.07 | 6.54 | Gbar_D03_453833 | A/G | 0.08 | 6.79 | Gbar_D03_490683 | G/A | 0.08 | 6.53 |
| Gbar_D03_416025 | C/T | 0.07 | 6.66 | Gbar_D03_453867 | T/C | 0.08 | 6.69 | Gbar_D03_490974 | A/T | 0.08 | 6.14 |
| Gbar_D03_416026 | A/G | 0.07 | 6.79 | Gbar_D03_453869 | G/A | 0.08 | 6.90 | Gbar_D03_491146 | T/C | 0.08 | 6.17 |
| Gbar_D03_416081 | T/C | 0.07 | 6.13 | Gbar_D03_453971 | C/A | 0.08 | 6.52 | Gbar_D03_491506 | C/T | 0.08 | 6.75 |
| Gbar_D03_417385 | G/C | 0.08 | 6.25 | Gbar_D03_454071 | G/A | 0.07 | 6.17 | Gbar_D03_491514 | A/T | 0.08 | 6.69 |
| Gbar_D03_417844 | G/A | 0.07 | 6.67 | Gbar_D03_454111 | A/G | 0.08 | 6.43 | Gbar_D03_491757 | C/A | 0.07 | 6.29 |
| Gbar_D03_418695 | T/C | 0.08 | 6.56 | Gbar_D03_454158 | T/C | 0.08 | 6.86 | Gbar_D03_491764 | G/A | 0.08 | 6.32 |
| Gbar_D03_418726 | A/G | 0.08 | 6.64 | Gbar_D03_454523 | G/A | 0.08 | 6.97 | Gbar_D03_492127 | G/A | 0.08 | 6.69 |
| Gbar_D03_419056 | G/T | 0.08 | 6.84 | Gbar_D03_454549 | G/T | 0.08 | 6.29 | Gbar_D03_492496 | G/A | 0.08 | 6.11 |
| Gbar_D03_419441 | C/A | 0.08 | 6.74 | Gbar_D03_454777 | C/A | 0.08 | 6.38 | Gbar_D03_492515 | T/C | 0.08 | 6.06 |
| Gbar_D03_419471 | A/G | 0.07 | 6.26 | Gbar_D03_455317 | C/T | 0.08 | 6.13 | Gbar_D03_492543 | C/T | 0.08 | 6.22 |
| Gbar_D03_420166 | C/T | 0.08 | 6.48 | Gbar_D03_455546 | T/C | 0.09 | 6.12 | Gbar_D03_493428 | G/T | 0.08 | 6.90 |
| Gbar_D03_420689 | C/T | 0.08 | 6.05 | Gbar_D03_456156 | T/A | 0.08 | 6.20 | Gbar_D03_493543 | G/T | 0.07 | 6.44 |
| Gbar_D03_420773 | G/A | 0.08 | 6.31 | Gbar_D03_456615 | T/C | 0.08 | 6.53 | Gbar_D03_493656 | G/C | 0.08 | 7.17 |
| Gbar_D03_422415 | G/T | 0.08 | 6.39 | Gbar_D03_456764 | A/C | 0.08 | 7.00 | Gbar_D03_493884 | G/A | 0.08 | 6.85 |
| Gbar_D03_423244 | T/G | 0.07 | 6.89 | Gbar_D03_456785 | G/T | 0.07 | 6.10 | Gbar_D03_494172 | A/G | 0.08 | 6.92 |
| Gbar_D03_423451 | T/G | 0.07 | 6.48 | Gbar_D03_456854 | T/A | 0.07 | 6.27 | Gbar_D03_494449 | C/A | 0.08 | 6.82 |

**Table S2.** List of the associated SNPs identified in different datasets through GWAS for *FW* resistance in Sea Island cotton. (Continued)

| SNP | Ref/Alt | MAF | -log (p-value) | SNP | Ref/Alt | MAF | -log (p-value) | SNP | Ref/Alt | MAF | -log (p-value) |
| --- | --- | --- | --- | --- | --- | --- | --- | --- | --- | --- | --- |
| **2016** |  |  |  | Gbar_D03_519276 | A/G | 0.08 | 6.58 | Gbar_D03_550970 | A/G | 0.08 | 6.14 |
| Gbar_D03_494851 | T/C | 0.08 | 6.88 | Gbar_D03_519537 | G/T | 0.08 | 6.69 | Gbar_D03_551060 | C/T | 0.08 | 6.69 |
| Gbar_D03_495058 | C/G | 0.08 | 6.34 | Gbar_D03_519585 | T/G | 0.08 | 6.25 | Gbar_D03_551093 | G/T | 0.08 | 7.07 |
| Gbar_D03_495334 | T/C | 0.08 | 6.85 | Gbar_D03_519680 | C/G | 0.07 | 6.70 | Gbar_D03_551243 | T/C | 0.08 | 6.61 |
| Gbar_D03_496070 | C/T | 0.08 | 6.17 | Gbar_D03_519697 | T/C | 0.07 | 6.42 | Gbar_D03_552199 | A/T | 0.08 | 7.42 |
| Gbar_D03_496135 | A/G | 0.07 | 6.44 | Gbar_D03_519700 | T/C | 0.07 | 6.42 | Gbar_D03_553317 | A/T | 0.08 | 6.92 |
| Gbar_D03_496307 | T/C | 0.08 | 6.80 | Gbar_D03_519733 | A/G | 0.07 | 6.27 | Gbar_D03_553400 | G/A | 0.08 | 6.49 |
| Gbar_D03_496437 | G/T | 0.08 | 7.13 | Gbar_D03_519752 | A/G | 0.08 | 6.90 | Gbar_D03_553621 | A/G | 0.08 | 6.27 |
| Gbar_D03_496666 | G/A | 0.08 | 6.62 | Gbar_D03_519828 | C/T | 0.08 | 7.00 | Gbar_D03_553708 | G/A | 0.08 | 6.41 |
| Gbar_D03_496839 | A/T | 0.08 | 6.65 | Gbar_D03_519838 | A/G | 0.08 | 6.61 | Gbar_D03_554990 | G/A | 0.07 | 6.63 |
| Gbar_D03_496847 | A/G | 0.07 | 6.24 | Gbar_D03_519855 | C/T | 0.08 | 6.48 | Gbar_D03_555179 | T/C | 0.07 | 6.47 |
| Gbar_D03_497738 | A/C | 0.07 | 6.37 | Gbar_D03_519894 | T/C | 0.08 | 7.08 | Gbar_D03_555523 | G/A | 0.08 | 6.63 |
| Gbar_D03_497951 | G/A | 0.08 | 7.32 | Gbar_D03_519902 | G/A | 0.08 | 6.99 | Gbar_D03_555903 | A/G | 0.07 | 6.65 |
| Gbar_D03_498371 | T/A | 0.08 | 6.86 | Gbar_D03_519931 | C/T | 0.08 | 7.39 | Gbar_D03_556131 | C/T | 0.08 | 6.85 |
| Gbar_D03_498674 | T/G | 0.07 | 6.13 | Gbar_D03_519978 | T/C | 0.07 | 6.21 | Gbar_D03_556309 | T/C | 0.08 | 6.89 |
| Gbar_D03_498852 | T/A | 0.07 | 6.01 | Gbar_D03_520101 | C/T | 0.08 | 6.79 | Gbar_D03_556375 | T/C | 0.08 | 6.94 |
| Gbar_D03_500198 | G/A | 0.08 | 6.50 | Gbar_D03_520350 | C/T | 0.08 | 6.57 | Gbar_D03_556487 | A/T | 0.08 | 7.00 |
| Gbar_D03_500515 | A/G | 0.08 | 6.59 | Gbar_D03_520368 | C/A | 0.08 | 6.68 | Gbar_D03_556612 | A/G | 0.08 | 6.39 |
| Gbar_D03_500568 | A/G | 0.08 | 6.19 | Gbar_D03_520624 | T/A | 0.08 | 7.21 | Gbar_D03_556944 | T/A | 0.07 | 6.11 |
| Gbar_D03_500815 | C/T | 0.08 | 6.53 | Gbar_D03_520660 | C/T | 0.08 | 7.28 | Gbar_D03_556956 | G/A | 0.07 | 6.05 |
| Gbar_D03_501474 | A/C | 0.08 | 6.91 | Gbar_D03_521083 | G/A | 0.08 | 6.57 | Gbar_D03_556972 | G/A | 0.07 | 6.00 |
| Gbar_D03_501600 | C/T | 0.08 | 6.80 | Gbar_D03_521153 | A/C | 0.08 | 6.48 | Gbar_D03_557193 | G/T | 0.08 | 7.54 |
| Gbar_D03_501668 | C/T | 0.08 | 6.21 | Gbar_D03_523494 | C/A | 0.07 | 6.51 | Gbar_D03_557269 | A/G | 0.07 | 6.92 |
| Gbar_D03_501713 | T/C | 0.08 | 6.47 | Gbar_D03_523860 | C/T | 0.08 | 7.03 | Gbar_D03_557938 | G/T | 0.07 | 6.13 |
| Gbar_D03_501715 | C/A | 0.08 | 6.71 | Gbar_D03_523865 | C/T | 0.08 | 7.23 | Gbar_D03_558287 | C/T | 0.08 | 6.72 |
| Gbar_D03_501946 | T/C | 0.08 | 6.23 | Gbar_D03_524047 | C/T | 0.08 | 6.60 | Gbar_D03_558772 | A/T | 0.08 | 6.26 |
| Gbar_D03_502048 | C/T | 0.08 | 6.68 | Gbar_D03_524133 | T/C | 0.08 | 7.24 | Gbar_D03_560351 | G/C | 0.08 | 7.26 |
| Gbar_D03_502197 | T/C | 0.08 | 6.71 | Gbar_D03_524400 | T/C | 0.07 | 6.20 | Gbar_D03_561004 | T/A | 0.08 | 7.04 |
| Gbar_D03_502320 | A/G | 0.07 | 6.13 | Gbar_D03_525581 | G/A | 0.08 | 6.75 | Gbar_D03_561294 | G/A | 0.07 | 6.46 |
| Gbar_D03_502354 | G/T | 0.08 | 6.22 | Gbar_D03_525876 | A/G | 0.08 | 6.43 | Gbar_D03_561297 | C/T | 0.07 | 6.60 |
| Gbar_D03_502804 | A/G | 0.07 | 7.11 | Gbar_D03_526356 | A/G | 0.08 | 6.37 | Gbar_D03_561330 | C/T | 0.08 | 6.84 |
| Gbar_D03_502823 | C/T | 0.07 | 6.56 | Gbar_D03_526457 | T/C | 0.08 | 6.52 | Gbar_D03_565696 | G/T | 0.08 | 6.07 |
| Gbar_D03_503008 | A/G | 0.08 | 6.23 | Gbar_D03_526472 | A/G | 0.08 | 6.82 | Gbar_D03_565718 | T/G | 0.08 | 6.53 |
| Gbar_D03_503017 | T/C | 0.08 | 6.19 | Gbar_D03_527752 | G/A | 0.08 | 6.16 | Gbar_D03_565872 | T/G | 0.08 | 6.95 |
| Gbar_D03_503066 | C/T | 0.07 | 6.17 | Gbar_D03_527972 | C/T | 0.08 | 6.62 | Gbar_D03_566189 | T/A | 0.07 | 7.25 |
| Gbar_D03_503118 | T/C | 0.08 | 6.80 | Gbar_D03_528020 | C/T | 0.08 | 6.74 | Gbar_D03_566277 | A/G | 0.07 | 6.54 |
| Gbar_D03_503165 | T/C | 0.08 | 7.00 | Gbar_D03_528041 | T/C | 0.07 | 6.58 | Gbar_D03_567217 | G/A | 0.08 | 6.41 |
| Gbar_D03_503175 | T/A | 0.08 | 6.87 | Gbar_D03_528046 | C/T | 0.07 | 6.39 | Gbar_D03_568405 | C/T | 0.07 | 6.36 |
| Gbar_D03_503307 | C/A | 0.08 | 6.73 | Gbar_D03_528052 | T/C | 0.07 | 6.51 | Gbar_D03_568757 | T/C | 0.08 | 6.80 |
| Gbar_D03_503316 | T/C | 0.08 | 6.23 | Gbar_D03_528129 | A/C | 0.07 | 6.66 | Gbar_D03_568803 | C/T | 0.08 | 6.21 |
| Gbar_D03_503374 | C/T | 0.08 | 6.43 | Gbar_D03_528449 | C/A | 0.08 | 6.04 | Gbar_D03_569237 | G/A | 0.08 | 6.62 |
| Gbar_D03_503381 | G/T | 0.08 | 6.47 | Gbar_D03_528807 | A/G | 0.08 | 6.56 | Gbar_D03_569310 | C/T | 0.08 | 6.82 |
| Gbar_D03_503512 | A/G | 0.08 | 6.36 | Gbar_D03_530107 | G/A | 0.08 | 6.62 | Gbar_D03_570302 | G/A | 0.08 | 6.53 |
| Gbar_D03_503636 | T/C | 0.08 | 6.32 | Gbar_D03_530338 | T/C | 0.08 | 6.52 | Gbar_D03_570360 | C/T | 0.08 | 6.81 |
| Gbar_D03_503662 | T/C | 0.10 | 6.34 | Gbar_D03_530744 | A/C | 0.07 | 6.14 | Gbar_D03_570401 | G/A | 0.08 | 6.56 |
| Gbar_D03_503765 | T/C | 0.07 | 6.13 | Gbar_D03_530851 | A/G | 0.08 | 6.91 | Gbar_D03_571081 | A/C | 0.08 | 6.78 |
| Gbar_D03_503973 | G/A | 0.07 | 6.44 | Gbar_D03_531756 | G/T | 0.08 | 6.97 | Gbar_D03_571117 | G/A | 0.07 | 6.49 |
| Gbar_D03_504000 | T/G | 0.11 | 6.22 | Gbar_D03_532612 | T/G | 0.08 | 7.08 | Gbar_D03_571546 | A/G | 0.07 | 6.06 |
| Gbar_D03_504030 | C/T | 0.08 | 6.30 | Gbar_D03_533744 | T/C | 0.08 | 6.71 | Gbar_D03_572307 | G/A | 0.08 | 6.40 |
| Gbar_D03_504141 | A/G | 0.08 | 6.73 | Gbar_D03_534225 | G/A | 0.08 | 6.07 | Gbar_D03_572820 | G/A | 0.08 | 6.31 |
| Gbar_D03_504389 | C/T | 0.07 | 6.04 | Gbar_D03_534462 | G/C | 0.08 | 6.90 | Gbar_D03_572846 | T/A | 0.08 | 6.37 |
| Gbar_D03_504411 | G/A | 0.08 | 6.77 | Gbar_D03_534685 | G/A | 0.07 | 6.31 | Gbar_D03_574251 | G/A | 0.08 | 6.72 |
| Gbar_D03_504566 | T/C | 0.08 | 6.17 | Gbar_D03_535236 | T/C | 0.07 | 6.18 | Gbar_D03_574319 | T/C | 0.08 | 6.88 |
| Gbar_D03_504575 | G/A | 0.08 | 6.13 | Gbar_D03_535883 | T/A | 0.08 | 7.80 | Gbar_D03_574349 | G/A | 0.08 | 6.68 |
| Gbar_D03_504576 | C/T | 0.08 | 6.98 | Gbar_D03_536094 | A/G | 0.07 | 6.43 | Gbar_D03_574485 | T/A | 0.08 | 7.07 |
| Gbar_D03_504805 | T/C | 0.08 | 7.23 | Gbar_D03_536096 | G/A | 0.07 | 6.25 | Gbar_D03_574526 | A/G | 0.08 | 6.77 |
| Gbar_D03_504998 | T/C | 0.08 | 6.51 | Gbar_D03_536787 | G/A | 0.08 | 6.45 | Gbar_D03_575215 | T/C | 0.07 | 6.05 |
| Gbar_D03_505033 | A/G | 0.08 | 6.81 | Gbar_D03_536850 | A/T | 0.08 | 6.99 | Gbar_D03_576234 | G/A | 0.08 | 6.67 |
| Gbar_D03_505069 | G/A | 0.07 | 6.97 | Gbar_D03_536889 | A/T | 0.08 | 6.01 | Gbar_D03_576262 | C/T | 0.08 | 6.74 |
| Gbar_D03_505306 | T/A | 0.07 | 6.01 | Gbar_D03_537009 | T/C | 0.08 | 6.44 | Gbar_D03_577309 | A/G | 0.08 | 6.70 |
| Gbar_D03_505828 | C/A | 0.08 | 6.02 | Gbar_D03_537091 | G/T | 0.08 | 6.87 | Gbar_D03_577764 | G/A | 0.07 | 6.21 |
| Gbar_D03_505971 | C/T | 0.07 | 6.01 | Gbar_D03_537524 | T/C | 0.08 | 6.68 | Gbar_D03_580128 | T/C | 0.08 | 6.34 |
| Gbar_D03_506076 | T/C | 0.08 | 6.53 | Gbar_D03_537961 | C/T | 0.08 | 6.07 | Gbar_D03_580269 | A/G | 0.08 | 7.01 |
| Gbar_D03_506177 | C/T | 0.08 | 6.61 | Gbar_D03_538074 | T/C | 0.08 | 6.51 | Gbar_D03_580780 | T/C | 0.08 | 6.47 |
| Gbar_D03_506188 | A/C | 0.08 | 6.76 | Gbar_D03_538327 | C/T | 0.08 | 6.58 | Gbar_D03_580781 | G/A | 0.07 | 6.56 |
| Gbar_D03_506208 | C/G | 0.08 | 6.65 | Gbar_D03_538413 | C/T | 0.08 | 6.68 | Gbar_D03_581198 | T/G | 0.07 | 6.44 |
| Gbar_D03_506264 | C/T | 0.07 | 6.92 | Gbar_D03_538597 | T/C | 0.08 | 6.21 | Gbar_D03_581868 | C/G | 0.08 | 6.50 |
| Gbar_D03_506275 | A/T | 0.07 | 6.81 | Gbar_D03_540155 | A/T | 0.07 | 6.26 | Gbar_D03_582226 | T/C | 0.08 | 6.93 |
| Gbar_D03_506335 | A/G | 0.07 | 6.61 | Gbar_D03_540171 | A/G | 0.07 | 6.07 | Gbar_D03_582312 | C/T | 0.07 | 6.19 |
| Gbar_D03_506353 | A/T | 0.07 | 6.16 | Gbar_D03_540330 | A/G | 0.07 | 6.07 | Gbar_D03_582513 | A/T | 0.08 | 7.13 |
| Gbar_D03_506357 | A/G | 0.08 | 6.66 | Gbar_D03_540437 | T/A | 0.07 | 6.32 | Gbar_D03_583841 | A/G | 0.08 | 6.60 |
| Gbar_D03_506428 | A/C | 0.07 | 6.59 | Gbar_D03_540438 | T/C | 0.07 | 6.51 | Gbar_D03_586180 | G/T | 0.08 | 6.25 |
| Gbar_D03_506443 | C/T | 0.07 | 6.42 | Gbar_D03_541604 | C/T | 0.08 | 7.01 | Gbar_D03_586207 | A/G | 0.07 | 6.33 |
| Gbar_D03_506471 | G/A | 0.07 | 6.68 | Gbar_D03_541634 | T/A | 0.08 | 6.93 | Gbar_D03_586239 | A/G | 0.08 | 6.60 |
| Gbar_D03_506491 | C/T | 0.08 | 6.91 | Gbar_D03_541711 | T/C | 0.07 | 6.95 | Gbar_D03_586240 | C/T | 0.08 | 6.27 |
| Gbar_D03_506522 | A/G | 0.07 | 6.95 | Gbar_D03_541801 | C/T | 0.08 | 6.44 | Gbar_D03_586718 | T/A | 0.08 | 6.60 |
| Gbar_D03_506560 | T/C | 0.07 | 6.02 | Gbar_D03_541848 | T/A | 0.07 | 6.67 | Gbar_D03_587654 | C/T | 0.08 | 6.64 |
| Gbar_D03_506634 | G/A | 0.07 | 6.07 | Gbar_D03_542054 | C/A | 0.07 | 6.50 | Gbar_D03_587662 | G/A | 0.08 | 6.50 |
| Gbar_D03_506689 | T/C | 0.07 | 6.13 | Gbar_D03_542293 | A/G | 0.08 | 7.04 | Gbar_D03_587674 | G/A | 0.08 | 6.24 |
| Gbar_D03_506792 | A/C | 0.08 | 7.16 | Gbar_D03_542441 | A/G | 0.08 | 7.10 | Gbar_D03_587701 | A/T | 0.08 | 6.33 |
| Gbar_D03_506900 | C/T | 0.07 | 6.41 | Gbar_D03_542785 | A/C | 0.08 | 6.64 | Gbar_D03_587907 | T/A | 0.07 | 6.32 |
| Gbar_D03_507278 | A/G | 0.10 | 7.00 | Gbar_D03_542796 | A/C | 0.08 | 6.81 | Gbar_D03_587910 | C/T | 0.07 | 6.53 |
| Gbar_D03_507303 | A/G | 0.08 | 7.16 | Gbar_D03_543292 | A/C | 0.08 | 7.50 | Gbar_D03_588355 | G/A | 0.07 | 6.69 |
| Gbar_D03_507341 | G/A | 0.07 | 6.56 | Gbar_D03_543367 | A/T | 0.08 | 6.45 | Gbar_D03_588368 | T/A | 0.08 | 6.57 |
| Gbar_D03_507512 | T/C | 0.08 | 6.11 | Gbar_D03_545173 | C/T | 0.08 | 6.68 | Gbar_D03_588540 | G/A | 0.08 | 6.61 |
| Gbar_D03_507538 | G/A | 0.08 | 6.70 | Gbar_D03_545273 | C/T | 0.08 | 6.81 | Gbar_D03_588734 | A/G | 0.08 | 7.19 |
| Gbar_D03_507583 | T/C | 0.08 | 6.79 | Gbar_D03_545286 | A/G | 0.08 | 6.80 | Gbar_D03_589494 | T/C | 0.08 | 7.00 |
| Gbar_D03_507601 | T/C | 0.08 | 6.90 | Gbar_D03_545509 | G/A | 0.08 | 6.67 | Gbar_D03_589584 | A/G | 0.08 | 6.03 |
| Gbar_D03_507695 | G/A | 0.08 | 6.54 | Gbar_D03_545512 | A/T | 0.07 | 6.82 | Gbar_D03_589606 | C/T | 0.08 | 6.66 |
| Gbar_D03_508454 | A/G | 0.08 | 6.36 | Gbar_D03_546536 | C/T | 0.08 | 6.11 | Gbar_D03_589777 | G/A | 0.08 | 6.73 |
| Gbar_D03_508555 | A/G | 0.08 | 7.56 | Gbar_D03_546569 | C/G | 0.08 | 6.27 | Gbar_D03_591495 | G/A | 0.08 | 6.77 |
| Gbar_D03_509339 | T/C | 0.08 | 6.63 | Gbar_D03_546637 | T/C | 0.08 | 6.10 | Gbar_D03_591529 | C/T | 0.08 | 7.05 |
| Gbar_D03_509731 | G/A | 0.08 | 7.02 | Gbar_D03_546685 | T/C | 0.08 | 6.40 | Gbar_D03_591662 | G/C | 0.08 | 6.23 |
| Gbar_D03_510004 | C/T | 0.07 | 6.98 | Gbar_D03_546709 | A/T | 0.08 | 6.60 | Gbar_D03_591765 | A/G | 0.08 | 6.44 |
| Gbar_D03_510058 | C/T | 0.07 | 6.76 | Gbar_D03_546783 | A/G | 0.08 | 6.88 | Gbar_D03_592967 | A/C | 0.08 | 6.21 |
| Gbar_D03_510314 | C/T | 0.08 | 6.71 | Gbar_D03_546915 | T/A | 0.08 | 6.82 | Gbar_D03_593143 | A/T | 0.08 | 6.88 |
| Gbar_D03_510625 | A/G | 0.08 | 6.62 | Gbar_D03_546934 | G/T | 0.08 | 6.71 | Gbar_D03_593281 | A/T | 0.08 | 6.61 |
| Gbar_D03_510669 | G/T | 0.08 | 6.97 | Gbar_D03_546945 | A/G | 0.07 | 6.49 | Gbar_D03_593846 | C/T | 0.08 | 6.70 |
| Gbar_D03_510852 | A/T | 0.08 | 7.04 | Gbar_D03_547140 | A/G | 0.08 | 6.77 | Gbar_D03_593978 | A/T | 0.07 | 6.12 |
| Gbar_D03_510856 | T/C | 0.07 | 6.33 | Gbar_D03_547507 | T/C | 0.08 | 6.01 | Gbar_D03_593983 | C/T | 0.07 | 6.52 |
| Gbar_D03_510923 | T/G | 0.07 | 6.91 | Gbar_D03_547677 | A/G | 0.08 | 6.25 | Gbar_D03_594208 | G/A | 0.08 | 6.11 |
| Gbar_D03_511182 | A/G | 0.08 | 6.93 | Gbar_D03_547888 | T/C | 0.08 | 6.15 | Gbar_D03_594215 | C/T | 0.08 | 6.04 |
| Gbar_D03_512403 | T/A | 0.08 | 6.43 | Gbar_D03_547906 | C/T | 0.08 | 6.22 | Gbar_D03_594218 | A/C | 0.08 | 6.37 |
| Gbar_D03_512774 | G/A | 0.08 | 6.40 | Gbar_D03_548405 | T/C | 0.08 | 7.13 | Gbar_D03_594365 | T/G | 0.08 | 7.01 |
| Gbar_D03_516561 | A/G | 0.08 | 7.00 | Gbar_D03_548415 | C/A | 0.09 | 7.20 | Gbar_D03_594571 | G/A | 0.08 | 7.27 |
| Gbar_D03_517023 | C/G | 0.08 | 6.21 | Gbar_D03_548478 | A/T | 0.08 | 6.10 | Gbar_D03_594606 | G/A | 0.08 | 7.27 |
| Gbar_D03_517148 | T/A | 0.08 | 6.41 | Gbar_D03_548506 | A/G | 0.08 | 6.92 | Gbar_D03_595001 | T/C | 0.08 | 6.88 |
| Gbar_D03_517899 | T/C | 0.08 | 6.57 | Gbar_D03_549502 | G/T | 0.08 | 6.24 | Gbar_D03_595135 | C/G | 0.08 | 6.14 |
| Gbar_D03_517918 | T/A | 0.07 | 6.13 | Gbar_D03_549930 | T/C | 0.08 | 6.02 | Gbar_D03_595376 | C/G | 0.08 | 6.65 |
| Gbar_D03_518070 | G/A | 0.08 | 7.43 | Gbar_D03_549974 | A/C | 0.08 | 6.50 | Gbar_D03_595385 | A/G | 0.08 | 6.83 |

**Table S2.** List of the associated SNPs identified in different datasets through GWAS for *FW* resistance in Sea Island cotton. (Continued)

| SNP | Ref/Alt | MAF | -log (p-value) | SNP | Ref/Alt | MAF | -log (p-value) | SNP | Ref/Alt | MAF | -log (p-value) |
| --- | --- | --- | --- | --- | --- | --- | --- | --- | --- | --- | --- |
| **2016** |  |  |  | Gbar_D03_630508 | A/G | 0.08 | 6.79 | Gbar_D03_680781 | A/T | 0.08 | 6.44 |
| Gbar_D03_595520 | T/G | 0.08 | 7.62 | Gbar_D03_631132 | A/T | 0.08 | 6.75 | Gbar_D03_681635 | T/C | 0.08 | 6.10 |
| Gbar_D03_595940 | G/T | 0.08 | 6.89 | Gbar_D03_631407 | T/C | 0.08 | 6.24 | Gbar_D03_681810 | C/T | 0.08 | 6.07 |
| Gbar_D03_596036 | G/A | 0.08 | 6.99 | Gbar_D03_632343 | T/C | 0.08 | 7.15 | Gbar_D03_681899 | C/A | 0.09 | 6.27 |
| Gbar_D03_597218 | T/C | 0.08 | 6.57 | Gbar_D03_632717 | C/T | 0.08 | 6.94 | Gbar_D03_682804 | T/C | 0.08 | 7.33 |
| Gbar_D03_597459 | C/T | 0.08 | 6.28 | Gbar_D03_632788 | C/T | 0.08 | 7.30 | Gbar_D03_682989 | C/A | 0.08 | 7.40 |
| Gbar_D03_597762 | T/C | 0.08 | 6.62 | Gbar_D03_633338 | C/G | 0.08 | 7.00 | Gbar_D03_683619 | C/T | 0.08 | 7.27 |
| Gbar_D03_598106 | G/A | 0.08 | 6.47 | Gbar_D03_635525 | A/T | 0.08 | 6.16 | Gbar_D03_683791 | G/A | 0.07 | 6.12 |
| Gbar_D03_598463 | T/C | 0.08 | 7.02 | Gbar_D03_635625 | A/G | 0.08 | 6.73 | Gbar_D03_684447 | G/A | 0.08 | 6.15 |
| Gbar_D03_598794 | G/A | 0.08 | 6.93 | Gbar_D03_635739 | G/A | 0.08 | 6.98 | Gbar_D03_684662 | C/A | 0.08 | 6.83 |
| Gbar_D03_599878 | A/C | 0.08 | 6.73 | Gbar_D03_636387 | T/C | 0.08 | 7.20 | Gbar_D03_684675 | T/C | 0.07 | 6.37 |
| Gbar_D03_599920 | G/T | 0.08 | 6.08 | Gbar_D03_638422 | A/T | 0.08 | 7.43 | Gbar_D03_684681 | C/A | 0.07 | 6.27 |
| Gbar_D03_600088 | T/C | 0.08 | 6.53 | Gbar_D03_638844 | G/C | 0.08 | 6.90 | Gbar_D03_684963 | C/T | 0.08 | 7.02 |
| Gbar_D03_600309 | A/T | 0.08 | 6.54 | Gbar_D03_639346 | A/C | 0.08 | 6.51 | Gbar_D03_685055 | G/A | 0.08 | 7.14 |
| Gbar_D03_600372 | G/A | 0.08 | 7.31 | Gbar_D03_640131 | T/A | 0.08 | 6.56 | Gbar_D03_685350 | G/A | 0.08 | 6.74 |
| Gbar_D03_600532 | A/C | 0.08 | 6.39 | Gbar_D03_640851 | C/T | 0.08 | 6.59 | Gbar_D03_685401 | G/A | 0.08 | 6.77 |
| Gbar_D03_600948 | G/A | 0.08 | 6.07 | Gbar_D03_641404 | T/A | 0.08 | 6.89 | Gbar_D03_686876 | C/T | 0.07 | 6.17 |
| Gbar_D03_601270 | G/A | 0.08 | 7.21 | Gbar_D03_641609 | A/G | 0.08 | 7.23 | Gbar_D03_687839 | T/C | 0.08 | 7.36 |
| Gbar_D03_601371 | C/G | 0.08 | 6.59 | Gbar_D03_641827 | T/C | 0.08 | 7.21 | Gbar_D03_687857 | G/A | 0.08 | 7.27 |
| Gbar_D03_601400 | G/A | 0.08 | 6.91 | Gbar_D03_641868 | A/C | 0.08 | 7.01 | Gbar_D03_687926 | A/T | 0.08 | 6.96 |
| Gbar_D03_601850 | T/C | 0.07 | 6.06 | Gbar_D03_642084 | G/A | 0.08 | 6.79 | Gbar_D03_688264 | G/A | 0.08 | 7.15 |
| Gbar_D03_602149 | G/T | 0.08 | 6.74 | Gbar_D03_642090 | A/G | 0.08 | 6.81 | Gbar_D03_688289 | A/G | 0.08 | 6.99 |
| Gbar_D03_602267 | C/A | 0.08 | 6.82 | Gbar_D03_643791 | A/T | 0.08 | 6.89 | Gbar_D03_688440 | T/A | 0.08 | 7.08 |
| Gbar_D03_603037 | T/C | 0.08 | 6.53 | Gbar_D03_643823 | G/C | 0.07 | 6.72 | Gbar_D03_688829 | G/A | 0.08 | 6.61 |
| Gbar_D03_603254 | G/A | 0.08 | 7.48 | Gbar_D03_643845 | A/G | 0.07 | 6.78 | Gbar_D03_688914 | T/A | 0.08 | 6.69 |
| Gbar_D03_603337 | T/G | 0.08 | 7.79 | Gbar_D03_644112 | C/T | 0.08 | 6.55 | Gbar_D03_689331 | G/A | 0.08 | 6.74 |
| Gbar_D03_604017 | C/T | 0.08 | 6.15 | Gbar_D03_644194 | A/G | 0.08 | 7.19 | Gbar_D03_689737 | G/C | 0.08 | 6.38 |
| Gbar_D03_604273 | G/A | 0.08 | 7.28 | Gbar_D03_644468 | A/T | 0.08 | 7.11 | Gbar_D03_689840 | T/A | 0.08 | 7.14 |
| Gbar_D03_604394 | A/T | 0.08 | 6.60 | Gbar_D03_644565 | A/T | 0.08 | 6.34 | Gbar_D03_690139 | C/T | 0.08 | 7.16 |
| Gbar_D03_604678 | A/G | 0.08 | 7.29 | Gbar_D03_644961 | G/A | 0.08 | 7.00 | Gbar_D03_690265 | T/C | 0.08 | 7.64 |
| Gbar_D03_604883 | G/T | 0.07 | 6.09 | Gbar_D03_647095 | C/G | 0.08 | 6.06 | Gbar_D03_690328 | T/G | 0.08 | 7.36 |
| Gbar_D03_605420 | A/C | 0.08 | 6.77 | Gbar_D03_647296 | C/A | 0.08 | 6.74 | Gbar_D03_690397 | T/C | 0.08 | 7.31 |
| Gbar_D03_605961 | A/G | 0.08 | 6.21 | Gbar_D03_648421 | T/C | 0.08 | 7.13 | Gbar_D03_690907 | T/C | 0.08 | 6.81 |
| Gbar_D03_605977 | C/T | 0.08 | 6.03 | Gbar_D03_648646 | A/T | 0.08 | 7.19 | Gbar_D03_691097 | T/C | 0.08 | 7.33 |
| Gbar_D03_606155 | A/G | 0.08 | 7.54 | Gbar_D03_648950 | G/A | 0.08 | 6.97 | Gbar_D03_691206 | G/A | 0.08 | 7.21 |
| Gbar_D03_606191 | T/C | 0.08 | 6.32 | Gbar_D03_649503 | A/G | 0.07 | 6.09 | Gbar_D03_691386 | T/C | 0.08 | 6.35 |
| Gbar_D03_606328 | G/C | 0.08 | 6.93 | Gbar_D03_650186 | G/T | 0.08 | 7.33 | Gbar_D03_691411 | C/T | 0.08 | 7.12 |
| Gbar_D03_606418 | C/T | 0.08 | 6.83 | Gbar_D03_651878 | C/T | 0.08 | 6.49 | Gbar_D03_691670 | A/G | 0.08 | 7.52 |
| Gbar_D03_607668 | A/G | 0.08 | 6.81 | Gbar_D03_651982 | C/T | 0.08 | 6.88 | Gbar_D03_691695 | T/A | 0.08 | 7.44 |
| Gbar_D03_607819 | G/T | 0.08 | 6.96 | Gbar_D03_652175 | T/C | 0.08 | 6.71 | Gbar_D03_691815 | G/A | 0.08 | 6.80 |
| Gbar_D03_608224 | A/G | 0.08 | 7.46 | Gbar_D03_652236 | C/T | 0.08 | 6.86 | Gbar_D03_691924 | C/G | 0.08 | 7.00 |
| Gbar_D03_609313 | C/A | 0.08 | 6.45 | Gbar_D03_652617 | C/T | 0.08 | 6.57 | Gbar_D03_692297 | C/T | 0.08 | 6.89 |
| Gbar_D03_609723 | G/A | 0.08 | 6.41 | Gbar_D03_652926 | C/T | 0.08 | 6.90 | Gbar_D03_692350 | A/C | 0.08 | 7.40 |
| Gbar_D03_609844 | A/G | 0.08 | 6.84 | Gbar_D03_653219 | G/A | 0.08 | 6.03 | Gbar_D03_692682 | G/C | 0.08 | 6.18 |
| Gbar_D03_609904 | C/T | 0.08 | 7.17 | Gbar_D03_653227 | G/A | 0.08 | 6.27 | Gbar_D03_693008 | A/T | 0.08 | 6.22 |
| Gbar_D03_610091 | A/T | 0.08 | 6.63 | Gbar_D03_653249 | C/T | 0.07 | 6.83 | Gbar_D03_693260 | G/A | 0.08 | 6.12 |
| Gbar_D03_610595 | T/C | 0.08 | 6.49 | Gbar_D03_653369 | T/C | 0.08 | 7.24 | Gbar_D03_693585 | A/G | 0.08 | 7.11 |
| Gbar_D03_611407 | A/T | 0.08 | 6.14 | Gbar_D03_653399 | C/A | 0.08 | 6.91 | Gbar_D03_693592 | T/C | 0.08 | 7.07 |
| Gbar_D03_611581 | C/A | 0.08 | 7.16 | Gbar_D03_653541 | G/T | 0.09 | 7.26 | Gbar_D03_693634 | A/G | 0.08 | 7.01 |
| Gbar_D03_612296 | G/A | 0.08 | 6.15 | Gbar_D03_653844 | C/T | 0.08 | 7.56 | Gbar_D03_693671 | T/C | 0.08 | 7.10 |
| Gbar_D03_612329 | A/G | 0.07 | 6.41 | Gbar_D03_653863 | G/A | 0.08 | 7.63 | Gbar_D03_693705 | G/A | 0.08 | 7.07 |
| Gbar_D03_612422 | A/G | 0.08 | 6.74 | Gbar_D03_653900 | A/T | 0.08 | 8.00 | Gbar_D03_694047 | T/C | 0.08 | 6.35 |
| Gbar_D03_612444 | T/C | 0.08 | 6.80 | Gbar_D03_655498 | C/T | 0.08 | 6.44 | Gbar_D03_694240 | T/A | 0.08 | 6.17 |
| Gbar_D03_612619 | A/G | 0.08 | 6.74 | Gbar_D03_655685 | G/A | 0.08 | 6.67 | Gbar_D03_694243 | A/T | 0.08 | 6.10 |
| Gbar_D03_612946 | A/T | 0.08 | 7.54 | Gbar_D03_656976 | A/G | 0.08 | 7.40 | Gbar_D03_694384 | T/C | 0.08 | 7.20 |
| Gbar_D03_613046 | A/G | 0.08 | 7.09 | Gbar_D03_656992 | T/C | 0.08 | 7.12 | Gbar_D03_694393 | C/T | 0.08 | 6.43 |
| Gbar_D03_613067 | T/C | 0.08 | 6.93 | Gbar_D03_657002 | A/G | 0.08 | 6.90 | Gbar_D03_694535 | T/C | 0.08 | 6.41 |
| Gbar_D03_613631 | C/A | 0.08 | 6.90 | Gbar_D03_658071 | T/A | 0.08 | 6.76 | Gbar_D03_695003 | A/G | 0.08 | 6.32 |
| Gbar_D03_613813 | A/C | 0.08 | 6.14 | Gbar_D03_658423 | C/G | 0.08 | 6.47 | Gbar_D03_695028 | G/T | 0.09 | 6.86 |
| Gbar_D03_613955 | A/G | 0.08 | 6.89 | Gbar_D03_658777 | T/C | 0.08 | 7.56 | Gbar_D03_695231 | C/T | 0.08 | 6.04 |
| Gbar_D03_614340 | G/A | 0.08 | 6.43 | Gbar_D03_660586 | A/G | 0.08 | 7.16 | Gbar_D03_695381 | A/C | 0.08 | 6.29 |
| Gbar_D03_614536 | T/C | 0.08 | 6.81 | Gbar_D03_660707 | T/C | 0.08 | 6.93 | Gbar_D03_697224 | G/C | 0.08 | 7.24 |
| Gbar_D03_614896 | T/C | 0.08 | 7.54 | Gbar_D03_661386 | C/A | 0.08 | 7.00 | Gbar_D03_697559 | A/G | 0.08 | 6.46 |
| Gbar_D03_614953 | T/C | 0.08 | 7.30 | Gbar_D03_662294 | A/G | 0.08 | 7.65 | Gbar_D03_697734 | G/T | 0.08 | 6.83 |
| Gbar_D03_615142 | G/C | 0.08 | 6.46 | Gbar_D03_662391 | C/A | 0.08 | 6.63 | Gbar_D03_699771 | A/G | 0.08 | 7.09 |
| Gbar_D03_615180 | A/G | 0.08 | 7.30 | Gbar_D03_662434 | A/C | 0.08 | 6.40 | Gbar_D03_700031 | A/G | 0.08 | 6.89 |
| Gbar_D03_615713 | T/A | 0.08 | 6.98 | Gbar_D03_662519 | G/A | 0.08 | 6.74 | Gbar_D03_700149 | C/T | 0.08 | 6.55 |
| Gbar_D03_615776 | T/C | 0.08 | 7.07 | Gbar_D03_662524 | C/T | 0.08 | 6.61 | Gbar_D03_700713 | A/C | 0.08 | 6.87 |
| Gbar_D03_615790 | A/T | 0.08 | 7.30 | Gbar_D03_662685 | C/T | 0.08 | 7.11 | Gbar_D03_700805 | C/G | 0.08 | 6.08 |
| Gbar_D03_616070 | G/A | 0.08 | 7.00 | Gbar_D03_662796 | G/A | 0.08 | 6.69 | Gbar_D03_700896 | T/C | 0.07 | 6.65 |
| Gbar_D03_616321 | T/C | 0.09 | 6.78 | Gbar_D03_662920 | A/T | 0.07 | 6.42 | Gbar_D03_701142 | A/T | 0.08 | 7.32 |
| Gbar_D03_616638 | T/C | 0.08 | 7.04 | Gbar_D03_662986 | T/A | 0.08 | 7.34 | Gbar_D03_701637 | A/T | 0.07 | 6.10 |
| Gbar_D03_617081 | G/A | 0.08 | 7.38 | Gbar_D03_663730 | T/C | 0.08 | 7.01 | Gbar_D03_702165 | T/G | 0.08 | 6.87 |
| Gbar_D03_617139 | C/T | 0.08 | 7.59 | Gbar_D03_664095 | G/C | 0.08 | 7.30 | Gbar_D03_702396 | G/C | 0.07 | 6.38 |
| Gbar_D03_617385 | G/A | 0.08 | 6.20 | Gbar_D03_664883 | T/G | 0.08 | 6.44 | Gbar_D03_702618 | A/T | 0.08 | 6.45 |
| Gbar_D03_618792 | G/A | 0.08 | 6.12 | Gbar_D03_665422 | G/A | 0.08 | 6.85 | Gbar_D03_703034 | T/C | 0.08 | 6.04 |
| Gbar_D03_618859 | A/C | 0.08 | 6.14 | Gbar_D03_665945 | C/T | 0.08 | 6.73 | Gbar_D03_703054 | C/A | 0.08 | 6.32 |
| Gbar_D03_618863 | A/T | 0.08 | 6.20 | Gbar_D03_666060 | T/C | 0.07 | 6.04 | Gbar_D03_703060 | C/T | 0.08 | 6.47 |
| Gbar_D03_619355 | G/T | 0.08 | 6.16 | Gbar_D03_666072 | C/T | 0.07 | 6.01 | Gbar_D03_703065 | T/C | 0.08 | 6.35 |
| Gbar_D03_619689 | G/A | 0.08 | 6.56 | Gbar_D03_666082 | C/T | 0.08 | 6.02 | Gbar_D03_703498 | C/T | 0.08 | 7.58 |
| Gbar_D03_619731 | C/G | 0.08 | 6.60 | Gbar_D03_666219 | T/C | 0.08 | 6.82 | Gbar_D03_703532 | C/T | 0.08 | 6.46 |
| Gbar_D03_620292 | T/C | 0.08 | 7.42 | Gbar_D03_666320 | C/T | 0.08 | 6.94 | Gbar_D03_704291 | T/A | 0.07 | 6.77 |
| Gbar_D03_620407 | T/C | 0.08 | 6.40 | Gbar_D03_666372 | G/A | 0.08 | 7.11 | Gbar_D03_706397 | G/T | 0.08 | 7.16 |
| Gbar_D03_620424 | A/C | 0.08 | 6.39 | Gbar_D03_666912 | T/C | 0.08 | 6.10 | Gbar_D03_706853 | T/C | 0.08 | 7.02 |
| Gbar_D03_620702 | G/C | 0.08 | 6.98 | Gbar_D03_667281 | C/A | 0.08 | 7.35 | Gbar_D03_706901 | A/G | 0.08 | 7.07 |
| Gbar_D03_621175 | G/T | 0.07 | 6.80 | Gbar_D03_667390 | T/A | 0.08 | 7.04 | Gbar_D03_707326 | T/C | 0.08 | 7.40 |
| Gbar_D03_621182 | A/T | 0.07 | 6.75 | Gbar_D03_667639 | A/C | 0.08 | 6.97 | Gbar_D03_708318 | A/C | 0.08 | 6.94 |
| Gbar_D03_621353 | T/G | 0.08 | 6.57 | Gbar_D03_669501 | G/A | 0.08 | 7.39 | Gbar_D03_709037 | C/G | 0.07 | 6.23 |
| Gbar_D03_621810 | G/A | 0.08 | 7.54 | Gbar_D03_670305 | A/G | 0.08 | 6.60 | Gbar_D03_709055 | T/C | 0.08 | 6.45 |
| Gbar_D03_621871 | A/T | 0.08 | 7.30 | Gbar_D03_670662 | G/T | 0.07 | 6.30 | Gbar_D03_709387 | T/C | 0.08 | 6.63 |
| Gbar_D03_621993 | C/T | 0.08 | 6.25 | Gbar_D03_670681 | C/A | 0.08 | 7.07 | Gbar_D03_709747 | A/T | 0.08 | 7.08 |
| Gbar_D03_622680 | A/G | 0.08 | 6.92 | Gbar_D03_670927 | A/G | 0.08 | 6.84 | Gbar_D03_709909 | C/G | 0.08 | 7.35 |
| Gbar_D03_622721 | G/A | 0.08 | 6.20 | Gbar_D03_671090 | T/C | 0.07 | 7.07 | Gbar_D03_711023 | G/A | 0.08 | 6.09 |
| Gbar_D03_622755 | C/T | 0.08 | 6.99 | Gbar_D03_671656 | A/T | 0.08 | 6.25 | Gbar_D03_711031 | C/T | 0.08 | 6.75 |
| Gbar_D03_622781 | C/G | 0.08 | 6.55 | Gbar_D03_671919 | C/T | 0.08 | 6.94 | Gbar_D03_711056 | A/G | 0.08 | 6.69 |
| Gbar_D03_624152 | G/A | 0.08 | 7.10 | Gbar_D03_671942 | G/T | 0.07 | 6.76 | Gbar_D03_711322 | C/T | 0.08 | 6.57 |
| Gbar_D03_624169 | T/C | 0.08 | 6.07 | Gbar_D03_672496 | G/A | 0.08 | 6.82 | Gbar_D03_711548 | C/T | 0.08 | 6.38 |
| Gbar_D03_624216 | G/C | 0.08 | 6.81 | Gbar_D03_673021 | C/T | 0.08 | 7.04 | Gbar_D03_711741 | T/C | 0.08 | 6.44 |
| Gbar_D03_624387 | A/G | 0.08 | 6.96 | Gbar_D03_673181 | A/C | 0.08 | 6.84 | Gbar_D03_711951 | G/A | 0.08 | 6.64 |
| Gbar_D03_625037 | C/G | 0.08 | 7.25 | Gbar_D03_673236 | T/G | 0.08 | 6.80 | Gbar_D03_711956 | C/T | 0.08 | 6.44 |
| Gbar_D03_625152 | C/T | 0.08 | 6.99 | Gbar_D03_673341 | C/A | 0.08 | 6.39 | Gbar_D03_711964 | T/C | 0.08 | 6.62 |
| Gbar_D03_626683 | A/G | 0.07 | 6.68 | Gbar_D03_674661 | C/A | 0.08 | 6.47 | Gbar_D03_712396 | T/C | 0.08 | 6.17 |
| Gbar_D03_626688 | G/C | 0.07 | 6.62 | Gbar_D03_675228 | T/A | 0.08 | 6.74 | Gbar_D03_712651 | A/C | 0.07 | 6.42 |
| Gbar_D03_627517 | C/T | 0.08 | 7.19 | Gbar_D03_676610 | T/A | 0.08 | 6.41 | Gbar_D03_712797 | G/A | 0.07 | 6.64 |
| Gbar_D03_628525 | C/T | 0.08 | 6.83 | Gbar_D03_677040 | C/A | 0.08 | 6.16 | Gbar_D03_713238 | A/G | 0.08 | 6.37 |
| Gbar_D03_629115 | G/C | 0.08 | 6.56 | Gbar_D03_678108 | C/T | 0.09 | 6.72 | Gbar_D03_713258 | C/T | 0.08 | 6.81 |
| Gbar_D03_630237 | T/C | 0.08 | 7.05 | Gbar_D03_678703 | T/C | 0.08 | 6.30 | Gbar_D03_713484 | A/T | 0.08 | 7.22 |
| Gbar_D03_630241 | C/T | 0.08 | 6.99 | Gbar_D03_680715 | G/A | 0.07 | 6.47 | Gbar_D03_713500 | T/C | 0.08 | 7.18 |
| Gbar_D03_630358 | A/G | 0.08 | 6.58 | Gbar_D03_680754 | A/C | 0.08 | 6.70 | Gbar_D03_714281 | A/C | 0.08 | 7.10 |
| Gbar_D03_630448 | A/C | 0.08 | 7.31 | Gbar_D03_680769 | T/C | 0.08 | 6.83 | Gbar_D03_715022 | T/C | 0.08 | 6.22 |

**Table S2.** List of the associated SNPs identified in different datasets through GWAS for *FW* resistance in Sea Island cotton. (Continued)

| SNP | Ref/Alt | MAF | -log (p-value) | SNP | Ref/Alt | MAF | -log (p-value) | SNP | Ref/Alt | MAF | -log (p-value) |
| --- | --- | --- | --- | --- | --- | --- | --- | --- | --- | --- | --- |
| **2016** |  |  |  | Gbar_D03_750908 | G/T | 0.08 | 6.93 | Gbar_D03_777093 | C/T | 0.08 | 7.68 |
| Gbar_D03_715304 | A/G | 0.08 | 7.30 | Gbar_D03_750962 | G/C | 0.08 | 6.97 | Gbar_D03_777111 | T/G | 0.08 | 7.46 |
| Gbar_D03_715379 | T/A | 0.08 | 6.43 | Gbar_D03_751937 | C/T | 0.07 | 6.16 | Gbar_D03_777135 | T/G | 0.08 | 7.81 |
| Gbar_D03_715785 | C/T | 0.08 | 6.81 | Gbar_D03_752091 | T/A | 0.08 | 6.93 | Gbar_D03_777146 | A/G | 0.08 | 7.30 |
| Gbar_D03_715832 | A/G | 0.08 | 6.71 | Gbar_D03_752271 | T/C | 0.08 | 6.75 | Gbar_D03_777215 | T/A | 0.08 | 7.55 |
| Gbar_D03_716243 | T/C | 0.08 | 7.13 | Gbar_D03_752691 | A/C | 0.08 | 6.28 | Gbar_D03_777669 | C/T | 0.08 | 7.05 |
| Gbar_D03_716250 | G/A | 0.08 | 7.39 | Gbar_D03_752720 | G/A | 0.09 | 6.25 | Gbar_D03_777938 | T/G | 0.08 | 6.29 |
| Gbar_D03_716447 | A/G | 0.08 | 7.09 | Gbar_D03_753046 | C/G | 0.08 | 6.91 | Gbar_D03_778036 | T/C | 0.08 | 6.04 |
| Gbar_D03_716766 | A/G | 0.08 | 7.16 | Gbar_D03_753863 | A/G | 0.08 | 6.77 | Gbar_D03_778610 | G/A | 0.08 | 6.21 |
| Gbar_D03_717015 | A/G | 0.08 | 6.67 | Gbar_D03_754158 | C/T | 0.08 | 8.07 | Gbar_D03_779389 | T/C | 0.08 | 7.12 |
| Gbar_D03_717407 | T/C | 0.08 | 7.47 | Gbar_D03_754171 | A/G | 0.08 | 6.72 | Gbar_D03_779918 | C/T | 0.08 | 7.14 |
| Gbar_D03_717478 | A/C | 0.08 | 6.69 | Gbar_D03_754191 | A/T | 0.08 | 7.29 | Gbar_D03_780704 | A/G | 0.08 | 8.17 |
| Gbar_D03_717670 | T/C | 0.08 | 6.30 | Gbar_D03_754844 | A/G | 0.08 | 6.73 | Gbar_D03_781370 | C/T | 0.08 | 6.61 |
| Gbar_D03_717695 | G/A | 0.08 | 6.59 | Gbar_D03_754968 | G/A | 0.08 | 6.64 | Gbar_D03_786604 | A/T | 0.08 | 7.42 |
| Gbar_D03_717707 | C/T | 0.08 | 6.23 | Gbar_D03_755431 | T/C | 0.08 | 6.88 | Gbar_D03_789050 | C/T | 0.08 | 6.94 |
| Gbar_D03_717742 | G/A | 0.08 | 6.38 | Gbar_D03_755820 | T/C | 0.08 | 7.18 | Gbar_D03_789434 | A/G | 0.08 | 6.75 |
| Gbar_D03_717779 | T/C | 0.09 | 7.52 | Gbar_D03_756699 | C/A | 0.08 | 6.26 | Gbar_D03_789555 | G/A | 0.08 | 6.37 |
| Gbar_D03_717803 | C/G | 0.09 | 7.08 | Gbar_D03_756879 | A/G | 0.08 | 6.89 | Gbar_D03_789594 | A/C | 0.08 | 6.18 |
| Gbar_D03_717823 | C/A | 0.08 | 7.70 | Gbar_D03_757401 | T/C | 0.08 | 6.58 | Gbar_D03_790890 | G/C | 0.08 | 7.23 |
| Gbar_D03_717913 | A/T | 0.08 | 6.10 | Gbar_D03_757690 | G/A | 0.09 | 7.19 | Gbar_D03_791028 | T/G | 0.08 | 6.84 |
| Gbar_D03_718086 | T/C | 0.08 | 6.31 | Gbar_D03_757878 | C/T | 0.08 | 7.14 | Gbar_D03_791052 | T/C | 0.08 | 6.78 |
| Gbar_D03_718221 | C/A | 0.08 | 7.20 | Gbar_D03_757949 | T/A | 0.08 | 6.92 | Gbar_D03_791537 | C/G | 0.08 | 6.46 |
| Gbar_D03_718281 | A/G | 0.08 | 8.24 | Gbar_D03_757977 | T/A | 0.08 | 7.30 | Gbar_D03_791742 | T/C | 0.08 | 7.06 |
| Gbar_D03_718293 | T/C | 0.08 | 8.05 | Gbar_D03_757988 | A/C | 0.08 | 7.09 | Gbar_D03_791768 | T/A | 0.08 | 7.07 |
| Gbar_D03_718418 | C/T | 0.08 | 6.44 | Gbar_D03_758002 | A/G | 0.08 | 7.24 | Gbar_D03_792002 | A/G | 0.08 | 6.91 |
| Gbar_D03_718489 | C/T | 0.08 | 6.66 | Gbar_D03_758220 | T/A | 0.08 | 7.17 | Gbar_D03_792243 | G/A | 0.08 | 6.66 |
| Gbar_D03_718632 | T/C | 0.08 | 6.02 | Gbar_D03_758463 | T/C | 0.08 | 6.37 | Gbar_D03_792244 | T/C | 0.08 | 6.73 |
| Gbar_D03_719951 | G/C | 0.08 | 6.86 | Gbar_D03_759120 | T/C | 0.08 | 6.93 | Gbar_D03_792267 | A/G | 0.08 | 6.82 |
| Gbar_D03_719969 | G/A | 0.07 | 6.39 | Gbar_D03_760090 | T/A | 0.08 | 6.57 | Gbar_D03_792353 | A/T | 0.08 | 6.62 |
| Gbar_D03_721035 | T/C | 0.08 | 7.24 | Gbar_D03_760235 | C/T | 0.08 | 6.10 | Gbar_D03_792622 | C/T | 0.08 | 7.18 |
| Gbar_D03_721318 | T/A | 0.08 | 6.13 | Gbar_D03_760482 | T/G | 0.08 | 6.88 | Gbar_D03_792702 | T/C | 0.08 | 7.40 |
| Gbar_D03_721848 | T/A | 0.08 | 6.95 | Gbar_D03_760524 | T/C | 0.08 | 6.87 | Gbar_D03_792827 | A/G | 0.08 | 6.49 |
| Gbar_D03_722369 | G/A | 0.07 | 6.05 | Gbar_D03_761174 | G/A | 0.08 | 6.25 | Gbar_D03_792847 | C/T | 0.08 | 7.17 |
| Gbar_D03_722427 | A/G | 0.07 | 6.47 | Gbar_D03_761235 | C/A | 0.08 | 6.36 | Gbar_D03_793315 | C/T | 0.08 | 6.99 |
| Gbar_D03_722630 | C/T | 0.08 | 6.69 | Gbar_D03_761532 | T/C | 0.08 | 6.75 | Gbar_D03_793333 | C/T | 0.08 | 7.38 |
| Gbar_D03_722745 | T/C | 0.08 | 7.09 | Gbar_D03_761605 | A/G | 0.08 | 7.00 | Gbar_D03_793566 | G/A | 0.08 | 7.11 |
| Gbar_D03_723335 | C/T | 0.08 | 6.18 | Gbar_D03_761776 | C/T | 0.08 | 7.23 | Gbar_D03_793819 | G/A | 0.08 | 6.32 |
| Gbar_D03_723816 | G/A | 0.08 | 7.74 | Gbar_D03_762044 | G/T | 0.08 | 7.34 | Gbar_D03_794186 | T/A | 0.08 | 7.04 |
| Gbar_D03_723821 | C/T | 0.08 | 7.43 | Gbar_D03_762239 | C/T | 0.08 | 7.34 | Gbar_D03_794227 | T/G | 0.08 | 7.16 |
| Gbar_D03_724046 | C/T | 0.08 | 6.14 | Gbar_D03_762484 | T/C | 0.08 | 6.48 | Gbar_D03_794306 | G/A | 0.08 | 7.16 |
| Gbar_D03_724074 | A/G | 0.08 | 6.29 | Gbar_D03_762656 | G/A | 0.08 | 7.01 | Gbar_D03_794426 | G/T | 0.08 | 6.98 |
| Gbar_D03_724108 | C/T | 0.08 | 6.21 | Gbar_D03_762788 | T/A | 0.08 | 6.90 | Gbar_D03_794437 | C/A | 0.08 | 7.04 |
| Gbar_D03_724928 | C/T | 0.08 | 6.02 | Gbar_D03_764099 | A/G | 0.08 | 6.65 | Gbar_D03_794472 | C/T | 0.08 | 6.91 |
| Gbar_D03_727507 | T/C | 0.07 | 6.18 | Gbar_D03_764500 | G/T | 0.08 | 6.07 | Gbar_D03_794550 | G/A | 0.08 | 6.94 |
| Gbar_D03_728310 | T/A | 0.08 | 6.70 | Gbar_D03_764536 | T/A | 0.08 | 6.25 | Gbar_D03_795285 | C/T | 0.08 | 6.44 |
| Gbar_D03_728441 | A/C | 0.08 | 6.48 | Gbar_D03_764555 | G/A | 0.08 | 6.01 | Gbar_D03_795465 | T/A | 0.08 | 6.93 |
| Gbar_D03_728634 | T/A | 0.08 | 7.38 | Gbar_D03_764818 | G/C | 0.08 | 6.02 | Gbar_D03_795516 | T/A | 0.08 | 7.24 |
| Gbar_D03_729235 | T/A | 0.07 | 6.26 | Gbar_D03_764820 | T/C | 0.07 | 6.04 | Gbar_D03_795532 | G/A | 0.08 | 6.72 |
| Gbar_D03_729685 | C/A | 0.08 | 7.39 | Gbar_D03_764935 | C/T | 0.08 | 6.40 | Gbar_D03_795574 | A/G | 0.08 | 7.21 |
| Gbar_D03_729694 | T/G | 0.08 | 7.39 | Gbar_D03_765112 | T/C | 0.08 | 6.52 | Gbar_D03_795798 | G/A | 0.08 | 7.13 |
| Gbar_D03_730450 | T/C | 0.08 | 7.39 | Gbar_D03_766362 | C/T | 0.08 | 7.26 | Gbar_D03_795872 | T/G | 0.08 | 6.29 |
| Gbar_D03_730631 | T/A | 0.08 | 6.25 | Gbar_D03_766470 | G/C | 0.08 | 7.38 | Gbar_D03_796062 | G/T | 0.08 | 6.45 |
| Gbar_D03_730643 | G/A | 0.08 | 7.10 | Gbar_D03_766476 | G/C | 0.08 | 7.07 | Gbar_D03_796082 | G/A | 0.08 | 6.07 |
| Gbar_D03_730898 | G/A | 0.08 | 6.29 | Gbar_D03_766747 | T/A | 0.08 | 6.58 | Gbar_D03_796100 | T/C | 0.08 | 6.42 |
| Gbar_D03_731173 | C/T | 0.08 | 6.62 | Gbar_D03_767268 | A/G | 0.08 | 7.84 | Gbar_D03_796101 | A/C | 0.08 | 6.50 |
| Gbar_D03_731174 | A/G | 0.08 | 6.75 | Gbar_D03_767400 | A/G | 0.08 | 7.07 | Gbar_D03_796198 | T/C | 0.08 | 6.30 |
| Gbar_D03_731506 | A/G | 0.08 | 7.17 | Gbar_D03_767483 | G/A | 0.08 | 6.93 | Gbar_D03_796307 | A/G | 0.08 | 6.88 |
| Gbar_D03_731523 | C/T | 0.08 | 6.98 | Gbar_D03_768013 | T/C | 0.08 | 7.20 | Gbar_D03_796823 | T/C | 0.08 | 7.01 |
| Gbar_D03_731617 | C/T | 0.08 | 6.14 | Gbar_D03_768014 | C/T | 0.08 | 7.14 | Gbar_D03_796888 | T/C | 0.08 | 7.30 |
| Gbar_D03_731669 | C/A | 0.08 | 7.19 | Gbar_D03_768062 | C/T | 0.08 | 6.90 | Gbar_D03_797066 | G/T | 0.08 | 7.31 |
| Gbar_D03_732313 | A/G | 0.08 | 7.28 | Gbar_D03_768071 | A/G | 0.08 | 7.03 | Gbar_D03_797150 | A/T | 0.08 | 6.84 |
| Gbar_D03_732565 | G/A | 0.08 | 7.17 | Gbar_D03_768188 | A/G | 0.08 | 6.88 | Gbar_D03_797573 | T/A | 0.08 | 6.43 |
| Gbar_D03_732765 | T/A | 0.08 | 6.25 | Gbar_D03_768195 | G/C | 0.08 | 7.14 | Gbar_D03_798678 | G/T | 0.08 | 6.08 |
| Gbar_D03_733818 | T/C | 0.08 | 7.37 | Gbar_D03_768199 | G/T | 0.08 | 7.14 | Gbar_D03_798962 | C/T | 0.08 | 6.84 |
| Gbar_D03_733937 | C/T | 0.08 | 7.16 | Gbar_D03_768721 | T/C | 0.08 | 6.15 | Gbar_D03_799134 | A/G | 0.08 | 7.07 |
| Gbar_D03_733973 | A/G | 0.08 | 7.25 | Gbar_D03_768750 | T/C | 0.08 | 6.27 | Gbar_D03_799247 | C/T | 0.08 | 6.25 |
| Gbar_D03_733995 | G/A | 0.08 | 6.17 | Gbar_D03_769125 | C/A | 0.09 | 6.92 | Gbar_D03_799255 | A/T | 0.08 | 6.44 |
| Gbar_D03_734227 | T/C | 0.08 | 6.93 | Gbar_D03_769406 | A/T | 0.08 | 7.22 | Gbar_D03_799256 | A/T | 0.08 | 6.44 |
| Gbar_D03_736371 | G/T | 0.08 | 7.12 | Gbar_D03_769597 | A/G | 0.08 | 6.86 | Gbar_D03_799319 | T/C | 0.08 | 6.12 |
| Gbar_D03_737104 | T/A | 0.08 | 7.16 | Gbar_D03_769704 | A/G | 0.08 | 7.04 | Gbar_D03_799394 | C/A | 0.08 | 6.48 |
| Gbar_D03_737229 | G/A | 0.08 | 7.05 | Gbar_D03_770296 | T/C | 0.08 | 6.91 | Gbar_D03_800106 | C/T | 0.08 | 7.43 |
| Gbar_D03_737284 | T/C | 0.07 | 6.47 | Gbar_D03_770656 | G/A | 0.08 | 7.22 | Gbar_D03_800241 | A/G | 0.08 | 7.05 |
| Gbar_D03_737374 | G/A | 0.08 | 7.07 | Gbar_D03_771239 | T/C | 0.08 | 6.82 | Gbar_D03_800458 | C/T | 0.08 | 7.16 |
| Gbar_D03_737402 | A/G | 0.08 | 6.67 | Gbar_D03_771322 | T/C | 0.08 | 6.69 | Gbar_D03_802124 | G/A | 0.08 | 7.06 |
| Gbar_D03_738020 | G/A | 0.08 | 6.47 | Gbar_D03_771391 | A/G | 0.08 | 6.56 | Gbar_D03_802482 | T/C | 0.08 | 6.01 |
| Gbar_D03_738692 | G/T | 0.08 | 6.91 | Gbar_D03_771395 | T/C | 0.08 | 7.03 | Gbar_D03_802651 | A/T | 0.08 | 6.03 |
| Gbar_D03_738725 | C/T | 0.08 | 7.21 | Gbar_D03_771406 | A/G | 0.08 | 6.92 | Gbar_D03_802954 | C/A | 0.08 | 7.49 |
| Gbar_D03_739176 | T/C | 0.08 | 6.82 | Gbar_D03_771825 | G/A | 0.08 | 7.20 | Gbar_D03_802966 | C/T | 0.08 | 7.34 |
| Gbar_D03_740008 | G/C | 0.08 | 7.39 | Gbar_D03_772080 | G/T | 0.08 | 6.74 | Gbar_D03_803002 | A/T | 0.08 | 6.61 |
| Gbar_D03_740731 | A/G | 0.08 | 6.06 | Gbar_D03_772090 | G/A | 0.08 | 6.92 | Gbar_D03_803219 | C/T | 0.08 | 7.36 |
| Gbar_D03_740811 | A/G | 0.08 | 6.97 | Gbar_D03_772091 | G/A | 0.08 | 7.20 | Gbar_D03_803493 | C/T | 0.08 | 6.52 |
| Gbar_D03_741159 | C/G | 0.08 | 7.37 | Gbar_D03_772111 | C/T | 0.08 | 6.75 | Gbar_D03_803800 | C/G | 0.08 | 7.15 |
| Gbar_D03_743507 | A/G | 0.08 | 6.31 | Gbar_D03_772929 | A/G | 0.08 | 6.78 | Gbar_D03_804931 | A/C | 0.08 | 6.93 |
| Gbar_D03_743650 | A/T | 0.08 | 6.07 | Gbar_D03_773583 | A/T | 0.08 | 6.02 | Gbar_D03_804990 | T/C | 0.08 | 6.45 |
| Gbar_D03_743755 | T/C | 0.08 | 6.49 | Gbar_D03_773717 | G/A | 0.08 | 7.19 | Gbar_D03_805316 | G/T | 0.08 | 6.28 |
| Gbar_D03_743815 | G/A | 0.08 | 7.24 | Gbar_D03_773986 | C/T | 0.08 | 6.10 | Gbar_D03_805693 | A/T | 0.08 | 6.09 |
| Gbar_D03_743864 | T/C | 0.08 | 6.92 | Gbar_D03_774109 | G/A | 0.08 | 6.49 | Gbar_D03_806228 | G/T | 0.08 | 6.83 |
| Gbar_D03_743905 | T/C | 0.08 | 7.11 | Gbar_D03_774292 | C/G | 0.08 | 6.33 | Gbar_D03_806237 | A/G | 0.08 | 6.86 |
| Gbar_D03_744095 | A/T | 0.08 | 7.39 | Gbar_D03_774400 | C/T | 0.08 | 7.04 | Gbar_D03_806328 | G/C | 0.08 | 7.26 |
| Gbar_D03_744220 | T/G | 0.08 | 7.40 | Gbar_D03_774415 | C/T | 0.08 | 7.58 | Gbar_D03_806361 | A/G | 0.08 | 7.05 |
| Gbar_D03_744715 | A/C | 0.08 | 6.25 | Gbar_D03_774439 | T/C | 0.08 | 7.27 | Gbar_D03_806465 | T/C | 0.08 | 6.40 |
| Gbar_D03_744859 | G/C | 0.08 | 7.04 | Gbar_D03_774458 | C/T | 0.08 | 7.09 | Gbar_D03_806529 | A/G | 0.08 | 6.72 |
| Gbar_D03_745085 | G/A | 0.08 | 6.03 | Gbar_D03_774516 | A/C | 0.08 | 7.04 | Gbar_D03_806539 | T/C | 0.08 | 6.26 |
| Gbar_D03_745620 | T/G | 0.08 | 6.18 | Gbar_D03_774523 | A/T | 0.08 | 6.84 | Gbar_D03_806589 | T/C | 0.08 | 6.10 |
| Gbar_D03_746844 | A/C | 0.08 | 7.34 | Gbar_D03_775070 | C/T | 0.08 | 6.98 | Gbar_D03_807754 | T/A | 0.08 | 7.51 |
| Gbar_D03_746977 | C/G | 0.08 | 7.57 | Gbar_D03_775094 | T/A | 0.08 | 6.75 | Gbar_D03_807990 | C/T | 0.08 | 7.34 |
| Gbar_D03_747092 | A/T | 0.08 | 6.77 | Gbar_D03_775183 | A/C | 0.08 | 6.98 | Gbar_D03_808150 | A/C | 0.08 | 6.76 |
| Gbar_D03_748718 | G/A | 0.08 | 6.31 | Gbar_D03_775461 | T/G | 0.08 | 7.06 | Gbar_D03_808265 | C/G | 0.08 | 6.19 |
| Gbar_D03_748883 | C/A | 0.08 | 6.68 | Gbar_D03_775562 | A/G | 0.08 | 6.96 | Gbar_D03_808550 | T/G | 0.08 | 7.41 |
| Gbar_D03_748898 | A/G | 0.08 | 6.52 | Gbar_D03_775614 | G/A | 0.08 | 7.32 | Gbar_D03_809610 | C/T | 0.08 | 7.07 |
| Gbar_D03_748937 | C/A | 0.09 | 6.01 | Gbar_D03_776147 | G/A | 0.08 | 6.65 | Gbar_D03_809907 | T/A | 0.08 | 7.96 |
| Gbar_D03_749121 | A/T | 0.08 | 6.71 | Gbar_D03_776247 | A/G | 0.08 | 6.55 | Gbar_D03_809936 | T/C | 0.08 | 7.58 |
| Gbar_D03_749122 | T/A | 0.08 | 6.62 | Gbar_D03_776271 | C/A | 0.08 | 6.81 | Gbar_D03_810081 | C/T | 0.08 | 6.39 |
| Gbar_D03_749369 | C/T | 0.08 | 6.83 | Gbar_D03_776345 | A/G | 0.08 | 6.74 | Gbar_D03_810116 | C/T | 0.08 | 6.55 |
| Gbar_D03_749685 | G/A | 0.08 | 7.17 | Gbar_D03_776580 | G/C | 0.08 | 6.88 | Gbar_D03_810150 | G/A | 0.08 | 6.74 |
| Gbar_D03_749891 | A/T | 0.08 | 6.43 | Gbar_D03_776590 | A/G | 0.08 | 6.65 | Gbar_D03_810613 | A/G | 0.08 | 8.07 |
| Gbar_D03_750257 | G/A | 0.08 | 6.28 | Gbar_D03_776611 | G/C | 0.08 | 6.81 | Gbar_D03_810680 | G/A | 0.07 | 6.57 |
| Gbar_D03_750408 | C/T | 0.08 | 7.16 | Gbar_D03_776691 | C/A | 0.08 | 6.40 | Gbar_D03_811064 | A/T | 0.08 | 6.60 |
| Gbar_D03_750411 | G/A | 0.08 | 7.22 | Gbar_D03_776798 | T/C | 0.08 | 7.10 | Gbar_D03_811336 | T/C | 0.08 | 7.33 |
| Gbar_D03_750737 | C/T | 0.08 | 7.32 | Gbar_D03_776847 | C/G | 0.08 | 6.93 | Gbar_D03_811591 | T/C | 0.08 | 7.04 |

**Table S2.** List of the associated SNPs identified in different datasets through GWAS for *FW* resistance in Sea Island cotton. (Continued)

| SNP | Ref/Alt | MAF | -log (p-value) | SNP | Ref/Alt | MAF | -log (p-value) | SNP | Ref/Alt | MAF | -log (p-value) |
| --- | --- | --- | --- | --- | --- | --- | --- | --- | --- | --- | --- |
| **2016** |  |  |  | Gbar_D03_851878 | T/C | 0.08 | 7.16 | Gbar_D03_896764 | A/T | 0.08 | 6.99 |
| Gbar_D03_811659 | C/T | 0.08 | 6.85 | Gbar_D03_852191 | C/T | 0.08 | 7.27 | Gbar_D03_897819 | A/C | 0.08 | 7.13 |
| Gbar_D03_811778 | A/C | 0.08 | 7.19 | Gbar_D03_853726 | A/G | 0.08 | 6.99 | Gbar_D03_897840 | C/G | 0.08 | 6.37 |
| Gbar_D03_812251 | C/G | 0.08 | 7.02 | Gbar_D03_855664 | G/A | 0.08 | 6.84 | Gbar_D03_897899 | G/C | 0.08 | 6.90 |
| Gbar_D03_813482 | C/T | 0.08 | 7.39 | Gbar_D03_855672 | C/T | 0.08 | 6.18 | Gbar_D03_897901 | C/T | 0.08 | 6.89 |
| Gbar_D03_813572 | T/G | 0.08 | 7.06 | Gbar_D03_856654 | C/A | 0.08 | 6.61 | Gbar_D03_898193 | G/A | 0.08 | 7.26 |
| Gbar_D03_813580 | A/C | 0.07 | 6.74 | Gbar_D03_857269 | C/T | 0.08 | 6.83 | Gbar_D03_898245 | G/A | 0.08 | 7.17 |
| Gbar_D03_813730 | T/A | 0.08 | 7.10 | Gbar_D03_857625 | T/C | 0.08 | 6.25 | Gbar_D03_898356 | T/C | 0.08 | 6.33 |
| Gbar_D03_813783 | T/C | 0.08 | 6.78 | Gbar_D03_857695 | T/C | 0.08 | 6.16 | Gbar_D03_898377 | G/A | 0.08 | 6.34 |
| Gbar_D03_813832 | A/G | 0.08 | 6.28 | Gbar_D03_857949 | A/G | 0.08 | 6.64 | Gbar_D03_898709 | A/G | 0.08 | 6.75 |
| Gbar_D03_814065 | A/T | 0.08 | 6.31 | Gbar_D03_858238 | T/C | 0.08 | 6.84 | Gbar_D03_899069 | T/C | 0.08 | 6.50 |
| Gbar_D03_815481 | A/G | 0.08 | 6.92 | Gbar_D03_858711 | C/T | 0.08 | 7.06 | Gbar_D03_899482 | A/G | 0.08 | 6.16 |
| Gbar_D03_815663 | C/T | 0.08 | 6.03 | Gbar_D03_859170 | G/A | 0.08 | 7.08 | Gbar_D03_900042 | T/C | 0.08 | 6.75 |
| Gbar_D03_815732 | C/T | 0.08 | 6.96 | Gbar_D03_859805 | G/A | 0.08 | 6.49 | Gbar_D03_900147 | C/T | 0.08 | 6.85 |
| Gbar_D03_816340 | G/A | 0.08 | 7.52 | Gbar_D03_859825 | C/T | 0.08 | 6.95 | Gbar_D03_900804 | T/C | 0.08 | 7.15 |
| Gbar_D03_817303 | G/A | 0.09 | 7.14 | Gbar_D03_860521 | A/G | 0.08 | 6.93 | Gbar_D03_901197 | G/A | 0.07 | 6.34 |
| Gbar_D03_817476 | G/A | 0.08 | 6.47 | Gbar_D03_860559 | G/A | 0.08 | 6.39 | Gbar_D03_901272 | G/A | 0.08 | 7.05 |
| Gbar_D03_818461 | T/C | 0.08 | 7.22 | Gbar_D03_860585 | C/T | 0.08 | 6.78 | Gbar_D03_901276 | G/T | 0.08 | 6.99 |
| Gbar_D03_818955 | C/A | 0.08 | 6.37 | Gbar_D03_861068 | T/C | 0.08 | 6.70 | Gbar_D03_901536 | C/A | 0.08 | 6.67 |
| Gbar_D03_819316 | C/G | 0.08 | 7.44 | Gbar_D03_861112 | A/G | 0.08 | 7.51 | Gbar_D03_902288 | G/C | 0.08 | 6.90 |
| Gbar_D03_819566 | C/T | 0.08 | 6.88 | Gbar_D03_861183 | T/C | 0.08 | 7.35 | Gbar_D03_902828 | T/C | 0.08 | 6.50 |
| Gbar_D03_820090 | A/C | 0.07 | 6.26 | Gbar_D03_861307 | G/A | 0.08 | 6.73 | Gbar_D03_903181 | G/A | 0.08 | 6.22 |
| Gbar_D03_821496 | T/C | 0.08 | 7.23 | Gbar_D03_861348 | C/T | 0.08 | 6.96 | Gbar_D03_903930 | T/A | 0.08 | 7.66 |
| Gbar_D03_821544 | C/T | 0.08 | 6.74 | Gbar_D03_861362 | A/G | 0.08 | 6.87 | Gbar_D03_903935 | G/A | 0.08 | 7.60 |
| Gbar_D03_822027 | A/T | 0.08 | 7.53 | Gbar_D03_861829 | G/T | 0.08 | 6.29 | Gbar_D03_904409 | G/T | 0.08 | 6.90 |
| Gbar_D03_822378 | A/C | 0.08 | 6.15 | Gbar_D03_861889 | A/G | 0.08 | 6.93 | Gbar_D03_906027 | G/C | 0.08 | 6.78 |
| Gbar_D03_822389 | G/A | 0.08 | 6.15 | Gbar_D03_862149 | C/G | 0.08 | 6.41 | Gbar_D03_906494 | T/C | 0.08 | 7.37 |
| Gbar_D03_823477 | G/T | 0.08 | 6.90 | Gbar_D03_863216 | A/G | 0.08 | 7.05 | Gbar_D03_906561 | A/C | 0.08 | 6.72 |
| Gbar_D03_825160 | G/T | 0.08 | 7.50 | Gbar_D03_863260 | A/T | 0.08 | 6.09 | Gbar_D03_906646 | A/T | 0.08 | 7.18 |
| Gbar_D03_825670 | A/G | 0.08 | 6.77 | Gbar_D03_863288 | T/A | 0.08 | 6.97 | Gbar_D03_906765 | T/G | 0.08 | 7.28 |
| Gbar_D03_826341 | T/C | 0.07 | 6.72 | Gbar_D03_863380 | A/G | 0.08 | 6.79 | Gbar_D03_907447 | A/G | 0.08 | 6.75 |
| Gbar_D03_826386 | A/T | 0.07 | 6.79 | Gbar_D03_863504 | G/T | 0.08 | 6.21 | Gbar_D03_907819 | G/T | 0.08 | 7.30 |
| Gbar_D03_826631 | G/A | 0.08 | 8.32 | Gbar_D03_864223 | T/C | 0.08 | 6.43 | Gbar_D03_907826 | C/T | 0.08 | 7.08 |
| Gbar_D03_826777 | C/T | 0.08 | 7.59 | Gbar_D03_864909 | A/G | 0.08 | 7.06 | Gbar_D03_907859 | G/A | 0.08 | 6.97 |
| Gbar_D03_827276 | G/A | 0.08 | 6.81 | Gbar_D03_865179 | G/A | 0.08 | 6.82 | Gbar_D03_908044 | A/T | 0.08 | 6.69 |
| Gbar_D03_829019 | A/G | 0.08 | 7.05 | Gbar_D03_865347 | T/C | 0.08 | 7.39 | Gbar_D03_908268 | A/G | 0.08 | 7.07 |
| Gbar_D03_829425 | A/T | 0.08 | 7.06 | Gbar_D03_866738 | C/A | 0.08 | 6.53 | Gbar_D03_908768 | A/C | 0.08 | 6.55 |
| Gbar_D03_830950 | A/G | 0.08 | 6.00 | Gbar_D03_866739 | G/T | 0.08 | 6.46 | Gbar_D03_908812 | T/C | 0.08 | 6.39 |
| Gbar_D03_831211 | T/C | 0.08 | 6.15 | Gbar_D03_867612 | T/A | 0.08 | 7.44 | Gbar_D03_909011 | G/C | 0.08 | 6.92 |
| Gbar_D03_831772 | A/G | 0.07 | 6.60 | Gbar_D03_868257 | G/A | 0.08 | 6.46 | Gbar_D03_909073 | C/T | 0.08 | 7.31 |
| Gbar_D03_831844 | C/T | 0.08 | 6.40 | Gbar_D03_868367 | G/A | 0.08 | 7.37 | Gbar_D03_909136 | G/T | 0.08 | 6.54 |
| Gbar_D03_831944 | T/A | 0.08 | 7.18 | Gbar_D03_868494 | G/A | 0.08 | 6.28 | Gbar_D03_910027 | A/T | 0.08 | 6.21 |
| Gbar_D03_832044 | G/A | 0.08 | 7.01 | Gbar_D03_868547 | T/C | 0.08 | 6.94 | Gbar_D03_910165 | T/C | 0.08 | 6.98 |
| Gbar_D03_832128 | T/C | 0.08 | 7.26 | Gbar_D03_869436 | C/A | 0.08 | 6.92 | Gbar_D03_911317 | C/A | 0.08 | 7.00 |
| Gbar_D03_832209 | G/A | 0.08 | 7.26 | Gbar_D03_870721 | G/T | 0.08 | 7.26 | Gbar_D03_911427 | G/A | 0.08 | 7.12 |
| Gbar_D03_832789 | C/G | 0.08 | 7.41 | Gbar_D03_871316 | C/T | 0.08 | 6.55 | Gbar_D03_913182 | C/T | 0.08 | 7.48 |
| Gbar_D03_833775 | T/C | 0.07 | 6.44 | Gbar_D03_872949 | C/T | 0.08 | 7.26 | Gbar_D03_913474 | T/G | 0.08 | 6.08 |
| Gbar_D03_833918 | T/C | 0.08 | 6.98 | Gbar_D03_874004 | T/C | 0.07 | 6.02 | Gbar_D03_913749 | G/A | 0.08 | 6.40 |
| Gbar_D03_834213 | G/A | 0.08 | 7.11 | Gbar_D03_875334 | T/A | 0.08 | 6.79 | Gbar_D03_913869 | G/A | 0.08 | 6.22 |
| Gbar_D03_834357 | G/A | 0.08 | 7.09 | Gbar_D03_876178 | A/G | 0.08 | 6.96 | Gbar_D03_914283 | G/C | 0.08 | 6.79 |
| Gbar_D03_834385 | G/A | 0.08 | 6.41 | Gbar_D03_876290 | G/T | 0.08 | 6.66 | Gbar_D03_915438 | C/G | 0.08 | 7.07 |
| Gbar_D03_834806 | T/C | 0.08 | 7.57 | Gbar_D03_877058 | A/G | 0.09 | 6.17 | Gbar_D03_915814 | T/C | 0.07 | 6.12 |
| Gbar_D03_835978 | A/G | 0.08 | 7.33 | Gbar_D03_877654 | G/A | 0.08 | 6.96 | Gbar_D03_916489 | T/G | 0.08 | 6.61 |
| Gbar_D03_836246 | T/A | 0.08 | 6.99 | Gbar_D03_880446 | C/G | 0.08 | 7.14 | Gbar_D03_917666 | C/T | 0.09 | 6.24 |
| Gbar_D03_836250 | A/G | 0.08 | 6.95 | Gbar_D03_881676 | G/A | 0.08 | 7.47 | Gbar_D03_918390 | G/A | 0.08 | 7.12 |
| Gbar_D03_837176 | G/A | 0.08 | 6.47 | Gbar_D03_882444 | T/A | 0.08 | 6.87 | Gbar_D03_918454 | A/G | 0.08 | 7.63 |
| Gbar_D03_837245 | G/A | 0.08 | 6.90 | Gbar_D03_882817 | T/A | 0.08 | 7.15 | Gbar_D03_919295 | G/T | 0.08 | 6.31 |
| Gbar_D03_837687 | A/T | 0.08 | 6.55 | Gbar_D03_882889 | G/A | 0.08 | 6.36 | Gbar_D03_919316 | A/T | 0.08 | 6.72 |
| Gbar_D03_837695 | A/T | 0.08 | 6.59 | Gbar_D03_883127 | G/A | 0.08 | 6.52 | Gbar_D03_919375 | A/G | 0.07 | 6.24 |
| Gbar_D03_837933 | T/A | 0.08 | 6.96 | Gbar_D03_883704 | C/G | 0.08 | 6.18 | Gbar_D03_919378 | T/C | 0.08 | 6.43 |
| Gbar_D03_837993 | A/C | 0.08 | 7.26 | Gbar_D03_883984 | A/T | 0.08 | 6.24 | Gbar_D03_919408 | C/T | 0.08 | 7.12 |
| Gbar_D03_838032 | G/C | 0.08 | 6.67 | Gbar_D03_884142 | A/G | 0.08 | 6.61 | Gbar_D03_919480 | T/G | 0.08 | 7.33 |
| Gbar_D03_839089 | A/G | 0.08 | 6.88 | Gbar_D03_884147 | A/T | 0.08 | 7.05 | Gbar_D03_919504 | G/A | 0.08 | 6.76 |
| Gbar_D03_839863 | T/C | 0.08 | 7.10 | Gbar_D03_884158 | A/T | 0.08 | 6.89 | Gbar_D03_920104 | A/G | 0.08 | 7.57 |
| Gbar_D03_839876 | T/C | 0.08 | 7.30 | Gbar_D03_884404 | A/C | 0.07 | 6.54 | Gbar_D03_920484 | C/T | 0.08 | 6.24 |
| Gbar_D03_840601 | G/T | 0.08 | 6.49 | Gbar_D03_884492 | A/G | 0.08 | 7.03 | Gbar_D03_922129 | T/C | 0.08 | 6.79 |
| Gbar_D03_840765 | T/C | 0.08 | 6.64 | Gbar_D03_884660 | A/C | 0.08 | 6.19 | Gbar_D03_923381 | C/T | 0.08 | 6.34 |
| Gbar_D03_841312 | C/G | 0.08 | 7.30 | Gbar_D03_885798 | A/T | 0.08 | 7.06 | Gbar_D03_925438 | T/A | 0.08 | 6.87 |
| Gbar_D03_841994 | A/C | 0.08 | 6.91 | Gbar_D03_885841 | C/A | 0.08 | 6.54 | Gbar_D03_925463 | C/T | 0.08 | 7.28 |
| Gbar_D03_842586 | A/G | 0.08 | 6.96 | Gbar_D03_886146 | C/T | 0.08 | 6.01 | Gbar_D03_925948 | C/T | 0.08 | 6.79 |
| Gbar_D03_843572 | A/C | 0.08 | 6.81 | Gbar_D03_886165 | G/A | 0.08 | 6.49 | Gbar_D03_926451 | G/A | 0.08 | 6.02 |
| Gbar_D03_843648 | A/T | 0.08 | 6.80 | Gbar_D03_886259 | C/T | 0.08 | 6.73 | Gbar_D03_926550 | A/G | 0.08 | 6.98 |
| Gbar_D03_843867 | C/T | 0.08 | 7.24 | Gbar_D03_886524 | C/A | 0.08 | 7.07 | Gbar_D03_927253 | A/T | 0.08 | 6.34 |
| Gbar_D03_845453 | A/G | 0.09 | 6.72 | Gbar_D03_886537 | C/T | 0.08 | 6.82 | Gbar_D03_927586 | A/G | 0.08 | 6.06 |
| Gbar_D03_845600 | G/A | 0.09 | 7.04 | Gbar_D03_887470 | G/A | 0.08 | 6.10 | Gbar_D03_927832 | C/T | 0.08 | 6.46 |
| Gbar_D03_845689 | A/G | 0.08 | 7.21 | Gbar_D03_887508 | T/C | 0.08 | 6.19 | Gbar_D03_927886 | C/T | 0.08 | 6.69 |
| Gbar_D03_845829 | G/A | 0.08 | 6.47 | Gbar_D03_887891 | A/G | 0.08 | 6.43 | Gbar_D03_928268 | A/C | 0.08 | 7.13 |
| Gbar_D03_845864 | T/G | 0.08 | 6.85 | Gbar_D03_888250 | T/C | 0.08 | 7.66 | Gbar_D03_928586 | T/A | 0.08 | 7.00 |
| Gbar_D03_845887 | G/A | 0.09 | 7.37 | Gbar_D03_888540 | A/G | 0.08 | 6.56 | Gbar_D03_928931 | A/T | 0.08 | 6.78 |
| Gbar_D03_845918 | C/T | 0.08 | 7.38 | Gbar_D03_888570 | T/C | 0.08 | 6.65 | Gbar_D03_928963 | T/A | 0.08 | 7.02 |
| Gbar_D03_845927 | C/A | 0.08 | 6.95 | Gbar_D03_889027 | C/T | 0.08 | 6.45 | Gbar_D03_929263 | A/C | 0.08 | 6.94 |
| Gbar_D03_845978 | C/T | 0.08 | 6.71 | Gbar_D03_889762 | G/A | 0.08 | 6.83 | Gbar_D03_929354 | C/T | 0.08 | 6.28 |
| Gbar_D03_846044 | C/T | 0.08 | 6.95 | Gbar_D03_889853 | G/A | 0.08 | 7.25 | Gbar_D03_929439 | A/G | 0.08 | 6.86 |
| Gbar_D03_846113 | A/G | 0.08 | 7.97 | Gbar_D03_890328 | G/A | 0.08 | 7.45 | Gbar_D03_929567 | T/C | 0.08 | 6.61 |
| Gbar_D03_846244 | C/T | 0.08 | 7.14 | Gbar_D03_891244 | C/G | 0.09 | 6.19 | Gbar_D03_929578 | C/T | 0.08 | 6.53 |
| Gbar_D03_846411 | G/A | 0.08 | 7.46 | Gbar_D03_891425 | A/T | 0.08 | 7.07 | Gbar_D03_929895 | A/G | 0.08 | 7.20 |
| Gbar_D03_846776 | A/T | 0.08 | 6.78 | Gbar_D03_891465 | T/C | 0.08 | 6.92 | Gbar_D03_929903 | C/T | 0.08 | 7.30 |
| Gbar_D03_846928 | T/C | 0.08 | 6.59 | Gbar_D03_891531 | C/T | 0.08 | 6.28 | Gbar_D03_929941 | T/C | 0.08 | 7.36 |
| Gbar_D03_847147 | G/A | 0.08 | 7.18 | Gbar_D03_891608 | G/A | 0.08 | 6.65 | Gbar_D03_930105 | T/A | 0.08 | 7.22 |
| Gbar_D03_847189 | G/A | 0.08 | 6.72 | Gbar_D03_891771 | A/C | 0.08 | 6.58 | Gbar_D03_930482 | T/A | 0.08 | 6.69 |
| Gbar_D03_847202 | A/T | 0.08 | 6.78 | Gbar_D03_891846 | A/G | 0.08 | 6.76 | Gbar_D03_930562 | G/A | 0.08 | 6.61 |
| Gbar_D03_847223 | A/G | 0.08 | 6.52 | Gbar_D03_892558 | A/G | 0.08 | 6.92 | Gbar_D03_931362 | C/T | 0.08 | 6.07 |
| Gbar_D03_847355 | T/G | 0.08 | 7.47 | Gbar_D03_892638 | A/G | 0.08 | 7.08 | Gbar_D03_931881 | T/A | 0.08 | 6.27 |
| Gbar_D03_847401 | G/A | 0.08 | 6.95 | Gbar_D03_892644 | G/A | 0.08 | 6.85 | Gbar_D03_932359 | G/T | 0.08 | 7.01 |
| Gbar_D03_847995 | G/A | 0.08 | 6.12 | Gbar_D03_892753 | G/A | 0.08 | 6.05 | Gbar_D03_932811 | T/C | 0.08 | 7.39 |
| Gbar_D03_848275 | T/C | 0.07 | 6.06 | Gbar_D03_892901 | T/C | 0.08 | 6.43 | Gbar_D03_932910 | A/G | 0.08 | 6.57 |
| Gbar_D03_848436 | T/A | 0.08 | 6.07 | Gbar_D03_892950 | G/A | 0.08 | 6.03 | Gbar_D03_933036 | A/T | 0.08 | 6.88 |
| Gbar_D03_848499 | C/T | 0.08 | 6.52 | Gbar_D03_893090 | T/C | 0.08 | 6.43 | Gbar_D03_933312 | T/C | 0.08 | 6.06 |
| Gbar_D03_848643 | A/C | 0.08 | 7.67 | Gbar_D03_893103 | C/T | 0.08 | 6.68 | Gbar_D03_933325 | A/G | 0.08 | 6.15 |
| Gbar_D03_848661 | C/T | 0.08 | 7.94 | Gbar_D03_893132 | T/A | 0.08 | 6.46 | Gbar_D03_934354 | G/C | 0.09 | 6.16 |
| Gbar_D03_849103 | C/A | 0.08 | 6.00 | Gbar_D03_893264 | A/G | 0.08 | 6.75 | Gbar_D03_934504 | C/T | 0.08 | 7.02 |
| Gbar_D03_849352 | A/G | 0.08 | 6.63 | Gbar_D03_893355 | C/G | 0.08 | 6.95 | Gbar_D03_934684 | C/T | 0.08 | 6.62 |
| Gbar_D03_849388 | G/A | 0.08 | 6.67 | Gbar_D03_893458 | A/G | 0.08 | 7.07 | Gbar_D03_934831 | A/T | 0.08 | 7.02 |
| Gbar_D03_849439 | A/G | 0.08 | 6.61 | Gbar_D03_893670 | T/A | 0.08 | 6.90 | Gbar_D03_935005 | T/C | 0.08 | 6.66 |
| Gbar_D03_849633 | C/T | 0.08 | 6.33 | Gbar_D03_895012 | T/A | 0.08 | 7.14 | Gbar_D03_935178 | C/T | 0.08 | 6.97 |
| Gbar_D03_850056 | C/A | 0.08 | 7.35 | Gbar_D03_895774 | T/A | 0.08 | 6.67 | Gbar_D03_935248 | G/A | 0.08 | 7.21 |
| Gbar_D03_850089 | C/G | 0.08 | 6.33 | Gbar_D03_895885 | C/T | 0.08 | 6.65 | Gbar_D03_935584 | T/C | 0.08 | 7.09 |
| Gbar_D03_850208 | C/T | 0.08 | 7.38 | Gbar_D03_895969 | A/G | 0.08 | 6.77 | Gbar_D03_936037 | A/T | 0.08 | 6.12 |
| Gbar_D03_850677 | C/A | 0.08 | 6.60 | Gbar_D03_896192 | G/A | 0.08 | 7.14 | Gbar_D03_936082 | T/A | 0.08 | 6.36 |
| Gbar_D03_850678 | T/C | 0.08 | 6.51 | Gbar_D03_896279 | C/T | 0.08 | 6.41 | Gbar_D03_936812 | A/G | 0.08 | 7.09 |

**Table S2.** List of the associated SNPs identified in different datasets through GWAS for *FW* resistance in Sea Island cotton. (Continued)

| SNP | Ref/Alt | MAF | -log (p-value) | SNP | Ref/Alt | MAF | -log (p-value) | SNP | Ref/Alt | MAF | -log (p-value) |
| --- | --- | --- | --- | --- | --- | --- | --- | --- | --- | --- | --- |
| **2016** |  |  |  | Gbar_D03_1930207 | A/G | 0.18 | 6.86 | Gbar_D03_1068871 | A/G | 0.14 | 7.24 |
| Gbar_D03_937095 | T/A | 0.08 | 7.02 | Gbar_D03_1931209 | T/A | 0.18 | 6.58 | Gbar_D03_1075321 | T/C | 0.14 | 6.53 |
| Gbar_D03_937309 | G/T | 0.07 | 6.45 | Gbar_D03_1940049 | T/G | 0.18 | 6.13 | Gbar_D03_1075812 | A/G | 0.15 | 8.75 |
| Gbar_D03_937576 | T/C | 0.08 | 6.59 | Gbar_D03_1956911 | A/T | 0.18 | 6.41 | Gbar_D03_1076207 | A/C | 0.15 | 7.00 |
| Gbar_D03_937673 | A/T | 0.08 | 7.33 | Gbar_D03_1957237 | T/G | 0.09 | 6.14 | Gbar_D03_1076579 | T/C | 0.15 | 7.37 |
| Gbar_D03_937692 | G/A | 0.08 | 7.40 | Gbar_D03_1957546 | A/G | 0.18 | 7.07 | Gbar_D03_1076588 | G/A | 0.15 | 7.68 |
| Gbar_D03_938152 | T/C | 0.08 | 6.04 | Gbar_D03_1963579 | G/A | 0.18 | 6.94 | Gbar_D03_1076691 | A/G | 0.15 | 7.50 |
| Gbar_D03_938167 | A/G | 0.08 | 6.10 | Gbar_D03_1970210 | A/T | 0.18 | 7.76 | Gbar_D03_1084319 | T/C | 0.15 | 7.37 |
| Gbar_D03_938670 | T/C | 0.08 | 6.19 | Gbar_D03_1980162 | A/T | 0.12 | 6.96 | Gbar_D03_1085843 | T/G | 0.15 | 6.72 |
| Gbar_D03_939095 | C/T | 0.08 | 7.46 | Gbar_D03_1980968 | A/G | 0.18 | 6.59 | Gbar_D03_1086685 | C/G | 0.15 | 8.07 |
| Gbar_D03_939478 | G/A | 0.08 | 7.03 | Gbar_D03_1982034 | T/C | 0.18 | 6.30 | Gbar_D03_1087424 | A/C | 0.15 | 7.81 |
| Gbar_D03_940347 | A/T | 0.08 | 6.85 | Gbar_D03_1984614 | G/A | 0.17 | 6.16 | Gbar_D03_1092095 | G/C | 0.16 | 9.02 |
| Gbar_D03_940981 | T/G | 0.08 | 6.30 | Gbar_D03_1984911 | T/C | 0.18 | 7.89 | Gbar_D03_1097512 | G/A | 0.14 | 6.46 |
| Gbar_D03_942540 | A/G | 0.08 | 7.14 | Gbar_D03_1989485 | T/C | 0.18 | 7.55 | Gbar_D03_1099556 | C/T | 0.18 | 8.51 |
| Gbar_D03_942558 | C/T | 0.08 | 7.51 | Gbar_D03_1989700 | A/C | 0.17 | 7.15 | Gbar_D03_1100160 | A/C | 0.16 | 7.80 |
| Gbar_D03_942661 | G/A | 0.08 | 7.08 | Gbar_D03_1990362 | A/G | 0.18 | 7.50 | Gbar_D03_1101128 | A/G | 0.16 | 7.27 |
| Gbar_D03_942735 | A/C | 0.08 | 7.32 | Gbar_D03_2007434 | T/A | 0.18 | 6.75 | Gbar_D03_1101164 | G/A | 0.16 | 7.94 |
| Gbar_D03_942849 | C/A | 0.08 | 7.19 | Gbar_D03_2008527 | A/G | 0.18 | 7.03 | Gbar_D03_1102735 | A/G | 0.15 | 7.04 |
| Gbar_D03_942868 | A/G | 0.08 | 6.36 | Gbar_D03_2014620 | T/C | 0.18 | 7.12 | Gbar_D03_1103325 | T/C | 0.16 | 7.41 |
| Gbar_D03_953927 | G/A | 0.08 | 6.80 | Gbar_D03_2014708 | A/G | 0.17 | 7.45 | Gbar_D03_1105012 | G/T | 0.16 | 8.16 |
| Gbar_D03_959041 | A/G | 0.08 | 7.26 | Gbar_D03_2015383 | G/C | 0.18 | 6.91 | Gbar_D03_1107259 | A/C | 0.16 | 8.42 |
| Gbar_D03_968233 | A/T | 0.08 | 6.57 | Gbar_D03_2015612 | T/C | 0.18 | 7.25 | Gbar_D03_1107653 | T/C | 0.16 | 7.27 |
| Gbar_D03_987798 | G/A | 0.09 | 6.08 | Gbar_D03_2015714 | C/T | 0.18 | 6.39 | Gbar_D03_1107899 | A/G | 0.15 | 6.90 |
| Gbar_D03_987802 | T/G | 0.09 | 6.42 | Gbar_D03_2019774 | A/G | 0.17 | 6.60 | Gbar_D03_1107934 | A/G | 0.16 | 7.32 |
| Gbar_D12_5382642 | T/G | 0.11 | 6.17 | Gbar_D03_2019798 | T/C | 0.18 | 6.77 | Gbar_D03_1108433 | A/G | 0.16 | 7.36 |
| **2018** |  |  |  | Gbar_D03_2020134 | C/A | 0.18 | 6.18 | Gbar_D03_1110567 | A/G | 0.21 | 13.12 |
| Gbar_D03_1050696 | G/T | 0.38 | 9.35 | Gbar_D03_2022329 | C/T | 0.18 | 6.18 | Gbar_D03_1111610 | T/C | 0.16 | 8.00 |
| Gbar_D03_1050724 | G/T | 0.39 | 8.75 | Gbar_D03_2022860 | T/G | 0.18 | 6.46 | Gbar_D03_1112502 | C/G | 0.15 | 6.04 |
| Gbar_D03_1052369 | A/C | 0.37 | 6.81 | Gbar_D03_2022908 | T/C | 0.18 | 7.05 | Gbar_D03_1115166 | A/T | 0.16 | 7.93 |
| Gbar_D03_1053536 | A/T | 0.38 | 9.70 | Gbar_D03_2023405 | T/A | 0.18 | 6.50 | Gbar_D03_1115729 | T/G | 0.16 | 6.41 |
| Gbar_D03_1057322 | C/T | 0.39 | 9.88 | Gbar_D03_2023546 | T/C | 0.18 | 7.87 | Gbar_D03_1116342 | G/A | 0.16 | 8.04 |
| Gbar_D03_1057381 | G/A | 0.39 | 10.17 | Gbar_D03_2025058 | T/C | 0.18 | 6.14 | Gbar_D03_1116402 | A/G | 0.16 | 7.81 |
| Gbar_D03_1057671 | G/T | 0.40 | 6.94 | Gbar_D03_2028897 | T/G | 0.17 | 6.15 | Gbar_D03_1116623 | A/G | 0.15 | 6.51 |
| Gbar_D03_1057751 | G/A | 0.40 | 6.59 | Gbar_D03_2030758 | T/C | 0.18 | 7.05 | Gbar_D03_1117378 | A/G | 0.16 | 8.59 |
| Gbar_D03_1057802 | G/C | 0.38 | 6.77 | Gbar_D03_2031835 | T/G | 0.18 | 7.60 | Gbar_D03_1117759 | A/G | 0.16 | 7.49 |
| Gbar_D03_1057819 | T/C | 0.39 | 7.24 | Gbar_D03_2034483 | T/G | 0.18 | 6.60 | Gbar_D03_1118107 | A/G | 0.16 | 6.62 |
| Gbar_D03_1058771 | T/C | 0.41 | 7.54 | Gbar_D03_2034515 | T/C | 0.18 | 7.21 | Gbar_D03_1118571 | T/C | 0.16 | 7.56 |
| Gbar_D03_1058777 | C/A | 0.41 | 7.32 | Gbar_D03_2034622 | T/C | 0.18 | 7.59 | Gbar_D03_1118866 | A/G | 0.16 | 7.97 |
| Gbar_D03_1058910 | C/T | 0.40 | 8.88 | Gbar_D03_2034913 | T/C | 0.17 | 6.59 | Gbar_D03_1119560 | C/A | 0.15 | 6.09 |
| Gbar_D03_1060248 | G/C | 0.41 | 6.30 | Gbar_D03_2036939 | A/G | 0.17 | 6.10 | Gbar_D03_1119592 | A/G | 0.16 | 6.16 |
| Gbar_D03_1068053 | A/G | 0.15 | 6.82 | Gbar_D03_2037183 | A/G | 0.17 | 6.90 | Gbar_D03_1119718 | G/T | 0.17 | 7.49 |
| Gbar_D03_1075812 | A/G | 0.15 | 6.49 | Gbar_D03_2038069 | A/G | 0.17 | 6.38 | Gbar_D03_1120632 | G/A | 0.17 | 7.09 |
| Gbar_D03_1092095 | G/C | 0.16 | 6.27 | Gbar_D03_2040140 | T/C | 0.18 | 6.73 | Gbar_D03_1121167 | G/A | 0.16 | 7.21 |
| Gbar_D03_1099556 | C/T | 0.18 | 6.03 | Gbar_D03_2040228 | A/G | 0.18 | 6.40 | Gbar_D03_1121203 | T/C | 0.15 | 6.99 |
| Gbar_D03_1100160 | A/C | 0.16 | 6.10 | Gbar_D03_2041970 | A/G | 0.18 | 6.82 | Gbar_D03_1121577 | G/A | 0.16 | 7.82 |
| Gbar_D03_1101164 | G/A | 0.16 | 6.04 | Gbar_D03_2042357 | T/C | 0.18 | 7.57 | Gbar_D03_1125472 | G/C | 0.16 | 7.15 |
| Gbar_D03_1105012 | G/T | 0.16 | 6.64 | Gbar_D03_2043305 | G/A | 0.18 | 7.18 | Gbar_D03_1126238 | T/C | 0.15 | 7.11 |
| Gbar_D03_1107259 | A/C | 0.16 | 6.79 | Gbar_D03_2045627 | G/C | 0.18 | 6.63 | Gbar_D03_1126669 | T/C | 0.16 | 6.95 |
| Gbar_D03_1110567 | A/G | 0.21 | 10.00 | Gbar_D03_2046359 | A/G | 0.18 | 6.34 | Gbar_D03_1142935 | T/C | 0.16 | 7.01 |
| Gbar_D03_1111610 | T/C | 0.16 | 6.44 | Gbar_D03_2046523 | T/C | 0.18 | 7.43 | Gbar_D03_1142946 | C/G | 0.16 | 7.21 |
| Gbar_D03_1115166 | A/T | 0.16 | 6.35 | Gbar_D03_2047187 | A/C | 0.18 | 7.26 | Gbar_D03_1159232 | A/G | 0.16 | 8.52 |
| Gbar_D03_1116342 | G/A | 0.16 | 6.63 | Gbar_D03_2047669 | T/C | 0.18 | 8.03 | Gbar_D03_1159584 | A/G | 0.17 | 7.60 |
| Gbar_D03_1116402 | A/G | 0.16 | 6.23 | Gbar_D03_2047804 | T/C | 0.17 | 7.03 | Gbar_D03_1318456 | A/T | 0.16 | 7.89 |
| Gbar_D03_1117378 | A/G | 0.16 | 6.57 | Gbar_D03_2048430 | A/G | 0.18 | 6.24 | Gbar_D03_1328529 | C/T | 0.14 | 7.09 |
| Gbar_D03_1117759 | A/G | 0.16 | 6.26 | Gbar_D03_2049119 | A/C | 0.18 | 7.98 | Gbar_D03_1329020 | A/G | 0.13 | 7.32 |
| Gbar_D03_1118866 | A/G | 0.16 | 6.17 | Gbar_D03_2049740 | A/G | 0.18 | 6.63 | Gbar_D03_1329117 | C/T | 0.13 | 6.05 |
| Gbar_D03_1121577 | G/A | 0.16 | 6.27 | Gbar_D03_2050294 | G/T | 0.18 | 7.47 | Gbar_D03_1330863 | A/G | 0.13 | 6.65 |
| Gbar_D03_1126238 | T/C | 0.15 | 6.01 | Gbar_D03_2051583 | A/G | 0.18 | 6.23 | Gbar_D03_1331499 | T/C | 0.14 | 6.73 |
| Gbar_D03_1159232 | A/G | 0.16 | 7.06 | Gbar_D03_2051748 | T/C | 0.18 | 6.78 | Gbar_D03_1331651 | A/G | 0.13 | 6.70 |
| Gbar_D03_1318456 | A/T | 0.16 | 6.01 | Gbar_D03_2052908 | A/G | 0.18 | 7.27 | Gbar_D03_1331884 | T/A | 0.13 | 6.26 |
| Gbar_D03_1335353 | G/T | 0.16 | 6.87 | Gbar_D03_2053671 | A/G | 0.17 | 6.22 | Gbar_D03_1332091 | T/G | 0.12 | 6.99 |
| Gbar_D03_1336922 | A/G | 0.16 | 6.50 | Gbar_D03_2054298 | T/C | 0.18 | 7.25 | Gbar_D03_1333123 | A/G | 0.13 | 6.13 |
| Gbar_D03_1337989 | A/G | 0.15 | 6.48 | Gbar_D03_2054754 | T/C | 0.18 | 6.13 | Gbar_D03_1335353 | G/T | 0.16 | 9.20 |
| Gbar_D03_1340760 | A/G | 0.14 | 6.43 | Gbar_D03_2055339 | C/T | 0.18 | 7.31 | Gbar_D03_1336153 | A/G | 0.15 | 8.05 |
| Gbar_D03_1343889 | A/G | 0.16 | 7.33 | Gbar_D03_2057422 | G/A | 0.18 | 7.76 | Gbar_D03_1336922 | A/G | 0.16 | 8.26 |
| Gbar_D03_1344534 | T/C | 0.16 | 6.25 | Gbar_D03_2060260 | C/A | 0.18 | 7.24 | Gbar_D03_1337989 | A/G | 0.15 | 8.75 |
| Gbar_D03_1345643 | T/G | 0.16 | 6.40 | Gbar_D03_2070493 | T/C | 0.18 | 6.72 | Gbar_D03_1340760 | A/G | 0.14 | 7.58 |
| Gbar_D03_1345907 | T/C | 0.16 | 6.57 | Gbar_D03_2070820 | A/T | 0.19 | 6.45 | Gbar_D03_1341476 | G/T | 0.13 | 6.03 |
| Gbar_D03_1347556 | A/C | 0.16 | 6.60 | Gbar_D03_2072242 | A/C | 0.18 | 7.59 | Gbar_D03_1342587 | G/A | 0.16 | 8.40 |
| Gbar_D03_1348970 | A/C | 0.15 | 6.07 | Gbar_D03_2072293 | T/C | 0.18 | 7.86 | Gbar_D03_1343503 | G/A | 0.15 | 8.20 |
| Gbar_D03_1349307 | A/G | 0.15 | 6.38 | Gbar_D03_2073552 | G/A | 0.18 | 6.94 | Gbar_D03_1343889 | A/G | 0.16 | 10.04 |
| Gbar_D03_1629970 | C/T | 0.18 | 8.64 | Gbar_D03_2085912 | T/C | 0.18 | 7.19 | Gbar_D03_1344273 | T/C | 0.16 | 7.82 |
| Gbar_D03_1664579 | T/C | 0.18 | 8.13 | Gbar_D03_2096694 | T/A | 0.18 | 6.09 | Gbar_D03_1344534 | T/C | 0.16 | 8.34 |
| Gbar_D03_1672718 | G/A | 0.19 | 7.88 | Gbar_D03_2214857 | A/G | 0.16 | 7.40 | Gbar_D03_1345643 | T/G | 0.16 | 8.23 |
| Gbar_D03_1676638 | A/C | 0.19 | 7.82 | Gbar_D03_2215669 | A/T | 0.17 | 7.89 | Gbar_D03_1345907 | T/C | 0.16 | 8.80 |
| Gbar_D03_1703450 | T/A | 0.17 | 7.66 | Gbar_D03_2222499 | T/C | 0.16 | 6.97 | Gbar_D03_1347556 | A/C | 0.16 | 8.84 |
| Gbar_D03_1822281 | G/A | 0.18 | 6.59 | Gbar_D03_2222921 | A/G | 0.17 | 7.44 | Gbar_D03_1348970 | A/C | 0.15 | 8.16 |
| Gbar_D03_1824274 | C/T | 0.18 | 6.74 | Gbar_D03_2224222 | A/G | 0.20 | 7.06 | Gbar_D03_1349307 | A/G | 0.15 | 8.66 |
| Gbar_D03_1824622 | T/C | 0.18 | 6.23 | Gbar_D03_2224946 | A/G | 0.16 | 6.96 | Gbar_D03_1352685 | T/C | 0.14 | 6.92 |
| Gbar_D03_1824899 | T/C | 0.18 | 8.10 | Gbar_D03_2224965 | A/G | 0.17 | 7.23 | Gbar_D03_1354369 | T/G | 0.16 | 8.16 |
| Gbar_D03_1826623 | C/T | 0.18 | 7.28 | Gbar_D03_2228451 | A/G | 0.14 | 6.62 | Gbar_D03_1354636 | T/C | 0.13 | 6.91 |
| Gbar_D03_1828505 | T/A | 0.18 | 6.85 | Gbar_D03_2249026 | C/T | 0.09 | 6.01 | Gbar_D03_1354803 | T/C | 0.13 | 6.73 |
| Gbar_D03_1843065 | T/G | 0.17 | 7.56 | Gbar_D03_2249706 | T/C | 0.15 | 6.89 | Gbar_D03_1355184 | T/C | 0.14 | 6.35 |
| Gbar_D03_1846306 | T/G | 0.18 | 6.66 | Gbar_D03_2255845 | T/G | 0.14 | 6.79 | Gbar_D03_1357765 | A/T | 0.13 | 6.24 |
| Gbar_D03_1849825 | T/C | 0.17 | 6.32 | Gbar_D03_2256560 | T/A | 0.14 | 6.73 | Gbar_D03_1629970 | C/T | 0.18 | 11.52 |
| Gbar_D03_1855104 | A/G | 0.17 | 6.52 | Gbar_D05_54270545 | T/A | 0.06 | 6.32 | Gbar_D03_1664579 | T/C | 0.18 | 10.44 |
| Gbar_D03_1856190 | G/A | 0.17 | 6.21 | **2019** |  |  |  | Gbar_D03_1672718 | G/A | 0.19 | 10.24 |
| Gbar_D03_1857894 | A/G | 0.18 | 7.33 | Gbar_A02_98595082 | T/A | 0.44 | 6.06 | Gbar_D03_1676638 | A/C | 0.19 | 10.45 |
| Gbar_D03_1858765 | G/A | 0.18 | 7.14 | Gbar_A11_89241215 | T/G | 0.15 | 6.26 | Gbar_D03_1703450 | T/A | 0.17 | 8.72 |
| Gbar_D03_1860357 | T/C | 0.18 | 7.52 | Gbar_A11_89242365 | G/A | 0.12 | 6.41 | Gbar_D03_1822281 | G/A | 0.18 | 6.96 |
| Gbar_D03_1872855 | G/C | 0.18 | 7.40 | Gbar_A11_89242384 | T/C | 0.11 | 6.04 | Gbar_D03_1824274 | C/T | 0.18 | 7.24 |
| Gbar_D03_1873143 | T/C | 0.17 | 6.36 | Gbar_A13_40120505 | A/C | 0.12 | 6.15 | Gbar_D03_1824899 | T/C | 0.18 | 8.29 |
| Gbar_D03_1879874 | T/C | 0.09 | 6.25 | Gbar_D03_1002381 | T/C | 0.13 | 6.36 | Gbar_D03_1826623 | C/T | 0.18 | 7.51 |
| Gbar_D03_1882031 | T/C | 0.18 | 6.67 | Gbar_D03_1009181 | G/A | 0.13 | 6.75 | Gbar_D03_1828505 | T/A | 0.18 | 6.86 |
| Gbar_D03_1886621 | A/G | 0.18 | 6.55 | Gbar_D03_1022935 | T/G | 0.14 | 6.17 | Gbar_D03_1843065 | T/G | 0.17 | 7.30 |
| Gbar_D03_1890205 | T/G | 0.18 | 7.40 | Gbar_D03_1024618 | A/G | 0.15 | 6.63 | Gbar_D03_1846306 | T/G | 0.18 | 7.03 |
| Gbar_D03_1892354 | T/G | 0.18 | 7.15 | Gbar_D03_1024724 | T/C | 0.15 | 6.35 | Gbar_D03_1847461 | A/G | 0.17 | 6.94 |
| Gbar_D03_1896485 | T/C | 0.17 | 7.41 | Gbar_D03_1025046 | C/T | 0.14 | 6.46 | Gbar_D03_1849825 | T/C | 0.17 | 6.96 |
| Gbar_D03_1897259 | T/C | 0.17 | 6.24 | Gbar_D03_1029916 | T/C | 0.14 | 6.84 | Gbar_D03_1850013 | T/C | 0.17 | 6.48 |
| Gbar_D03_1901449 | G/A | 0.18 | 6.22 | Gbar_D03_1036297 | T/G | 0.14 | 6.97 | Gbar_D03_1855104 | A/G | 0.17 | 6.90 |
| Gbar_D03_1903013 | T/C | 0.18 | 6.84 | Gbar_D03_1042425 | A/T | 0.13 | 6.07 | Gbar_D03_1857894 | A/G | 0.18 | 6.67 |
| Gbar_D03_1904562 | C/T | 0.17 | 6.47 | Gbar_D03_1044462 | G/A | 0.14 | 6.07 | Gbar_D03_1858765 | G/A | 0.18 | 7.41 |
| Gbar_D03_1904856 | A/G | 0.18 | 6.87 | Gbar_D03_1058447 | A/G | 0.15 | 7.06 | Gbar_D03_1860357 | T/C | 0.18 | 7.95 |
| Gbar_D03_1912152 | T/C | 0.17 | 6.52 | Gbar_D03_1061196 | T/A | 0.15 | 6.86 | Gbar_D03_1872855 | G/C | 0.18 | 7.68 |
| Gbar_D03_1918313 | T/C | 0.15 | 6.26 | Gbar_D03_1061302 | A/G | 0.15 | 6.29 | Gbar_D03_1873143 | T/C | 0.17 | 6.44 |
| Gbar_D03_1921525 | T/C | 0.17 | 6.35 | Gbar_D03_1062012 | G/A | 0.15 | 7.85 | Gbar_D03_1879874 | T/C | 0.09 | 6.22 |
| Gbar_D03_1922876 | T/C | 0.18 | 7.86 | Gbar_D03_1063181 | T/C | 0.15 | 7.51 | Gbar_D03_1881025 | G/A | 0.09 | 6.01 |
| Gbar_D03_1923348 | C/A | 0.18 | 6.98 | Gbar_D03_1065012 | A/G | 0.14 | 6.53 | Gbar_D03_1882031 | T/C | 0.18 | 6.55 |
| Gbar_D03_1924417 | A/G | 0.17 | 6.63 | Gbar_D03_1067829 | A/G | 0.15 | 7.94 | Gbar_D03_1886621 | A/G | 0.18 | 6.98 |
| Gbar_D03_1928629 | T/C | 0.18 | 7.82 | Gbar_D03_1068053 | A/G | 0.15 | 8.86 | Gbar_D03_1890205 | T/G | 0.18 | 7.20 |

**Table S2.** List of the associated SNPs identified in different datasets through GWAS for *FW* resistance in Sea Island cotton. (Continued)

| SNP | Ref/Alt | MAF | -log (p-value) | SNP | Ref/Alt | MAF | -log (p-value) | SNP | Ref/Alt | MAF | -log (p-value) |
| --- | --- | --- | --- | --- | --- | --- | --- | --- | --- | --- | --- |
| **2019** |  |  |  | **Mean** |  |  |  | Gbar_D03_1703450 | T/A | 0.17 | 8.03 |
| Gbar_D03_1892354 | T/G | 0.18 | 7.77 | Gbar_A07_82872256 | A/T | 0.13 | 6.62 | Gbar_D03_1822281 | G/A | 0.18 | 6.08 |
| Gbar_D03_1896485 | T/C | 0.17 | 7.00 | Gbar_A11_89241215 | T/G | 0.15 | 6.94 | Gbar_D03_1824274 | C/T | 0.18 | 6.33 |
| Gbar_D03_1897259 | T/C | 0.17 | 6.25 | Gbar_A11_89242281 | G/A | 0.13 | 6.07 | Gbar_D03_1824899 | T/C | 0.18 | 7.51 |
| Gbar_D03_1901449 | G/A | 0.18 | 6.60 | Gbar_A11_89242339 | A/T | 0.13 | 6.05 | Gbar_D03_1826623 | C/T | 0.18 | 6.60 |
| Gbar_D03_1903013 | T/C | 0.18 | 7.21 | Gbar_A11_89242355 | T/A | 0.12 | 6.35 | Gbar_D03_1828505 | T/A | 0.18 | 6.15 |
| Gbar_D03_1904562 | C/T | 0.17 | 6.82 | Gbar_A11_89242365 | G/A | 0.12 | 6.97 | Gbar_D03_1843065 | T/G | 0.17 | 6.37 |
| Gbar_D03_1904856 | A/G | 0.18 | 7.37 | Gbar_A11_89242374 | T/C | 0.13 | 6.40 | Gbar_D03_1846306 | T/G | 0.18 | 6.32 |
| Gbar_D03_1907553 | T/A | 0.20 | 6.08 | Gbar_A11_89242384 | T/C | 0.11 | 7.14 | Gbar_D03_1847461 | A/G | 0.17 | 6.18 |
| Gbar_D03_1912152 | T/C | 0.17 | 6.76 | Gbar_A11_89242399 | C/T | 0.11 | 6.25 | Gbar_D03_1849825 | T/C | 0.17 | 6.40 |
| Gbar_D03_1921525 | T/C | 0.17 | 6.82 | Gbar_A13_40120505 | A/C | 0.12 | 6.33 | Gbar_D03_1855104 | A/G | 0.17 | 6.23 |
| Gbar_D03_1922876 | T/C | 0.18 | 7.73 | Gbar_D03_1002381 | T/C | 0.13 | 6.39 | Gbar_D03_1858765 | G/A | 0.18 | 6.46 |
| Gbar_D03_1923348 | C/A | 0.18 | 7.30 | Gbar_D03_1009181 | G/A | 0.13 | 6.84 | Gbar_D03_1860357 | T/C | 0.18 | 6.92 |
| Gbar_D03_1924417 | A/G | 0.17 | 7.08 | Gbar_D03_1022935 | T/G | 0.14 | 6.01 | Gbar_D03_1872855 | G/C | 0.18 | 6.83 |
| Gbar_D03_1928629 | T/C | 0.18 | 7.75 | Gbar_D03_1024618 | A/G | 0.15 | 6.59 | Gbar_D03_1878030 | T/C | 0.08 | 6.11 |
| Gbar_D03_1930207 | A/G | 0.18 | 7.01 | Gbar_D03_1024724 | T/C | 0.15 | 6.22 | Gbar_D03_1879874 | T/C | 0.09 | 6.24 |
| Gbar_D03_1931209 | T/A | 0.18 | 6.95 | Gbar_D03_1025046 | C/T | 0.14 | 6.31 | Gbar_D03_1881025 | G/A | 0.09 | 6.01 |
| Gbar_D03_1940049 | T/G | 0.18 | 6.57 | Gbar_D03_1029916 | T/C | 0.14 | 6.63 | Gbar_D03_1882031 | T/C | 0.18 | 6.15 |
| Gbar_D03_1956911 | A/T | 0.18 | 6.99 | Gbar_D03_1036297 | T/G | 0.14 | 6.93 | Gbar_D03_1886621 | A/G | 0.18 | 6.23 |
| Gbar_D03_1957546 | A/G | 0.18 | 7.41 | Gbar_D03_1042425 | A/T | 0.13 | 6.16 | Gbar_D03_1890205 | T/G | 0.18 | 6.29 |
| Gbar_D03_1963579 | G/A | 0.18 | 7.52 | Gbar_D03_1058447 | A/G | 0.15 | 7.12 | Gbar_D03_1892354 | T/G | 0.18 | 6.94 |
| Gbar_D03_1965407 | A/G | 0.17 | 6.53 | Gbar_D03_1061196 | T/A | 0.15 | 6.64 | Gbar_D03_1896485 | T/C | 0.17 | 6.00 |
| Gbar_D03_1970210 | A/T | 0.18 | 7.72 | Gbar_D03_1061302 | A/G | 0.15 | 6.35 | Gbar_D03_1903013 | T/C | 0.18 | 6.41 |
| Gbar_D03_1975755 | A/G | 0.18 | 6.44 | Gbar_D03_1062012 | G/A | 0.15 | 7.56 | Gbar_D03_1904856 | A/G | 0.18 | 6.45 |
| Gbar_D03_1980162 | A/T | 0.12 | 7.53 | Gbar_D03_1063181 | T/C | 0.15 | 7.35 | Gbar_D03_1912152 | T/C | 0.17 | 6.13 |
| Gbar_D03_1980968 | A/G | 0.18 | 6.86 | Gbar_D03_1065012 | A/G | 0.14 | 6.42 | Gbar_D03_1922876 | T/C | 0.18 | 6.70 |
| Gbar_D03_1982034 | T/C | 0.18 | 6.63 | Gbar_D03_1067829 | A/G | 0.15 | 7.52 | Gbar_D03_1923348 | C/A | 0.18 | 6.41 |
| Gbar_D03_1984614 | G/A | 0.17 | 6.28 | Gbar_D03_1068053 | A/G | 0.15 | 8.30 | Gbar_D03_1924417 | A/G | 0.17 | 6.11 |
| Gbar_D03_1984911 | T/C | 0.18 | 7.99 | Gbar_D03_1068871 | A/G | 0.14 | 6.68 | Gbar_D03_1928629 | T/C | 0.18 | 6.82 |
| Gbar_D03_1989485 | T/C | 0.18 | 7.75 | Gbar_D03_1075321 | T/C | 0.14 | 6.70 | Gbar_D03_1930207 | A/G | 0.18 | 6.13 |
| Gbar_D03_1989700 | A/C | 0.17 | 7.41 | Gbar_D03_1075812 | A/G | 0.15 | 8.29 | Gbar_D03_1931209 | T/A | 0.18 | 6.24 |
| Gbar_D03_1990362 | A/G | 0.18 | 7.80 | Gbar_D03_1076207 | A/C | 0.15 | 6.41 | Gbar_D03_1941440 | A/T | 0.09 | 6.02 |
| Gbar_D03_2007434 | T/A | 0.18 | 7.24 | Gbar_D03_1076579 | T/C | 0.15 | 7.19 | Gbar_D03_1956911 | A/T | 0.18 | 6.01 |
| Gbar_D03_2008527 | A/G | 0.18 | 7.53 | Gbar_D03_1076588 | G/A | 0.15 | 7.54 | Gbar_D03_1957546 | A/G | 0.18 | 6.62 |
| Gbar_D03_2014620 | T/C | 0.18 | 7.58 | Gbar_D03_1076691 | A/G | 0.15 | 7.05 | Gbar_D03_1963579 | G/A | 0.18 | 6.51 |
| Gbar_D03_2014708 | A/G | 0.17 | 6.95 | Gbar_D03_1084319 | T/C | 0.15 | 6.90 | Gbar_D03_1970210 | A/T | 0.18 | 6.76 |
| Gbar_D03_2015383 | G/C | 0.18 | 7.04 | Gbar_D03_1085843 | T/G | 0.15 | 6.25 | Gbar_D03_1980162 | A/T | 0.12 | 6.99 |
| Gbar_D03_2015612 | T/C | 0.18 | 7.51 | Gbar_D03_1086685 | C/G | 0.15 | 7.66 | Gbar_D03_1980968 | A/G | 0.18 | 6.41 |
| Gbar_D03_2015714 | C/T | 0.18 | 6.64 | Gbar_D03_1087424 | A/C | 0.15 | 7.37 | Gbar_D03_1984911 | T/C | 0.18 | 6.94 |
| Gbar_D03_2019774 | A/G | 0.17 | 7.10 | Gbar_D03_1092095 | G/C | 0.16 | 8.57 | Gbar_D03_1989485 | T/C | 0.18 | 7.11 |
| Gbar_D03_2019798 | T/C | 0.18 | 7.28 | Gbar_D03_1099556 | C/T | 0.18 | 7.72 | Gbar_D03_1989700 | A/C | 0.17 | 6.57 |
| Gbar_D03_2020134 | C/A | 0.18 | 6.58 | Gbar_D03_1100160 | A/C | 0.16 | 7.45 | Gbar_D03_1990362 | A/G | 0.18 | 6.88 |
| Gbar_D03_2022329 | C/T | 0.18 | 6.23 | Gbar_D03_1101128 | A/G | 0.16 | 7.18 | Gbar_D03_2007434 | T/A | 0.18 | 6.39 |
| Gbar_D03_2022860 | T/G | 0.18 | 6.84 | Gbar_D03_1101164 | G/A | 0.16 | 7.74 | Gbar_D03_2008527 | A/G | 0.18 | 6.58 |
| Gbar_D03_2022908 | T/C | 0.18 | 7.30 | Gbar_D03_1102735 | A/G | 0.15 | 6.64 | Gbar_D03_2014620 | T/C | 0.18 | 6.66 |
| Gbar_D03_2023153 | C/T | 0.18 | 6.28 | Gbar_D03_1103325 | T/C | 0.16 | 6.96 | Gbar_D03_2014708 | A/G | 0.17 | 6.02 |
| Gbar_D03_2023405 | T/A | 0.18 | 6.67 | Gbar_D03_1105012 | G/T | 0.16 | 8.14 | Gbar_D03_2015383 | G/C | 0.18 | 6.22 |
| Gbar_D03_2023546 | T/C | 0.18 | 8.05 | Gbar_D03_1107259 | A/C | 0.16 | 8.07 | Gbar_D03_2015612 | T/C | 0.18 | 6.56 |
| Gbar_D03_2025058 | T/C | 0.18 | 6.91 | Gbar_D03_1107653 | T/C | 0.16 | 6.98 | Gbar_D03_2019774 | A/G | 0.17 | 6.22 |
| Gbar_D03_2028897 | T/G | 0.17 | 6.44 | Gbar_D03_1107899 | A/G | 0.15 | 6.47 | Gbar_D03_2019798 | T/C | 0.18 | 6.22 |
| Gbar_D03_2030758 | T/C | 0.18 | 7.23 | Gbar_D03_1107934 | A/G | 0.16 | 7.04 | Gbar_D03_2022908 | T/C | 0.18 | 6.46 |
| Gbar_D03_2031835 | T/G | 0.18 | 8.09 | Gbar_D03_1108433 | A/G | 0.16 | 7.13 | Gbar_D03_2023546 | T/C | 0.18 | 7.11 |
| Gbar_D03_2034483 | T/G | 0.18 | 6.85 | Gbar_D03_1110567 | A/G | 0.21 | 11.01 | Gbar_D03_2025058 | T/C | 0.18 | 6.42 |
| Gbar_D03_2034515 | T/C | 0.18 | 7.06 | Gbar_D03_1111610 | T/C | 0.16 | 7.69 | Gbar_D03_2030758 | T/C | 0.18 | 6.42 |
| Gbar_D03_2034622 | T/C | 0.18 | 7.61 | Gbar_D03_1112502 | C/G | 0.15 | 6.01 | Gbar_D03_2031835 | T/G | 0.18 | 7.17 |
| Gbar_D03_2034913 | T/C | 0.17 | 6.96 | Gbar_D03_1115166 | A/T | 0.16 | 7.44 | Gbar_D03_2034483 | T/G | 0.18 | 6.03 |
| Gbar_D03_2036939 | A/G | 0.17 | 6.45 | Gbar_D03_1116342 | G/A | 0.16 | 7.70 | Gbar_D03_2034515 | T/C | 0.18 | 6.15 |
| Gbar_D03_2037183 | A/G | 0.17 | 6.71 | Gbar_D03_1116402 | A/G | 0.16 | 7.46 | Gbar_D03_2034622 | T/C | 0.18 | 6.61 |
| Gbar_D03_2038069 | A/G | 0.17 | 6.63 | Gbar_D03_1116623 | A/G | 0.15 | 6.33 | Gbar_D03_2034913 | T/C | 0.17 | 6.02 |
| Gbar_D03_2039439 | A/G | 0.18 | 6.70 | Gbar_D03_1117378 | A/G | 0.16 | 8.11 | Gbar_D03_2040140 | T/C | 0.18 | 6.09 |
| Gbar_D03_2040140 | T/C | 0.18 | 6.95 | Gbar_D03_1117759 | A/G | 0.16 | 7.38 | Gbar_D03_2040228 | A/G | 0.18 | 6.03 |
| Gbar_D03_2040228 | A/G | 0.18 | 6.79 | Gbar_D03_1118107 | A/G | 0.16 | 6.43 | Gbar_D03_2041970 | A/G | 0.18 | 6.33 |
| Gbar_D03_2041970 | A/G | 0.18 | 7.22 | Gbar_D03_1118571 | T/C | 0.16 | 7.25 | Gbar_D03_2042357 | T/C | 0.18 | 6.79 |
| Gbar_D03_2042357 | T/C | 0.18 | 7.69 | Gbar_D03_1118866 | A/G | 0.16 | 7.66 | Gbar_D03_2043305 | G/A | 0.18 | 6.71 |
| Gbar_D03_2043305 | G/A | 0.18 | 7.54 | Gbar_D03_1119718 | G/T | 0.17 | 7.24 | Gbar_D03_2046359 | A/G | 0.18 | 6.14 |
| Gbar_D03_2045627 | G/C | 0.18 | 6.71 | Gbar_D03_1120632 | G/A | 0.17 | 6.79 | Gbar_D03_2047187 | A/C | 0.18 | 6.40 |
| Gbar_D03_2046359 | A/G | 0.18 | 6.83 | Gbar_D03_1121167 | G/A | 0.16 | 7.08 | Gbar_D03_2047669 | T/C | 0.18 | 7.39 |
| Gbar_D03_2046523 | T/C | 0.18 | 6.84 | Gbar_D03_1121203 | T/C | 0.15 | 6.88 | Gbar_D03_2047804 | T/C | 0.17 | 6.51 |
| Gbar_D03_2047187 | A/C | 0.18 | 7.33 | Gbar_D03_1121577 | G/A | 0.16 | 7.41 | Gbar_D03_2049119 | A/C | 0.18 | 7.03 |
| Gbar_D03_2047669 | T/C | 0.18 | 8.19 | Gbar_D03_1125472 | G/C | 0.16 | 7.05 | Gbar_D03_2049740 | A/G | 0.18 | 6.10 |
| Gbar_D03_2047730 | T/C | 0.17 | 6.12 | Gbar_D03_1126238 | T/C | 0.15 | 7.01 | Gbar_D03_2050294 | G/T | 0.18 | 7.03 |
| Gbar_D03_2047804 | T/C | 0.17 | 7.24 | Gbar_D03_1126669 | T/C | 0.16 | 6.57 | Gbar_D03_2051748 | T/C | 0.18 | 6.39 |
| Gbar_D03_2048430 | A/G | 0.18 | 6.64 | Gbar_D03_1142935 | T/C | 0.16 | 6.37 | Gbar_D03_2052908 | A/G | 0.18 | 6.36 |
| Gbar_D03_2049119 | A/C | 0.18 | 7.96 | Gbar_D03_1142946 | C/G | 0.16 | 6.76 | Gbar_D03_2054298 | T/C | 0.18 | 6.51 |
| Gbar_D03_2049740 | A/G | 0.18 | 7.08 | Gbar_D03_1159232 | A/G | 0.16 | 7.97 | Gbar_D03_2055339 | C/T | 0.18 | 6.85 |
| Gbar_D03_2050294 | G/T | 0.18 | 7.89 | Gbar_D03_1159584 | A/G | 0.17 | 7.28 | Gbar_D03_2057422 | G/A | 0.18 | 6.90 |
| Gbar_D03_2051583 | A/G | 0.18 | 6.50 | Gbar_D03_1318456 | A/T | 0.16 | 7.28 | Gbar_D03_2060260 | C/A | 0.18 | 8.14 |
| Gbar_D03_2051748 | T/C | 0.18 | 7.18 | Gbar_D03_1328529 | C/T | 0.14 | 7.05 | Gbar_D03_2070493 | T/C | 0.18 | 6.11 |
| Gbar_D03_2052908 | A/G | 0.18 | 7.31 | Gbar_D03_1329020 | A/G | 0.13 | 7.39 | Gbar_D03_2070820 | A/T | 0.19 | 6.37 |
| Gbar_D03_2053671 | A/G | 0.17 | 6.53 | Gbar_D03_1329117 | C/T | 0.13 | 6.20 | Gbar_D03_2072242 | A/C | 0.18 | 6.69 |
| Gbar_D03_2054298 | T/C | 0.18 | 7.44 | Gbar_D03_1330863 | A/G | 0.13 | 6.57 | Gbar_D03_2072293 | T/C | 0.18 | 7.01 |
| Gbar_D03_2054754 | T/C | 0.18 | 6.25 | Gbar_D03_1331499 | T/C | 0.14 | 6.75 | Gbar_D03_2073552 | G/A | 0.18 | 6.52 |
| Gbar_D03_2055339 | C/T | 0.18 | 7.72 | Gbar_D03_1331651 | A/G | 0.13 | 6.71 | Gbar_D03_2085912 | T/C | 0.18 | 6.67 |
| Gbar_D03_2057422 | G/A | 0.18 | 7.84 | Gbar_D03_1331884 | T/A | 0.13 | 6.20 | Gbar_D03_2214857 | A/G | 0.16 | 7.98 |
| Gbar_D03_2060260 | C/A | 0.18 | 9.13 | Gbar_D03_1332091 | T/G | 0.12 | 7.06 | Gbar_D03_2215669 | A/T | 0.17 | 8.42 |
| Gbar_D03_2070493 | T/C | 0.18 | 6.87 | Gbar_D03_1333123 | A/G | 0.13 | 6.22 | Gbar_D03_2222499 | T/C | 0.16 | 7.86 |
| Gbar_D03_2070820 | A/T | 0.19 | 7.28 | Gbar_D03_1335353 | G/T | 0.16 | 8.44 | Gbar_D03_2222921 | A/G | 0.17 | 7.76 |
| Gbar_D03_2072242 | A/C | 0.18 | 7.73 | Gbar_D03_1336153 | A/G | 0.15 | 7.12 | Gbar_D03_2224222 | A/G | 0.20 | 8.40 |
| Gbar_D03_2072293 | T/C | 0.18 | 8.06 | Gbar_D03_1336922 | A/G | 0.16 | 7.56 | Gbar_D03_2224946 | A/G | 0.16 | 7.87 |
| Gbar_D03_2073552 | G/A | 0.18 | 7.38 | Gbar_D03_1337989 | A/G | 0.15 | 7.67 | Gbar_D03_2224965 | A/G | 0.17 | 8.07 |
| Gbar_D03_2085912 | T/C | 0.18 | 7.68 | Gbar_D03_1340760 | A/G | 0.14 | 7.19 | Gbar_D03_2228451 | A/G | 0.14 | 7.83 |
| Gbar_D03_2096694 | T/A | 0.18 | 6.38 | Gbar_D03_1342587 | G/A | 0.16 | 7.59 | Gbar_D03_2249706 | T/C | 0.15 | 7.91 |
| Gbar_D03_2214857 | A/G | 0.16 | 8.58 | Gbar_D03_1343503 | G/A | 0.15 | 7.43 | Gbar_D03_2255845 | T/G | 0.14 | 7.47 |
| Gbar_D03_2215669 | A/T | 0.17 | 9.01 | Gbar_D03_1343889 | A/G | 0.16 | 8.93 | Gbar_D03_2255926 | T/C | 0.14 | 6.69 |
| Gbar_D03_2222499 | T/C | 0.16 | 8.07 | Gbar_D03_1344273 | T/C | 0.16 | 7.04 | Gbar_D03_2256558 | T/A | 0.14 | 7.26 |
| Gbar_D03_2222921 | A/G | 0.17 | 8.44 | Gbar_D03_1344534 | T/C | 0.16 | 7.49 | Gbar_D03_2256560 | T/A | 0.14 | 7.83 |
| Gbar_D03_2224222 | A/G | 0.20 | 8.90 | Gbar_D03_1345643 | T/G | 0.16 | 7.18 | Gbar_D03_2289243 | A/G | 0.14 | 6.49 |
| Gbar_D03_2224946 | A/G | 0.16 | 8.18 | Gbar_D03_1345907 | T/C | 0.16 | 7.83 | Gbar_D03_2368579 | T/A | 0.17 | 6.43 |
| Gbar_D03_2224965 | A/G | 0.17 | 8.56 | Gbar_D03_1347556 | A/C | 0.16 | 7.84 | Gbar_D03_809907 | T/A | 0.08 | 6.03 |
| Gbar_D03_2228451 | A/G | 0.14 | 8.01 | Gbar_D03_1348970 | A/C | 0.15 | 7.34 | Gbar_D03_993066 | T/C | 0.13 | 6.25 |
| Gbar_D03_2249706 | T/C | 0.15 | 7.98 | Gbar_D03_1349307 | A/G | 0.15 | 7.67 | **BLUP** |  |  |  |
| Gbar_D03_2255845 | T/G | 0.14 | 7.81 | Gbar_D03_1352685 | T/C | 0.14 | 6.60 | Gbar_A02_98595082 | T/A | 0.44 | 6.16 |
| Gbar_D03_2255926 | T/C | 0.14 | 6.80 | Gbar_D03_1354369 | T/G | 0.16 | 7.61 | Gbar_A07_82872256 | A/T | 0.13 | 6.11 |
| Gbar_D03_2256558 | T/A | 0.14 | 7.43 | Gbar_D03_1354636 | T/C | 0.13 | 6.45 | Gbar_A11_89241215 | T/G | 0.15 | 6.54 |
| Gbar_D03_2256560 | T/A | 0.14 | 8.07 | Gbar_D03_1354803 | T/C | 0.13 | 6.40 | Gbar_A11_89242365 | G/A | 0.12 | 6.53 |
| Gbar_D03_2289243 | A/G | 0.14 | 6.54 | Gbar_D03_1355184 | T/C | 0.14 | 6.14 | Gbar_A11_89242384 | T/C | 0.11 | 6.53 |
| Gbar_D03_2368579 | T/A | 0.17 | 6.63 | Gbar_D03_1629970 | C/T | 0.18 | 10.10 | Gbar_A13_40120505 | A/C | 0.12 | 6.23 |
| Gbar_D03_993066 | T/C | 0.13 | 6.15 | Gbar_D03_1664579 | T/C | 0.18 | 8.89 | Gbar_D03_1002381 | T/C | 0.13 | 6.58 |
| Gbar_D03_995976 | T/C | 0.13 | 6.05 | Gbar_D03_1672718 | G/A | 0.19 | 8.86 | Gbar_D03_1009181 | G/A | 0.13 | 7.04 |
| Gbar_D06_44882448 | C/A | 0.17 | 6.19 | Gbar_D03_1676638 | A/C | 0.19 | 9.06 | Gbar_D03_1022935 | T/G | 0.14 | 6.19 |

**Table S2.** List of the associated SNPs identified in different datasets through GWAS for *FW* resistance in Sea Island cotton. (Continued)

| SNP | Ref/Alt | MAF | -log (p-value) | SNP | Ref/Alt | MAF | -log (p-value) | SNP | Ref/Alt | MAF | -log (p-value) |
| --- | --- | --- | --- | --- | --- | --- | --- | --- | --- | --- | --- |
| **BLUP** |  |  |  | Gbar_D03_1342587 | G/A | 0.16 | 7.90 | Gbar_D03_2020134 | C/A | 0.18 | 6.08 |
| Gbar_D03_1024618 | A/G | 0.15 | 6.76 | Gbar_D03_1343503 | G/A | 0.15 | 7.71 | Gbar_D03_2022860 | T/G | 0.18 | 6.11 |
| Gbar_D03_1024724 | T/C | 0.15 | 6.42 | Gbar_D03_1343889 | A/G | 0.16 | 9.31 | Gbar_D03_2022908 | T/C | 0.18 | 6.78 |
| Gbar_D03_1025046 | C/T | 0.14 | 6.50 | Gbar_D03_1344273 | T/C | 0.16 | 7.34 | Gbar_D03_2023405 | T/A | 0.18 | 6.29 |
| Gbar_D03_1029916 | T/C | 0.14 | 6.84 | Gbar_D03_1344534 | T/C | 0.16 | 7.80 | Gbar_D03_2023546 | T/C | 0.18 | 7.47 |
| Gbar_D03_1036297 | T/G | 0.14 | 7.12 | Gbar_D03_1345643 | T/G | 0.16 | 7.52 | Gbar_D03_2025058 | T/C | 0.18 | 6.67 |
| Gbar_D03_1042425 | A/T | 0.13 | 6.33 | Gbar_D03_1345907 | T/C | 0.16 | 8.20 | Gbar_D03_2030758 | T/C | 0.18 | 6.75 |
| Gbar_D03_1044462 | G/A | 0.14 | 6.12 | Gbar_D03_1347556 | A/C | 0.16 | 8.19 | Gbar_D03_2031835 | T/G | 0.18 | 7.53 |
| Gbar_D03_1058447 | A/G | 0.15 | 7.30 | Gbar_D03_1348970 | A/C | 0.15 | 7.69 | Gbar_D03_2034483 | T/G | 0.18 | 6.32 |
| Gbar_D03_1061196 | T/A | 0.15 | 6.84 | Gbar_D03_1349307 | A/G | 0.15 | 7.97 | Gbar_D03_2034515 | T/C | 0.18 | 6.50 |
| Gbar_D03_1061302 | A/G | 0.15 | 6.53 | Gbar_D03_1352685 | T/C | 0.14 | 6.86 | Gbar_D03_2034622 | T/C | 0.18 | 6.98 |
| Gbar_D03_1062012 | G/A | 0.15 | 7.81 | Gbar_D03_1354369 | T/G | 0.16 | 7.89 | Gbar_D03_2034913 | T/C | 0.17 | 6.35 |
| Gbar_D03_1063181 | T/C | 0.15 | 7.59 | Gbar_D03_1354636 | T/C | 0.13 | 6.72 | Gbar_D03_2037183 | A/G | 0.17 | 6.12 |
| Gbar_D03_1065012 | A/G | 0.14 | 6.58 | Gbar_D03_1354803 | T/C | 0.13 | 6.64 | Gbar_D03_2038069 | A/G | 0.17 | 6.04 |
| Gbar_D03_1067829 | A/G | 0.15 | 7.77 | Gbar_D03_1355184 | T/C | 0.14 | 6.36 | Gbar_D03_2040140 | T/C | 0.18 | 6.38 |
| Gbar_D03_1068053 | A/G | 0.15 | 8.63 | Gbar_D03_1357765 | A/T | 0.13 | 6.07 | Gbar_D03_2040228 | A/G | 0.18 | 6.32 |
| Gbar_D03_1068871 | A/G | 0.14 | 6.98 | Gbar_D03_1629970 | C/T | 0.18 | 10.52 | Gbar_D03_2041970 | A/G | 0.18 | 6.65 |
| Gbar_D03_1075321 | T/C | 0.14 | 6.86 | Gbar_D03_1664579 | T/C | 0.18 | 9.27 | Gbar_D03_2042357 | T/C | 0.18 | 7.14 |
| Gbar_D03_1075812 | A/G | 0.15 | 8.59 | Gbar_D03_1672718 | G/A | 0.19 | 9.23 | Gbar_D03_2043305 | G/A | 0.18 | 7.04 |
| Gbar_D03_1076207 | A/C | 0.15 | 6.66 | Gbar_D03_1676638 | A/C | 0.19 | 9.42 | Gbar_D03_2045627 | G/C | 0.18 | 6.16 |
| Gbar_D03_1076579 | T/C | 0.15 | 7.45 | Gbar_D03_1703450 | T/A | 0.17 | 8.35 | Gbar_D03_2046359 | A/G | 0.18 | 6.44 |
| Gbar_D03_1076588 | G/A | 0.15 | 7.81 | Gbar_D03_1822281 | G/A | 0.18 | 6.39 | Gbar_D03_2046523 | T/C | 0.18 | 6.29 |
| Gbar_D03_1076691 | A/G | 0.15 | 7.30 | Gbar_D03_1824274 | C/T | 0.18 | 6.63 | Gbar_D03_2047187 | A/C | 0.18 | 6.74 |
| Gbar_D03_1084319 | T/C | 0.15 | 7.14 | Gbar_D03_1824899 | T/C | 0.18 | 7.88 | Gbar_D03_2047669 | T/C | 0.18 | 7.74 |
| Gbar_D03_1085843 | T/G | 0.15 | 6.51 | Gbar_D03_1826623 | C/T | 0.18 | 6.95 | Gbar_D03_2047804 | T/C | 0.17 | 6.81 |
| Gbar_D03_1086685 | C/G | 0.15 | 7.92 | Gbar_D03_1828505 | T/A | 0.18 | 6.44 | Gbar_D03_2048430 | A/G | 0.18 | 6.09 |
| Gbar_D03_1087424 | A/C | 0.15 | 7.62 | Gbar_D03_1843065 | T/G | 0.17 | 6.75 | Gbar_D03_2049119 | A/C | 0.18 | 7.39 |
| Gbar_D03_1092095 | G/C | 0.16 | 8.86 | Gbar_D03_1846306 | T/G | 0.18 | 6.64 | Gbar_D03_2049740 | A/G | 0.18 | 6.41 |
| Gbar_D03_1097512 | G/A | 0.14 | 6.18 | Gbar_D03_1847461 | A/G | 0.17 | 6.43 | Gbar_D03_2050294 | G/T | 0.18 | 7.37 |
| Gbar_D03_1099556 | C/T | 0.18 | 7.95 | Gbar_D03_1849825 | T/C | 0.17 | 6.67 | Gbar_D03_2051583 | A/G | 0.18 | 6.01 |
| Gbar_D03_1100160 | A/C | 0.16 | 7.72 | Gbar_D03_1850013 | T/C | 0.17 | 6.16 | Gbar_D03_2051748 | T/C | 0.18 | 6.72 |
| Gbar_D03_1101128 | A/G | 0.16 | 7.40 | Gbar_D03_1853808 | T/C | 0.09 | 6.08 | Gbar_D03_2052908 | A/G | 0.18 | 6.70 |
| Gbar_D03_1101164 | G/A | 0.16 | 7.98 | Gbar_D03_1855104 | A/G | 0.17 | 6.52 | Gbar_D03_2054298 | T/C | 0.18 | 6.84 |
| Gbar_D03_1102735 | A/G | 0.15 | 6.84 | Gbar_D03_1857894 | A/G | 0.18 | 6.17 | Gbar_D03_2055339 | C/T | 0.18 | 7.20 |
| Gbar_D03_1103325 | T/C | 0.16 | 7.21 | Gbar_D03_1858765 | G/A | 0.18 | 6.80 | Gbar_D03_2057422 | G/A | 0.18 | 7.24 |
| Gbar_D03_1105012 | G/T | 0.16 | 8.40 | Gbar_D03_1860357 | T/C | 0.18 | 7.28 | Gbar_D03_2057743 | C/T | 0.09 | 6.12 |
| Gbar_D03_1107259 | A/C | 0.16 | 8.37 | Gbar_D03_1860429 | G/A | 0.08 | 6.01 | Gbar_D03_2060260 | C/A | 0.18 | 8.48 |
| Gbar_D03_1107653 | T/C | 0.16 | 7.23 | Gbar_D03_1872855 | G/C | 0.18 | 7.17 | Gbar_D03_2070493 | T/C | 0.18 | 6.43 |
| Gbar_D03_1107899 | A/G | 0.15 | 6.73 | Gbar_D03_1878030 | T/C | 0.08 | 6.41 | Gbar_D03_2070820 | A/T | 0.19 | 6.68 |
| Gbar_D03_1107934 | A/G | 0.16 | 7.27 | Gbar_D03_1879874 | T/C | 0.09 | 6.57 | Gbar_D03_2072242 | A/C | 0.18 | 7.04 |
| Gbar_D03_1108433 | A/G | 0.16 | 7.38 | Gbar_D03_1881025 | G/A | 0.09 | 6.34 | Gbar_D03_2072293 | T/C | 0.18 | 7.39 |
| Gbar_D03_1110567 | A/G | 0.21 | 11.57 | Gbar_D03_1882031 | T/C | 0.18 | 6.43 | Gbar_D03_2073552 | G/A | 0.18 | 6.85 |
| Gbar_D03_1111610 | T/C | 0.16 | 7.94 | Gbar_D03_1886621 | A/G | 0.18 | 6.52 | Gbar_D03_2085912 | T/C | 0.18 | 7.01 |
| Gbar_D03_1112502 | C/G | 0.15 | 6.16 | Gbar_D03_1890205 | T/G | 0.18 | 6.64 | Gbar_D03_2096694 | T/A | 0.18 | 6.05 |
| Gbar_D03_1115166 | A/T | 0.16 | 7.71 | Gbar_D03_1892354 | T/G | 0.18 | 7.28 | Gbar_D03_2214857 | A/G | 0.16 | 8.32 |
| Gbar_D03_1115729 | T/G | 0.16 | 6.22 | Gbar_D03_1896485 | T/C | 0.17 | 6.35 | Gbar_D03_2215669 | A/T | 0.17 | 8.77 |
| Gbar_D03_1116342 | G/A | 0.16 | 8.00 | Gbar_D03_1901449 | G/A | 0.18 | 6.02 | Gbar_D03_2222499 | T/C | 0.16 | 8.13 |
| Gbar_D03_1116402 | A/G | 0.16 | 7.74 | Gbar_D03_1903013 | T/C | 0.18 | 6.72 | Gbar_D03_2222921 | A/G | 0.17 | 8.09 |
| Gbar_D03_1116623 | A/G | 0.15 | 6.53 | Gbar_D03_1904562 | C/T | 0.17 | 6.27 | Gbar_D03_2224222 | A/G | 0.20 | 8.72 |
| Gbar_D03_1117378 | A/G | 0.16 | 8.41 | Gbar_D03_1904856 | A/G | 0.18 | 6.77 | Gbar_D03_2224946 | A/G | 0.16 | 8.16 |
| Gbar_D03_1117759 | A/G | 0.16 | 7.62 | Gbar_D03_1912152 | T/C | 0.17 | 6.41 | Gbar_D03_2224965 | A/G | 0.17 | 8.37 |
| Gbar_D03_1118107 | A/G | 0.16 | 6.62 | Gbar_D03_1921525 | T/C | 0.17 | 6.25 | Gbar_D03_2228451 | A/G | 0.14 | 8.12 |
| Gbar_D03_1118571 | T/C | 0.16 | 7.52 | Gbar_D03_1922237 | A/T | 0.08 | 6.28 | Gbar_D03_2249026 | C/T | 0.09 | 6.09 |
| Gbar_D03_1118866 | A/G | 0.16 | 7.91 | Gbar_D03_1922408 | T/C | 0.09 | 6.16 | Gbar_D03_2249706 | T/C | 0.15 | 8.20 |
| Gbar_D03_1119560 | C/A | 0.15 | 6.01 | Gbar_D03_1922876 | T/C | 0.18 | 7.08 | Gbar_D03_2255845 | T/G | 0.14 | 7.80 |
| Gbar_D03_1119718 | G/T | 0.17 | 7.47 | Gbar_D03_1923098 | A/G | 0.08 | 6.17 | Gbar_D03_2255926 | T/C | 0.14 | 6.94 |
| Gbar_D03_1120632 | G/A | 0.17 | 7.02 | Gbar_D03_1923348 | C/A | 0.18 | 6.74 | Gbar_D03_2256558 | T/A | 0.14 | 7.53 |
| Gbar_D03_1121167 | G/A | 0.16 | 7.28 | Gbar_D03_1924417 | A/G | 0.17 | 6.46 | Gbar_D03_2256560 | T/A | 0.14 | 8.15 |
| Gbar_D03_1121203 | T/C | 0.15 | 7.07 | Gbar_D03_1928629 | T/C | 0.18 | 7.17 | Gbar_D03_2289243 | A/G | 0.14 | 6.67 |
| Gbar_D03_1121577 | G/A | 0.16 | 7.71 | Gbar_D03_1930207 | A/G | 0.18 | 6.45 | Gbar_D03_2368579 | T/A | 0.17 | 6.53 |
| Gbar_D03_1125472 | G/C | 0.16 | 7.24 | Gbar_D03_1931209 | T/A | 0.18 | 6.54 | Gbar_D03_603337 | T/G | 0.08 | 6.03 |
| Gbar_D03_1126238 | T/C | 0.15 | 7.25 | Gbar_D03_1940049 | T/G | 0.18 | 6.03 | Gbar_D03_630448 | A/C | 0.08 | 6.07 |
| Gbar_D03_1126669 | T/C | 0.16 | 6.79 | Gbar_D03_1941440 | A/T | 0.09 | 6.33 | Gbar_D03_641827 | T/C | 0.08 | 6.06 |
| Gbar_D03_1142935 | T/C | 0.16 | 6.63 | Gbar_D03_1956911 | A/T | 0.18 | 6.32 | Gbar_D03_644468 | A/T | 0.08 | 6.20 |
| Gbar_D03_1142946 | C/G | 0.16 | 7.00 | Gbar_D03_1957237 | T/G | 0.09 | 6.32 | Gbar_D03_653541 | G/T | 0.09 | 6.03 |
| Gbar_D03_1159232 | A/G | 0.16 | 8.29 | Gbar_D03_1957546 | A/G | 0.18 | 6.95 | Gbar_D03_653900 | A/T | 0.08 | 6.00 |
| Gbar_D03_1159584 | A/G | 0.17 | 7.52 | Gbar_D03_1963579 | G/A | 0.18 | 6.84 | Gbar_D03_717823 | C/A | 0.08 | 6.12 |
| Gbar_D03_1279385 | G/A | 0.10 | 6.16 | Gbar_D03_1970210 | A/T | 0.18 | 7.12 | Gbar_D03_718281 | A/G | 0.08 | 6.17 |
| Gbar_D03_1318456 | A/T | 0.16 | 7.56 | Gbar_D03_1980162 | A/T | 0.12 | 7.34 | Gbar_D03_718293 | T/C | 0.08 | 6.09 |
| Gbar_D03_1324656 | A/C | 0.13 | 6.07 | Gbar_D03_1980968 | A/G | 0.18 | 6.68 | Gbar_D03_723816 | G/A | 0.08 | 6.07 |
| Gbar_D03_1328529 | C/T | 0.14 | 7.29 | Gbar_D03_1984911 | T/C | 0.18 | 7.31 | Gbar_D03_746977 | C/G | 0.08 | 6.11 |
| Gbar_D03_1329020 | A/G | 0.13 | 7.62 | Gbar_D03_1989223 | C/T | 0.09 | 6.04 | Gbar_D03_754158 | C/T | 0.08 | 6.16 |
| Gbar_D03_1329117 | C/T | 0.13 | 6.36 | Gbar_D03_1989485 | T/C | 0.18 | 7.46 | Gbar_D03_754191 | A/T | 0.08 | 6.20 |
| Gbar_D03_1330258 | A/G | 0.13 | 6.07 | Gbar_D03_1989700 | A/C | 0.17 | 6.90 | Gbar_D03_772090 | G/A | 0.08 | 6.01 |
| Gbar_D03_1330863 | A/G | 0.13 | 6.83 | Gbar_D03_1990362 | A/G | 0.18 | 7.24 | Gbar_D03_772091 | G/A | 0.08 | 6.25 |
| Gbar_D03_1331499 | T/C | 0.14 | 6.97 | Gbar_D03_2007434 | T/A | 0.18 | 6.71 | Gbar_D03_777135 | T/G | 0.08 | 6.05 |
| Gbar_D03_1331651 | A/G | 0.13 | 6.91 | Gbar_D03_2008527 | A/G | 0.18 | 6.93 | Gbar_D03_809907 | T/A | 0.08 | 6.30 |
| Gbar_D03_1331884 | T/A | 0.13 | 6.42 | Gbar_D03_2014620 | T/C | 0.18 | 6.99 | Gbar_D03_809936 | T/C | 0.08 | 6.15 |
| Gbar_D03_1332091 | T/G | 0.12 | 7.29 | Gbar_D03_2014708 | A/G | 0.17 | 6.36 | Gbar_D03_810613 | A/G | 0.08 | 6.02 |
| Gbar_D03_1333123 | A/G | 0.13 | 6.39 | Gbar_D03_2015383 | G/C | 0.18 | 6.54 | Gbar_D03_822027 | A/T | 0.08 | 6.05 |
| Gbar_D03_1335353 | G/T | 0.16 | 8.79 | Gbar_D03_2015612 | T/C | 0.18 | 6.89 | Gbar_D03_826631 | G/A | 0.08 | 6.09 |
| Gbar_D03_1336153 | A/G | 0.15 | 7.42 | Gbar_D03_2015714 | C/T | 0.18 | 6.12 | Gbar_D03_848643 | A/C | 0.08 | 6.12 |
| Gbar_D03_1336922 | A/G | 0.16 | 7.89 | Gbar_D03_2019628 | C/T | 0.09 | 6.02 | Gbar_D03_993066 | T/C | 0.13 | 6.44 |
| Gbar_D03_1337989 | A/G | 0.15 | 8.02 | Gbar_D03_2019774 | A/G | 0.17 | 6.54 | Gbar_D03_995976 | T/C | 0.13 | 6.06 |
| Gbar_D03_1340760 | A/G | 0.14 | 7.48 | Gbar_D03_2019798 | T/C | 0.18 | 6.58 | Gbar_D06_44882448 | C/A | 0.17 | 6.03 |

**Table S3.** Summary of the total associated SNPs identified through GWAS for *FW* resistance in Sea Island cotton.

| SNP | Ref/Alt | MAF | -log (P-value) in different environments | | | | | | SNP | Ref/Alt | MAF | -log (P-value) in different environments | | | | | |
| --- | --- | --- | --- | --- | --- | --- | --- | --- | --- | --- | --- | --- | --- | --- | --- | --- | --- |
|  |  |  | 2015 | 2016 | 2018 | 2019 | Mean | BLUP |  |  |  | 2015 | 2016 | 2018 | 2019 | Mean | BLUP |
| Gbar_A02_98595082 | T/A | 0.44 | - | - | - | 6.06 | - | 6.16 | Gbar_D03_1159584 | A/G | 0.17 | - | - | - | 7.60 | 7.28 | 7.52 |
| Gbar_A07_82872256 | A/T | 0.13 | - | - | - | - | 6.62 | 6.11 | Gbar_D03_1173668 | C/T | 0.08 | - | 6.05 | - | - | - | - |
| Gbar_A11_89241215 | T/G | 0.15 | - | - | - | 6.26 | 6.94 | 6.54 | Gbar_D03_1174973 | G/A | 0.08 | 6.15 | 6.13 | - | - | - | - |
| Gbar_A11_89242281 | G/A | 0.13 | - | - | - | - | 6.07 | - | Gbar_D03_1180020 | G/A | 0.08 | 6.06 | 6.31 | - | - | - | - |
| Gbar_A11_89242339 | A/T | 0.13 | - | - | - | - | 6.05 | - | Gbar_D03_1187747 | T/C | 0.08 | 6.29 | 6.95 | - | - | - | - |
| Gbar_A11_89242355 | T/A | 0.12 | - | - | - | - | 6.35 | - | Gbar_D03_1192013 | G/C | 0.08 | 6.49 | 6.41 | - | - | - | - |
| Gbar_A11_89242365 | G/A | 0.12 | - | - | - | 6.41 | 6.97 | 6.53 | Gbar_D03_1224465 | A/C | 0.08 | 6.06 | - | - | - | - | - |
| Gbar_A11_89242374 | T/C | 0.13 | - | - | - | - | 6.40 | - | Gbar_D03_1226116 | G/A | 0.08 | - | 6.16 | - | - | - | - |
| Gbar_A11_89242384 | T/C | 0.11 | - | - | - | 6.04 | 7.14 | 6.53 | Gbar_D03_1266739 | G/A | 0.08 | - | 6.15 | - | - | - | - |
| Gbar_A11_89242399 | C/T | 0.11 | - | - | - | - | 6.25 | - | Gbar_D03_1269091 | A/T | 0.08 | 6.71 | 7.05 | - | - | - | - |
| Gbar_A13_40120505 | A/C | 0.12 | - | - | - | 6.15 | 6.33 | 6.23 | Gbar_D03_1274626 | C/G | 0.08 | - | 6.23 | - | - | - | - |
| Gbar_D03_1001198 | G/A | 0.08 | 6.79 | 6.94 | - | - | - | - | Gbar_D03_1277678 | A/T | 0.08 | 6.60 | 6.87 | - | - | - | - |
| Gbar_D03_1002381 | T/C | 0.13 | - | - | - | 6.36 | 6.39 | 6.58 | Gbar_D03_1279352 | C/T | 0.08 | 6.77 | 6.75 | - | - | - | - |
| Gbar_D03_1009181 | G/A | 0.13 | - | - | - | 6.75 | 6.84 | 7.04 | Gbar_D03_1279385 | G/A | 0.10 | 6.35 | 6.32 | - | - | - | 6.16 |
| Gbar_D03_1021128 | T/C | 0.08 | 6.52 | 6.50 | - | - | - | - | Gbar_D03_1281305 | C/T | 0.07 | 6.37 | 6.25 | - | - | - | - |
| Gbar_D03_1022935 | T/G | 0.14 | - | - | - | 6.17 | 6.01 | 6.19 | Gbar_D03_1318456 | A/T | 0.16 | - | - | 6.01 | 7.89 | 7.28 | 7.56 |
| Gbar_D03_1024618 | A/G | 0.15 | - | - | - | 6.63 | 6.59 | 6.76 | Gbar_D03_1319515 | C/T | 0.08 | 6.79 | 7.33 | - | - | - | - |
| Gbar_D03_1024724 | T/C | 0.15 | - | - | - | 6.35 | 6.22 | 6.42 | Gbar_D03_1321491 | C/T | 0.08 | - | 6.46 | - | - | - | - |
| Gbar_D03_1025046 | C/T | 0.14 | - | - | - | 6.46 | 6.31 | 6.50 | Gbar_D03_1323180 | C/A | 0.08 | - | 6.75 | - | - | - | - |
| Gbar_D03_1029916 | T/C | 0.14 | - | - | - | 6.84 | 6.63 | 6.84 | Gbar_D03_1324656 | A/C | 0.13 | - | - | - | - | - | 6.07 |
| Gbar_D03_1033138 | A/G | 0.09 | 6.46 | 6.93 | - | - | - | - | Gbar_D03_1328529 | C/T | 0.14 | - | - | - | 7.09 | 7.05 | 7.29 |
| Gbar_D03_1036297 | T/G | 0.14 | 6.48 | - | - | 6.97 | 6.93 | 7.12 | Gbar_D03_1329020 | A/G | 0.13 | 6.21 | 6.18 | - | 7.32 | 7.39 | 7.62 |
| Gbar_D03_1041984 | G/A | 0.08 | - | 6.45 | - | - | - | - | Gbar_D03_1329117 | C/T | 0.13 | - | - | - | 6.05 | 6.20 | 6.36 |
| Gbar_D03_1042425 | A/T | 0.13 | - | - | - | 6.07 | 6.16 | 6.33 | Gbar_D03_1330258 | A/G | 0.13 | - | - | - | - | - | 6.07 |
| Gbar_D03_1044462 | G/A | 0.14 | - | - | - | 6.07 | - | 6.12 | Gbar_D03_1330863 | A/G | 0.13 | 6.33 | 6.40 | - | 6.65 | 6.57 | 6.83 |
| Gbar_D03_1050696 | G/T | 0.38 | 7.66 | - | 9.35 | - | - | - | Gbar_D03_1331499 | T/C | 0.14 | - | - | - | 6.73 | 6.75 | 6.97 |
| Gbar_D03_1050724 | G/T | 0.39 | 7.56 | - | 8.75 | - | - | - | Gbar_D03_1331651 | A/G | 0.13 | - | - | - | 6.70 | 6.71 | 6.91 |
| Gbar_D03_1052369 | A/C | 0.37 | 6.10 | - | 6.81 | - | - | - | Gbar_D03_1331884 | T/A | 0.13 | - | - | - | 6.26 | 6.20 | 6.42 |
| Gbar_D03_1053536 | A/T | 0.38 | 6.66 | - | 9.70 | - | - | - | Gbar_D03_1332091 | T/G | 0.12 | 6.02 | 7.07 | - | 6.99 | 7.06 | 7.29 |
| Gbar_D03_1057322 | C/T | 0.39 | 7.71 | - | 9.88 | - | - | - | Gbar_D03_1333123 | A/G | 0.13 | - | - | - | 6.13 | 6.22 | 6.39 |
| Gbar_D03_1057381 | G/A | 0.39 | 7.88 | - | 10.17 | - | - | - | Gbar_D03_1335353 | G/T | 0.16 | - | - | 6.87 | 9.20 | 8.44 | 8.79 |
| Gbar_D03_1057671 | G/T | 0.40 | - | - | 6.94 | - | - | - | Gbar_D03_1336153 | A/G | 0.15 | - | - | - | 8.05 | 7.12 | 7.42 |
| Gbar_D03_1057751 | G/A | 0.40 | - | - | 6.59 | - | - | - | Gbar_D03_1336922 | A/G | 0.16 | 6.20 | - | 6.50 | 8.26 | 7.56 | 7.89 |
| Gbar_D03_1057802 | G/C | 0.38 | - | - | 6.77 | - | - | - | Gbar_D03_1337989 | A/G | 0.15 | - | - | 6.48 | 8.75 | 7.67 | 8.02 |
| Gbar_D03_1057819 | T/C | 0.39 | - | - | 7.24 | - | - | - | Gbar_D03_1340760 | A/G | 0.14 | - | - | 6.43 | 7.58 | 7.19 | 7.48 |
| Gbar_D03_1058447 | A/G | 0.15 | - | - | - | 7.06 | 7.12 | 7.30 | Gbar_D03_1341476 | G/T | 0.13 | - | - | - | 6.03 | - | - |
| Gbar_D03_1058771 | T/C | 0.41 | 6.13 | - | 7.54 | - | - | - | Gbar_D03_1342587 | G/A | 0.16 | - | - | - | 8.40 | 7.59 | 7.90 |
| Gbar_D03_1058777 | C/A | 0.41 | 6.19 | - | 7.32 | - | - | - | Gbar_D03_1343503 | G/A | 0.15 | - | - | - | 8.20 | 7.43 | 7.71 |
| Gbar_D03_1058910 | C/T | 0.40 | 6.73 | - | 8.88 | - | - | - | Gbar_D03_1343889 | A/G | 0.16 | - | - | 7.33 | 10.04 | 8.93 | 9.31 |
| Gbar_D03_1060248 | G/C | 0.41 | - | - | 6.30 | - | - | - | Gbar_D03_1344273 | T/C | 0.16 | - | - | - | 7.82 | 7.04 | 7.34 |
| Gbar_D03_1061196 | T/A | 0.15 | 6.37 | - | - | 6.86 | 6.64 | 6.84 | Gbar_D03_1344534 | T/C | 0.16 | - | - | 6.25 | 8.34 | 7.49 | 7.80 |
| Gbar_D03_1061302 | A/G | 0.15 | - | - | - | 6.29 | 6.35 | 6.53 | Gbar_D03_1345643 | T/G | 0.16 | - | - | 6.40 | 8.23 | 7.18 | 7.52 |
| Gbar_D03_1062012 | G/A | 0.15 | - | - | - | 7.85 | 7.56 | 7.81 | Gbar_D03_1345907 | T/C | 0.16 | 6.01 | - | 6.57 | 8.80 | 7.83 | 8.20 |
| Gbar_D03_1062430 | T/C | 0.10 | 6.36 | - | - | - | - | - | Gbar_D03_1347556 | A/C | 0.16 | - | - | 6.60 | 8.84 | 7.84 | 8.19 |
| Gbar_D03_1063181 | T/C | 0.15 | - | 6.09 | - | 7.51 | 7.35 | 7.59 | Gbar_D03_1348970 | A/C | 0.15 | 6.06 | - | 6.07 | 8.16 | 7.34 | 7.69 |
| Gbar_D03_1064857 | C/G | 0.09 | 6.53 | 7.04 | - | - | - | - | Gbar_D03_1349307 | A/G | 0.15 | - | - | 6.38 | 8.66 | 7.67 | 7.97 |
| Gbar_D03_1065012 | A/G | 0.14 | - | - | - | 6.53 | 6.42 | 6.58 | Gbar_D03_1352685 | T/C | 0.14 | - | - | - | 6.92 | 6.60 | 6.86 |
| Gbar_D03_1067829 | A/G | 0.15 | - | - | - | 7.94 | 7.52 | 7.77 | Gbar_D03_1354369 | T/G | 0.16 | - | - | - | 8.16 | 7.61 | 7.89 |
| Gbar_D03_1068053 | A/G | 0.15 | 6.22 | 6.45 | 6.82 | 8.86 | 8.30 | 8.63 | Gbar_D03_1354636 | T/C | 0.13 | - | - | - | 6.91 | 6.45 | 6.72 |
| Gbar_D03_1068871 | A/G | 0.14 | - | - | - | 7.24 | 6.68 | 6.98 | Gbar_D03_1354803 | T/C | 0.13 | - | - | - | 6.73 | 6.40 | 6.64 |
| Gbar_D03_1075321 | T/C | 0.14 | - | - | - | 6.53 | 6.70 | 6.86 | Gbar_D03_1355184 | T/C | 0.14 | - | - | - | 6.35 | 6.14 | 6.36 |
| Gbar_D03_1075812 | A/G | 0.15 | 6.01 | 6.34 | 6.49 | 8.75 | 8.29 | 8.59 | Gbar_D03_1357765 | A/T | 0.13 | - | - | - | 6.24 | - | 6.07 |
| Gbar_D03_1076207 | A/C | 0.15 | - | - | - | 7.00 | 6.41 | 6.66 | Gbar_D03_1359249 | A/C | 0.08 | 6.02 | - | - | - | - | - |
| Gbar_D03_1076579 | T/C | 0.15 | 6.72 | 6.36 | - | 7.37 | 7.19 | 7.45 | Gbar_D03_1359810 | A/G | 0.09 | 7.68 | 7.79 | - | - | - | - |
| Gbar_D03_1076588 | G/A | 0.15 | 6.70 | 6.54 | - | 7.68 | 7.54 | 7.81 | Gbar_D03_1400528 | A/G | 0.08 | 6.15 | - | - | - | - | - |
| Gbar_D03_1076691 | A/G | 0.15 | - | - | - | 7.50 | 7.05 | 7.30 | Gbar_D03_1456870 | G/A | 0.08 | 6.50 | 6.13 | - | - | - | - |
| Gbar_D03_1084319 | T/C | 0.15 | - | - | - | 7.37 | 6.90 | 7.14 | Gbar_D03_1460925 | G/C | 0.08 | 6.23 | 6.18 | - | - | - | - |
| Gbar_D03_1085843 | T/G | 0.15 | - | - | - | 6.72 | 6.25 | 6.51 | Gbar_D03_1471416 | G/A | 0.09 | 6.73 | 6.64 | - | - | - | - |
| Gbar_D03_1086685 | C/G | 0.15 | - | - | - | 8.07 | 7.66 | 7.92 | Gbar_D03_1471482 | C/A | 0.09 | 6.29 | 6.12 | - | - | - | - |
| Gbar_D03_1087424 | A/C | 0.15 | 6.29 | - | - | 7.81 | 7.37 | 7.62 | Gbar_D03_1501667 | T/C | 0.09 | 6.53 | 6.46 | - | - | - | - |
| Gbar_D03_1092095 | G/C | 0.16 | 6.30 | - | 6.27 | 9.02 | 8.57 | 8.86 | Gbar_D03_1501683 | C/T | 0.08 | 6.34 | 6.26 | - | - | - | - |
| Gbar_D03_1097512 | G/A | 0.14 | - | - | - | 6.46 | - | 6.18 | Gbar_D03_1505214 | T/G | 0.09 | 6.07 | - | - | - | - | - |
| Gbar_D03_1099556 | C/T | 0.18 | - | - | 6.03 | 8.51 | 7.72 | 7.95 | Gbar_D03_1505218 | T/C | 0.09 | 6.06 | - | - | - | - | - |
| Gbar_D03_1100160 | A/C | 0.16 | - | - | 6.10 | 7.80 | 7.45 | 7.72 | Gbar_D03_1509599 | T/C | 0.08 | 6.10 | 6.27 | - | - | - | - |
| Gbar_D03_1101128 | A/G | 0.16 | - | 6.13 | - | 7.27 | 7.18 | 7.40 | Gbar_D03_1513731 | A/T | 0.08 | 6.32 | 6.34 | - | - | - | - |
| Gbar_D03_1101164 | G/A | 0.16 | - | 6.05 | 6.04 | 7.94 | 7.74 | 7.98 | Gbar_D03_1513733 | C/A | 0.09 | 6.40 | 6.40 | - | - | - | - |
| Gbar_D03_1102735 | A/G | 0.15 | - | - | - | 7.04 | 6.64 | 6.84 | Gbar_D03_1513734 | C/A | 0.09 | 6.11 | 6.25 | - | - | - | - |
| Gbar_D03_1103325 | T/C | 0.16 | - | - | - | 7.41 | 6.96 | 7.21 | Gbar_D03_1531348 | C/T | 0.09 | - | 6.27 | - | - | - | - |
| Gbar_D03_1105006 | A/G | 0.08 | 6.19 | 6.78 | - | - | - | - | Gbar_D03_1534765 | C/T | 0.08 | - | 6.11 | - | - | - | - |
| Gbar_D03_1105012 | G/T | 0.16 | 6.55 | 7.70 | 6.64 | 8.16 | 8.14 | 8.40 | Gbar_D03_1537617 | T/G | 0.08 | 6.24 | 6.12 | - | - | - | - |
| Gbar_D03_1105045 | C/T | 0.08 | 6.02 | 6.44 | - | - | - | - | Gbar_D03_1541480 | A/G | 0.08 | 6.24 | 6.25 | - | - | - | - |
| Gbar_D03_1107259 | A/C | 0.16 | 6.20 | 6.78 | 6.79 | 8.42 | 8.07 | 8.37 | Gbar_D03_1541489 | T/A | 0.09 | 6.33 | 6.41 | - | - | - | - |
| Gbar_D03_1107653 | T/C | 0.16 | - | 6.08 | - | 7.27 | 6.98 | 7.23 | Gbar_D03_1601122 | C/T | 0.08 | 6.32 | 6.54 | - | - | - | - |
| Gbar_D03_1107899 | A/G | 0.15 | - | - | - | 6.90 | 6.47 | 6.73 | Gbar_D03_1601160 | C/T | 0.08 | 6.20 | 6.33 | - | - | - | - |
| Gbar_D03_1107934 | A/G | 0.16 | - | - | - | 7.32 | 7.04 | 7.27 | Gbar_D03_1620201 | A/C | 0.08 | 6.38 | 6.77 | - | - | - | - |
| Gbar_D03_1108433 | A/G | 0.16 | - | 6.39 | - | 7.36 | 7.13 | 7.38 | Gbar_D03_1629970 | C/T | 0.18 | - | 6.94 | 8.64 | 11.52 | 10.10 | 10.52 |
| Gbar_D03_1110567 | A/G | 0.21 | 6.99 | 8.11 | 10.00 | 13.12 | 11.01 | 11.57 | Gbar_D03_1638142 | A/C | 0.08 | - | 6.42 | - | - | - | - |
| Gbar_D03_1111610 | T/C | 0.16 | - | - | 6.44 | 8.00 | 7.69 | 7.94 | Gbar_D03_1663528 | G/A | 0.08 | - | 6.17 | - | - | - | - |
| Gbar_D03_1112502 | C/G | 0.15 | - | - | - | 6.04 | 6.01 | 6.16 | Gbar_D03_1664579 | T/C | 0.18 | - | - | 8.13 | 10.44 | 8.89 | 9.27 |
| Gbar_D03_1115166 | A/T | 0.16 | - | - | 6.35 | 7.93 | 7.44 | 7.71 | Gbar_D03_1672432 | A/T | 0.08 | 6.18 | 6.52 | - | - | - | - |
| Gbar_D03_1115729 | T/G | 0.16 | - | - | - | 6.41 | - | 6.22 | Gbar_D03_1672718 | G/A | 0.19 | - | 6.09 | 7.88 | 10.24 | 8.86 | 9.23 |
| Gbar_D03_1116342 | G/A | 0.16 | 6.53 | 6.75 | 6.63 | 8.04 | 7.70 | 8.00 | Gbar_D03_1676638 | A/C | 0.19 | - | - | 7.82 | 10.45 | 9.06 | 9.42 |
| Gbar_D03_1116402 | A/G | 0.16 | 6.17 | 6.40 | 6.23 | 7.81 | 7.46 | 7.74 | Gbar_D03_1686833 | A/G | 0.08 | 6.15 | 6.24 | - | - | - | - |
| Gbar_D03_1116623 | A/G | 0.15 | - | - | - | 6.51 | 6.33 | 6.53 | Gbar_D03_1699544 | G/A | 0.08 | 6.14 | 6.13 | - | - | - | - |
| Gbar_D03_1117378 | A/G | 0.16 | - | 6.34 | 6.57 | 8.59 | 8.11 | 8.41 | Gbar_D03_1700917 | T/G | 0.08 | 6.47 | 6.31 | - | - | - | - |
| Gbar_D03_1117759 | A/G | 0.16 | 6.31 | 6.65 | 6.26 | 7.49 | 7.38 | 7.62 | Gbar_D03_1703450 | T/A | 0.17 | - | 6.26 | 7.66 | 8.72 | 8.03 | 8.35 |
| Gbar_D03_1118107 | A/G | 0.16 | - | - | - | 6.62 | 6.43 | 6.62 | Gbar_D03_1705967 | A/T | 0.08 | 6.52 | 6.33 | - | - | - | - |
| Gbar_D03_1118571 | T/C | 0.16 | - | 6.32 | - | 7.56 | 7.25 | 7.52 | Gbar_D03_1714170 | A/G | 0.09 | 6.53 | 6.07 | - | - | - | - |
| Gbar_D03_1118866 | A/G | 0.16 | - | - | 6.17 | 7.97 | 7.66 | 7.91 | Gbar_D03_1763630 | G/T | 0.08 | 6.22 | - | - | - | - | - |
| Gbar_D03_1119560 | C/A | 0.15 | - | - | - | 6.09 | - | 6.01 | Gbar_D03_1805146 | G/A | 0.09 | 6.11 | - | - | - | - | - |
| Gbar_D03_1119592 | A/G | 0.16 | - | - | - | 6.16 | - | - | Gbar_D03_1822281 | G/A | 0.18 | - | - | 6.59 | 6.96 | 6.08 | 6.39 |
| Gbar_D03_1119718 | G/T | 0.17 | - | - | - | 7.49 | 7.24 | 7.47 | Gbar_D03_1824274 | C/T | 0.18 | - | - | 6.74 | 7.24 | 6.33 | 6.63 |
| Gbar_D03_1120632 | G/A | 0.17 | - | - | - | 7.09 | 6.79 | 7.02 | Gbar_D03_1824622 | T/C | 0.18 | - | - | 6.23 | - | - | - |
| Gbar_D03_1121167 | G/A | 0.16 | - | 6.33 | - | 7.21 | 7.08 | 7.28 | Gbar_D03_1824899 | T/C | 0.18 | 6.51 | 6.65 | 8.10 | 8.29 | 7.51 | 7.88 |
| Gbar_D03_1121203 | T/C | 0.15 | - | 6.02 | - | 6.99 | 6.88 | 7.07 | Gbar_D03_1826623 | C/T | 0.18 | - | - | 7.28 | 7.51 | 6.60 | 6.95 |
| Gbar_D03_1121577 | G/A | 0.16 | 6.07 | 6.56 | 6.27 | 7.82 | 7.41 | 7.71 | Gbar_D03_1828505 | T/A | 0.18 | - | - | 6.85 | 6.86 | 6.15 | 6.44 |
| Gbar_D03_1125472 | G/C | 0.16 | - | - | - | 7.15 | 7.05 | 7.24 | Gbar_D03_1833455 | T/A | 0.09 | 6.38 | - | - | - | - | - |
| Gbar_D03_1126238 | T/C | 0.15 | 6.54 | 6.66 | 6.01 | 7.11 | 7.01 | 7.25 | Gbar_D03_1838463 | G/A | 0.09 | 6.05 | - | - | - | - | - |
| Gbar_D03_1126669 | T/C | 0.16 | - | - | - | 6.95 | 6.57 | 6.79 | Gbar_D03_1839061 | G/A | 0.09 | 6.22 | 6.35 | - | - | - | - |
| Gbar_D03_1131117 | A/G | 0.08 | 6.10 | - | - | - | - | - | Gbar_D03_1843065 | T/G | 0.17 | - | 6.50 | 7.56 | 7.30 | 6.37 | 6.75 |
| Gbar_D03_1131867 | G/A | 0.08 | - | 6.50 | - | - | - | - | Gbar_D03_1846306 | T/G | 0.18 | 6.07 | - | 6.66 | 7.03 | 6.32 | 6.64 |
| Gbar_D03_1133323 | G/A | 0.08 | 6.20 | 6.08 | - | - | - | - | Gbar_D03_1847461 | A/G | 0.17 | - | - | - | 6.94 | 6.18 | 6.43 |
| Gbar_D03_1140499 | C/A | 0.08 | 6.52 | 6.52 | - | - | - | - | Gbar_D03_1849825 | T/C | 0.17 | - | - | 6.32 | 6.96 | 6.40 | 6.67 |
| Gbar_D03_1140510 | T/G | 0.08 | - | 6.16 | - | - | - | - | Gbar_D03_1850013 | T/C | 0.17 | - | - | - | 6.48 | - | 6.16 |
| Gbar_D03_1141035 | C/T | 0.08 | 6.93 | 6.86 | - | - | - | - | Gbar_D03_1853808 | T/C | 0.09 | 7.28 | 7.24 | - | - | - | 6.08 |
| Gbar_D03_1141451 | G/T | 0.08 | 6.56 | 7.19 | - | - | - | - | Gbar_D03_1854675 | A/G | 0.09 | 6.13 | - | - | - | - | - |
| Gbar_D03_1142341 | A/G | 0.08 | 6.06 | 6.36 | - | - | - | - | Gbar_D03_1855104 | A/G | 0.17 | - | - | 6.52 | 6.90 | 6.23 | 6.52 |
| Gbar_D03_1142935 | T/C | 0.16 | - | - | - | 7.01 | 6.37 | 6.63 | Gbar_D03_1855408 | T/C | 0.09 | 6.13 | - | - | - | - | - |
| Gbar_D03_1142946 | C/G | 0.16 | - | - | - | 7.21 | 6.76 | 7.00 | Gbar_D03_1855413 | C/T | 0.09 | 6.71 | 6.13 | - | - | - | - |
| Gbar_D03_1145550 | T/C | 0.08 | - | 6.13 | - | - | - | - | Gbar_D03_1855570 | T/C | 0.09 | 6.55 | - | - | - | - | - |
| Gbar_D03_1159232 | A/G | 0.16 | 6.23 | 6.66 | 7.06 | 8.52 | 7.97 | 8.29 | Gbar_D03_1856190 | G/A | 0.17 | - | - | 6.21 | - | - | - |

**Table S3.** Summary of the total associated SNPs identified through GWAS for *FW* resistance in Sea Island cotton. (Continued)

| SNP | Ref/Alt | MAF | -log (P-value) in different environments | | | | | | SNP | Ref/Alt | MAF | -log (P-value) in different environments | | | | | |
| --- | --- | --- | --- | --- | --- | --- | --- | --- | --- | --- | --- | --- | --- | --- | --- | --- | --- |
|  |  |  | 2015 | 2016 | 2018 | 2019 | Mean | BLUP |  |  |  | 2015 | 2016 | 2018 | 2019 | Mean | BLUP |
| Gbar_D03_1857894 | A/G | 0.18 | - | - | 7.33 | 6.67 | - | 6.17 | Gbar_D03_2035829 | T/G | 0.09 | 6.50 | 6.04 | - | - | - | - |
| Gbar_D03_1858765 | G/A | 0.18 | - | - | 7.14 | 7.41 | 6.46 | 6.80 | Gbar_D03_2035835 | T/C | 0.09 | 6.72 | 6.42 | - | - | - | - |
| Gbar_D03_1860152 | G/A | 0.09 | 6.19 | - | - | - | - | - | Gbar_D03_2036939 | A/G | 0.17 | - | - | 6.10 | 6.45 | - | - |
| Gbar_D03_1860357 | T/C | 0.18 | - | - | 7.52 | 7.95 | 6.92 | 7.28 | Gbar_D03_2037183 | A/G | 0.17 | - | - | 6.90 | 6.71 | - | 6.12 |
| Gbar_D03_1860402 | A/G | 0.08 | 6.23 | - | - | - | - | - | Gbar_D03_2038069 | A/G | 0.17 | - | - | 6.38 | 6.63 | - | 6.04 |
| Gbar_D03_1860417 | T/C | 0.08 | 6.33 | - | - | - | - | - | Gbar_D03_2039439 | A/G | 0.18 | - | - | - | 6.70 | - | - |
| Gbar_D03_1860429 | G/A | 0.08 | 6.88 | 6.94 | - | - | - | 6.01 | Gbar_D03_2040140 | T/C | 0.18 | - | - | 6.73 | 6.95 | 6.09 | 6.38 |
| Gbar_D03_1862522 | T/G | 0.09 | 6.35 | - | - | - | - | - | Gbar_D03_2040228 | A/G | 0.18 | - | - | 6.40 | 6.79 | 6.03 | 6.32 |
| Gbar_D03_1872855 | G/C | 0.18 | - | - | 7.40 | 7.68 | 6.83 | 7.17 | Gbar_D03_2041970 | A/G | 0.18 | - | - | 6.82 | 7.22 | 6.33 | 6.65 |
| Gbar_D03_1873143 | T/C | 0.17 | - | - | 6.36 | 6.44 | - | - | Gbar_D03_2042357 | T/C | 0.18 | - | - | 7.57 | 7.69 | 6.79 | 7.14 |
| Gbar_D03_1876166 | G/C | 0.09 | 7.07 | 6.97 | - | - | - | - | Gbar_D03_2043305 | G/A | 0.18 | - | - | 7.18 | 7.54 | 6.71 | 7.04 |
| Gbar_D03_1878030 | T/C | 0.08 | 7.75 | 7.41 | - | - | 6.11 | 6.41 | Gbar_D03_2045627 | G/C | 0.18 | - | - | 6.63 | 6.71 | - | 6.16 |
| Gbar_D03_1878576 | G/A | 0.10 | 6.39 | - | - | - | - | - | Gbar_D03_2046359 | A/G | 0.18 | - | - | 6.34 | 6.83 | 6.14 | 6.44 |
| Gbar_D03_1879874 | T/C | 0.09 | 7.43 | 7.27 | 6.25 | 6.22 | 6.24 | 6.57 | Gbar_D03_2046523 | T/C | 0.18 | - | - | 7.43 | 6.84 | - | 6.29 |
| Gbar_D03_1881025 | G/A | 0.09 | 7.19 | 7.38 | - | 6.01 | 6.01 | 6.34 | Gbar_D03_2047187 | A/C | 0.18 | - | - | 7.26 | 7.33 | 6.40 | 6.74 |
| Gbar_D03_1882031 | T/C | 0.18 | - | - | 6.67 | 6.55 | 6.15 | 6.43 | Gbar_D03_2047669 | T/C | 0.18 | 6.30 | 6.30 | 8.03 | 8.19 | 7.39 | 7.74 |
| Gbar_D03_1884123 | A/G | 0.08 | 6.11 | - | - | - | - | - | Gbar_D03_2047730 | T/C | 0.17 | - | - | - | 6.12 | - | - |
| Gbar_D03_1886621 | A/G | 0.18 | - | - | 6.55 | 6.98 | 6.23 | 6.52 | Gbar_D03_2047804 | T/C | 0.17 | - | - | 7.03 | 7.24 | 6.51 | 6.81 |
| Gbar_D03_1888773 | G/T | 0.10 | 6.13 | - | - | - | - | - | Gbar_D03_2048430 | A/G | 0.18 | - | - | 6.24 | 6.64 | - | 6.09 |
| Gbar_D03_1890205 | T/G | 0.18 | - | - | 7.40 | 7.20 | 6.29 | 6.64 | Gbar_D03_2049119 | A/C | 0.18 | - | - | 7.98 | 7.96 | 7.03 | 7.39 |
| Gbar_D03_1892354 | T/G | 0.18 | - | - | 7.15 | 7.77 | 6.94 | 7.28 | Gbar_D03_2049740 | A/G | 0.18 | - | - | 6.63 | 7.08 | 6.10 | 6.41 |
| Gbar_D03_1895389 | G/A | 0.09 | 6.17 | - | - | - | - | - | Gbar_D03_2050294 | G/T | 0.18 | - | - | 7.47 | 7.89 | 7.03 | 7.37 |
| Gbar_D03_1896485 | T/C | 0.17 | - | - | 7.41 | 7.00 | 6.00 | 6.35 | Gbar_D03_2051583 | A/G | 0.18 | - | - | 6.23 | 6.50 | - | 6.01 |
| Gbar_D03_1896583 | T/C | 0.09 | 6.02 | - | - | - | - | - | Gbar_D03_2051748 | T/C | 0.18 | - | - | 6.78 | 7.18 | 6.39 | 6.72 |
| Gbar_D03_1897259 | T/C | 0.17 | - | - | 6.24 | 6.25 | - | - | Gbar_D03_2052908 | A/G | 0.18 | - | - | 7.27 | 7.31 | 6.36 | 6.70 |
| Gbar_D03_1899807 | G/A | 0.09 | 6.07 | - | - | - | - | - | Gbar_D03_2053671 | A/G | 0.17 | - | - | 6.22 | 6.53 | - | - |
| Gbar_D03_1901449 | G/A | 0.18 | - | - | 6.22 | 6.60 | - | 6.02 | Gbar_D03_2054298 | T/C | 0.18 | - | - | 7.25 | 7.44 | 6.51 | 6.84 |
| Gbar_D03_1903013 | T/C | 0.18 | - | - | 6.84 | 7.21 | 6.41 | 6.72 | Gbar_D03_2054754 | T/C | 0.18 | - | - | 6.13 | 6.25 | - | - |
| Gbar_D03_1904562 | C/T | 0.17 | - | - | 6.47 | 6.82 | - | 6.27 | Gbar_D03_2055339 | C/T | 0.18 | - | - | 7.31 | 7.72 | 6.85 | 7.20 |
| Gbar_D03_1904856 | A/G | 0.18 | - | - | 6.87 | 7.37 | 6.45 | 6.77 | Gbar_D03_2056886 | C/A | 0.09 | 6.61 | 6.78 | - | - | - | - |
| Gbar_D03_1907553 | T/A | 0.20 | - | - | - | 6.08 | - | - | Gbar_D03_2057422 | G/A | 0.18 | - | - | 7.76 | 7.84 | 6.90 | 7.24 |
| Gbar_D03_1910529 | A/G | 0.46 | 6.07 | - | - | - | - | - | Gbar_D03_2057743 | C/T | 0.09 | 7.54 | 7.24 | - | - | - | 6.12 |
| Gbar_D03_1912152 | T/C | 0.17 | - | - | 6.52 | 6.76 | 6.13 | 6.41 | Gbar_D03_2058409 | T/A | 0.10 | 6.05 | - | - | - | - | - |
| Gbar_D03_1918313 | T/C | 0.15 | - | - | 6.26 | - | - | - | Gbar_D03_2060260 | C/A | 0.18 | - | - | 7.24 | 9.13 | 8.14 | 8.48 |
| Gbar_D03_1918318 | C/A | 0.08 | 6.99 | 6.63 | - | - | - | - | Gbar_D03_2068470 | T/A | 0.09 | 6.14 | - | - | - | - | - |
| Gbar_D03_1918338 | A/G | 0.09 | 6.76 | 6.29 | - | - | - | - | Gbar_D03_2070020 | G/A | 0.09 | 6.23 | - | - | - | - | - |
| Gbar_D03_1918360 | G/A | 0.09 | 6.46 | - | - | - | - | - | Gbar_D03_2070146 | C/T | 0.09 | 6.08 | - | - | - | - | - |
| Gbar_D03_1918465 | C/A | 0.09 | 7.13 | 6.48 | - | - | - | - | Gbar_D03_2070493 | T/C | 0.18 | - | - | 6.72 | 6.87 | 6.11 | 6.43 |
| Gbar_D03_1921525 | T/C | 0.17 | - | - | 6.35 | 6.82 | - | 6.25 | Gbar_D03_2070820 | A/T | 0.19 | - | - | 6.45 | 7.28 | 6.37 | 6.68 |
| Gbar_D03_1922237 | A/T | 0.08 | 7.24 | 7.25 | - | - | - | 6.28 | Gbar_D03_2072242 | A/C | 0.18 | - | - | 7.59 | 7.73 | 6.69 | 7.04 |
| Gbar_D03_1922302 | T/C | 0.09 | 6.59 | - | - | - | - | - | Gbar_D03_2072293 | T/C | 0.18 | - | - | 7.86 | 8.06 | 7.01 | 7.39 |
| Gbar_D03_1922310 | A/G | 0.08 | 6.56 | - | - | - | - | - | Gbar_D03_2073552 | G/A | 0.18 | - | - | 6.94 | 7.38 | 6.52 | 6.85 |
| Gbar_D03_1922363 | G/A | 0.09 | 7.00 | 6.98 | - | - | - | - | Gbar_D03_2081936 | T/G | 0.08 | 7.27 | 7.48 | - | - | - | - |
| Gbar_D03_1922408 | T/C | 0.09 | 7.08 | 7.13 | - | - | - | 6.16 | Gbar_D03_2081948 | A/G | 0.08 | 7.10 | 7.71 | - | - | - | - |
| Gbar_D03_1922876 | T/C | 0.18 | - | - | 7.86 | 7.73 | 6.70 | 7.08 | Gbar_D03_2081955 | G/A | 0.08 | 6.24 | 6.19 | - | - | - | - |
| Gbar_D03_1923098 | A/G | 0.08 | 7.06 | 7.07 | - | - | - | 6.17 | Gbar_D03_2085912 | T/C | 0.18 | - | - | 7.19 | 7.68 | 6.67 | 7.01 |
| Gbar_D03_1923348 | C/A | 0.18 | - | - | 6.98 | 7.30 | 6.41 | 6.74 | Gbar_D03_2096694 | T/A | 0.18 | - | - | 6.09 | 6.38 | - | 6.05 |
| Gbar_D03_1924417 | A/G | 0.17 | - | - | 6.63 | 7.08 | 6.11 | 6.46 | Gbar_D03_2112513 | G/A | 0.08 | 6.82 | 6.45 | - | - | - | - |
| Gbar_D03_1926752 | T/C | 0.08 | 6.62 | 6.74 | - | - | - | - | Gbar_D03_2112544 | G/A | 0.08 | 6.35 | 6.10 | - | - | - | - |
| Gbar_D03_1926776 | G/A | 0.08 | 6.75 | 6.65 | - | - | - | - | Gbar_D03_2125937 | T/C | 0.09 | 6.48 | 6.54 | - | - | - | - |
| Gbar_D03_1926816 | A/G | 0.09 | 6.07 | - | - | - | - | - | Gbar_D03_2129927 | G/A | 0.08 | 6.92 | 6.47 | - | - | - | - |
| Gbar_D03_1926884 | G/A | 0.09 | 6.16 | 6.03 | - | - | - | - | Gbar_D03_2130833 | C/G | 0.08 | 6.66 | 6.47 | - | - | - | - |
| Gbar_D03_1928210 | C/G | 0.10 | 6.58 | - | - | - | - | - | Gbar_D03_2137861 | A/G | 0.08 | 7.10 | 6.95 | - | - | - | - |
| Gbar_D03_1928539 | T/C | 0.09 | 6.24 | - | - | - | - | - | Gbar_D03_2179602 | C/T | 0.10 | 6.03 | - | - | - | - | - |
| Gbar_D03_1928629 | T/C | 0.18 | - | - | 7.82 | 7.75 | 6.82 | 7.17 | Gbar_D03_2195873 | A/T | 0.46 | 6.08 | - | - | - | - | - |
| Gbar_D03_1928830 | G/T | 0.09 | 6.92 | 6.77 | - | - | - | - | Gbar_D03_220809 | A/T | 0.08 | 6.22 | 6.58 | - | - | - | - |
| Gbar_D03_1930207 | A/G | 0.18 | - | - | 6.86 | 7.01 | 6.13 | 6.45 | Gbar_D03_220931 | A/G | 0.08 | - | 6.46 | - | - | - | - |
| Gbar_D03_1931209 | T/A | 0.18 | - | - | 6.58 | 6.95 | 6.24 | 6.54 | Gbar_D03_220948 | G/C | 0.08 | 6.07 | 6.62 | - | - | - | - |
| Gbar_D03_1940049 | T/G | 0.18 | - | - | 6.13 | 6.57 | - | 6.03 | Gbar_D03_221262 | T/C | 0.08 | - | 6.48 | - | - | - | - |
| Gbar_D03_1941440 | A/T | 0.09 | 6.94 | 6.98 | - | - | 6.02 | 6.33 | Gbar_D03_2214857 | A/G | 0.16 | - | 6.51 | 7.40 | 8.58 | 7.98 | 8.32 |
| Gbar_D03_1956848 | T/C | 0.09 | 6.45 | 6.26 | - | - | - | - | Gbar_D03_2215669 | A/T | 0.17 | 6.51 | 6.94 | 7.89 | 9.01 | 8.42 | 8.77 |
| Gbar_D03_1956911 | A/T | 0.18 | - | - | 6.41 | 6.99 | 6.01 | 6.32 | Gbar_D03_222160 | T/C | 0.08 | 6.20 | 6.63 | - | - | - | - |
| Gbar_D03_1957237 | T/G | 0.09 | 7.38 | 7.42 | 6.14 | - | - | 6.32 | Gbar_D03_222186 | T/C | 0.08 | 6.09 | 6.90 | - | - | - | - |
| Gbar_D03_1957546 | A/G | 0.18 | - | - | 7.07 | 7.41 | 6.62 | 6.95 | Gbar_D03_222231 | A/G | 0.08 | 6.30 | 7.01 | - | - | - | - |
| Gbar_D03_1960995 | C/T | 0.09 | 6.15 | - | - | - | - | - | Gbar_D03_222232 | A/G | 0.08 | 6.30 | 7.01 | - | - | - | - |
| Gbar_D03_1963579 | G/A | 0.18 | - | - | 6.94 | 7.52 | 6.51 | 6.84 | Gbar_D03_2222437 | T/C | 0.09 | 6.02 | - | - | - | - | - |
| Gbar_D03_1965407 | A/G | 0.17 | - | - | - | 6.53 | - | - | Gbar_D03_2222499 | T/C | 0.16 | - | 6.43 | 6.97 | 8.07 | 7.86 | 8.13 |
| Gbar_D03_1970210 | A/T | 0.18 | - | - | 7.76 | 7.72 | 6.76 | 7.12 | Gbar_D03_222249 | A/G | 0.08 | 6.40 | 7.04 | - | - | - | - |
| Gbar_D03_1975755 | A/G | 0.18 | - | - | - | 6.44 | - | - | Gbar_D03_2222921 | A/G | 0.17 | - | 6.48 | 7.44 | 8.44 | 7.76 | 8.09 |
| Gbar_D03_1980162 | A/T | 0.12 | 6.69 | 6.49 | 6.96 | 7.53 | 6.99 | 7.34 | Gbar_D03_2223697 | G/T | 0.09 | 6.25 | 6.34 | - | - | - | - |
| Gbar_D03_1980968 | A/G | 0.18 | - | - | 6.59 | 6.86 | 6.41 | 6.68 | Gbar_D03_2224222 | A/G | 0.20 | - | 6.30 | 7.06 | 8.90 | 8.40 | 8.72 |
| Gbar_D03_1982034 | T/C | 0.18 | - | - | 6.30 | 6.63 | - | - | Gbar_D03_2224946 | A/G | 0.16 | 6.43 | 6.82 | 6.96 | 8.18 | 7.87 | 8.16 |
| Gbar_D03_1984614 | G/A | 0.17 | - | - | 6.16 | 6.28 | - | - | Gbar_D03_2224965 | A/G | 0.17 | - | - | 7.23 | 8.56 | 8.07 | 8.37 |
| Gbar_D03_1984911 | T/C | 0.18 | - | - | 7.89 | 7.99 | 6.94 | 7.31 | Gbar_D03_222590 | C/G | 0.08 | - | 6.43 | - | - | - | - |
| Gbar_D03_1989223 | C/T | 0.09 | 6.94 | 6.82 | - | - | - | 6.04 | Gbar_D03_222591 | C/G | 0.08 | 6.34 | 6.65 | - | - | - | - |
| Gbar_D03_1989306 | C/T | 0.09 | 6.54 | 6.12 | - | - | - | - | Gbar_D03_222616 | A/G | 0.08 | 6.50 | 6.58 | - | - | - | - |
| Gbar_D03_1989485 | T/C | 0.18 | 6.45 | 6.58 | 7.55 | 7.75 | 7.11 | 7.46 | Gbar_D03_222655 | G/T | 0.08 | 6.69 | 7.17 | - | - | - | - |
| Gbar_D03_1989700 | A/C | 0.17 | - | - | 7.15 | 7.41 | 6.57 | 6.90 | Gbar_D03_222693 | T/C | 0.07 | 6.10 | 6.90 | - | - | - | - |
| Gbar_D03_1989856 | T/G | 0.10 | 6.10 | - | - | - | - | - | Gbar_D03_2228451 | A/G | 0.14 | 6.54 | 6.05 | 6.62 | 8.01 | 7.83 | 8.12 |
| Gbar_D03_1990362 | A/G | 0.18 | - | - | 7.50 | 7.80 | 6.88 | 7.24 | Gbar_D03_222937 | G/A | 0.08 | 6.59 | 7.00 | - | - | - | - |
| Gbar_D03_2004879 | C/T | 0.08 | 6.64 | 6.37 | - | - | - | - | Gbar_D03_222941 | C/A | 0.08 | 6.10 | 6.61 | - | - | - | - |
| Gbar_D03_2007434 | T/A | 0.18 | - | - | 6.75 | 7.24 | 6.39 | 6.71 | Gbar_D03_223181 | C/T | 0.08 | - | 6.62 | - | - | - | - |
| Gbar_D03_2008527 | A/G | 0.18 | - | - | 7.03 | 7.53 | 6.58 | 6.93 | Gbar_D03_223331 | G/A | 0.08 | - | 6.31 | - | - | - | - |
| Gbar_D03_2014620 | T/C | 0.18 | - | - | 7.12 | 7.58 | 6.66 | 6.99 | Gbar_D03_223405 | A/G | 0.07 | - | 6.28 | - | - | - | - |
| Gbar_D03_2014708 | A/G | 0.17 | - | - | 7.45 | 6.95 | 6.02 | 6.36 | Gbar_D03_223567 | C/A | 0.08 | - | 6.17 | - | - | - | - |
| Gbar_D03_2015383 | G/C | 0.18 | - | - | 6.91 | 7.04 | 6.22 | 6.54 | Gbar_D03_224081 | T/C | 0.08 | 6.13 | 6.54 | - | - | - | - |
| Gbar_D03_2015612 | T/C | 0.18 | - | - | 7.25 | 7.51 | 6.56 | 6.89 | Gbar_D03_224097 | C/T | 0.08 | 6.32 | 6.61 | - | - | - | - |
| Gbar_D03_2015714 | C/T | 0.18 | - | - | 6.39 | 6.64 | - | 6.12 | Gbar_D03_224098 | G/T | 0.08 | 6.47 | 6.87 | - | - | - | - |
| Gbar_D03_2019628 | C/T | 0.09 | 7.05 | 7.06 | - | - | - | 6.02 | Gbar_D03_224122 | T/C | 0.08 | 6.62 | 7.08 | - | - | - | - |
| Gbar_D03_2019774 | A/G | 0.17 | - | - | 6.60 | 7.10 | 6.22 | 6.54 | Gbar_D03_224295 | C/G | 0.08 | - | 6.60 | - | - | - | - |
| Gbar_D03_2019798 | T/C | 0.18 | - | - | 6.77 | 7.28 | 6.22 | 6.58 | Gbar_D03_224785 | C/T | 0.08 | - | 6.47 | - | - | - | - |
| Gbar_D03_2020134 | C/A | 0.18 | - | - | 6.18 | 6.58 | - | 6.08 | Gbar_D03_2249026 | C/T | 0.09 | 7.37 | 7.28 | 6.01 | - | - | 6.09 |
| Gbar_D03_2022329 | C/T | 0.18 | - | - | 6.18 | 6.23 | - | - | Gbar_D03_2249706 | T/C | 0.15 | 6.07 | 6.85 | 6.89 | 7.98 | 7.91 | 8.20 |
| Gbar_D03_2022615 | T/G | 0.09 | 7.06 | 6.98 | - | - | - | - | Gbar_D03_2255845 | T/G | 0.14 | 6.68 | 6.29 | 6.79 | 7.81 | 7.47 | 7.80 |
| Gbar_D03_2022638 | G/A | 0.08 | - | 6.10 | - | - | - | - | Gbar_D03_2255926 | T/C | 0.14 | 6.17 | - | - | 6.80 | 6.69 | 6.94 |
| Gbar_D03_2022860 | T/G | 0.18 | - | - | 6.46 | 6.84 | - | 6.11 | Gbar_D03_2256558 | T/A | 0.14 | 6.55 | 6.15 | - | 7.43 | 7.26 | 7.53 |
| Gbar_D03_2022908 | T/C | 0.18 | - | - | 7.05 | 7.30 | 6.46 | 6.78 | Gbar_D03_2256560 | T/A | 0.14 | 7.13 | 6.79 | 6.73 | 8.07 | 7.83 | 8.15 |
| Gbar_D03_2023153 | C/T | 0.18 | - | - | - | 6.28 | - | - | Gbar_D03_225809 | T/C | 0.08 | - | 6.02 | - | - | - | - |
| Gbar_D03_2023405 | T/A | 0.18 | - | - | 6.50 | 6.67 | - | 6.29 | Gbar_D03_2259062 | G/T | 0.08 | 6.16 | - | - | - | - | - |
| Gbar_D03_2023546 | T/C | 0.18 | 6.13 | - | 7.87 | 8.05 | 7.11 | 7.47 | Gbar_D03_226153 | A/T | 0.08 | 6.15 | 6.55 | - | - | - | - |
| Gbar_D03_2025058 | T/C | 0.18 | - | - | 6.14 | 6.91 | 6.42 | 6.67 | Gbar_D03_226586 | C/T | 0.07 | 6.42 | 6.66 | - | - | - | - |
| Gbar_D03_2028897 | T/G | 0.17 | - | - | 6.15 | 6.44 | - | - | Gbar_D03_2268413 | A/C | 0.09 | 6.07 | - | - | - | - | - |
| Gbar_D03_2030544 | A/G | 0.09 | 6.22 | - | - | - | - | - | Gbar_D03_227406 | C/T | 0.08 | 6.19 | 6.98 | - | - | - | - |
| Gbar_D03_2030758 | T/C | 0.18 | - | - | 7.05 | 7.23 | 6.42 | 6.75 | Gbar_D03_227599 | A/T | 0.08 | 6.58 | 7.35 | - | - | - | - |
| Gbar_D03_2031835 | T/G | 0.18 | - | - | 7.60 | 8.09 | 7.17 | 7.53 | Gbar_D03_228487 | G/A | 0.07 | - | 6.03 | - | - | - | - |
| Gbar_D03_2034483 | T/G | 0.18 | - | - | 6.60 | 6.85 | 6.03 | 6.32 | Gbar_D03_228490 | T/G | 0.07 | - | 6.06 | - | - | - | - |
| Gbar_D03_2034515 | T/C | 0.18 | - | - | 7.21 | 7.06 | 6.15 | 6.50 | Gbar_D03_228548 | G/A | 0.07 | 6.38 | 6.92 | - | - | - | - |
| Gbar_D03_2034622 | T/C | 0.18 | - | - | 7.59 | 7.61 | 6.61 | 6.98 | Gbar_D03_228570 | A/G | 0.08 | 6.43 | 6.80 | - | - | - | - |
| Gbar_D03_2034913 | T/C | 0.17 | - | - | 6.59 | 6.96 | 6.02 | 6.35 | Gbar_D03_228596 | A/G | 0.08 | 6.42 | 6.73 | - | - | - | - |
| Gbar_D03_2035736 | G/C | 0.09 | 6.54 | - | - | - | - | - | Gbar_D03_228613 | A/G | 0.07 | - | 6.05 | - | - | - | - |

**Table S3.** Summary of the total associated SNPs identified through GWAS for *FW* resistance in Sea Island cotton. (Continued)

| SNP | Ref/Alt | MAF | -log (P-value) in different environments | | | | | | SNP | Ref/Alt | MAF | -log (P-value) in different environments | | | | | |
| --- | --- | --- | --- | --- | --- | --- | --- | --- | --- | --- | --- | --- | --- | --- | --- | --- | --- |
|  |  |  | 2015 | 2016 | 2018 | 2019 | Mean | BLUP |  |  |  | 2015 | 2016 | 2018 | 2019 | Mean | BLUP |
| Gbar_D03_228651 | A/C | 0.08 | - | 6.24 | - | - | - | - | Gbar_D03_281099 | C/T | 0.08 | 6.00 | 6.80 | - | - | - | - |
| Gbar_D03_228685 | T/C | 0.07 | - | 6.29 | - | - | - | - | Gbar_D03_281165 | G/A | 0.08 | - | 6.66 | - | - | - | - |
| Gbar_D03_228760 | C/T | 0.07 | - | 6.08 | - | - | - | - | Gbar_D03_281564 | T/G | 0.08 | 6.16 | 6.73 | - | - | - | - |
| Gbar_D03_228776 | C/T | 0.07 | - | 6.21 | - | - | - | - | Gbar_D03_284501 | G/A | 0.08 | 6.17 | 6.36 | - | - | - | - |
| Gbar_D03_228780 | C/A | 0.07 | - | 6.17 | - | - | - | - | Gbar_D03_285695 | T/G | 0.07 | 6.42 | 6.72 | - | - | - | - |
| Gbar_D03_228815 | T/C | 0.08 | 6.04 | 6.88 | - | - | - | - | Gbar_D03_285970 | G/A | 0.08 | 6.44 | 7.01 | - | - | - | - |
| Gbar_D03_2289243 | A/G | 0.14 | - | - | - | 6.54 | 6.49 | 6.67 | Gbar_D03_287296 | T/A | 0.08 | 6.33 | 6.95 | - | - | - | - |
| Gbar_D03_228992 | T/G | 0.08 | - | 6.16 | - | - | - | - | Gbar_D03_289432 | C/A | 0.08 | 6.31 | 7.07 | - | - | - | - |
| Gbar_D03_229404 | A/G | 0.07 | - | 6.09 | - | - | - | - | Gbar_D03_290201 | G/A | 0.08 | 6.54 | 7.04 | - | - | - | - |
| Gbar_D03_229831 | C/T | 0.08 | 6.20 | 6.91 | - | - | - | - | Gbar_D03_290416 | A/T | 0.08 | 6.82 | 6.67 | - | - | - | - |
| Gbar_D03_229844 | T/C | 0.08 | 6.20 | 6.86 | - | - | - | - | Gbar_D03_290586 | T/A | 0.08 | 6.61 | 7.07 | - | - | - | - |
| Gbar_D03_230010 | T/C | 0.08 | - | 6.81 | - | - | - | - | Gbar_D03_291025 | A/T | 0.08 | 6.51 | 6.51 | - | - | - | - |
| Gbar_D03_230011 | G/C | 0.08 | - | 6.55 | - | - | - | - | Gbar_D03_291710 | G/A | 0.08 | 6.32 | 6.59 | - | - | - | - |
| Gbar_D03_230025 | C/G | 0.08 | - | 6.62 | - | - | - | - | Gbar_D03_291826 | G/A | 0.08 | - | 6.14 | - | - | - | - |
| Gbar_D03_230553 | C/G | 0.08 | 6.49 | 7.16 | - | - | - | - | Gbar_D03_292036 | A/T | 0.07 | - | 6.59 | - | - | - | - |
| Gbar_D03_230585 | A/G | 0.08 | 6.11 | 6.77 | - | - | - | - | Gbar_D03_292283 | T/G | 0.08 | - | 6.37 | - | - | - | - |
| Gbar_D03_231449 | T/A | 0.07 | - | 6.15 | - | - | - | - | Gbar_D03_293205 | A/T | 0.08 | 6.15 | 6.12 | - | - | - | - |
| Gbar_D03_231767 | C/A | 0.08 | 6.17 | 6.74 | - | - | - | - | Gbar_D03_293228 | A/G | 0.08 | 6.06 | 6.42 | - | - | - | - |
| Gbar_D03_231982 | A/T | 0.08 | 6.01 | 6.35 | - | - | - | - | Gbar_D03_293274 | C/T | 0.08 | - | 6.40 | - | - | - | - |
| Gbar_D03_232520 | C/A | 0.08 | 6.06 | 6.11 | - | - | - | - | Gbar_D03_293403 | T/C | 0.08 | - | 6.16 | - | - | - | - |
| Gbar_D03_232691 | T/C | 0.08 | - | 6.40 | - | - | - | - | Gbar_D03_293435 | T/G | 0.08 | - | 6.22 | - | - | - | - |
| Gbar_D03_232810 | G/A | 0.09 | 6.25 | 6.95 | - | - | - | - | Gbar_D03_295398 | T/C | 0.08 | - | 6.59 | - | - | - | - |
| Gbar_D03_233018 | T/C | 0.08 | 6.18 | 6.55 | - | - | - | - | Gbar_D03_295844 | A/T | 0.08 | 6.03 | 6.33 | - | - | - | - |
| Gbar_D03_233033 | G/A | 0.08 | - | 6.43 | - | - | - | - | Gbar_D03_295872 | T/A | 0.08 | - | 6.19 | - | - | - | - |
| Gbar_D03_233232 | C/A | 0.07 | - | 6.22 | - | - | - | - | Gbar_D03_296209 | G/T | 0.08 | 6.63 | 7.16 | - | - | - | - |
| Gbar_D03_233239 | A/T | 0.07 | - | 6.20 | - | - | - | - | Gbar_D03_296278 | A/T | 0.08 | 6.52 | 7.07 | - | - | - | - |
| Gbar_D03_233431 | G/T | 0.07 | 6.36 | 6.55 | - | - | - | - | Gbar_D03_296398 | C/A | 0.08 | - | 6.35 | - | - | - | - |
| Gbar_D03_233938 | A/T | 0.08 | - | 6.25 | - | - | - | - | Gbar_D03_296456 | G/A | 0.08 | 6.03 | 6.36 | - | - | - | - |
| Gbar_D03_236618 | C/T | 0.08 | 6.37 | 6.98 | - | - | - | - | Gbar_D03_296723 | G/A | 0.09 | - | 6.22 | - | - | - | - |
| Gbar_D03_236653 | A/G | 0.08 | 6.53 | 7.34 | - | - | - | - | Gbar_D03_296879 | T/G | 0.08 | - | 6.20 | - | - | - | - |
| Gbar_D03_236777 | T/C | 0.08 | 6.66 | 6.85 | - | - | - | - | Gbar_D03_297638 | C/T | 0.08 | 6.38 | 6.58 | - | - | - | - |
| Gbar_D03_2368579 | T/A | 0.17 | - | - | - | 6.63 | 6.43 | 6.53 | Gbar_D03_297696 | C/G | 0.08 | 6.19 | 6.87 | - | - | - | - |
| Gbar_D03_236894 | C/T | 0.08 | - | 6.38 | - | - | - | - | Gbar_D03_297725 | T/C | 0.08 | 6.62 | 7.12 | - | - | - | - |
| Gbar_D03_237064 | G/T | 0.08 | 6.18 | 6.52 | - | - | - | - | Gbar_D03_297808 | G/C | 0.08 | 6.62 | 6.95 | - | - | - | - |
| Gbar_D03_237071 | C/A | 0.08 | 6.41 | 6.74 | - | - | - | - | Gbar_D03_298221 | A/T | 0.08 | 6.19 | 6.54 | - | - | - | - |
| Gbar_D03_237378 | G/A | 0.08 | 6.95 | 7.33 | - | - | - | - | Gbar_D03_298574 | C/A | 0.07 | - | 6.01 | - | - | - | - |
| Gbar_D03_237484 | C/T | 0.08 | - | 6.13 | - | - | - | - | Gbar_D03_298740 | G/A | 0.08 | 6.14 | 6.88 | - | - | - | - |
| Gbar_D03_237550 | T/A | 0.08 | 6.23 | 6.70 | - | - | - | - | Gbar_D03_298944 | T/C | 0.08 | 6.09 | 6.43 | - | - | - | - |
| Gbar_D03_237715 | C/T | 0.08 | 6.01 | 6.45 | - | - | - | - | Gbar_D03_300323 | A/T | 0.08 | - | 6.46 | - | - | - | - |
| Gbar_D03_237734 | G/A | 0.08 | - | 6.48 | - | - | - | - | Gbar_D03_300361 | G/A | 0.08 | 6.15 | 6.79 | - | - | - | - |
| Gbar_D03_237828 | A/G | 0.07 | - | 6.31 | - | - | - | - | Gbar_D03_302594 | A/G | 0.07 | 6.53 | 6.60 | - | - | - | - |
| Gbar_D03_237923 | G/A | 0.07 | - | 6.05 | - | - | - | - | Gbar_D03_305070 | T/G | 0.08 | 6.26 | 6.98 | - | - | - | - |
| Gbar_D03_238573 | T/C | 0.07 | - | 6.05 | - | - | - | - | Gbar_D03_305083 | A/G | 0.08 | 6.29 | 7.09 | - | - | - | - |
| Gbar_D03_238766 | G/T | 0.07 | - | 6.16 | - | - | - | - | Gbar_D03_306408 | A/G | 0.08 | - | 6.01 | - | - | - | - |
| Gbar_D03_239840 | C/T | 0.07 | 6.03 | 6.05 | - | - | - | - | Gbar_D03_306763 | T/C | 0.08 | - | 6.20 | - | - | - | - |
| Gbar_D03_240787 | T/C | 0.08 | - | 6.37 | - | - | - | - | Gbar_D03_307265 | C/T | 0.08 | - | 6.53 | - | - | - | - |
| Gbar_D03_241600 | A/G | 0.08 | - | 6.15 | - | - | - | - | Gbar_D03_307978 | A/G | 0.07 | 6.03 | 6.53 | - | - | - | - |
| Gbar_D03_241686 | G/A | 0.08 | - | 6.09 | - | - | - | - | Gbar_D03_307986 | G/A | 0.07 | 6.14 | 6.59 | - | - | - | - |
| Gbar_D03_241833 | A/C | 0.07 | - | 6.13 | - | - | - | - | Gbar_D03_308036 | C/A | 0.07 | - | 6.64 | - | - | - | - |
| Gbar_D03_241847 | A/G | 0.07 | - | 6.07 | - | - | - | - | Gbar_D03_308087 | T/C | 0.08 | 6.65 | 6.85 | - | - | - | - |
| Gbar_D03_242138 | G/A | 0.08 | 6.06 | 6.68 | - | - | - | - | Gbar_D03_309082 | G/T | 0.08 | - | 6.48 | - | - | - | - |
| Gbar_D03_242166 | T/A | 0.07 | 6.52 | 6.83 | - | - | - | - | Gbar_D03_310128 | T/C | 0.07 | - | 6.49 | - | - | - | - |
| Gbar_D03_242755 | C/T | 0.08 | - | 6.91 | - | - | - | - | Gbar_D03_310501 | T/C | 0.07 | 6.22 | 6.06 | - | - | - | - |
| Gbar_D03_243816 | G/T | 0.08 | 6.15 | 6.84 | - | - | - | - | Gbar_D03_310752 | T/C | 0.08 | - | 6.33 | - | - | - | - |
| Gbar_D03_244284 | C/T | 0.08 | 6.36 | 6.62 | - | - | - | - | Gbar_D03_311216 | T/G | 0.08 | - | 6.33 | - | - | - | - |
| Gbar_D03_244354 | C/T | 0.08 | - | 6.14 | - | - | - | - | Gbar_D03_311296 | T/G | 0.08 | - | 6.64 | - | - | - | - |
| Gbar_D03_245344 | C/T | 0.07 | - | 6.37 | - | - | - | - | Gbar_D03_311607 | A/T | 0.08 | - | 6.51 | - | - | - | - |
| Gbar_D03_245629 | A/G | 0.08 | 6.20 | 6.37 | - | - | - | - | Gbar_D03_311657 | T/C | 0.08 | - | 6.10 | - | - | - | - |
| Gbar_D03_245656 | G/A | 0.08 | - | 6.19 | - | - | - | - | Gbar_D03_311975 | G/A | 0.08 | - | 6.11 | - | - | - | - |
| Gbar_D03_245684 | C/A | 0.08 | 6.47 | 6.90 | - | - | - | - | Gbar_D03_314965 | A/G | 0.07 | - | 6.45 | - | - | - | - |
| Gbar_D03_245980 | A/T | 0.08 | 6.26 | 6.63 | - | - | - | - | Gbar_D03_315553 | C/G | 0.08 | 6.91 | 7.29 | - | - | - | - |
| Gbar_D03_246179 | C/G | 0.08 | - | 6.76 | - | - | - | - | Gbar_D03_315594 | A/G | 0.08 | 6.51 | 7.04 | - | - | - | - |
| Gbar_D03_246396 | G/T | 0.07 | 6.16 | 6.42 | - | - | - | - | Gbar_D03_317643 | T/G | 0.08 | 6.61 | 6.98 | - | - | - | - |
| Gbar_D03_246409 | C/A | 0.07 | 6.01 | 6.24 | - | - | - | - | Gbar_D03_317762 | G/A | 0.08 | 6.11 | 6.29 | - | - | - | - |
| Gbar_D03_246540 | T/C | 0.08 | 6.19 | 7.18 | - | - | - | - | Gbar_D03_317766 | G/A | 0.08 | 6.04 | 6.12 | - | - | - | - |
| Gbar_D03_247958 | A/G | 0.08 | 6.88 | 7.09 | - | - | - | - | Gbar_D03_317770 | A/G | 0.08 | - | 6.19 | - | - | - | - |
| Gbar_D03_248386 | A/G | 0.08 | - | 6.40 | - | - | - | - | Gbar_D03_325034 | C/T | 0.08 | 6.26 | 6.91 | - | - | - | - |
| Gbar_D03_248567 | A/G | 0.08 | 6.16 | 6.73 | - | - | - | - | Gbar_D03_325159 | A/G | 0.08 | - | 6.43 | - | - | - | - |
| Gbar_D03_248695 | T/C | 0.07 | - | 6.15 | - | - | - | - | Gbar_D03_325218 | T/A | 0.08 | - | 6.32 | - | - | - | - |
| Gbar_D03_250966 | C/T | 0.08 | 6.16 | 6.82 | - | - | - | - | Gbar_D03_326108 | C/T | 0.08 | 6.32 | 6.90 | - | - | - | - |
| Gbar_D03_251337 | T/C | 0.08 | - | 6.40 | - | - | - | - | Gbar_D03_326859 | G/C | 0.08 | - | 6.42 | - | - | - | - |
| Gbar_D03_251461 | C/G | 0.08 | - | 6.35 | - | - | - | - | Gbar_D03_327186 | T/A | 0.08 | - | 6.22 | - | - | - | - |
| Gbar_D03_251470 | C/G | 0.08 | - | 6.34 | - | - | - | - | Gbar_D03_327187 | T/A | 0.07 | 6.08 | 6.57 | - | - | - | - |
| Gbar_D03_251539 | C/G | 0.08 | - | 6.46 | - | - | - | - | Gbar_D03_327895 | A/G | 0.08 | 6.35 | 6.81 | - | - | - | - |
| Gbar_D03_252667 | G/T | 0.08 | 6.31 | 6.49 | - | - | - | - | Gbar_D03_328439 | C/T | 0.08 | - | 6.57 | - | - | - | - |
| Gbar_D03_253369 | G/T | 0.08 | - | 6.42 | - | - | - | - | Gbar_D03_328537 | A/T | 0.07 | - | 6.03 | - | - | - | - |
| Gbar_D03_254335 | T/A | 0.08 | 6.01 | 6.20 | - | - | - | - | Gbar_D03_335127 | A/C | 0.08 | 6.48 | 6.95 | - | - | - | - |
| Gbar_D03_255137 | C/T | 0.08 | 6.05 | 6.73 | - | - | - | - | Gbar_D03_335393 | T/A | 0.08 | - | 6.37 | - | - | - | - |
| Gbar_D03_255247 | C/T | 0.08 | 6.10 | 6.69 | - | - | - | - | Gbar_D03_336173 | T/C | 0.08 | 6.26 | 7.12 | - | - | - | - |
| Gbar_D03_256410 | T/C | 0.08 | 6.27 | 6.71 | - | - | - | - | Gbar_D03_336255 | A/G | 0.08 | 6.44 | 7.20 | - | - | - | - |
| Gbar_D03_257735 | G/C | 0.08 | 6.10 | 6.41 | - | - | - | - | Gbar_D03_336492 | A/G | 0.07 | - | 6.41 | - | - | - | - |
| Gbar_D03_258830 | C/A | 0.08 | 6.35 | 7.12 | - | - | - | - | Gbar_D03_337013 | A/C | 0.08 | - | 6.43 | - | - | - | - |
| Gbar_D03_259632 | C/G | 0.07 | - | 6.49 | - | - | - | - | Gbar_D03_337300 | A/T | 0.08 | 6.37 | 6.86 | - | - | - | - |
| Gbar_D03_261728 | C/T | 0.07 | - | 6.61 | - | - | - | - | Gbar_D03_337548 | T/G | 0.07 | 6.18 | 6.24 | - | - | - | - |
| Gbar_D03_263048 | T/C | 0.07 | - | 6.05 | - | - | - | - | Gbar_D03_337829 | G/T | 0.08 | 6.44 | 7.00 | - | - | - | - |
| Gbar_D03_263853 | C/T | 0.08 | 6.12 | 6.59 | - | - | - | - | Gbar_D03_337979 | C/G | 0.07 | - | 6.21 | - | - | - | - |
| Gbar_D03_263871 | C/G | 0.08 | - | 6.48 | - | - | - | - | Gbar_D03_338062 | C/T | 0.08 | - | 6.44 | - | - | - | - |
| Gbar_D03_264339 | A/G | 0.08 | - | 6.21 | - | - | - | - | Gbar_D03_338447 | A/T | 0.08 | 6.01 | 6.57 | - | - | - | - |
| Gbar_D03_264342 | C/T | 0.07 | - | 6.03 | - | - | - | - | Gbar_D03_338578 | C/T | 0.08 | 6.18 | 6.58 | - | - | - | - |
| Gbar_D03_264452 | C/T | 0.08 | - | 6.17 | - | - | - | - | Gbar_D03_339295 | C/T | 0.08 | 6.08 | 6.19 | - | - | - | - |
| Gbar_D03_264598 | A/T | 0.07 | 6.13 | 6.33 | - | - | - | - | Gbar_D03_339349 | C/A | 0.08 | 6.09 | 6.40 | - | - | - | - |
| Gbar_D03_264615 | A/G | 0.08 | 6.09 | 6.42 | - | - | - | - | Gbar_D03_340016 | A/G | 0.08 | - | 6.18 | - | - | - | - |
| Gbar_D03_265107 | T/A | 0.08 | 6.10 | 6.22 | - | - | - | - | Gbar_D03_341144 | T/C | 0.08 | - | 6.15 | - | - | - | - |
| Gbar_D03_266246 | A/C | 0.08 | 6.92 | 7.45 | - | - | - | - | Gbar_D03_341171 | G/A | 0.08 | 6.86 | 7.77 | - | - | - | - |
| Gbar_D03_267536 | A/G | 0.07 | - | 6.37 | - | - | - | - | Gbar_D03_341305 | C/A | 0.08 | 6.16 | 6.67 | - | - | - | - |
| Gbar_D03_267782 | T/C | 0.08 | 6.21 | 6.81 | - | - | - | - | Gbar_D03_341872 | G/A | 0.08 | - | 6.36 | - | - | - | - |
| Gbar_D03_267841 | A/T | 0.08 | 6.32 | 6.85 | - | - | - | - | Gbar_D03_342392 | T/A | 0.08 | 6.30 | 6.49 | - | - | - | - |
| Gbar_D03_267866 | A/G | 0.08 | 6.44 | 6.69 | - | - | - | - | Gbar_D03_342724 | A/G | 0.08 | 6.08 | 6.60 | - | - | - | - |
| Gbar_D03_268910 | T/A | 0.08 | 6.10 | 6.66 | - | - | - | - | Gbar_D03_342870 | A/G | 0.08 | 6.38 | 6.86 | - | - | - | - |
| Gbar_D03_269537 | A/T | 0.08 | - | 6.55 | - | - | - | - | Gbar_D03_343188 | C/G | 0.08 | 6.32 | 6.72 | - | - | - | - |
| Gbar_D03_269574 | T/A | 0.08 | - | 6.12 | - | - | - | - | Gbar_D03_343677 | T/C | 0.08 | 6.14 | 6.56 | - | - | - | - |
| Gbar_D03_275717 | C/T | 0.08 | - | 6.30 | - | - | - | - | Gbar_D03_344490 | T/C | 0.08 | 6.24 | 6.74 | - | - | - | - |
| Gbar_D03_276754 | G/A | 0.08 | 6.59 | 6.87 | - | - | - | - | Gbar_D03_344918 | T/A | 0.08 | - | 6.55 | - | - | - | - |
| Gbar_D03_276895 | G/A | 0.08 | 6.10 | 6.04 | - | - | - | - | Gbar_D03_345367 | A/G | 0.07 | - | 6.15 | - | - | - | - |
| Gbar_D03_276918 | A/T | 0.08 | 6.16 | 6.13 | - | - | - | - | Gbar_D03_345426 | G/A | 0.08 | 6.17 | 6.79 | - | - | - | - |
| Gbar_D03_276927 | A/T | 0.08 | 6.12 | 6.01 | - | - | - | - | Gbar_D03_346133 | A/T | 0.08 | 6.82 | 7.58 | - | - | - | - |
| Gbar_D03_277326 | A/T | 0.08 | 6.18 | 6.86 | - | - | - | - | Gbar_D03_346147 | G/T | 0.08 | 7.06 | 7.79 | - | - | - | - |
| Gbar_D03_280479 | T/C | 0.08 | 6.13 | 6.72 | - | - | - | - | Gbar_D03_346174 | A/T | 0.08 | 7.05 | 7.91 | - | - | - | - |
| Gbar_D03_280483 | T/A | 0.08 | 6.03 | 6.80 | - | - | - | - | Gbar_D03_347075 | G/A | 0.08 | 6.07 | 6.77 | - | - | - | - |
| Gbar_D03_280605 | C/T | 0.08 | 6.20 | 6.82 | - | - | - | - | Gbar_D03_347151 | T/C | 0.08 | 6.40 | 7.04 | - | - | - | - |

**Table S3.** Summary of the total associated SNPs identified through GWAS for *FW* resistance in Sea Island cotton. (Continued)

| SNP | Ref/Alt | MAF | -log (P-value) in different environments | | | | | | SNP | Ref/Alt | MAF | -log (P-value) in different environments | | | | | |
| --- | --- | --- | --- | --- | --- | --- | --- | --- | --- | --- | --- | --- | --- | --- | --- | --- | --- |
|  |  |  | 2015 | 2016 | 2018 | 2019 | Mean | BLUP |  |  |  | 2015 | 2016 | 2018 | 2019 | Mean | BLUP |
| Gbar_D03_348413 | G/A | 0.08 | - | 6.59 | - | - | - | - | Gbar_D03_400518 | C/T | 0.08 | - | 6.30 | - | - | - | - |
| Gbar_D03_349195 | A/G | 0.08 | 6.08 | 6.75 | - | - | - | - | Gbar_D03_401365 | G/A | 0.08 | 6.47 | 6.67 | - | - | - | - |
| Gbar_D03_350601 | G/A | 0.08 | - | 6.75 | - | - | - | - | Gbar_D03_401374 | A/G | 0.07 | - | 6.60 | - | - | - | - |
| Gbar_D03_350909 | G/A | 0.08 | - | 6.20 | - | - | - | - | Gbar_D03_401592 | T/C | 0.08 | 6.47 | 7.05 | - | - | - | - |
| Gbar_D03_351193 | C/G | 0.08 | 6.64 | 7.53 | - | - | - | - | Gbar_D03_401895 | G/A | 0.08 | - | 6.03 | - | - | - | - |
| Gbar_D03_351674 | T/C | 0.07 | - | 6.18 | - | - | - | - | Gbar_D03_402831 | C/T | 0.08 | 6.27 | 6.83 | - | - | - | - |
| Gbar_D03_351774 | G/A | 0.08 | - | 6.06 | - | - | - | - | Gbar_D03_402897 | G/A | 0.08 | 6.61 | 6.89 | - | - | - | - |
| Gbar_D03_351798 | G/A | 0.07 | 6.48 | 6.08 | - | - | - | - | Gbar_D03_403327 | T/C | 0.08 | 6.34 | 6.65 | - | - | - | - |
| Gbar_D03_351883 | A/G | 0.08 | 6.47 | 6.89 | - | - | - | - | Gbar_D03_403389 | G/C | 0.08 | - | 6.28 | - | - | - | - |
| Gbar_D03_351895 | A/G | 0.08 | 6.59 | 6.90 | - | - | - | - | Gbar_D03_403792 | A/C | 0.08 | 6.13 | 6.84 | - | - | - | - |
| Gbar_D03_351917 | G/A | 0.08 | 6.19 | 6.53 | - | - | - | - | Gbar_D03_405154 | G/T | 0.08 | 6.63 | 6.90 | - | - | - | - |
| Gbar_D03_351927 | A/G | 0.08 | 6.57 | 6.80 | - | - | - | - | Gbar_D03_405198 | G/A | 0.08 | 6.19 | 6.36 | - | - | - | - |
| Gbar_D03_352115 | A/G | 0.08 | - | 6.26 | - | - | - | - | Gbar_D03_405384 | G/T | 0.08 | - | 6.29 | - | - | - | - |
| Gbar_D03_353095 | T/C | 0.08 | 6.91 | 7.01 | - | - | - | - | Gbar_D03_405700 | A/T | 0.08 | 6.39 | 6.85 | - | - | - | - |
| Gbar_D03_353785 | T/C | 0.07 | 6.13 | 6.41 | - | - | - | - | Gbar_D03_405726 | A/G | 0.08 | 6.47 | 7.03 | - | - | - | - |
| Gbar_D03_353967 | A/G | 0.08 | - | 6.10 | - | - | - | - | Gbar_D03_405730 | A/T | 0.08 | 6.46 | 6.99 | - | - | - | - |
| Gbar_D03_354247 | A/G | 0.08 | 6.01 | 6.43 | - | - | - | - | Gbar_D03_406053 | G/A | 0.08 | 6.51 | 7.38 | - | - | - | - |
| Gbar_D03_355098 | C/T | 0.07 | 6.59 | 6.75 | - | - | - | - | Gbar_D03_406139 | T/C | 0.08 | 6.43 | 7.30 | - | - | - | - |
| Gbar_D03_357211 | T/C | 0.08 | 6.19 | 6.64 | - | - | - | - | Gbar_D03_406564 | C/T | 0.08 | 6.14 | 6.68 | - | - | - | - |
| Gbar_D03_357800 | G/A | 0.08 | 6.06 | 6.48 | - | - | - | - | Gbar_D03_406637 | C/A | 0.08 | 6.55 | 7.02 | - | - | - | - |
| Gbar_D03_359041 | G/A | 0.08 | 6.29 | 6.87 | - | - | - | - | Gbar_D03_408929 | G/A | 0.08 | - | 6.15 | - | - | - | - |
| Gbar_D03_359727 | A/C | 0.08 | - | 6.00 | - | - | - | - | Gbar_D03_409395 | A/T | 0.08 | - | 6.60 | - | - | - | - |
| Gbar_D03_359854 | A/C | 0.08 | - | 6.91 | - | - | - | - | Gbar_D03_409507 | G/A | 0.08 | - | 6.64 | - | - | - | - |
| Gbar_D03_360080 | C/T | 0.07 | - | 6.16 | - | - | - | - | Gbar_D03_409648 | T/G | 0.08 | - | 6.61 | - | - | - | - |
| Gbar_D03_361972 | G/A | 0.08 | 6.30 | 6.85 | - | - | - | - | Gbar_D03_409945 | C/T | 0.08 | 6.83 | 7.33 | - | - | - | - |
| Gbar_D03_362303 | C/T | 0.08 | 6.26 | 6.83 | - | - | - | - | Gbar_D03_410090 | C/T | 0.08 | - | 6.75 | - | - | - | - |
| Gbar_D03_362551 | T/G | 0.08 | - | 6.73 | - | - | - | - | Gbar_D03_410594 | G/A | 0.08 | - | 6.04 | - | - | - | - |
| Gbar_D03_362767 | G/A | 0.08 | 6.32 | 6.85 | - | - | - | - | Gbar_D03_410928 | G/A | 0.08 | 6.41 | 6.97 | - | - | - | - |
| Gbar_D03_362782 | G/A | 0.08 | - | 6.41 | - | - | - | - | Gbar_D03_411252 | T/G | 0.08 | 6.23 | 6.38 | - | - | - | - |
| Gbar_D03_362973 | A/G | 0.08 | - | 6.18 | - | - | - | - | Gbar_D03_411418 | A/G | 0.08 | 6.11 | 6.59 | - | - | - | - |
| Gbar_D03_363140 | G/T | 0.08 | 6.29 | 6.94 | - | - | - | - | Gbar_D03_411439 | T/C | 0.08 | 6.15 | 6.66 | - | - | - | - |
| Gbar_D03_363238 | G/A | 0.08 | 6.06 | 6.42 | - | - | - | - | Gbar_D03_411532 | A/G | 0.08 | - | 6.04 | - | - | - | - |
| Gbar_D03_363257 | C/A | 0.08 | - | 6.40 | - | - | - | - | Gbar_D03_411581 | G/A | 0.08 | 6.23 | 6.15 | - | - | - | - |
| Gbar_D03_363323 | T/A | 0.08 | - | 6.11 | - | - | - | - | Gbar_D03_411885 | C/T | 0.07 | 6.22 | 6.43 | - | - | - | - |
| Gbar_D03_363877 | A/G | 0.07 | - | 6.55 | - | - | - | - | Gbar_D03_412682 | C/A | 0.07 | 6.30 | 6.78 | - | - | - | - |
| Gbar_D03_364835 | C/T | 0.08 | - | 6.40 | - | - | - | - | Gbar_D03_412737 | G/A | 0.08 | 6.61 | 7.13 | - | - | - | - |
| Gbar_D03_364890 | C/T | 0.08 | - | 6.40 | - | - | - | - | Gbar_D03_412749 | A/C | 0.08 | 6.54 | 6.69 | - | - | - | - |
| Gbar_D03_364949 | C/T | 0.08 | - | 6.04 | - | - | - | - | Gbar_D03_413017 | T/A | 0.08 | 6.25 | 7.01 | - | - | - | - |
| Gbar_D03_365221 | T/C | 0.08 | - | 6.32 | - | - | - | - | Gbar_D03_413379 | T/C | 0.08 | - | 6.26 | - | - | - | - |
| Gbar_D03_366012 | G/A | 0.08 | - | 6.15 | - | - | - | - | Gbar_D03_413952 | C/T | 0.07 | - | 6.18 | - | - | - | - |
| Gbar_D03_366106 | G/A | 0.08 | 6.22 | 6.70 | - | - | - | - | Gbar_D03_414289 | T/A | 0.08 | 6.30 | 6.75 | - | - | - | - |
| Gbar_D03_366280 | G/C | 0.08 | - | 6.45 | - | - | - | - | Gbar_D03_414431 | T/C | 0.08 | - | 6.07 | - | - | - | - |
| Gbar_D03_367761 | G/A | 0.07 | - | 6.27 | - | - | - | - | Gbar_D03_416021 | G/A | 0.07 | 6.23 | 6.54 | - | - | - | - |
| Gbar_D03_367828 | T/C | 0.08 | 6.68 | 7.26 | - | - | - | - | Gbar_D03_416025 | C/T | 0.07 | 6.20 | 6.66 | - | - | - | - |
| Gbar_D03_367935 | T/C | 0.07 | - | 6.63 | - | - | - | - | Gbar_D03_416026 | A/G | 0.07 | 6.26 | 6.79 | - | - | - | - |
| Gbar_D03_368207 | T/A | 0.08 | 6.22 | 6.82 | - | - | - | - | Gbar_D03_416081 | T/C | 0.07 | - | 6.13 | - | - | - | - |
| Gbar_D03_368537 | G/A | 0.08 | 6.08 | 6.67 | - | - | - | - | Gbar_D03_417385 | G/C | 0.08 | 6.02 | 6.25 | - | - | - | - |
| Gbar_D03_368561 | A/G | 0.08 | 6.33 | 6.87 | - | - | - | - | Gbar_D03_417844 | G/A | 0.07 | 6.17 | 6.67 | - | - | - | - |
| Gbar_D03_368579 | T/C | 0.08 | 6.10 | 6.72 | - | - | - | - | Gbar_D03_418695 | T/C | 0.08 | 6.13 | 6.56 | - | - | - | - |
| Gbar_D03_368619 | G/A | 0.08 | 6.57 | 7.31 | - | - | - | - | Gbar_D03_418726 | A/G | 0.08 | 6.15 | 6.64 | - | - | - | - |
| Gbar_D03_369041 | T/C | 0.08 | 6.09 | 6.96 | - | - | - | - | Gbar_D03_419056 | G/T | 0.08 | 6.17 | 6.84 | - | - | - | - |
| Gbar_D03_369550 | A/G | 0.07 | - | 6.49 | - | - | - | - | Gbar_D03_419441 | C/A | 0.08 | 6.26 | 6.74 | - | - | - | - |
| Gbar_D03_369830 | T/G | 0.08 | 6.54 | 7.23 | - | - | - | - | Gbar_D03_419471 | A/G | 0.07 | - | 6.26 | - | - | - | - |
| Gbar_D03_369956 | T/G | 0.07 | - | 6.15 | - | - | - | - | Gbar_D03_420166 | C/T | 0.08 | - | 6.48 | - | - | - | - |
| Gbar_D03_369979 | A/G | 0.08 | 6.53 | 6.82 | - | - | - | - | Gbar_D03_420689 | C/T | 0.08 | - | 6.05 | - | - | - | - |
| Gbar_D03_370056 | G/T | 0.08 | - | 6.25 | - | - | - | - | Gbar_D03_420773 | G/A | 0.08 | - | 6.31 | - | - | - | - |
| Gbar_D03_370347 | T/C | 0.07 | - | 6.04 | - | - | - | - | Gbar_D03_422415 | G/T | 0.08 | - | 6.39 | - | - | - | - |
| Gbar_D03_370407 | G/A | 0.07 | - | 6.03 | - | - | - | - | Gbar_D03_423244 | T/G | 0.07 | 6.78 | 6.89 | - | - | - | - |
| Gbar_D03_371073 | G/A | 0.08 | 6.26 | 6.76 | - | - | - | - | Gbar_D03_423451 | T/G | 0.07 | - | 6.48 | - | - | - | - |
| Gbar_D03_371111 | C/T | 0.08 | 6.30 | 6.87 | - | - | - | - | Gbar_D03_426537 | G/C | 0.08 | - | 6.08 | - | - | - | - |
| Gbar_D03_371364 | G/A | 0.08 | 6.24 | 6.84 | - | - | - | - | Gbar_D03_426566 | T/C | 0.08 | - | 6.17 | - | - | - | - |
| Gbar_D03_371913 | T/C | 0.08 | 6.27 | 6.75 | - | - | - | - | Gbar_D03_426619 | T/A | 0.08 | - | 6.15 | - | - | - | - |
| Gbar_D03_372101 | G/A | 0.07 | - | 6.03 | - | - | - | - | Gbar_D03_427960 | G/A | 0.07 | 6.59 | 6.69 | - | - | - | - |
| Gbar_D03_372618 | C/T | 0.08 | 6.26 | 6.84 | - | - | - | - | Gbar_D03_428631 | T/A | 0.08 | - | 6.61 | - | - | - | - |
| Gbar_D03_373236 | C/T | 0.08 | 6.04 | 6.68 | - | - | - | - | Gbar_D03_428867 | G/C | 0.08 | - | 6.38 | - | - | - | - |
| Gbar_D03_374672 | A/G | 0.08 | - | 6.14 | - | - | - | - | Gbar_D03_429255 | T/G | 0.07 | - | 6.50 | - | - | - | - |
| Gbar_D03_377608 | T/C | 0.08 | - | 6.57 | - | - | - | - | Gbar_D03_429289 | C/T | 0.07 | 6.32 | 6.68 | - | - | - | - |
| Gbar_D03_377835 | T/C | 0.08 | 6.09 | 6.53 | - | - | - | - | Gbar_D03_429383 | A/G | 0.08 | 6.19 | 6.63 | - | - | - | - |
| Gbar_D03_378142 | G/A | 0.08 | 6.45 | 7.21 | - | - | - | - | Gbar_D03_430264 | A/C | 0.08 | 6.56 | 7.11 | - | - | - | - |
| Gbar_D03_378662 | T/C | 0.07 | - | 6.07 | - | - | - | - | Gbar_D03_430991 | A/G | 0.07 | - | 6.14 | - | - | - | - |
| Gbar_D03_379122 | T/G | 0.07 | - | 6.54 | - | - | - | - | Gbar_D03_431084 | C/G | 0.08 | 6.48 | 6.89 | - | - | - | - |
| Gbar_D03_379635 | C/T | 0.08 | - | 6.11 | - | - | - | - | Gbar_D03_431163 | T/C | 0.08 | - | 6.21 | - | - | - | - |
| Gbar_D03_379739 | G/A | 0.08 | 6.44 | 6.85 | - | - | - | - | Gbar_D03_431380 | C/T | 0.08 | - | 6.57 | - | - | - | - |
| Gbar_D03_380046 | C/T | 0.08 | - | 6.26 | - | - | - | - | Gbar_D03_431389 | T/C | 0.08 | - | 6.07 | - | - | - | - |
| Gbar_D03_380070 | C/A | 0.08 | - | 6.29 | - | - | - | - | Gbar_D03_431689 | T/C | 0.08 | 6.20 | 6.98 | - | - | - | - |
| Gbar_D03_380281 | A/C | 0.08 | 6.03 | 6.67 | - | - | - | - | Gbar_D03_431887 | T/A | 0.07 | - | 6.07 | - | - | - | - |
| Gbar_D03_380335 | T/A | 0.08 | - | 6.29 | - | - | - | - | Gbar_D03_431958 | A/G | 0.07 | - | 6.12 | - | - | - | - |
| Gbar_D03_380949 | C/G | 0.08 | 6.23 | 6.50 | - | - | - | - | Gbar_D03_432032 | T/C | 0.08 | 6.16 | 6.93 | - | - | - | - |
| Gbar_D03_381143 | C/T | 0.07 | - | 6.22 | - | - | - | - | Gbar_D03_432048 | A/G | 0.08 | 6.32 | 6.86 | - | - | - | - |
| Gbar_D03_381277 | G/T | 0.07 | - | 6.10 | - | - | - | - | Gbar_D03_432073 | A/G | 0.08 | 6.01 | 6.71 | - | - | - | - |
| Gbar_D03_381403 | C/A | 0.08 | - | 6.33 | - | - | - | - | Gbar_D03_432198 | C/T | 0.08 | 6.13 | 6.95 | - | - | - | - |
| Gbar_D03_381478 | T/G | 0.08 | 6.27 | 6.82 | - | - | - | - | Gbar_D03_432338 | A/C | 0.08 | - | 6.45 | - | - | - | - |
| Gbar_D03_381665 | T/G | 0.08 | - | 6.14 | - | - | - | - | Gbar_D03_432339 | C/G | 0.08 | - | 6.21 | - | - | - | - |
| Gbar_D03_381736 | A/C | 0.08 | - | 6.07 | - | - | - | - | Gbar_D03_432703 | A/C | 0.08 | 7.11 | 7.94 | - | - | - | - |
| Gbar_D03_382233 | G/A | 0.08 | 6.42 | 6.77 | - | - | - | - | Gbar_D03_433030 | C/T | 0.08 | 6.50 | 6.53 | - | - | - | - |
| Gbar_D03_382256 | G/A | 0.08 | 6.55 | 7.09 | - | - | - | - | Gbar_D03_433567 | A/T | 0.08 | - | 6.32 | - | - | - | - |
| Gbar_D03_382399 | A/G | 0.08 | - | 6.14 | - | - | - | - | Gbar_D03_434058 | T/G | 0.08 | 6.67 | 7.31 | - | - | - | - |
| Gbar_D03_382485 | A/T | 0.08 | 6.33 | 6.73 | - | - | - | - | Gbar_D03_434175 | G/A | 0.08 | 6.11 | 6.21 | - | - | - | - |
| Gbar_D03_382861 | T/C | 0.07 | 6.37 | 6.73 | - | - | - | - | Gbar_D03_434233 | G/A | 0.08 | - | 6.41 | - | - | - | - |
| Gbar_D03_382874 | T/C | 0.07 | 6.54 | 6.91 | - | - | - | - | Gbar_D03_434363 | A/G | 0.08 | 6.13 | 6.18 | - | - | - | - |
| Gbar_D03_383853 | C/T | 0.08 | 6.09 | 6.30 | - | - | - | - | Gbar_D03_434386 | G/A | 0.08 | 6.35 | 6.32 | - | - | - | - |
| Gbar_D03_383926 | G/A | 0.08 | 6.20 | 7.04 | - | - | - | - | Gbar_D03_434425 | A/G | 0.08 | 6.36 | 6.63 | - | - | - | - |
| Gbar_D03_384502 | C/A | 0.08 | - | 6.24 | - | - | - | - | Gbar_D03_434460 | T/C | 0.08 | 6.02 | 6.11 | - | - | - | - |
| Gbar_D03_384657 | A/G | 0.08 | 6.40 | 6.38 | - | - | - | - | Gbar_D03_434671 | C/T | 0.08 | 6.25 | 6.63 | - | - | - | - |
| Gbar_D03_385646 | A/T | 0.08 | - | 6.10 | - | - | - | - | Gbar_D03_434694 | A/T | 0.08 | - | 6.27 | - | - | - | - |
| Gbar_D03_385751 | A/G | 0.08 | 6.66 | 6.80 | - | - | - | - | Gbar_D03_435100 | C/T | 0.07 | 7.17 | 6.86 | - | - | - | - |
| Gbar_D03_385959 | T/G | 0.08 | 6.38 | 7.03 | - | - | - | - | Gbar_D03_435183 | C/T | 0.08 | - | 6.07 | - | - | - | - |
| Gbar_D03_386126 | T/G | 0.08 | - | 6.46 | - | - | - | - | Gbar_D03_435277 | G/C | 0.08 | 6.18 | 6.27 | - | - | - | - |
| Gbar_D03_386546 | T/C | 0.08 | - | 6.71 | - | - | - | - | Gbar_D03_435301 | A/T | 0.08 | 6.30 | 6.46 | - | - | - | - |
| Gbar_D03_388400 | T/A | 0.08 | 6.11 | 6.53 | - | - | - | - | Gbar_D03_435671 | G/T | 0.08 | 6.77 | 6.69 | - | - | - | - |
| Gbar_D03_388455 | T/C | 0.08 | 7.15 | 7.29 | - | - | - | - | Gbar_D03_437353 | G/C | 0.08 | 6.12 | 6.52 | - | - | - | - |
| Gbar_D03_388476 | A/T | 0.08 | - | 6.03 | - | - | - | - | Gbar_D03_437356 | C/T | 0.08 | 6.06 | 6.45 | - | - | - | - |
| Gbar_D03_389413 | T/C | 0.08 | - | 6.06 | - | - | - | - | Gbar_D03_437487 | A/G | 0.08 | 7.07 | 7.72 | - | - | - | - |
| Gbar_D03_389479 | A/G | 0.08 | - | 6.35 | - | - | - | - | Gbar_D03_437532 | C/T | 0.08 | 6.81 | 7.36 | - | - | - | - |
| Gbar_D03_389547 | T/C | 0.07 | - | 6.32 | - | - | - | - | Gbar_D03_437857 | C/A | 0.08 | - | 6.28 | - | - | - | - |
| Gbar_D03_389739 | C/G | 0.08 | - | 6.14 | - | - | - | - | Gbar_D03_437877 | C/T | 0.08 | 6.07 | 6.89 | - | - | - | - |
| Gbar_D03_392094 | T/A | 0.08 | 6.30 | 6.88 | - | - | - | - | Gbar_D03_439082 | C/T | 0.08 | 6.32 | 6.69 | - | - | - | - |
| Gbar_D03_396757 | G/T | 0.08 | 6.01 | 6.31 | - | - | - | - | Gbar_D03_439089 | G/A | 0.08 | 6.29 | 6.71 | - | - | - | - |
| Gbar_D03_396864 | T/C | 0.08 | 6.04 | 6.64 | - | - | - | - | Gbar_D03_439090 | T/A | 0.08 | 6.26 | 6.63 | - | - | - | - |
| Gbar_D03_397540 | T/C | 0.08 | - | 6.10 | - | - | - | - | Gbar_D03_439136 | A/G | 0.08 | 6.09 | 6.48 | - | - | - | - |

**Table S3.** Summary of the total associated SNPs identified through GWAS for *FW* resistance in Sea Island cotton. (Continued)

| SNP | Ref/Alt | MAF | -log (P-value) in different environments | | | | | | SNP | Ref/Alt | MAF | -log (P-value) in different environments | | | | | |
| --- | --- | --- | --- | --- | --- | --- | --- | --- | --- | --- | --- | --- | --- | --- | --- | --- | --- |
|  |  |  | 2015 | 2016 | 2018 | 2019 | Mean | BLUP |  |  |  | 2015 | 2016 | 2018 | 2019 | Mean | BLUP |
| Gbar_D03_439359 | T/C | 0.08 | 6.08 | 6.75 | - | - | - | - | Gbar_D03_471649 | A/G | 0.07 | 6.17 | - | - | - | - | - |
| Gbar_D03_439612 | G/A | 0.08 | 6.63 | 7.12 | - | - | - | - | Gbar_D03_471728 | A/G | 0.08 | 6.48 | 6.89 | - | - | - | - |
| Gbar_D03_439614 | A/G | 0.08 | 6.56 | 7.26 | - | - | - | - | Gbar_D03_471731 | A/G | 0.08 | 6.43 | 6.66 | - | - | - | - |
| Gbar_D03_439744 | A/G | 0.08 | - | 6.79 | - | - | - | - | Gbar_D03_472231 | C/T | 0.08 | 6.24 | 6.81 | - | - | - | - |
| Gbar_D03_439883 | A/G | 0.08 | 6.21 | 6.60 | - | - | - | - | Gbar_D03_473086 | C/T | 0.07 | - | 6.41 | - | - | - | - |
| Gbar_D03_440489 | T/C | 0.07 | 6.60 | 6.48 | - | - | - | - | Gbar_D03_473099 | C/T | 0.08 | 6.23 | 6.67 | - | - | - | - |
| Gbar_D03_441607 | G/T | 0.08 | 6.56 | 7.02 | - | - | - | - | Gbar_D03_474031 | C/T | 0.07 | - | 6.05 | - | - | - | - |
| Gbar_D03_442128 | C/T | 0.08 | 6.03 | 6.16 | - | - | - | - | Gbar_D03_474407 | T/C | 0.08 | - | 6.43 | - | - | - | - |
| Gbar_D03_442279 | G/A | 0.08 | 6.13 | 6.82 | - | - | - | - | Gbar_D03_474411 | C/T | 0.08 | 6.27 | 6.75 | - | - | - | - |
| Gbar_D03_443612 | T/A | 0.07 | - | 6.54 | - | - | - | - | Gbar_D03_474425 | T/C | 0.08 | 6.09 | 6.49 | - | - | - | - |
| Gbar_D03_443813 | G/A | 0.08 | 6.51 | 7.05 | - | - | - | - | Gbar_D03_474518 | T/A | 0.08 | - | 6.59 | - | - | - | - |
| Gbar_D03_443832 | T/C | 0.08 | 6.39 | 6.90 | - | - | - | - | Gbar_D03_474723 | G/T | 0.08 | 6.05 | 6.26 | - | - | - | - |
| Gbar_D03_445097 | C/T | 0.08 | - | 6.39 | - | - | - | - | Gbar_D03_475216 | C/T | 0.08 | 6.53 | 7.28 | - | - | - | - |
| Gbar_D03_446345 | C/T | 0.08 | - | 6.56 | - | - | - | - | Gbar_D03_475398 | G/A | 0.08 | 6.12 | 6.68 | - | - | - | - |
| Gbar_D03_446437 | G/A | 0.08 | - | 6.02 | - | - | - | - | Gbar_D03_475454 | C/A | 0.08 | - | 6.52 | - | - | - | - |
| Gbar_D03_446603 | C/T | 0.08 | 6.17 | 6.47 | - | - | - | - | Gbar_D03_476094 | A/G | 0.07 | - | 6.46 | - | - | - | - |
| Gbar_D03_446682 | C/T | 0.08 | 6.51 | 6.96 | - | - | - | - | Gbar_D03_476170 | G/T | 0.08 | 6.41 | 6.87 | - | - | - | - |
| Gbar_D03_446956 | T/G | 0.08 | 6.26 | 6.83 | - | - | - | - | Gbar_D03_477311 | T/A | 0.08 | 6.33 | 6.65 | - | - | - | - |
| Gbar_D03_447043 | G/C | 0.07 | - | 6.19 | - | - | - | - | Gbar_D03_477449 | T/G | 0.07 | - | 6.40 | - | - | - | - |
| Gbar_D03_447194 | C/T | 0.08 | - | 6.07 | - | - | - | - | Gbar_D03_477691 | C/A | 0.07 | - | 6.09 | - | - | - | - |
| Gbar_D03_447239 | G/A | 0.08 | 6.34 | 7.12 | - | - | - | - | Gbar_D03_478387 | C/T | 0.08 | - | 6.42 | - | - | - | - |
| Gbar_D03_447613 | T/A | 0.08 | - | 6.83 | - | - | - | - | Gbar_D03_478691 | G/T | 0.08 | - | 6.61 | - | - | - | - |
| Gbar_D03_447811 | C/T | 0.07 | - | 6.58 | - | - | - | - | Gbar_D03_478840 | T/C | 0.08 | - | 6.22 | - | - | - | - |
| Gbar_D03_447822 | A/G | 0.07 | - | 6.16 | - | - | - | - | Gbar_D03_478976 | T/C | 0.08 | 6.27 | 7.07 | - | - | - | - |
| Gbar_D03_447988 | T/C | 0.08 | - | 6.36 | - | - | - | - | Gbar_D03_479127 | G/T | 0.07 | - | 6.03 | - | - | - | - |
| Gbar_D03_448346 | C/G | 0.08 | 6.85 | 7.19 | - | - | - | - | Gbar_D03_479959 | C/T | 0.07 | 6.00 | 6.22 | - | - | - | - |
| Gbar_D03_449116 | G/A | 0.07 | - | 6.13 | - | - | - | - | Gbar_D03_479982 | G/A | 0.07 | 6.20 | 6.26 | - | - | - | - |
| Gbar_D03_449644 | T/G | 0.08 | 6.17 | 7.01 | - | - | - | - | Gbar_D03_480243 | T/C | 0.08 | - | 6.19 | - | - | - | - |
| Gbar_D03_449732 | A/T | 0.08 | - | 6.04 | - | - | - | - | Gbar_D03_480382 | A/G | 0.08 | - | 6.45 | - | - | - | - |
| Gbar_D03_449901 | G/T | 0.08 | 6.15 | 6.39 | - | - | - | - | Gbar_D03_480663 | C/T | 0.08 | 6.40 | 6.76 | - | - | - | - |
| Gbar_D03_449954 | A/G | 0.08 | - | 6.56 | - | - | - | - | Gbar_D03_480666 | T/C | 0.08 | 6.14 | 6.52 | - | - | - | - |
| Gbar_D03_450193 | A/G | 0.07 | - | 6.44 | - | - | - | - | Gbar_D03_481380 | G/A | 0.08 | - | 6.56 | - | - | - | - |
| Gbar_D03_450582 | A/G | 0.08 | 6.31 | 6.83 | - | - | - | - | Gbar_D03_481529 | G/A | 0.08 | 6.61 | 6.89 | - | - | - | - |
| Gbar_D03_450697 | A/T | 0.07 | 6.22 | 6.64 | - | - | - | - | Gbar_D03_481659 | C/T | 0.08 | - | 6.19 | - | - | - | - |
| Gbar_D03_451008 | T/C | 0.08 | 6.41 | 6.92 | - | - | - | - | Gbar_D03_481987 | C/T | 0.08 | - | 6.51 | - | - | - | - |
| Gbar_D03_451901 | G/A | 0.08 | 6.13 | 6.97 | - | - | - | - | Gbar_D03_482747 | A/G | 0.08 | 6.61 | 7.01 | - | - | - | - |
| Gbar_D03_452383 | G/A | 0.08 | 6.99 | 7.56 | - | - | - | - | Gbar_D03_482860 | C/T | 0.08 | 6.22 | 6.87 | - | - | - | - |
| Gbar_D03_453697 | C/G | 0.08 | 6.09 | 6.92 | - | - | - | - | Gbar_D03_482946 | G/A | 0.08 | - | 6.63 | - | - | - | - |
| Gbar_D03_453723 | G/T | 0.07 | - | 6.17 | - | - | - | - | Gbar_D03_482995 | C/T | 0.08 | 6.20 | 6.86 | - | - | - | - |
| Gbar_D03_453752 | G/A | 0.07 | 6.54 | 6.75 | - | - | - | - | Gbar_D03_489394 | T/C | 0.08 | 6.44 | 7.21 | - | - | - | - |
| Gbar_D03_453779 | C/T | 0.08 | 6.16 | 6.42 | - | - | - | - | Gbar_D03_489419 | C/T | 0.08 | 6.11 | 6.72 | - | - | - | - |
| Gbar_D03_453781 | T/A | 0.07 | 6.01 | 6.39 | - | - | - | - | Gbar_D03_489609 | A/G | 0.08 | 6.07 | 6.32 | - | - | - | - |
| Gbar_D03_453833 | A/G | 0.08 | 6.39 | 6.79 | - | - | - | - | Gbar_D03_490436 | G/C | 0.08 | - | 6.35 | - | - | - | - |
| Gbar_D03_453867 | T/C | 0.08 | 6.12 | 6.69 | - | - | - | - | Gbar_D03_490683 | G/A | 0.08 | 6.04 | 6.53 | - | - | - | - |
| Gbar_D03_453869 | G/A | 0.08 | 6.33 | 6.90 | - | - | - | - | Gbar_D03_490974 | A/T | 0.08 | - | 6.14 | - | - | - | - |
| Gbar_D03_453971 | C/A | 0.08 | - | 6.52 | - | - | - | - | Gbar_D03_491146 | T/C | 0.08 | - | 6.17 | - | - | - | - |
| Gbar_D03_454071 | G/A | 0.07 | 6.03 | 6.17 | - | - | - | - | Gbar_D03_491506 | C/T | 0.08 | 6.30 | 6.75 | - | - | - | - |
| Gbar_D03_454111 | A/G | 0.08 | - | 6.43 | - | - | - | - | Gbar_D03_491514 | A/T | 0.08 | 6.24 | 6.69 | - | - | - | - |
| Gbar_D03_454158 | T/C | 0.08 | 6.41 | 6.86 | - | - | - | - | Gbar_D03_491757 | C/A | 0.07 | - | 6.29 | - | - | - | - |
| Gbar_D03_454523 | G/A | 0.08 | 6.03 | 6.97 | - | - | - | - | Gbar_D03_491764 | G/A | 0.08 | - | 6.32 | - | - | - | - |
| Gbar_D03_454549 | G/T | 0.08 | - | 6.29 | - | - | - | - | Gbar_D03_492127 | G/A | 0.08 | - | 6.69 | - | - | - | - |
| Gbar_D03_454777 | C/A | 0.08 | - | 6.38 | - | - | - | - | Gbar_D03_492496 | G/A | 0.08 | - | 6.11 | - | - | - | - |
| Gbar_D03_455317 | C/T | 0.08 | - | 6.13 | - | - | - | - | Gbar_D03_492515 | T/C | 0.08 | - | 6.06 | - | - | - | - |
| Gbar_D03_455546 | T/C | 0.09 | - | 6.12 | - | - | - | - | Gbar_D03_492543 | C/T | 0.08 | - | 6.22 | - | - | - | - |
| Gbar_D03_456156 | T/A | 0.08 | - | 6.20 | - | - | - | - | Gbar_D03_493428 | G/T | 0.08 | 6.22 | 6.90 | - | - | - | - |
| Gbar_D03_456615 | T/C | 0.08 | - | 6.53 | - | - | - | - | Gbar_D03_493543 | G/T | 0.07 | - | 6.44 | - | - | - | - |
| Gbar_D03_456764 | A/C | 0.08 | 6.48 | 7.00 | - | - | - | - | Gbar_D03_493656 | G/C | 0.08 | 6.39 | 7.17 | - | - | - | - |
| Gbar_D03_456785 | G/T | 0.07 | - | 6.10 | - | - | - | - | Gbar_D03_493884 | G/A | 0.08 | 6.04 | 6.85 | - | - | - | - |
| Gbar_D03_456854 | T/A | 0.07 | - | 6.27 | - | - | - | - | Gbar_D03_494172 | A/G | 0.08 | 6.35 | 6.92 | - | - | - | - |
| Gbar_D03_457126 | A/G | 0.08 | 6.60 | 7.06 | - | - | - | - | Gbar_D03_494449 | C/A | 0.08 | 6.33 | 6.82 | - | - | - | - |
| Gbar_D03_457147 | A/G | 0.08 | 6.74 | 7.19 | - | - | - | - | Gbar_D03_494851 | T/C | 0.08 | 6.40 | 6.88 | - | - | - | - |
| Gbar_D03_457364 | T/G | 0.08 | - | 6.36 | - | - | - | - | Gbar_D03_495058 | C/G | 0.08 | - | 6.34 | - | - | - | - |
| Gbar_D03_457555 | T/A | 0.08 | 6.11 | 6.58 | - | - | - | - | Gbar_D03_495334 | T/C | 0.08 | 6.51 | 6.85 | - | - | - | - |
| Gbar_D03_457649 | T/G | 0.08 | 6.32 | 7.05 | - | - | - | - | Gbar_D03_496070 | C/T | 0.08 | - | 6.17 | - | - | - | - |
| Gbar_D03_457899 | T/C | 0.08 | - | 6.64 | - | - | - | - | Gbar_D03_496135 | A/G | 0.07 | 6.22 | 6.44 | - | - | - | - |
| Gbar_D03_457905 | C/T | 0.08 | - | 6.23 | - | - | - | - | Gbar_D03_496307 | T/C | 0.08 | - | 6.80 | - | - | - | - |
| Gbar_D03_457921 | T/C | 0.07 | 6.01 | 6.43 | - | - | - | - | Gbar_D03_496437 | G/T | 0.08 | 6.85 | 7.13 | - | - | - | - |
| Gbar_D03_458018 | A/G | 0.08 | 6.68 | 7.24 | - | - | - | - | Gbar_D03_496666 | G/A | 0.08 | 6.33 | 6.62 | - | - | - | - |
| Gbar_D03_458610 | T/G | 0.08 | 6.08 | 6.53 | - | - | - | - | Gbar_D03_496839 | A/T | 0.08 | 6.10 | 6.65 | - | - | - | - |
| Gbar_D03_458775 | G/T | 0.08 | - | 6.27 | - | - | - | - | Gbar_D03_496847 | A/G | 0.07 | - | 6.24 | - | - | - | - |
| Gbar_D03_459639 | G/C | 0.08 | 6.24 | 6.99 | - | - | - | - | Gbar_D03_497738 | A/C | 0.07 | 6.06 | 6.37 | - | - | - | - |
| Gbar_D03_459837 | G/A | 0.08 | - | 6.65 | - | - | - | - | Gbar_D03_497951 | G/A | 0.08 | 6.56 | 7.32 | - | - | - | - |
| Gbar_D03_461284 | A/T | 0.07 | 6.60 | 6.90 | - | - | - | - | Gbar_D03_498371 | T/A | 0.08 | 6.31 | 6.86 | - | - | - | - |
| Gbar_D03_462175 | C/A | 0.08 | 6.21 | 6.70 | - | - | - | - | Gbar_D03_498674 | T/G | 0.07 | - | 6.13 | - | - | - | - |
| Gbar_D03_462259 | A/G | 0.08 | - | 6.68 | - | - | - | - | Gbar_D03_498852 | T/A | 0.07 | - | 6.01 | - | - | - | - |
| Gbar_D03_462396 | T/A | 0.07 | - | 6.37 | - | - | - | - | Gbar_D03_500198 | G/A | 0.08 | - | 6.50 | - | - | - | - |
| Gbar_D03_462575 | G/A | 0.08 | 6.69 | 7.28 | - | - | - | - | Gbar_D03_500515 | A/G | 0.08 | 6.32 | 6.59 | - | - | - | - |
| Gbar_D03_462735 | G/A | 0.08 | 6.46 | 6.88 | - | - | - | - | Gbar_D03_500568 | A/G | 0.08 | - | 6.19 | - | - | - | - |
| Gbar_D03_462750 | C/T | 0.08 | 6.06 | 6.49 | - | - | - | - | Gbar_D03_500815 | C/T | 0.08 | 6.09 | 6.53 | - | - | - | - |
| Gbar_D03_462782 | A/G | 0.08 | 6.19 | 6.83 | - | - | - | - | Gbar_D03_501474 | A/C | 0.08 | 6.46 | 6.91 | - | - | - | - |
| Gbar_D03_462898 | C/T | 0.08 | 6.63 | 7.18 | - | - | - | - | Gbar_D03_501600 | C/T | 0.08 | 6.13 | 6.80 | - | - | - | - |
| Gbar_D03_462912 | C/G | 0.08 | 6.42 | 6.88 | - | - | - | - | Gbar_D03_501668 | C/T | 0.08 | - | 6.21 | - | - | - | - |
| Gbar_D03_463030 | G/A | 0.07 | 7.68 | 7.81 | - | - | - | - | Gbar_D03_501713 | T/C | 0.08 | - | 6.47 | - | - | - | - |
| Gbar_D03_463042 | T/A | 0.07 | 7.39 | 7.72 | - | - | - | - | Gbar_D03_501715 | C/A | 0.08 | - | 6.71 | - | - | - | - |
| Gbar_D03_463069 | T/G | 0.08 | 6.69 | 7.15 | - | - | - | - | Gbar_D03_501946 | T/C | 0.08 | - | 6.23 | - | - | - | - |
| Gbar_D03_463234 | A/G | 0.08 | 6.26 | 6.94 | - | - | - | - | Gbar_D03_502048 | C/T | 0.08 | 6.57 | 6.68 | - | - | - | - |
| Gbar_D03_463271 | C/A | 0.08 | 6.26 | 6.64 | - | - | - | - | Gbar_D03_502197 | T/C | 0.08 | 6.24 | 6.71 | - | - | - | - |
| Gbar_D03_463326 | T/C | 0.08 | 6.53 | 6.99 | - | - | - | - | Gbar_D03_502320 | A/G | 0.07 | - | 6.13 | - | - | - | - |
| Gbar_D03_463364 | A/C | 0.08 | 6.32 | 6.88 | - | - | - | - | Gbar_D03_502354 | G/T | 0.08 | - | 6.22 | - | - | - | - |
| Gbar_D03_463611 | T/C | 0.07 | - | 6.22 | - | - | - | - | Gbar_D03_502804 | A/G | 0.07 | 6.57 | 7.11 | - | - | - | - |
| Gbar_D03_463649 | C/T | 0.08 | - | 6.34 | - | - | - | - | Gbar_D03_502823 | C/T | 0.07 | 6.21 | 6.56 | - | - | - | - |
| Gbar_D03_463691 | A/C | 0.08 | 6.71 | 7.36 | - | - | - | - | Gbar_D03_503008 | A/G | 0.08 | - | 6.23 | - | - | - | - |
| Gbar_D03_463991 | T/C | 0.08 | - | 6.40 | - | - | - | - | Gbar_D03_503017 | T/C | 0.08 | - | 6.19 | - | - | - | - |
| Gbar_D03_464328 | T/G | 0.08 | 6.01 | 6.62 | - | - | - | - | Gbar_D03_503066 | C/T | 0.07 | - | 6.17 | - | - | - | - |
| Gbar_D03_465054 | T/C | 0.07 | - | 6.09 | - | - | - | - | Gbar_D03_503118 | T/C | 0.08 | 6.34 | 6.80 | - | - | - | - |
| Gbar_D03_465332 | C/T | 0.08 | 6.48 | 7.04 | - | - | - | - | Gbar_D03_503165 | T/C | 0.08 | 6.35 | 7.00 | - | - | - | - |
| Gbar_D03_465413 | C/G | 0.08 | - | 6.79 | - | - | - | - | Gbar_D03_503175 | T/A | 0.08 | 6.27 | 6.87 | - | - | - | - |
| Gbar_D03_465477 | G/T | 0.08 | 6.13 | 6.58 | - | - | - | - | Gbar_D03_503307 | C/A | 0.08 | 6.11 | 6.73 | - | - | - | - |
| Gbar_D03_466275 | G/A | 0.08 | 6.09 | 6.71 | - | - | - | - | Gbar_D03_503316 | T/C | 0.08 | - | 6.23 | - | - | - | - |
| Gbar_D03_467959 | A/T | 0.08 | - | 6.64 | - | - | - | - | Gbar_D03_503374 | C/T | 0.08 | 6.14 | 6.43 | - | - | - | - |
| Gbar_D03_468281 | T/G | 0.08 | 6.72 | 7.19 | - | - | - | - | Gbar_D03_503381 | G/T | 0.08 | - | 6.47 | - | - | - | - |
| Gbar_D03_468539 | A/G | 0.07 | 6.17 | 6.30 | - | - | - | - | Gbar_D03_503512 | A/G | 0.08 | - | 6.36 | - | - | - | - |
| Gbar_D03_469034 | G/C | 0.07 | 6.71 | 7.24 | - | - | - | - | Gbar_D03_503636 | T/C | 0.08 | - | 6.32 | - | - | - | - |
| Gbar_D03_469828 | C/T | 0.08 | - | 6.66 | - | - | - | - | Gbar_D03_503662 | T/C | 0.10 | - | 6.34 | - | - | - | - |
| Gbar_D03_470216 | T/C | 0.08 | 6.14 | 6.27 | - | - | - | - | Gbar_D03_503765 | T/C | 0.07 | - | 6.13 | - | - | - | - |
| Gbar_D03_470365 | A/G | 0.08 | - | 6.59 | - | - | - | - | Gbar_D03_503973 | G/A | 0.07 | - | 6.44 | - | - | - | - |
| Gbar_D03_470969 | T/C | 0.08 | - | 6.11 | - | - | - | - | Gbar_D03_504000 | T/G | 0.11 | - | 6.22 | - | - | - | - |
| Gbar_D03_471004 | T/C | 0.08 | - | 6.13 | - | - | - | - | Gbar_D03_504030 | C/T | 0.08 | - | 6.30 | - | - | - | - |
| Gbar_D03_471549 | T/C | 0.08 | 6.16 | 6.29 | - | - | - | - | Gbar_D03_504141 | A/G | 0.08 | 6.11 | 6.73 | - | - | - | - |
| Gbar_D03_471597 | A/G | 0.07 | 6.31 | 6.06 | - | - | - | - | Gbar_D03_504389 | C/T | 0.07 | - | 6.04 | - | - | - | - |

**Table S3.** Summary of the total associated SNPs identified through GWAS for *FW* resistance in Sea Island cotton. (Continued)

| SNP | Ref/Alt | MAF | -log (P-value) in different environments | | | | | | SNP | Ref/Alt | MAF | -log (P-value) in different environments | | | | | |
| --- | --- | --- | --- | --- | --- | --- | --- | --- | --- | --- | --- | --- | --- | --- | --- | --- | --- |
|  |  |  | 2015 | 2016 | 2018 | 2019 | Mean | BLUP |  |  |  | 2015 | 2016 | 2018 | 2019 | Mean | BLUP |
| Gbar_D03_504411 | G/A | 0.08 | 6.22 | 6.77 | - | - | - | - | Gbar_D03_534685 | G/A | 0.07 | - | 6.31 | - | - | - | - |
| Gbar_D03_504566 | T/C | 0.08 | - | 6.17 | - | - | - | - | Gbar_D03_535236 | T/C | 0.07 | - | 6.18 | - | - | - | - |
| Gbar_D03_504575 | G/A | 0.08 | - | 6.13 | - | - | - | - | Gbar_D03_535883 | T/A | 0.08 | 7.48 | 7.80 | - | - | - | - |
| Gbar_D03_504576 | C/T | 0.08 | 6.28 | 6.98 | - | - | - | - | Gbar_D03_536094 | A/G | 0.07 | 6.13 | 6.43 | - | - | - | - |
| Gbar_D03_504805 | T/C | 0.08 | 6.04 | 7.23 | - | - | - | - | Gbar_D03_536096 | G/A | 0.07 | 6.19 | 6.25 | - | - | - | - |
| Gbar_D03_504998 | T/C | 0.08 | - | 6.51 | - | - | - | - | Gbar_D03_536787 | G/A | 0.08 | - | 6.45 | - | - | - | - |
| Gbar_D03_505033 | A/G | 0.08 | 6.41 | 6.81 | - | - | - | - | Gbar_D03_536850 | A/T | 0.08 | 6.29 | 6.99 | - | - | - | - |
| Gbar_D03_505069 | G/A | 0.07 | 6.88 | 6.97 | - | - | - | - | Gbar_D03_536889 | A/T | 0.08 | - | 6.01 | - | - | - | - |
| Gbar_D03_505306 | T/A | 0.07 | - | 6.01 | - | - | - | - | Gbar_D03_537009 | T/C | 0.08 | 6.03 | 6.44 | - | - | - | - |
| Gbar_D03_505828 | C/A | 0.08 | - | 6.02 | - | - | - | - | Gbar_D03_537091 | G/T | 0.08 | 6.36 | 6.87 | - | - | - | - |
| Gbar_D03_505971 | C/T | 0.07 | 6.04 | 6.01 | - | - | - | - | Gbar_D03_537524 | T/C | 0.08 | 6.05 | 6.68 | - | - | - | - |
| Gbar_D03_506076 | T/C | 0.08 | 6.32 | 6.53 | - | - | - | - | Gbar_D03_537961 | C/T | 0.08 | - | 6.07 | - | - | - | - |
| Gbar_D03_506177 | C/T | 0.08 | 6.15 | 6.61 | - | - | - | - | Gbar_D03_538074 | T/C | 0.08 | - | 6.51 | - | - | - | - |
| Gbar_D03_506188 | A/C | 0.08 | 6.12 | 6.76 | - | - | - | - | Gbar_D03_538327 | C/T | 0.08 | 6.16 | 6.58 | - | - | - | - |
| Gbar_D03_506208 | C/G | 0.08 | - | 6.65 | - | - | - | - | Gbar_D03_538413 | C/T | 0.08 | 6.04 | 6.68 | - | - | - | - |
| Gbar_D03_506264 | C/T | 0.07 | 6.71 | 6.92 | - | - | - | - | Gbar_D03_538597 | T/C | 0.08 | - | 6.21 | - | - | - | - |
| Gbar_D03_506275 | A/T | 0.07 | 6.86 | 6.81 | - | - | - | - | Gbar_D03_540155 | A/T | 0.07 | - | 6.26 | - | - | - | - |
| Gbar_D03_506335 | A/G | 0.07 | - | 6.61 | - | - | - | - | Gbar_D03_540171 | A/G | 0.07 | - | 6.07 | - | - | - | - |
| Gbar_D03_506353 | A/T | 0.07 | - | 6.16 | - | - | - | - | Gbar_D03_540330 | A/G | 0.07 | - | 6.07 | - | - | - | - |
| Gbar_D03_506357 | A/G | 0.08 | - | 6.66 | - | - | - | - | Gbar_D03_540437 | T/A | 0.07 | 6.13 | 6.32 | - | - | - | - |
| Gbar_D03_506428 | A/C | 0.07 | 6.46 | 6.59 | - | - | - | - | Gbar_D03_540438 | T/C | 0.07 | 6.25 | 6.51 | - | - | - | - |
| Gbar_D03_506443 | C/T | 0.07 | 6.14 | 6.42 | - | - | - | - | Gbar_D03_541604 | C/T | 0.08 | 6.56 | 7.01 | - | - | - | - |
| Gbar_D03_506471 | G/A | 0.07 | 6.42 | 6.68 | - | - | - | - | Gbar_D03_541634 | T/A | 0.08 | 6.40 | 6.93 | - | - | - | - |
| Gbar_D03_506491 | C/T | 0.08 | 6.26 | 6.91 | - | - | - | - | Gbar_D03_541711 | T/C | 0.07 | 6.44 | 6.95 | - | - | - | - |
| Gbar_D03_506522 | A/G | 0.07 | 6.40 | 6.95 | - | - | - | - | Gbar_D03_541801 | C/T | 0.08 | - | 6.44 | - | - | - | - |
| Gbar_D03_506560 | T/C | 0.07 | - | 6.02 | - | - | - | - | Gbar_D03_541848 | T/A | 0.07 | 6.31 | 6.67 | - | - | - | - |
| Gbar_D03_506634 | G/A | 0.07 | 6.01 | 6.07 | - | - | - | - | Gbar_D03_542054 | C/A | 0.07 | 6.20 | 6.50 | - | - | - | - |
| Gbar_D03_506689 | T/C | 0.07 | - | 6.13 | - | - | - | - | Gbar_D03_542293 | A/G | 0.08 | 6.31 | 7.04 | - | - | - | - |
| Gbar_D03_506792 | A/C | 0.08 | 6.60 | 7.16 | - | - | - | - | Gbar_D03_542441 | A/G | 0.08 | 6.22 | 7.10 | - | - | - | - |
| Gbar_D03_506900 | C/T | 0.07 | 6.10 | 6.41 | - | - | - | - | Gbar_D03_542785 | A/C | 0.08 | 6.09 | 6.64 | - | - | - | - |
| Gbar_D03_507278 | A/G | 0.10 | - | 7.00 | - | - | - | - | Gbar_D03_542796 | A/C | 0.08 | 6.17 | 6.81 | - | - | - | - |
| Gbar_D03_507303 | A/G | 0.08 | 6.76 | 7.16 | - | - | - | - | Gbar_D03_543292 | A/C | 0.08 | 6.82 | 7.50 | - | - | - | - |
| Gbar_D03_507341 | G/A | 0.07 | 6.01 | 6.56 | - | - | - | - | Gbar_D03_543367 | A/T | 0.08 | 6.04 | 6.45 | - | - | - | - |
| Gbar_D03_507512 | T/C | 0.08 | 6.07 | 6.11 | - | - | - | - | Gbar_D03_545173 | C/T | 0.08 | - | 6.68 | - | - | - | - |
| Gbar_D03_507538 | G/A | 0.08 | 6.19 | 6.70 | - | - | - | - | Gbar_D03_545273 | C/T | 0.08 | 6.26 | 6.81 | - | - | - | - |
| Gbar_D03_507583 | T/C | 0.08 | - | 6.79 | - | - | - | - | Gbar_D03_545286 | A/G | 0.08 | 6.11 | 6.80 | - | - | - | - |
| Gbar_D03_507601 | T/C | 0.08 | 6.37 | 6.90 | - | - | - | - | Gbar_D03_545509 | G/A | 0.08 | 6.19 | 6.67 | - | - | - | - |
| Gbar_D03_507695 | G/A | 0.08 | 6.26 | 6.54 | - | - | - | - | Gbar_D03_545512 | A/T | 0.07 | 6.18 | 6.82 | - | - | - | - |
| Gbar_D03_508454 | A/G | 0.08 | - | 6.36 | - | - | - | - | Gbar_D03_546536 | C/T | 0.08 | - | 6.11 | - | - | - | - |
| Gbar_D03_508555 | A/G | 0.08 | 7.19 | 7.56 | - | - | - | - | Gbar_D03_546569 | C/G | 0.08 | - | 6.27 | - | - | - | - |
| Gbar_D03_509339 | T/C | 0.08 | 6.04 | 6.63 | - | - | - | - | Gbar_D03_546637 | T/C | 0.08 | - | 6.10 | - | - | - | - |
| Gbar_D03_509731 | G/A | 0.08 | 6.20 | 7.02 | - | - | - | - | Gbar_D03_546685 | T/C | 0.08 | - | 6.40 | - | - | - | - |
| Gbar_D03_510004 | C/T | 0.07 | 6.74 | 6.98 | - | - | - | - | Gbar_D03_546709 | A/T | 0.08 | - | 6.60 | - | - | - | - |
| Gbar_D03_510058 | C/T | 0.07 | 6.35 | 6.76 | - | - | - | - | Gbar_D03_546783 | A/G | 0.08 | 6.37 | 6.88 | - | - | - | - |
| Gbar_D03_510314 | C/T | 0.08 | 6.03 | 6.71 | - | - | - | - | Gbar_D03_546915 | T/A | 0.08 | 6.50 | 6.82 | - | - | - | - |
| Gbar_D03_510625 | A/G | 0.08 | 6.09 | 6.62 | - | - | - | - | Gbar_D03_546934 | G/T | 0.08 | - | 6.71 | - | - | - | - |
| Gbar_D03_510669 | G/T | 0.08 | 6.51 | 6.97 | - | - | - | - | Gbar_D03_546945 | A/G | 0.07 | - | 6.49 | - | - | - | - |
| Gbar_D03_510852 | A/T | 0.08 | 6.42 | 7.04 | - | - | - | - | Gbar_D03_547140 | A/G | 0.08 | 6.20 | 6.77 | - | - | - | - |
| Gbar_D03_510856 | T/C | 0.07 | - | 6.33 | - | - | - | - | Gbar_D03_547507 | T/C | 0.08 | - | 6.01 | - | - | - | - |
| Gbar_D03_510923 | T/G | 0.07 | 6.73 | 6.91 | - | - | - | - | Gbar_D03_547677 | A/G | 0.08 | - | 6.25 | - | - | - | - |
| Gbar_D03_511182 | A/G | 0.08 | 6.38 | 6.93 | - | - | - | - | Gbar_D03_547888 | T/C | 0.08 | - | 6.15 | - | - | - | - |
| Gbar_D03_512403 | T/A | 0.08 | - | 6.43 | - | - | - | - | Gbar_D03_547906 | C/T | 0.08 | - | 6.22 | - | - | - | - |
| Gbar_D03_512774 | G/A | 0.08 | 6.32 | 6.40 | - | - | - | - | Gbar_D03_548405 | T/C | 0.08 | 6.53 | 7.13 | - | - | - | - |
| Gbar_D03_516561 | A/G | 0.08 | 6.35 | 7.00 | - | - | - | - | Gbar_D03_548415 | C/A | 0.09 | 6.48 | 7.20 | - | - | - | - |
| Gbar_D03_517023 | C/G | 0.08 | - | 6.21 | - | - | - | - | Gbar_D03_548478 | A/T | 0.08 | - | 6.10 | - | - | - | - |
| Gbar_D03_517148 | T/A | 0.08 | - | 6.41 | - | - | - | - | Gbar_D03_548506 | A/G | 0.08 | 6.32 | 6.92 | - | - | - | - |
| Gbar_D03_517899 | T/C | 0.08 | - | 6.57 | - | - | - | - | Gbar_D03_549502 | G/T | 0.08 | - | 6.24 | - | - | - | - |
| Gbar_D03_517918 | T/A | 0.07 | - | 6.13 | - | - | - | - | Gbar_D03_549930 | T/C | 0.08 | - | 6.02 | - | - | - | - |
| Gbar_D03_518070 | G/A | 0.08 | 6.70 | 7.43 | - | - | - | - | Gbar_D03_549974 | A/C | 0.08 | - | 6.50 | - | - | - | - |
| Gbar_D03_519276 | A/G | 0.08 | 6.28 | 6.58 | - | - | - | - | Gbar_D03_550970 | A/G | 0.08 | - | 6.14 | - | - | - | - |
| Gbar_D03_519537 | G/T | 0.08 | 6.13 | 6.69 | - | - | - | - | Gbar_D03_551060 | C/T | 0.08 | 6.25 | 6.69 | - | - | - | - |
| Gbar_D03_519585 | T/G | 0.08 | - | 6.25 | - | - | - | - | Gbar_D03_551093 | G/T | 0.08 | 6.47 | 7.07 | - | - | - | - |
| Gbar_D03_519680 | C/G | 0.07 | 6.16 | 6.70 | - | - | - | - | Gbar_D03_551243 | T/C | 0.08 | - | 6.61 | - | - | - | - |
| Gbar_D03_519697 | T/C | 0.07 | - | 6.42 | - | - | - | - | Gbar_D03_552199 | A/T | 0.08 | 6.83 | 7.42 | - | - | - | - |
| Gbar_D03_519700 | T/C | 0.07 | - | 6.42 | - | - | - | - | Gbar_D03_553317 | A/T | 0.08 | - | 6.92 | - | - | - | - |
| Gbar_D03_519733 | A/G | 0.07 | - | 6.27 | - | - | - | - | Gbar_D03_553400 | G/A | 0.08 | - | 6.49 | - | - | - | - |
| Gbar_D03_519752 | A/G | 0.08 | 6.59 | 6.90 | - | - | - | - | Gbar_D03_553621 | A/G | 0.08 | - | 6.27 | - | - | - | - |
| Gbar_D03_519828 | C/T | 0.08 | 6.27 | 7.00 | - | - | - | - | Gbar_D03_553708 | G/A | 0.08 | - | 6.41 | - | - | - | - |
| Gbar_D03_519838 | A/G | 0.08 | - | 6.61 | - | - | - | - | Gbar_D03_554990 | G/A | 0.07 | 6.06 | 6.63 | - | - | - | - |
| Gbar_D03_519855 | C/T | 0.08 | - | 6.48 | - | - | - | - | Gbar_D03_555179 | T/C | 0.07 | - | 6.47 | - | - | - | - |
| Gbar_D03_519894 | T/C | 0.08 | 6.45 | 7.08 | - | - | - | - | Gbar_D03_555523 | G/A | 0.08 | - | 6.63 | - | - | - | - |
| Gbar_D03_519902 | G/A | 0.08 | 6.51 | 6.99 | - | - | - | - | Gbar_D03_555903 | A/G | 0.07 | 6.25 | 6.65 | - | - | - | - |
| Gbar_D03_519931 | C/T | 0.08 | 6.67 | 7.39 | - | - | - | - | Gbar_D03_556131 | C/T | 0.08 | 6.14 | 6.85 | - | - | - | - |
| Gbar_D03_519978 | T/C | 0.07 | - | 6.21 | - | - | - | - | Gbar_D03_556309 | T/C | 0.08 | 6.30 | 6.89 | - | - | - | - |
| Gbar_D03_520101 | C/T | 0.08 | 6.43 | 6.79 | - | - | - | - | Gbar_D03_556375 | T/C | 0.08 | 6.58 | 6.94 | - | - | - | - |
| Gbar_D03_520350 | C/T | 0.08 | - | 6.57 | - | - | - | - | Gbar_D03_556487 | A/T | 0.08 | - | 7.00 | - | - | - | - |
| Gbar_D03_520368 | C/A | 0.08 | 6.05 | 6.68 | - | - | - | - | Gbar_D03_556612 | A/G | 0.08 | 6.12 | 6.39 | - | - | - | - |
| Gbar_D03_520624 | T/A | 0.08 | 6.18 | 7.21 | - | - | - | - | Gbar_D03_556944 | T/A | 0.07 | - | 6.11 | - | - | - | - |
| Gbar_D03_520660 | C/T | 0.08 | 6.51 | 7.28 | - | - | - | - | Gbar_D03_556956 | G/A | 0.07 | - | 6.05 | - | - | - | - |
| Gbar_D03_521083 | G/A | 0.08 | - | 6.57 | - | - | - | - | Gbar_D03_556972 | G/A | 0.07 | - | 6.00 | - | - | - | - |
| Gbar_D03_521153 | A/C | 0.08 | - | 6.48 | - | - | - | - | Gbar_D03_557193 | G/T | 0.08 | 6.94 | 7.54 | - | - | - | - |
| Gbar_D03_523494 | C/A | 0.07 | - | 6.51 | - | - | - | - | Gbar_D03_557269 | A/G | 0.07 | 6.59 | 6.92 | - | - | - | - |
| Gbar_D03_523860 | C/T | 0.08 | 6.57 | 7.03 | - | - | - | - | Gbar_D03_557938 | G/T | 0.07 | - | 6.13 | - | - | - | - |
| Gbar_D03_523865 | C/T | 0.08 | 6.56 | 7.23 | - | - | - | - | Gbar_D03_558287 | C/T | 0.08 | 6.21 | 6.72 | - | - | - | - |
| Gbar_D03_524047 | C/T | 0.08 | 6.30 | 6.60 | - | - | - | - | Gbar_D03_558772 | A/T | 0.08 | - | 6.26 | - | - | - | - |
| Gbar_D03_524133 | T/C | 0.08 | 6.55 | 7.24 | - | - | - | - | Gbar_D03_560351 | G/C | 0.08 | 6.34 | 7.26 | - | - | - | - |
| Gbar_D03_524400 | T/C | 0.07 | - | 6.20 | - | - | - | - | Gbar_D03_561004 | T/A | 0.08 | 6.39 | 7.04 | - | - | - | - |
| Gbar_D03_525581 | G/A | 0.08 | 6.06 | 6.75 | - | - | - | - | Gbar_D03_561294 | G/A | 0.07 | - | 6.46 | - | - | - | - |
| Gbar_D03_525876 | A/G | 0.08 | - | 6.43 | - | - | - | - | Gbar_D03_561297 | C/T | 0.07 | - | 6.60 | - | - | - | - |
| Gbar_D03_526356 | A/G | 0.08 | - | 6.37 | - | - | - | - | Gbar_D03_561330 | C/T | 0.08 | 6.34 | 6.84 | - | - | - | - |
| Gbar_D03_526457 | T/C | 0.08 | - | 6.52 | - | - | - | - | Gbar_D03_565696 | G/T | 0.08 | - | 6.07 | - | - | - | - |
| Gbar_D03_526472 | A/G | 0.08 | 6.29 | 6.82 | - | - | - | - | Gbar_D03_565718 | T/G | 0.08 | - | 6.53 | - | - | - | - |
| Gbar_D03_527752 | G/A | 0.08 | - | 6.16 | - | - | - | - | Gbar_D03_565872 | T/G | 0.08 | - | 6.95 | - | - | - | - |
| Gbar_D03_527972 | C/T | 0.08 | 6.01 | 6.62 | - | - | - | - | Gbar_D03_566189 | T/A | 0.07 | 6.89 | 7.25 | - | - | - | - |
| Gbar_D03_528020 | C/T | 0.08 | - | 6.74 | - | - | - | - | Gbar_D03_566277 | A/G | 0.07 | 6.47 | 6.54 | - | - | - | - |
| Gbar_D03_528041 | T/C | 0.07 | - | 6.58 | - | - | - | - | Gbar_D03_567217 | G/A | 0.08 | 6.06 | 6.41 | - | - | - | - |
| Gbar_D03_528046 | C/T | 0.07 | - | 6.39 | - | - | - | - | Gbar_D03_568405 | C/T | 0.07 | - | 6.36 | - | - | - | - |
| Gbar_D03_528052 | T/C | 0.07 | - | 6.51 | - | - | - | - | Gbar_D03_568757 | T/C | 0.08 | 6.19 | 6.80 | - | - | - | - |
| Gbar_D03_528129 | A/C | 0.07 | 6.10 | 6.66 | - | - | - | - | Gbar_D03_568803 | C/T | 0.08 | 6.18 | 6.21 | - | - | - | - |
| Gbar_D03_528449 | C/A | 0.08 | - | 6.04 | - | - | - | - | Gbar_D03_569237 | G/A | 0.08 | - | 6.62 | - | - | - | - |
| Gbar_D03_528807 | A/G | 0.08 | 6.01 | 6.56 | - | - | - | - | Gbar_D03_569310 | C/T | 0.08 | 6.40 | 6.82 | - | - | - | - |
| Gbar_D03_530107 | G/A | 0.08 | 6.24 | 6.62 | - | - | - | - | Gbar_D03_570302 | G/A | 0.08 | - | 6.53 | - | - | - | - |
| Gbar_D03_530338 | T/C | 0.08 | - | 6.52 | - | - | - | - | Gbar_D03_570360 | C/T | 0.08 | 6.32 | 6.81 | - | - | - | - |
| Gbar_D03_530744 | A/C | 0.07 | - | 6.14 | - | - | - | - | Gbar_D03_570401 | G/A | 0.08 | - | 6.56 | - | - | - | - |
| Gbar_D03_530851 | A/G | 0.08 | 6.05 | 6.91 | - | - | - | - | Gbar_D03_571081 | A/C | 0.08 | 6.45 | 6.78 | - | - | - | - |
| Gbar_D03_531756 | G/T | 0.08 | 6.32 | 6.97 | - | - | - | - | Gbar_D03_571117 | G/A | 0.07 | 6.13 | 6.49 | - | - | - | - |
| Gbar_D03_532612 | T/G | 0.08 | 6.50 | 7.08 | - | - | - | - | Gbar_D03_571546 | A/G | 0.07 | - | 6.06 | - | - | - | - |
| Gbar_D03_533744 | T/C | 0.08 | 6.21 | 6.71 | - | - | - | - | Gbar_D03_572307 | G/A | 0.08 | - | 6.40 | - | - | - | - |
| Gbar_D03_534225 | G/A | 0.08 | - | 6.07 | - | - | - | - | Gbar_D03_572820 | G/A | 0.08 | - | 6.31 | - | - | - | - |
| Gbar_D03_534462 | G/C | 0.08 | 6.02 | 6.90 | - | - | - | - | Gbar_D03_572846 | T/A | 0.08 | - | 6.37 | - | - | - | - |

**Table S3.** Summary of the total associated SNPs identified through GWAS for *FW* resistance in Sea Island cotton. (Continued)

| SNP | Ref/Alt | MAF | -log (P-value) in different environments | | | | | | SNP | Ref/Alt | MAF | -log (P-value) in different environments | | | | | |
| --- | --- | --- | --- | --- | --- | --- | --- | --- | --- | --- | --- | --- | --- | --- | --- | --- | --- |
|  |  |  | 2015 | 2016 | 2018 | 2019 | Mean | BLUP |  |  |  | 2015 | 2016 | 2018 | 2019 | Mean | BLUP |
| Gbar_D03_574251 | G/A | 0.08 | - | 6.72 | - | - | - | - | Gbar_D03_612444 | T/C | 0.08 | 6.17 | 6.80 | - | - | - | - |
| Gbar_D03_574319 | T/C | 0.08 | 6.16 | 6.88 | - | - | - | - | Gbar_D03_612619 | A/G | 0.08 | 6.08 | 6.74 | - | - | - | - |
| Gbar_D03_574349 | G/A | 0.08 | - | 6.68 | - | - | - | - | Gbar_D03_612946 | A/T | 0.08 | 7.05 | 7.54 | - | - | - | - |
| Gbar_D03_574485 | T/A | 0.08 | 6.59 | 7.07 | - | - | - | - | Gbar_D03_613046 | A/G | 0.08 | 6.75 | 7.09 | - | - | - | - |
| Gbar_D03_574526 | A/G | 0.08 | 6.34 | 6.77 | - | - | - | - | Gbar_D03_613067 | T/C | 0.08 | 6.52 | 6.93 | - | - | - | - |
| Gbar_D03_575215 | T/C | 0.07 | - | 6.05 | - | - | - | - | Gbar_D03_613631 | C/A | 0.08 | 7.08 | 6.90 | - | - | - | - |
| Gbar_D03_576234 | G/A | 0.08 | 6.13 | 6.67 | - | - | - | - | Gbar_D03_613813 | A/C | 0.08 | - | 6.14 | - | - | - | - |
| Gbar_D03_576262 | C/T | 0.08 | 6.19 | 6.74 | - | - | - | - | Gbar_D03_613955 | A/G | 0.08 | 6.34 | 6.89 | - | - | - | - |
| Gbar_D03_577309 | A/G | 0.08 | 6.25 | 6.70 | - | - | - | - | Gbar_D03_614340 | G/A | 0.08 | - | 6.43 | - | - | - | - |
| Gbar_D03_577764 | G/A | 0.07 | - | 6.21 | - | - | - | - | Gbar_D03_614536 | T/C | 0.08 | 6.22 | 6.81 | - | - | - | - |
| Gbar_D03_580128 | T/C | 0.08 | - | 6.34 | - | - | - | - | Gbar_D03_614896 | T/C | 0.08 | 7.00 | 7.54 | - | - | - | - |
| Gbar_D03_580269 | A/G | 0.08 | 6.45 | 7.01 | - | - | - | - | Gbar_D03_614953 | T/C | 0.08 | 7.00 | 7.30 | - | - | - | - |
| Gbar_D03_580780 | T/C | 0.08 | - | 6.47 | - | - | - | - | Gbar_D03_615142 | G/C | 0.08 | - | 6.46 | - | - | - | - |
| Gbar_D03_580781 | G/A | 0.07 | 6.02 | 6.56 | - | - | - | - | Gbar_D03_615180 | A/G | 0.08 | 6.77 | 7.30 | - | - | - | - |
| Gbar_D03_581198 | T/G | 0.07 | - | 6.44 | - | - | - | - | Gbar_D03_615713 | T/A | 0.08 | 6.61 | 6.98 | - | - | - | - |
| Gbar_D03_581868 | C/G | 0.08 | - | 6.50 | - | - | - | - | Gbar_D03_615776 | T/C | 0.08 | 6.88 | 7.07 | - | - | - | - |
| Gbar_D03_582226 | T/C | 0.08 | 6.37 | 6.93 | - | - | - | - | Gbar_D03_615790 | A/T | 0.08 | 7.04 | 7.30 | - | - | - | - |
| Gbar_D03_582312 | C/T | 0.07 | - | 6.19 | - | - | - | - | Gbar_D03_616070 | G/A | 0.08 | 6.65 | 7.00 | - | - | - | - |
| Gbar_D03_582513 | A/T | 0.08 | 6.50 | 7.13 | - | - | - | - | Gbar_D03_616321 | T/C | 0.09 | 6.29 | 6.78 | - | - | - | - |
| Gbar_D03_583841 | A/G | 0.08 | 6.06 | 6.60 | - | - | - | - | Gbar_D03_616638 | T/C | 0.08 | 6.33 | 7.04 | - | - | - | - |
| Gbar_D03_586180 | G/T | 0.08 | - | 6.25 | - | - | - | - | Gbar_D03_617081 | G/A | 0.08 | 6.60 | 7.38 | - | - | - | - |
| Gbar_D03_586207 | A/G | 0.07 | - | 6.33 | - | - | - | - | Gbar_D03_617139 | C/T | 0.08 | 7.18 | 7.59 | - | - | - | - |
| Gbar_D03_586239 | A/G | 0.08 | 6.17 | 6.60 | - | - | - | - | Gbar_D03_617385 | G/A | 0.08 | - | 6.20 | - | - | - | - |
| Gbar_D03_586240 | C/T | 0.08 | - | 6.27 | - | - | - | - | Gbar_D03_618792 | G/A | 0.08 | - | 6.12 | - | - | - | - |
| Gbar_D03_586718 | T/A | 0.08 | - | 6.60 | - | - | - | - | Gbar_D03_618859 | A/C | 0.08 | - | 6.14 | - | - | - | - |
| Gbar_D03_587654 | C/T | 0.08 | - | 6.64 | - | - | - | - | Gbar_D03_618863 | A/T | 0.08 | - | 6.20 | - | - | - | - |
| Gbar_D03_587662 | G/A | 0.08 | 6.14 | 6.50 | - | - | - | - | Gbar_D03_619355 | G/T | 0.08 | - | 6.16 | - | - | - | - |
| Gbar_D03_587674 | G/A | 0.08 | - | 6.24 | - | - | - | - | Gbar_D03_619689 | G/A | 0.08 | 6.22 | 6.56 | - | - | - | - |
| Gbar_D03_587701 | A/T | 0.08 | - | 6.33 | - | - | - | - | Gbar_D03_619731 | C/G | 0.08 | 6.10 | 6.60 | - | - | - | - |
| Gbar_D03_587907 | T/A | 0.07 | 6.09 | 6.32 | - | - | - | - | Gbar_D03_620292 | T/C | 0.08 | 6.96 | 7.42 | - | - | - | - |
| Gbar_D03_587910 | C/T | 0.07 | 6.29 | 6.53 | - | - | - | - | Gbar_D03_620407 | T/C | 0.08 | 6.04 | 6.40 | - | - | - | - |
| Gbar_D03_588355 | G/A | 0.07 | 6.31 | 6.69 | - | - | - | - | Gbar_D03_620424 | A/C | 0.08 | 6.15 | 6.39 | - | - | - | - |
| Gbar_D03_588368 | T/A | 0.08 | 6.25 | 6.57 | - | - | - | - | Gbar_D03_620702 | G/C | 0.08 | 6.10 | 6.98 | - | - | - | - |
| Gbar_D03_588540 | G/A | 0.08 | 6.11 | 6.61 | - | - | - | - | Gbar_D03_621175 | G/T | 0.07 | 7.16 | 6.80 | - | - | - | - |
| Gbar_D03_588734 | A/G | 0.08 | 6.27 | 7.19 | - | - | - | - | Gbar_D03_621182 | A/T | 0.07 | 7.07 | 6.75 | - | - | - | - |
| Gbar_D03_589494 | T/C | 0.08 | 6.30 | 7.00 | - | - | - | - | Gbar_D03_621353 | T/G | 0.08 | 6.27 | 6.57 | - | - | - | - |
| Gbar_D03_589584 | A/G | 0.08 | - | 6.03 | - | - | - | - | Gbar_D03_621810 | G/A | 0.08 | 7.28 | 7.54 | - | - | - | - |
| Gbar_D03_589606 | C/T | 0.08 | 6.09 | 6.66 | - | - | - | - | Gbar_D03_621871 | A/T | 0.08 | 7.09 | 7.30 | - | - | - | - |
| Gbar_D03_589777 | G/A | 0.08 | 6.55 | 6.73 | - | - | - | - | Gbar_D03_621993 | C/T | 0.08 | 6.12 | 6.25 | - | - | - | - |
| Gbar_D03_591495 | G/A | 0.08 | 6.14 | 6.77 | - | - | - | - | Gbar_D03_622680 | A/G | 0.08 | 6.50 | 6.92 | - | - | - | - |
| Gbar_D03_591529 | C/T | 0.08 | 6.46 | 7.05 | - | - | - | - | Gbar_D03_622721 | G/A | 0.08 | 6.17 | 6.20 | - | - | - | - |
| Gbar_D03_591662 | G/C | 0.08 | 6.03 | 6.23 | - | - | - | - | Gbar_D03_622755 | C/T | 0.08 | 6.80 | 6.99 | - | - | - | - |
| Gbar_D03_591765 | A/G | 0.08 | 6.09 | 6.44 | - | - | - | - | Gbar_D03_622781 | C/G | 0.08 | 6.31 | 6.55 | - | - | - | - |
| Gbar_D03_592967 | A/C | 0.08 | - | 6.21 | - | - | - | - | Gbar_D03_624152 | G/A | 0.08 | 6.72 | 7.10 | - | - | - | - |
| Gbar_D03_593143 | A/T | 0.08 | 6.35 | 6.88 | - | - | - | - | Gbar_D03_624169 | T/C | 0.08 | - | 6.07 | - | - | - | - |
| Gbar_D03_593281 | A/T | 0.08 | 6.05 | 6.61 | - | - | - | - | Gbar_D03_624216 | G/C | 0.08 | 6.18 | 6.81 | - | - | - | - |
| Gbar_D03_593846 | C/T | 0.08 | - | 6.70 | - | - | - | - | Gbar_D03_624387 | A/G | 0.08 | 6.32 | 6.96 | - | - | - | - |
| Gbar_D03_593978 | A/T | 0.07 | - | 6.12 | - | - | - | - | Gbar_D03_625037 | C/G | 0.08 | 6.54 | 7.25 | - | - | - | - |
| Gbar_D03_593983 | C/T | 0.07 | - | 6.52 | - | - | - | - | Gbar_D03_625152 | C/T | 0.08 | 6.40 | 6.99 | - | - | - | - |
| Gbar_D03_594208 | G/A | 0.08 | - | 6.11 | - | - | - | - | Gbar_D03_626683 | A/G | 0.07 | 6.60 | 6.68 | - | - | - | - |
| Gbar_D03_594215 | C/T | 0.08 | - | 6.04 | - | - | - | - | Gbar_D03_626688 | G/C | 0.07 | 6.41 | 6.62 | - | - | - | - |
| Gbar_D03_594218 | A/C | 0.08 | - | 6.37 | - | - | - | - | Gbar_D03_627517 | C/T | 0.08 | 6.69 | 7.19 | - | - | - | - |
| Gbar_D03_594365 | T/G | 0.08 | 6.43 | 7.01 | - | - | - | - | Gbar_D03_628525 | C/T | 0.08 | 6.40 | 6.83 | - | - | - | - |
| Gbar_D03_594571 | G/A | 0.08 | 6.83 | 7.27 | - | - | - | - | Gbar_D03_629115 | G/C | 0.08 | 6.54 | 6.56 | - | - | - | - |
| Gbar_D03_594606 | G/A | 0.08 | 7.03 | 7.27 | - | - | - | - | Gbar_D03_630237 | T/C | 0.08 | 7.08 | 7.05 | - | - | - | - |
| Gbar_D03_595001 | T/C | 0.08 | 6.59 | 6.88 | - | - | - | - | Gbar_D03_630241 | C/T | 0.08 | 7.05 | 6.99 | - | - | - | - |
| Gbar_D03_595135 | C/G | 0.08 | - | 6.14 | - | - | - | - | Gbar_D03_630358 | A/G | 0.08 | 6.24 | 6.58 | - | - | - | - |
| Gbar_D03_595376 | C/G | 0.08 | - | 6.65 | - | - | - | - | Gbar_D03_630448 | A/C | 0.08 | 7.62 | 7.31 | - | - | - | 6.07 |
| Gbar_D03_595385 | A/G | 0.08 | 6.13 | 6.83 | - | - | - | - | Gbar_D03_630508 | A/G | 0.08 | 6.50 | 6.79 | - | - | - | - |
| Gbar_D03_595520 | T/G | 0.08 | 6.70 | 7.62 | - | - | - | - | Gbar_D03_631132 | A/T | 0.08 | 6.44 | 6.75 | - | - | - | - |
| Gbar_D03_595940 | G/T | 0.08 | 6.48 | 6.89 | - | - | - | - | Gbar_D03_631407 | T/C | 0.08 | - | 6.24 | - | - | - | - |
| Gbar_D03_596036 | G/A | 0.08 | 6.68 | 6.99 | - | - | - | - | Gbar_D03_632343 | T/C | 0.08 | 6.38 | 7.15 | - | - | - | - |
| Gbar_D03_597218 | T/C | 0.08 | 6.01 | 6.57 | - | - | - | - | Gbar_D03_632717 | C/T | 0.08 | 6.90 | 6.94 | - | - | - | - |
| Gbar_D03_597459 | C/T | 0.08 | - | 6.28 | - | - | - | - | Gbar_D03_632788 | C/T | 0.08 | 6.78 | 7.30 | - | - | - | - |
| Gbar_D03_597762 | T/C | 0.08 | 6.28 | 6.62 | - | - | - | - | Gbar_D03_633338 | C/G | 0.08 | 6.70 | 7.00 | - | - | - | - |
| Gbar_D03_598106 | G/A | 0.08 | 6.26 | 6.47 | - | - | - | - | Gbar_D03_635525 | A/T | 0.08 | - | 6.16 | - | - | - | - |
| Gbar_D03_598463 | T/C | 0.08 | 6.12 | 7.02 | - | - | - | - | Gbar_D03_635625 | A/G | 0.08 | - | 6.73 | - | - | - | - |
| Gbar_D03_598794 | G/A | 0.08 | 6.61 | 6.93 | - | - | - | - | Gbar_D03_635739 | G/A | 0.08 | 6.48 | 6.98 | - | - | - | - |
| Gbar_D03_599878 | A/C | 0.08 | 6.30 | 6.73 | - | - | - | - | Gbar_D03_636387 | T/C | 0.08 | 7.02 | 7.20 | - | - | - | - |
| Gbar_D03_599920 | G/T | 0.08 | - | 6.08 | - | - | - | - | Gbar_D03_638422 | A/T | 0.08 | 6.97 | 7.43 | - | - | - | - |
| Gbar_D03_600088 | T/C | 0.08 | 6.19 | 6.53 | - | - | - | - | Gbar_D03_638844 | G/C | 0.08 | 6.40 | 6.90 | - | - | - | - |
| Gbar_D03_600309 | A/T | 0.08 | 6.16 | 6.54 | - | - | - | - | Gbar_D03_639346 | A/C | 0.08 | 6.60 | 6.51 | - | - | - | - |
| Gbar_D03_600372 | G/A | 0.08 | 6.78 | 7.31 | - | - | - | - | Gbar_D03_640131 | T/A | 0.08 | 6.25 | 6.56 | - | - | - | - |
| Gbar_D03_600532 | A/C | 0.08 | - | 6.39 | - | - | - | - | Gbar_D03_640851 | C/T | 0.08 | 6.49 | 6.59 | - | - | - | - |
| Gbar_D03_600948 | G/A | 0.08 | - | 6.07 | - | - | - | - | Gbar_D03_641404 | T/A | 0.08 | 6.85 | 6.89 | - | - | - | - |
| Gbar_D03_601270 | G/A | 0.08 | 7.19 | 7.21 | - | - | - | - | Gbar_D03_641609 | A/G | 0.08 | 6.88 | 7.23 | - | - | - | - |
| Gbar_D03_601371 | C/G | 0.08 | 6.24 | 6.59 | - | - | - | - | Gbar_D03_641827 | T/C | 0.08 | 6.67 | 7.21 | - | - | - | 6.06 |
| Gbar_D03_601400 | G/A | 0.08 | 6.65 | 6.91 | - | - | - | - | Gbar_D03_641868 | A/C | 0.08 | 6.39 | 7.01 | - | - | - | - |
| Gbar_D03_601850 | T/C | 0.07 | - | 6.06 | - | - | - | - | Gbar_D03_642084 | G/A | 0.08 | 6.52 | 6.79 | - | - | - | - |
| Gbar_D03_602149 | G/T | 0.08 | 6.07 | 6.74 | - | - | - | - | Gbar_D03_642090 | A/G | 0.08 | 6.58 | 6.81 | - | - | - | - |
| Gbar_D03_602267 | C/A | 0.08 | 6.31 | 6.82 | - | - | - | - | Gbar_D03_643791 | A/T | 0.08 | 6.53 | 6.89 | - | - | - | - |
| Gbar_D03_603037 | T/C | 0.08 | 6.31 | 6.53 | - | - | - | - | Gbar_D03_643823 | G/C | 0.07 | 6.56 | 6.72 | - | - | - | - |
| Gbar_D03_603254 | G/A | 0.08 | 6.71 | 7.48 | - | - | - | - | Gbar_D03_643845 | A/G | 0.07 | 6.73 | 6.78 | - | - | - | - |
| Gbar_D03_603337 | T/G | 0.08 | 7.22 | 7.79 | - | - | - | 6.03 | Gbar_D03_644112 | C/T | 0.08 | - | 6.55 | - | - | - | - |
| Gbar_D03_604017 | C/T | 0.08 | 6.12 | 6.15 | - | - | - | - | Gbar_D03_644194 | A/G | 0.08 | 6.49 | 7.19 | - | - | - | - |
| Gbar_D03_604273 | G/A | 0.08 | 6.65 | 7.28 | - | - | - | - | Gbar_D03_644468 | A/T | 0.08 | 6.91 | 7.11 | - | - | - | 6.20 |
| Gbar_D03_604394 | A/T | 0.08 | - | 6.60 | - | - | - | - | Gbar_D03_644565 | A/T | 0.08 | - | 6.34 | - | - | - | - |
| Gbar_D03_604678 | A/G | 0.08 | 6.63 | 7.29 | - | - | - | - | Gbar_D03_644961 | G/A | 0.08 | 6.44 | 7.00 | - | - | - | - |
| Gbar_D03_604883 | G/T | 0.07 | - | 6.09 | - | - | - | - | Gbar_D03_647095 | C/G | 0.08 | - | 6.06 | - | - | - | - |
| Gbar_D03_605420 | A/C | 0.08 | 6.74 | 6.77 | - | - | - | - | Gbar_D03_647296 | C/A | 0.08 | 6.01 | 6.74 | - | - | - | - |
| Gbar_D03_605961 | A/G | 0.08 | - | 6.21 | - | - | - | - | Gbar_D03_648421 | T/C | 0.08 | 6.06 | 7.13 | - | - | - | - |
| Gbar_D03_605977 | C/T | 0.08 | - | 6.03 | - | - | - | - | Gbar_D03_648646 | A/T | 0.08 | 6.29 | 7.19 | - | - | - | - |
| Gbar_D03_606155 | A/G | 0.08 | 6.97 | 7.54 | - | - | - | - | Gbar_D03_648950 | G/A | 0.08 | 6.52 | 6.97 | - | - | - | - |
| Gbar_D03_606191 | T/C | 0.08 | 6.09 | 6.32 | - | - | - | - | Gbar_D03_649503 | A/G | 0.07 | - | 6.09 | - | - | - | - |
| Gbar_D03_606328 | G/C | 0.08 | 6.73 | 6.93 | - | - | - | - | Gbar_D03_650186 | G/T | 0.08 | 6.88 | 7.33 | - | - | - | - |
| Gbar_D03_606418 | C/T | 0.08 | 6.09 | 6.83 | - | - | - | - | Gbar_D03_651878 | C/T | 0.08 | - | 6.49 | - | - | - | - |
| Gbar_D03_607668 | A/G | 0.08 | 6.74 | 6.81 | - | - | - | - | Gbar_D03_651982 | C/T | 0.08 | 6.13 | 6.88 | - | - | - | - |
| Gbar_D03_607819 | G/T | 0.08 | 6.32 | 6.96 | - | - | - | - | Gbar_D03_652175 | T/C | 0.08 | 6.32 | 6.71 | - | - | - | - |
| Gbar_D03_608224 | A/G | 0.08 | 6.95 | 7.46 | - | - | - | - | Gbar_D03_652236 | C/T | 0.08 | 6.33 | 6.86 | - | - | - | - |
| Gbar_D03_609313 | C/A | 0.08 | - | 6.45 | - | - | - | - | Gbar_D03_652617 | C/T | 0.08 | - | 6.57 | - | - | - | - |
| Gbar_D03_609723 | G/A | 0.08 | 6.10 | 6.41 | - | - | - | - | Gbar_D03_652926 | C/T | 0.08 | - | 6.90 | - | - | - | - |
| Gbar_D03_609844 | A/G | 0.08 | 6.35 | 6.84 | - | - | - | - | Gbar_D03_653219 | G/A | 0.08 | - | 6.03 | - | - | - | - |
| Gbar_D03_609904 | C/T | 0.08 | 6.42 | 7.17 | - | - | - | - | Gbar_D03_653227 | G/A | 0.08 | - | 6.27 | - | - | - | - |
| Gbar_D03_610091 | A/T | 0.08 | 6.36 | 6.63 | - | - | - | - | Gbar_D03_653249 | C/T | 0.07 | 6.16 | 6.83 | - | - | - | - |
| Gbar_D03_610595 | T/C | 0.08 | - | 6.49 | - | - | - | - | Gbar_D03_653369 | T/C | 0.08 | 6.98 | 7.24 | - | - | - | - |
| Gbar_D03_611407 | A/T | 0.08 | 6.16 | 6.14 | - | - | - | - | Gbar_D03_653399 | C/A | 0.08 | 6.55 | 6.91 | - | - | - | - |
| Gbar_D03_611581 | C/A | 0.08 | 6.18 | 7.16 | - | - | - | - | Gbar_D03_653541 | G/T | 0.09 | 7.05 | 7.26 | - | - | - | 6.03 |
| Gbar_D03_612296 | G/A | 0.08 | - | 6.15 | - | - | - | - | Gbar_D03_653844 | C/T | 0.08 | 7.34 | 7.56 | - | - | - | - |
| Gbar_D03_612329 | A/G | 0.07 | - | 6.41 | - | - | - | - | Gbar_D03_653863 | G/A | 0.08 | 6.86 | 7.63 | - | - | - | - |
| Gbar_D03_612422 | A/G | 0.08 | 6.09 | 6.74 | - | - | - | - | Gbar_D03_653900 | A/T | 0.08 | 7.52 | 8.00 | - | - | - | 6.00 |

**Table S3.** Summary of the total associated SNPs identified through GWAS for *FW* resistance in Sea Island cotton. (Continued)

| SNP | Ref/Alt | MAF | -log (P-value) in different environments | | | | | | SNP | Ref/Alt | MAF | -log (P-value) in different environments | | | | | |
| --- | --- | --- | --- | --- | --- | --- | --- | --- | --- | --- | --- | --- | --- | --- | --- | --- | --- |
|  |  |  | 2015 | 2016 | 2018 | 2019 | Mean | BLUP |  |  |  | 2015 | 2016 | 2018 | 2019 | Mean | BLUP |
| Gbar_D03_655498 | C/T | 0.08 | - | 6.44 | - | - | - | - | Gbar_D03_694240 | T/A | 0.08 | - | 6.17 | - | - | - | - |
| Gbar_D03_655685 | G/A | 0.08 | - | 6.67 | - | - | - | - | Gbar_D03_694243 | A/T | 0.08 | - | 6.10 | - | - | - | - |
| Gbar_D03_656976 | A/G | 0.08 | 6.78 | 7.40 | - | - | - | - | Gbar_D03_694384 | T/C | 0.08 | 6.96 | 7.20 | - | - | - | - |
| Gbar_D03_656992 | T/C | 0.08 | 6.70 | 7.12 | - | - | - | - | Gbar_D03_694393 | C/T | 0.08 | 6.17 | 6.43 | - | - | - | - |
| Gbar_D03_657002 | A/G | 0.08 | 6.46 | 6.90 | - | - | - | - | Gbar_D03_694535 | T/C | 0.08 | 6.09 | 6.41 | - | - | - | - |
| Gbar_D03_658071 | T/A | 0.08 | 6.46 | 6.76 | - | - | - | - | Gbar_D03_695003 | A/G | 0.08 | 6.43 | 6.32 | - | - | - | - |
| Gbar_D03_658423 | C/G | 0.08 | - | 6.47 | - | - | - | - | Gbar_D03_695028 | G/T | 0.09 | 6.85 | 6.86 | - | - | - | - |
| Gbar_D03_658777 | T/C | 0.08 | 7.00 | 7.56 | - | - | - | - | Gbar_D03_695231 | C/T | 0.08 | - | 6.04 | - | - | - | - |
| Gbar_D03_660586 | A/G | 0.08 | 6.89 | 7.16 | - | - | - | - | Gbar_D03_695381 | A/C | 0.08 | - | 6.29 | - | - | - | - |
| Gbar_D03_660707 | T/C | 0.08 | 6.53 | 6.93 | - | - | - | - | Gbar_D03_697224 | G/C | 0.08 | 6.75 | 7.24 | - | - | - | - |
| Gbar_D03_661386 | C/A | 0.08 | 6.29 | 7.00 | - | - | - | - | Gbar_D03_697559 | A/G | 0.08 | 6.14 | 6.46 | - | - | - | - |
| Gbar_D03_662294 | A/G | 0.08 | 7.33 | 7.65 | - | - | - | - | Gbar_D03_697734 | G/T | 0.08 | 6.65 | 6.83 | - | - | - | - |
| Gbar_D03_662391 | C/A | 0.08 | 6.24 | 6.63 | - | - | - | - | Gbar_D03_699771 | A/G | 0.08 | 6.99 | 7.09 | - | - | - | - |
| Gbar_D03_662434 | A/C | 0.08 | - | 6.40 | - | - | - | - | Gbar_D03_700031 | A/G | 0.08 | 6.08 | 6.89 | - | - | - | - |
| Gbar_D03_662519 | G/A | 0.08 | 6.67 | 6.74 | - | - | - | - | Gbar_D03_700149 | C/T | 0.08 | - | 6.55 | - | - | - | - |
| Gbar_D03_662524 | C/T | 0.08 | 6.46 | 6.61 | - | - | - | - | Gbar_D03_700713 | A/C | 0.08 | 6.61 | 6.87 | - | - | - | - |
| Gbar_D03_662685 | C/T | 0.08 | 6.85 | 7.11 | - | - | - | - | Gbar_D03_700805 | C/G | 0.08 | - | 6.08 | - | - | - | - |
| Gbar_D03_662796 | G/A | 0.08 | 6.68 | 6.69 | - | - | - | - | Gbar_D03_700896 | T/C | 0.07 | 6.14 | 6.65 | - | - | - | - |
| Gbar_D03_662920 | A/T | 0.07 | 6.07 | 6.42 | - | - | - | - | Gbar_D03_701142 | A/T | 0.08 | 6.76 | 7.32 | - | - | - | - |
| Gbar_D03_662986 | T/A | 0.08 | 6.87 | 7.34 | - | - | - | - | Gbar_D03_701637 | A/T | 0.07 | - | 6.10 | - | - | - | - |
| Gbar_D03_663730 | T/C | 0.08 | 6.93 | 7.01 | - | - | - | - | Gbar_D03_702165 | T/G | 0.08 | 6.32 | 6.87 | - | - | - | - |
| Gbar_D03_664095 | G/C | 0.08 | 6.93 | 7.30 | - | - | - | - | Gbar_D03_702396 | G/C | 0.07 | - | 6.38 | - | - | - | - |
| Gbar_D03_664883 | T/G | 0.08 | 6.33 | 6.44 | - | - | - | - | Gbar_D03_702618 | A/T | 0.08 | - | 6.45 | - | - | - | - |
| Gbar_D03_665422 | G/A | 0.08 | 6.84 | 6.85 | - | - | - | - | Gbar_D03_703034 | T/C | 0.08 | - | 6.04 | - | - | - | - |
| Gbar_D03_665945 | C/T | 0.08 | 6.55 | 6.73 | - | - | - | - | Gbar_D03_703054 | C/A | 0.08 | - | 6.32 | - | - | - | - |
| Gbar_D03_666060 | T/C | 0.07 | 6.06 | 6.04 | - | - | - | - | Gbar_D03_703060 | C/T | 0.08 | - | 6.47 | - | - | - | - |
| Gbar_D03_666072 | C/T | 0.07 | 6.34 | 6.01 | - | - | - | - | Gbar_D03_703065 | T/C | 0.08 | - | 6.35 | - | - | - | - |
| Gbar_D03_666082 | C/T | 0.08 | - | 6.02 | - | - | - | - | Gbar_D03_703498 | C/T | 0.08 | 7.30 | 7.58 | - | - | - | - |
| Gbar_D03_666219 | T/C | 0.08 | 6.52 | 6.82 | - | - | - | - | Gbar_D03_703532 | C/T | 0.08 | - | 6.46 | - | - | - | - |
| Gbar_D03_666320 | C/T | 0.08 | 6.89 | 6.94 | - | - | - | - | Gbar_D03_704291 | T/A | 0.07 | 6.17 | 6.77 | - | - | - | - |
| Gbar_D03_666372 | G/A | 0.08 | 6.22 | 7.11 | - | - | - | - | Gbar_D03_706397 | G/T | 0.08 | 6.52 | 7.16 | - | - | - | - |
| Gbar_D03_666912 | T/C | 0.08 | - | 6.10 | - | - | - | - | Gbar_D03_706853 | T/C | 0.08 | 6.27 | 7.02 | - | - | - | - |
| Gbar_D03_667281 | C/A | 0.08 | 6.97 | 7.35 | - | - | - | - | Gbar_D03_706901 | A/G | 0.08 | 6.71 | 7.07 | - | - | - | - |
| Gbar_D03_667390 | T/A | 0.08 | 6.85 | 7.04 | - | - | - | - | Gbar_D03_707326 | T/C | 0.08 | 6.94 | 7.40 | - | - | - | - |
| Gbar_D03_667639 | A/C | 0.08 | 6.23 | 6.97 | - | - | - | - | Gbar_D03_708318 | A/C | 0.08 | 6.66 | 6.94 | - | - | - | - |
| Gbar_D03_669501 | G/A | 0.08 | 7.22 | 7.39 | - | - | - | - | Gbar_D03_709037 | C/G | 0.07 | - | 6.23 | - | - | - | - |
| Gbar_D03_670305 | A/G | 0.08 | 6.14 | 6.60 | - | - | - | - | Gbar_D03_709055 | T/C | 0.08 | 6.25 | 6.45 | - | - | - | - |
| Gbar_D03_670662 | G/T | 0.07 | - | 6.30 | - | - | - | - | Gbar_D03_709387 | T/C | 0.08 | 6.34 | 6.63 | - | - | - | - |
| Gbar_D03_670681 | C/A | 0.08 | 6.66 | 7.07 | - | - | - | - | Gbar_D03_709747 | A/T | 0.08 | 6.70 | 7.08 | - | - | - | - |
| Gbar_D03_670927 | A/G | 0.08 | - | 6.84 | - | - | - | - | Gbar_D03_709909 | C/G | 0.08 | 6.69 | 7.35 | - | - | - | - |
| Gbar_D03_671090 | T/C | 0.07 | 6.52 | 7.07 | - | - | - | - | Gbar_D03_711023 | G/A | 0.08 | 6.08 | 6.09 | - | - | - | - |
| Gbar_D03_671656 | A/T | 0.08 | 6.24 | 6.25 | - | - | - | - | Gbar_D03_711031 | C/T | 0.08 | 6.09 | 6.75 | - | - | - | - |
| Gbar_D03_671919 | C/T | 0.08 | 6.47 | 6.94 | - | - | - | - | Gbar_D03_711056 | A/G | 0.08 | 6.29 | 6.69 | - | - | - | - |
| Gbar_D03_671942 | G/T | 0.07 | 6.37 | 6.76 | - | - | - | - | Gbar_D03_711322 | C/T | 0.08 | 6.19 | 6.57 | - | - | - | - |
| Gbar_D03_672496 | G/A | 0.08 | 6.32 | 6.82 | - | - | - | - | Gbar_D03_711548 | C/T | 0.08 | 6.19 | 6.38 | - | - | - | - |
| Gbar_D03_673021 | C/T | 0.08 | 6.67 | 7.04 | - | - | - | - | Gbar_D03_711741 | T/C | 0.08 | 6.38 | 6.44 | - | - | - | - |
| Gbar_D03_673181 | A/C | 0.08 | 6.42 | 6.84 | - | - | - | - | Gbar_D03_711951 | G/A | 0.08 | 6.16 | 6.64 | - | - | - | - |
| Gbar_D03_673236 | T/G | 0.08 | 6.50 | 6.80 | - | - | - | - | Gbar_D03_711956 | C/T | 0.08 | 6.05 | 6.44 | - | - | - | - |
| Gbar_D03_673341 | C/A | 0.08 | 6.20 | 6.39 | - | - | - | - | Gbar_D03_711964 | T/C | 0.08 | 6.05 | 6.62 | - | - | - | - |
| Gbar_D03_674661 | C/A | 0.08 | - | 6.47 | - | - | - | - | Gbar_D03_712396 | T/C | 0.08 | 6.05 | 6.17 | - | - | - | - |
| Gbar_D03_675228 | T/A | 0.08 | 6.46 | 6.74 | - | - | - | - | Gbar_D03_712651 | A/C | 0.07 | - | 6.42 | - | - | - | - |
| Gbar_D03_676610 | T/A | 0.08 | - | 6.41 | - | - | - | - | Gbar_D03_712797 | G/A | 0.07 | 6.25 | 6.64 | - | - | - | - |
| Gbar_D03_677040 | C/A | 0.08 | - | 6.16 | - | - | - | - | Gbar_D03_713238 | A/G | 0.08 | - | 6.37 | - | - | - | - |
| Gbar_D03_678108 | C/T | 0.09 | - | 6.72 | - | - | - | - | Gbar_D03_713258 | C/T | 0.08 | 6.11 | 6.81 | - | - | - | - |
| Gbar_D03_678703 | T/C | 0.08 | - | 6.30 | - | - | - | - | Gbar_D03_713484 | A/T | 0.08 | 6.94 | 7.22 | - | - | - | - |
| Gbar_D03_680715 | G/A | 0.07 | 6.05 | 6.47 | - | - | - | - | Gbar_D03_713500 | T/C | 0.08 | 6.78 | 7.18 | - | - | - | - |
| Gbar_D03_680754 | A/C | 0.08 | 6.63 | 6.70 | - | - | - | - | Gbar_D03_714281 | A/C | 0.08 | 7.00 | 7.10 | - | - | - | - |
| Gbar_D03_680769 | T/C | 0.08 | 6.66 | 6.83 | - | - | - | - | Gbar_D03_715022 | T/C | 0.08 | - | 6.22 | - | - | - | - |
| Gbar_D03_680781 | A/T | 0.08 | 6.23 | 6.44 | - | - | - | - | Gbar_D03_715304 | A/G | 0.08 | 7.57 | 7.30 | - | - | - | - |
| Gbar_D03_681635 | T/C | 0.08 | - | 6.10 | - | - | - | - | Gbar_D03_715379 | T/A | 0.08 | 6.74 | 6.43 | - | - | - | - |
| Gbar_D03_681810 | C/T | 0.08 | - | 6.07 | - | - | - | - | Gbar_D03_715785 | C/T | 0.08 | 6.27 | 6.81 | - | - | - | - |
| Gbar_D03_681899 | C/A | 0.09 | - | 6.27 | - | - | - | - | Gbar_D03_715832 | A/G | 0.08 | 6.45 | 6.71 | - | - | - | - |
| Gbar_D03_682804 | T/C | 0.08 | 7.02 | 7.33 | - | - | - | - | Gbar_D03_716243 | T/C | 0.08 | 6.81 | 7.13 | - | - | - | - |
| Gbar_D03_682989 | C/A | 0.08 | 6.68 | 7.40 | - | - | - | - | Gbar_D03_716250 | G/A | 0.08 | 7.01 | 7.39 | - | - | - | - |
| Gbar_D03_683619 | C/T | 0.08 | 6.72 | 7.27 | - | - | - | - | Gbar_D03_716447 | A/G | 0.08 | 6.59 | 7.09 | - | - | - | - |
| Gbar_D03_683791 | G/A | 0.07 | - | 6.12 | - | - | - | - | Gbar_D03_716766 | A/G | 0.08 | 6.71 | 7.16 | - | - | - | - |
| Gbar_D03_684447 | G/A | 0.08 | - | 6.15 | - | - | - | - | Gbar_D03_717015 | A/G | 0.08 | 6.14 | 6.67 | - | - | - | - |
| Gbar_D03_684662 | C/A | 0.08 | 6.13 | 6.83 | - | - | - | - | Gbar_D03_717407 | T/C | 0.08 | 7.19 | 7.47 | - | - | - | - |
| Gbar_D03_684675 | T/C | 0.07 | - | 6.37 | - | - | - | - | Gbar_D03_717478 | A/C | 0.08 | - | 6.69 | - | - | - | - |
| Gbar_D03_684681 | C/A | 0.07 | - | 6.27 | - | - | - | - | Gbar_D03_717670 | T/C | 0.08 | - | 6.30 | - | - | - | - |
| Gbar_D03_684963 | C/T | 0.08 | 6.42 | 7.02 | - | - | - | - | Gbar_D03_717695 | G/A | 0.08 | 6.12 | 6.59 | - | - | - | - |
| Gbar_D03_685055 | G/A | 0.08 | 6.43 | 7.14 | - | - | - | - | Gbar_D03_717707 | C/T | 0.08 | - | 6.23 | - | - | - | - |
| Gbar_D03_685350 | G/A | 0.08 | 6.28 | 6.74 | - | - | - | - | Gbar_D03_717742 | G/A | 0.08 | 6.40 | 6.38 | - | - | - | - |
| Gbar_D03_685401 | G/A | 0.08 | 6.14 | 6.77 | - | - | - | - | Gbar_D03_717779 | T/C | 0.09 | 7.42 | 7.52 | - | - | - | - |
| Gbar_D03_686876 | C/T | 0.07 | 6.08 | 6.17 | - | - | - | - | Gbar_D03_717803 | C/G | 0.09 | 6.60 | 7.08 | - | - | - | - |
| Gbar_D03_687839 | T/C | 0.08 | 6.52 | 7.36 | - | - | - | - | Gbar_D03_717823 | C/A | 0.08 | 7.37 | 7.70 | - | - | - | 6.12 |
| Gbar_D03_687857 | G/A | 0.08 | 6.69 | 7.27 | - | - | - | - | Gbar_D03_717913 | A/T | 0.08 | - | 6.10 | - | - | - | - |
| Gbar_D03_687926 | A/T | 0.08 | 6.47 | 6.96 | - | - | - | - | Gbar_D03_718086 | T/C | 0.08 | 6.27 | 6.31 | - | - | - | - |
| Gbar_D03_688264 | G/A | 0.08 | 6.80 | 7.15 | - | - | - | - | Gbar_D03_718221 | C/A | 0.08 | 6.99 | 7.20 | - | - | - | - |
| Gbar_D03_688289 | A/G | 0.08 | 6.50 | 6.99 | - | - | - | - | Gbar_D03_718281 | A/G | 0.08 | 7.80 | 8.24 | - | - | - | 6.17 |
| Gbar_D03_688440 | T/A | 0.08 | 6.64 | 7.08 | - | - | - | - | Gbar_D03_718293 | T/C | 0.08 | 7.64 | 8.05 | - | - | - | 6.09 |
| Gbar_D03_688829 | G/A | 0.08 | 6.29 | 6.61 | - | - | - | - | Gbar_D03_718418 | C/T | 0.08 | 6.05 | 6.44 | - | - | - | - |
| Gbar_D03_688914 | T/A | 0.08 | 6.54 | 6.69 | - | - | - | - | Gbar_D03_718489 | C/T | 0.08 | 6.39 | 6.66 | - | - | - | - |
| Gbar_D03_689331 | G/A | 0.08 | 6.14 | 6.74 | - | - | - | - | Gbar_D03_718632 | T/C | 0.08 | - | 6.02 | - | - | - | - |
| Gbar_D03_689737 | G/C | 0.08 | 6.15 | 6.38 | - | - | - | - | Gbar_D03_719951 | G/C | 0.08 | 6.73 | 6.86 | - | - | - | - |
| Gbar_D03_689840 | T/A | 0.08 | 6.63 | 7.14 | - | - | - | - | Gbar_D03_719969 | G/A | 0.07 | - | 6.39 | - | - | - | - |
| Gbar_D03_690139 | C/T | 0.08 | 6.76 | 7.16 | - | - | - | - | Gbar_D03_721035 | T/C | 0.08 | 6.83 | 7.24 | - | - | - | - |
| Gbar_D03_690265 | T/C | 0.08 | 7.01 | 7.64 | - | - | - | - | Gbar_D03_721318 | T/A | 0.08 | - | 6.13 | - | - | - | - |
| Gbar_D03_690328 | T/G | 0.08 | 6.82 | 7.36 | - | - | - | - | Gbar_D03_721848 | T/A | 0.08 | 6.45 | 6.95 | - | - | - | - |
| Gbar_D03_690397 | T/C | 0.08 | 6.61 | 7.31 | - | - | - | - | Gbar_D03_722369 | G/A | 0.07 | - | 6.05 | - | - | - | - |
| Gbar_D03_690907 | T/C | 0.08 | 6.24 | 6.81 | - | - | - | - | Gbar_D03_722427 | A/G | 0.07 | 6.08 | 6.47 | - | - | - | - |
| Gbar_D03_691097 | T/C | 0.08 | 6.89 | 7.33 | - | - | - | - | Gbar_D03_722630 | C/T | 0.08 | 6.42 | 6.69 | - | - | - | - |
| Gbar_D03_691206 | G/A | 0.08 | 6.76 | 7.21 | - | - | - | - | Gbar_D03_722745 | T/C | 0.08 | 6.85 | 7.09 | - | - | - | - |
| Gbar_D03_691386 | T/C | 0.08 | 6.08 | 6.35 | - | - | - | - | Gbar_D03_723335 | C/T | 0.08 | - | 6.18 | - | - | - | - |
| Gbar_D03_691411 | C/T | 0.08 | 6.68 | 7.12 | - | - | - | - | Gbar_D03_723816 | G/A | 0.08 | 6.87 | 7.74 | - | - | - | 6.07 |
| Gbar_D03_691670 | A/G | 0.08 | 6.84 | 7.52 | - | - | - | - | Gbar_D03_723821 | C/T | 0.08 | 6.68 | 7.43 | - | - | - | - |
| Gbar_D03_691695 | T/A | 0.08 | 6.88 | 7.44 | - | - | - | - | Gbar_D03_724046 | C/T | 0.08 | - | 6.14 | - | - | - | - |
| Gbar_D03_691815 | G/A | 0.08 | 6.61 | 6.80 | - | - | - | - | Gbar_D03_724074 | A/G | 0.08 | - | 6.29 | - | - | - | - |
| Gbar_D03_691924 | C/G | 0.08 | 6.99 | 7.00 | - | - | - | - | Gbar_D03_724108 | C/T | 0.08 | - | 6.21 | - | - | - | - |
| Gbar_D03_692297 | C/T | 0.08 | 6.86 | 6.89 | - | - | - | - | Gbar_D03_724928 | C/T | 0.08 | - | 6.02 | - | - | - | - |
| Gbar_D03_692350 | A/C | 0.08 | 6.80 | 7.40 | - | - | - | - | Gbar_D03_727507 | T/C | 0.07 | - | 6.18 | - | - | - | - |
| Gbar_D03_692682 | G/C | 0.08 | - | 6.18 | - | - | - | - | Gbar_D03_728310 | T/A | 0.08 | 6.39 | 6.70 | - | - | - | - |
| Gbar_D03_693008 | A/T | 0.08 | - | 6.22 | - | - | - | - | Gbar_D03_728441 | A/C | 0.08 | 6.22 | 6.48 | - | - | - | - |
| Gbar_D03_693260 | G/A | 0.08 | - | 6.12 | - | - | - | - | Gbar_D03_728634 | T/A | 0.08 | 6.65 | 7.38 | - | - | - | - |
| Gbar_D03_693585 | A/G | 0.08 | 6.73 | 7.11 | - | - | - | - | Gbar_D03_729235 | T/A | 0.07 | - | 6.26 | - | - | - | - |
| Gbar_D03_693592 | T/C | 0.08 | 6.61 | 7.07 | - | - | - | - | Gbar_D03_729685 | C/A | 0.08 | 7.26 | 7.39 | - | - | - | - |
| Gbar_D03_693634 | A/G | 0.08 | 6.50 | 7.01 | - | - | - | - | Gbar_D03_729694 | T/G | 0.08 | 7.27 | 7.39 | - | - | - | - |
| Gbar_D03_693671 | T/C | 0.08 | 6.63 | 7.10 | - | - | - | - | Gbar_D03_730450 | T/C | 0.08 | 7.14 | 7.39 | - | - | - | - |
| Gbar_D03_693705 | G/A | 0.08 | 6.48 | 7.07 | - | - | - | - | Gbar_D03_730631 | T/A | 0.08 | - | 6.25 | - | - | - | - |
| Gbar_D03_694047 | T/C | 0.08 | - | 6.35 | - | - | - | - | Gbar_D03_730643 | G/A | 0.08 | 6.54 | 7.10 | - | - | - | - |

**Table S3.** Summary of the total associated SNPs identified through GWAS for *FW* resistance in Sea Island cotton. (Continued)

| SNP | Ref/Alt | MAF | -log (P-value) in different environments | | | | | | SNP | Ref/Alt | MAF | -log (P-value) in different environments | | | | | |
| --- | --- | --- | --- | --- | --- | --- | --- | --- | --- | --- | --- | --- | --- | --- | --- | --- | --- |
|  |  |  | 2015 | 2016 | 2018 | 2019 | Mean | BLUP |  |  |  | 2015 | 2016 | 2018 | 2019 | Mean | BLUP |
| Gbar_D03_730898 | G/A | 0.08 | - | 6.29 | - | - | - | - | Gbar_D03_766476 | G/C | 0.08 | 6.49 | 7.07 | - | - | - | - |
| Gbar_D03_731173 | C/T | 0.08 | 6.15 | 6.62 | - | - | - | - | Gbar_D03_766747 | T/A | 0.08 | 6.42 | 6.58 | - | - | - | - |
| Gbar_D03_731174 | A/G | 0.08 | 6.21 | 6.75 | - | - | - | - | Gbar_D03_767268 | A/G | 0.08 | 7.64 | 7.84 | - | - | - | - |
| Gbar_D03_731506 | A/G | 0.08 | 6.80 | 7.17 | - | - | - | - | Gbar_D03_767400 | A/G | 0.08 | 6.57 | 7.07 | - | - | - | - |
| Gbar_D03_731523 | C/T | 0.08 | 6.64 | 6.98 | - | - | - | - | Gbar_D03_767483 | G/A | 0.08 | - | 6.93 | - | - | - | - |
| Gbar_D03_731617 | C/T | 0.08 | - | 6.14 | - | - | - | - | Gbar_D03_768013 | T/C | 0.08 | 6.78 | 7.20 | - | - | - | - |
| Gbar_D03_731669 | C/A | 0.08 | 6.64 | 7.19 | - | - | - | - | Gbar_D03_768014 | C/T | 0.08 | 6.83 | 7.14 | - | - | - | - |
| Gbar_D03_732313 | A/G | 0.08 | 6.80 | 7.28 | - | - | - | - | Gbar_D03_768062 | C/T | 0.08 | 6.19 | 6.90 | - | - | - | - |
| Gbar_D03_732565 | G/A | 0.08 | 6.61 | 7.17 | - | - | - | - | Gbar_D03_768071 | A/G | 0.08 | 6.82 | 7.03 | - | - | - | - |
| Gbar_D03_732765 | T/A | 0.08 | - | 6.25 | - | - | - | - | Gbar_D03_768188 | A/G | 0.08 | - | 6.88 | - | - | - | - |
| Gbar_D03_733818 | T/C | 0.08 | 6.69 | 7.37 | - | - | - | - | Gbar_D03_768195 | G/C | 0.08 | 6.23 | 7.14 | - | - | - | - |
| Gbar_D03_733937 | C/T | 0.08 | 6.88 | 7.16 | - | - | - | - | Gbar_D03_768199 | G/T | 0.08 | 6.23 | 7.14 | - | - | - | - |
| Gbar_D03_733973 | A/G | 0.08 | 7.61 | 7.25 | - | - | - | - | Gbar_D03_768721 | T/C | 0.08 | - | 6.15 | - | - | - | - |
| Gbar_D03_733995 | G/A | 0.08 | - | 6.17 | - | - | - | - | Gbar_D03_768750 | T/C | 0.08 | - | 6.27 | - | - | - | - |
| Gbar_D03_734227 | T/C | 0.08 | 6.70 | 6.93 | - | - | - | - | Gbar_D03_769125 | C/A | 0.09 | 6.79 | 6.92 | - | - | - | - |
| Gbar_D03_736371 | G/T | 0.08 | 6.65 | 7.12 | - | - | - | - | Gbar_D03_769406 | A/T | 0.08 | 6.92 | 7.22 | - | - | - | - |
| Gbar_D03_737104 | T/A | 0.08 | 7.37 | 7.16 | - | - | - | - | Gbar_D03_769597 | A/G | 0.08 | 6.38 | 6.86 | - | - | - | - |
| Gbar_D03_737229 | G/A | 0.08 | 6.94 | 7.05 | - | - | - | - | Gbar_D03_769704 | A/G | 0.08 | 7.08 | 7.04 | - | - | - | - |
| Gbar_D03_737284 | T/C | 0.07 | 6.32 | 6.47 | - | - | - | - | Gbar_D03_770296 | T/C | 0.08 | 6.70 | 6.91 | - | - | - | - |
| Gbar_D03_737374 | G/A | 0.08 | 6.57 | 7.07 | - | - | - | - | Gbar_D03_770656 | G/A | 0.08 | 6.90 | 7.22 | - | - | - | - |
| Gbar_D03_737402 | A/G | 0.08 | 6.43 | 6.67 | - | - | - | - | Gbar_D03_771239 | T/C | 0.08 | 6.50 | 6.82 | - | - | - | - |
| Gbar_D03_738020 | G/A | 0.08 | 6.40 | 6.47 | - | - | - | - | Gbar_D03_771322 | T/C | 0.08 | 6.39 | 6.69 | - | - | - | - |
| Gbar_D03_738692 | G/T | 0.08 | 6.56 | 6.91 | - | - | - | - | Gbar_D03_771391 | A/G | 0.08 | 6.29 | 6.56 | - | - | - | - |
| Gbar_D03_738725 | C/T | 0.08 | 6.72 | 7.21 | - | - | - | - | Gbar_D03_771395 | T/C | 0.08 | 6.68 | 7.03 | - | - | - | - |
| Gbar_D03_739176 | T/C | 0.08 | 6.57 | 6.82 | - | - | - | - | Gbar_D03_771406 | A/G | 0.08 | 6.54 | 6.92 | - | - | - | - |
| Gbar_D03_740008 | G/C | 0.08 | 6.80 | 7.39 | - | - | - | - | Gbar_D03_771825 | G/A | 0.08 | 6.48 | 7.20 | - | - | - | - |
| Gbar_D03_740731 | A/G | 0.08 | - | 6.06 | - | - | - | - | Gbar_D03_772080 | G/T | 0.08 | - | 6.74 | - | - | - | - |
| Gbar_D03_740811 | A/G | 0.08 | 6.80 | 6.97 | - | - | - | - | Gbar_D03_772090 | G/A | 0.08 | 6.50 | 6.92 | - | - | - | 6.01 |
| Gbar_D03_741159 | C/G | 0.08 | 6.98 | 7.37 | - | - | - | - | Gbar_D03_772091 | G/A | 0.08 | 6.85 | 7.20 | - | - | - | 6.25 |
| Gbar_D03_743507 | A/G | 0.08 | - | 6.31 | - | - | - | - | Gbar_D03_772111 | C/T | 0.08 | - | 6.75 | - | - | - | - |
| Gbar_D03_743650 | A/T | 0.08 | - | 6.07 | - | - | - | - | Gbar_D03_772929 | A/G | 0.08 | 6.37 | 6.78 | - | - | - | - |
| Gbar_D03_743755 | T/C | 0.08 | - | 6.49 | - | - | - | - | Gbar_D03_773583 | A/T | 0.08 | - | 6.02 | - | - | - | - |
| Gbar_D03_743815 | G/A | 0.08 | 6.77 | 7.24 | - | - | - | - | Gbar_D03_773717 | G/A | 0.08 | 6.70 | 7.19 | - | - | - | - |
| Gbar_D03_743864 | T/C | 0.08 | 6.53 | 6.92 | - | - | - | - | Gbar_D03_773986 | C/T | 0.08 | - | 6.10 | - | - | - | - |
| Gbar_D03_743905 | T/C | 0.08 | 6.79 | 7.11 | - | - | - | - | Gbar_D03_774109 | G/A | 0.08 | 6.26 | 6.49 | - | - | - | - |
| Gbar_D03_744095 | A/T | 0.08 | 6.50 | 7.39 | - | - | - | - | Gbar_D03_774292 | C/G | 0.08 | 6.09 | 6.33 | - | - | - | - |
| Gbar_D03_744220 | T/G | 0.08 | 6.75 | 7.40 | - | - | - | - | Gbar_D03_774400 | C/T | 0.08 | 6.66 | 7.04 | - | - | - | - |
| Gbar_D03_744715 | A/C | 0.08 | - | 6.25 | - | - | - | - | Gbar_D03_774415 | C/T | 0.08 | 7.44 | 7.58 | - | - | - | - |
| Gbar_D03_744859 | G/C | 0.08 | 6.32 | 7.04 | - | - | - | - | Gbar_D03_774439 | T/C | 0.08 | 7.08 | 7.27 | - | - | - | - |
| Gbar_D03_745085 | G/A | 0.08 | - | 6.03 | - | - | - | - | Gbar_D03_774458 | C/T | 0.08 | 7.04 | 7.09 | - | - | - | - |
| Gbar_D03_745620 | T/G | 0.08 | - | 6.18 | - | - | - | - | Gbar_D03_774516 | A/C | 0.08 | 6.64 | 7.04 | - | - | - | - |
| Gbar_D03_746844 | A/C | 0.08 | 7.12 | 7.34 | - | - | - | - | Gbar_D03_774523 | A/T | 0.08 | 6.50 | 6.84 | - | - | - | - |
| Gbar_D03_746977 | C/G | 0.08 | 7.36 | 7.57 | - | - | - | 6.11 | Gbar_D03_775070 | C/T | 0.08 | 6.61 | 6.98 | - | - | - | - |
| Gbar_D03_747092 | A/T | 0.08 | 6.22 | 6.77 | - | - | - | - | Gbar_D03_775094 | T/A | 0.08 | 6.66 | 6.75 | - | - | - | - |
| Gbar_D03_748718 | G/A | 0.08 | - | 6.31 | - | - | - | - | Gbar_D03_775183 | A/C | 0.08 | 6.73 | 6.98 | - | - | - | - |
| Gbar_D03_748883 | C/A | 0.08 | - | 6.68 | - | - | - | - | Gbar_D03_775461 | T/G | 0.08 | 6.66 | 7.06 | - | - | - | - |
| Gbar_D03_748898 | A/G | 0.08 | - | 6.52 | - | - | - | - | Gbar_D03_775562 | A/G | 0.08 | 6.94 | 6.96 | - | - | - | - |
| Gbar_D03_748937 | C/A | 0.09 | - | 6.01 | - | - | - | - | Gbar_D03_775614 | G/A | 0.08 | 6.78 | 7.32 | - | - | - | - |
| Gbar_D03_749121 | A/T | 0.08 | 6.74 | 6.71 | - | - | - | - | Gbar_D03_776147 | G/A | 0.08 | 6.08 | 6.65 | - | - | - | - |
| Gbar_D03_749122 | T/A | 0.08 | 6.65 | 6.62 | - | - | - | - | Gbar_D03_776247 | A/G | 0.08 | 6.38 | 6.55 | - | - | - | - |
| Gbar_D03_749369 | C/T | 0.08 | 6.47 | 6.83 | - | - | - | - | Gbar_D03_776271 | C/A | 0.08 | 6.36 | 6.81 | - | - | - | - |
| Gbar_D03_749685 | G/A | 0.08 | 6.45 | 7.17 | - | - | - | - | Gbar_D03_776345 | A/G | 0.08 | - | 6.74 | - | - | - | - |
| Gbar_D03_749891 | A/T | 0.08 | 6.21 | 6.43 | - | - | - | - | Gbar_D03_776580 | G/C | 0.08 | 6.44 | 6.88 | - | - | - | - |
| Gbar_D03_750257 | G/A | 0.08 | - | 6.28 | - | - | - | - | Gbar_D03_776590 | A/G | 0.08 | 6.31 | 6.65 | - | - | - | - |
| Gbar_D03_750408 | C/T | 0.08 | 6.72 | 7.16 | - | - | - | - | Gbar_D03_776611 | G/C | 0.08 | 6.47 | 6.81 | - | - | - | - |
| Gbar_D03_750411 | G/A | 0.08 | 6.81 | 7.22 | - | - | - | - | Gbar_D03_776691 | C/A | 0.08 | - | 6.40 | - | - | - | - |
| Gbar_D03_750737 | C/T | 0.08 | 6.90 | 7.32 | - | - | - | - | Gbar_D03_776798 | T/C | 0.08 | 6.47 | 7.10 | - | - | - | - |
| Gbar_D03_750908 | G/T | 0.08 | 6.76 | 6.93 | - | - | - | - | Gbar_D03_776847 | C/G | 0.08 | 6.48 | 6.93 | - | - | - | - |
| Gbar_D03_750962 | G/C | 0.08 | 7.23 | 6.97 | - | - | - | - | Gbar_D03_777093 | C/T | 0.08 | 7.10 | 7.68 | - | - | - | - |
| Gbar_D03_751530 | A/G | 0.09 | 6.24 | - | - | - | - | - | Gbar_D03_777111 | T/G | 0.08 | 6.72 | 7.46 | - | - | - | - |
| Gbar_D03_751937 | C/T | 0.07 | 6.15 | 6.16 | - | - | - | - | Gbar_D03_777135 | T/G | 0.08 | 7.24 | 7.81 | - | - | - | 6.05 |
| Gbar_D03_752091 | T/A | 0.08 | 7.07 | 6.93 | - | - | - | - | Gbar_D03_777146 | A/G | 0.08 | 6.84 | 7.30 | - | - | - | - |
| Gbar_D03_752271 | T/C | 0.08 | 6.37 | 6.75 | - | - | - | - | Gbar_D03_777215 | T/A | 0.08 | 7.22 | 7.55 | - | - | - | - |
| Gbar_D03_752691 | A/C | 0.08 | - | 6.28 | - | - | - | - | Gbar_D03_777669 | C/T | 0.08 | 6.42 | 7.05 | - | - | - | - |
| Gbar_D03_752720 | G/A | 0.09 | - | 6.25 | - | - | - | - | Gbar_D03_777938 | T/G | 0.08 | 6.30 | 6.29 | - | - | - | - |
| Gbar_D03_753046 | C/G | 0.08 | 6.89 | 6.91 | - | - | - | - | Gbar_D03_778036 | T/C | 0.08 | 6.04 | 6.04 | - | - | - | - |
| Gbar_D03_753863 | A/G | 0.08 | 6.23 | 6.77 | - | - | - | - | Gbar_D03_778610 | G/A | 0.08 | 6.05 | 6.21 | - | - | - | - |
| Gbar_D03_754158 | C/T | 0.08 | 7.42 | 8.07 | - | - | - | 6.16 | Gbar_D03_779389 | T/C | 0.08 | 6.75 | 7.12 | - | - | - | - |
| Gbar_D03_754171 | A/G | 0.08 | 6.43 | 6.72 | - | - | - | - | Gbar_D03_779918 | C/T | 0.08 | 6.79 | 7.14 | - | - | - | - |
| Gbar_D03_754191 | A/T | 0.08 | 7.31 | 7.29 | - | - | - | 6.20 | Gbar_D03_780704 | A/G | 0.08 | 7.79 | 8.17 | - | - | - | - |
| Gbar_D03_754844 | A/G | 0.08 | 6.46 | 6.73 | - | - | - | - | Gbar_D03_781370 | C/T | 0.08 | 6.35 | 6.61 | - | - | - | - |
| Gbar_D03_754968 | G/A | 0.08 | - | 6.64 | - | - | - | - | Gbar_D03_786604 | A/T | 0.08 | 6.92 | 7.42 | - | - | - | - |
| Gbar_D03_755431 | T/C | 0.08 | 6.55 | 6.88 | - | - | - | - | Gbar_D03_789050 | C/T | 0.08 | 6.61 | 6.94 | - | - | - | - |
| Gbar_D03_755820 | T/C | 0.08 | 6.75 | 7.18 | - | - | - | - | Gbar_D03_789434 | A/G | 0.08 | 6.59 | 6.75 | - | - | - | - |
| Gbar_D03_756699 | C/A | 0.08 | 6.11 | 6.26 | - | - | - | - | Gbar_D03_789555 | G/A | 0.08 | 6.26 | 6.37 | - | - | - | - |
| Gbar_D03_756879 | A/G | 0.08 | 6.49 | 6.89 | - | - | - | - | Gbar_D03_789594 | A/C | 0.08 | 6.36 | 6.18 | - | - | - | - |
| Gbar_D03_757401 | T/C | 0.08 | 6.30 | 6.58 | - | - | - | - | Gbar_D03_790890 | G/C | 0.08 | 6.55 | 7.23 | - | - | - | - |
| Gbar_D03_757690 | G/A | 0.09 | 6.47 | 7.19 | - | - | - | - | Gbar_D03_791028 | T/G | 0.08 | 6.34 | 6.84 | - | - | - | - |
| Gbar_D03_757878 | C/T | 0.08 | 6.92 | 7.14 | - | - | - | - | Gbar_D03_791052 | T/C | 0.08 | 6.46 | 6.78 | - | - | - | - |
| Gbar_D03_757949 | T/A | 0.08 | 6.75 | 6.92 | - | - | - | - | Gbar_D03_791537 | C/G | 0.08 | - | 6.46 | - | - | - | - |
| Gbar_D03_757977 | T/A | 0.08 | 6.76 | 7.30 | - | - | - | - | Gbar_D03_791742 | T/C | 0.08 | 6.81 | 7.06 | - | - | - | - |
| Gbar_D03_757988 | A/C | 0.08 | 6.53 | 7.09 | - | - | - | - | Gbar_D03_791768 | T/A | 0.08 | 6.56 | 7.07 | - | - | - | - |
| Gbar_D03_758002 | A/G | 0.08 | 6.78 | 7.24 | - | - | - | - | Gbar_D03_792002 | A/G | 0.08 | 6.50 | 6.91 | - | - | - | - |
| Gbar_D03_758220 | T/A | 0.08 | 6.60 | 7.17 | - | - | - | - | Gbar_D03_792243 | G/A | 0.08 | 6.14 | 6.66 | - | - | - | - |
| Gbar_D03_758463 | T/C | 0.08 | 6.04 | 6.37 | - | - | - | - | Gbar_D03_792244 | T/C | 0.08 | 6.44 | 6.73 | - | - | - | - |
| Gbar_D03_759120 | T/C | 0.08 | 6.62 | 6.93 | - | - | - | - | Gbar_D03_792267 | A/G | 0.08 | 6.51 | 6.82 | - | - | - | - |
| Gbar_D03_760090 | T/A | 0.08 | 6.29 | 6.57 | - | - | - | - | Gbar_D03_792353 | A/T | 0.08 | 6.12 | 6.62 | - | - | - | - |
| Gbar_D03_760235 | C/T | 0.08 | - | 6.10 | - | - | - | - | Gbar_D03_792622 | C/T | 0.08 | 6.82 | 7.18 | - | - | - | - |
| Gbar_D03_760482 | T/G | 0.08 | 6.44 | 6.88 | - | - | - | - | Gbar_D03_792702 | T/C | 0.08 | 6.98 | 7.40 | - | - | - | - |
| Gbar_D03_760524 | T/C | 0.08 | 6.40 | 6.87 | - | - | - | - | Gbar_D03_792827 | A/G | 0.08 | - | 6.49 | - | - | - | - |
| Gbar_D03_761174 | G/A | 0.08 | - | 6.25 | - | - | - | - | Gbar_D03_792847 | C/T | 0.08 | 6.35 | 7.17 | - | - | - | - |
| Gbar_D03_761235 | C/A | 0.08 | 6.41 | 6.36 | - | - | - | - | Gbar_D03_793315 | C/T | 0.08 | 6.75 | 6.99 | - | - | - | - |
| Gbar_D03_761532 | T/C | 0.08 | 6.32 | 6.75 | - | - | - | - | Gbar_D03_793333 | C/T | 0.08 | 7.09 | 7.38 | - | - | - | - |
| Gbar_D03_761605 | A/G | 0.08 | 6.82 | 7.00 | - | - | - | - | Gbar_D03_793566 | G/A | 0.08 | 6.89 | 7.11 | - | - | - | - |
| Gbar_D03_761776 | C/T | 0.08 | 6.52 | 7.23 | - | - | - | - | Gbar_D03_793819 | G/A | 0.08 | 6.10 | 6.32 | - | - | - | - |
| Gbar_D03_762044 | G/T | 0.08 | 6.87 | 7.34 | - | - | - | - | Gbar_D03_794186 | T/A | 0.08 | 6.35 | 7.04 | - | - | - | - |
| Gbar_D03_762239 | C/T | 0.08 | 7.22 | 7.34 | - | - | - | - | Gbar_D03_794227 | T/G | 0.08 | 6.66 | 7.16 | - | - | - | - |
| Gbar_D03_762484 | T/C | 0.08 | 6.13 | 6.48 | - | - | - | - | Gbar_D03_794306 | G/A | 0.08 | 6.53 | 7.16 | - | - | - | - |
| Gbar_D03_762656 | G/A | 0.08 | 6.60 | 7.01 | - | - | - | - | Gbar_D03_794426 | G/T | 0.08 | 6.29 | 6.98 | - | - | - | - |
| Gbar_D03_762788 | T/A | 0.08 | 6.28 | 6.90 | - | - | - | - | Gbar_D03_794437 | C/A | 0.08 | 6.55 | 7.04 | - | - | - | - |
| Gbar_D03_764099 | A/G | 0.08 | 6.46 | 6.65 | - | - | - | - | Gbar_D03_794472 | C/T | 0.08 | 6.51 | 6.91 | - | - | - | - |
| Gbar_D03_764500 | G/T | 0.08 | - | 6.07 | - | - | - | - | Gbar_D03_794550 | G/A | 0.08 | 6.31 | 6.94 | - | - | - | - |
| Gbar_D03_764536 | T/A | 0.08 | - | 6.25 | - | - | - | - | Gbar_D03_795285 | C/T | 0.08 | 6.27 | 6.44 | - | - | - | - |
| Gbar_D03_764555 | G/A | 0.08 | - | 6.01 | - | - | - | - | Gbar_D03_795465 | T/A | 0.08 | 6.66 | 6.93 | - | - | - | - |
| Gbar_D03_764818 | G/C | 0.08 | - | 6.02 | - | - | - | - | Gbar_D03_795516 | T/A | 0.08 | 6.90 | 7.24 | - | - | - | - |
| Gbar_D03_764820 | T/C | 0.07 | - | 6.04 | - | - | - | - | Gbar_D03_795532 | G/A | 0.08 | 6.35 | 6.72 | - | - | - | - |
| Gbar_D03_764935 | C/T | 0.08 | 6.43 | 6.40 | - | - | - | - | Gbar_D03_795574 | A/G | 0.08 | 6.72 | 7.21 | - | - | - | - |
| Gbar_D03_765112 | T/C | 0.08 | 6.54 | 6.52 | - | - | - | - | Gbar_D03_795798 | G/A | 0.08 | 6.78 | 7.13 | - | - | - | - |
| Gbar_D03_766362 | C/T | 0.08 | 6.92 | 7.26 | - | - | - | - | Gbar_D03_795872 | T/G | 0.08 | 6.15 | 6.29 | - | - | - | - |
| Gbar_D03_766470 | G/C | 0.08 | 6.95 | 7.38 | - | - | - | - | Gbar_D03_796062 | G/T | 0.08 | 6.28 | 6.45 | - | - | - | - |

**Table S3.** Summary of the total associated SNPs identified through GWAS for *FW* resistance in Sea Island cotton. (Continued)

| SNP | Ref/Alt | MAF | -log (P-value) in different environments | | | | | | SNP | Ref/Alt | MAF | -log (P-value) in different environments | | | | | |
| --- | --- | --- | --- | --- | --- | --- | --- | --- | --- | --- | --- | --- | --- | --- | --- | --- | --- |
|  |  |  | 2015 | 2016 | 2018 | 2019 | Mean | BLUP |  |  |  | 2015 | 2016 | 2018 | 2019 | Mean | BLUP |
| Gbar_D03_796082 | G/A | 0.08 | - | 6.07 | - | - | - | - | Gbar_D03_836246 | T/A | 0.08 | 6.75 | 6.99 | - | - | - | - |
| Gbar_D03_796100 | T/C | 0.08 | 6.25 | 6.42 | - | - | - | - | Gbar_D03_836250 | A/G | 0.08 | 6.78 | 6.95 | - | - | - | - |
| Gbar_D03_796101 | A/C | 0.08 | 6.53 | 6.50 | - | - | - | - | Gbar_D03_837176 | G/A | 0.08 | - | 6.47 | - | - | - | - |
| Gbar_D03_796198 | T/C | 0.08 | 6.03 | 6.30 | - | - | - | - | Gbar_D03_837245 | G/A | 0.08 | 6.64 | 6.90 | - | - | - | - |
| Gbar_D03_796307 | A/G | 0.08 | 6.16 | 6.88 | - | - | - | - | Gbar_D03_837687 | A/T | 0.08 | - | 6.55 | - | - | - | - |
| Gbar_D03_796823 | T/C | 0.08 | 6.40 | 7.01 | - | - | - | - | Gbar_D03_837695 | A/T | 0.08 | - | 6.59 | - | - | - | - |
| Gbar_D03_796888 | T/C | 0.08 | 6.89 | 7.30 | - | - | - | - | Gbar_D03_837933 | T/A | 0.08 | 6.48 | 6.96 | - | - | - | - |
| Gbar_D03_797066 | G/T | 0.08 | 6.81 | 7.31 | - | - | - | - | Gbar_D03_837993 | A/C | 0.08 | 6.67 | 7.26 | - | - | - | - |
| Gbar_D03_797150 | A/T | 0.08 | 6.30 | 6.84 | - | - | - | - | Gbar_D03_838032 | G/C | 0.08 | 6.38 | 6.67 | - | - | - | - |
| Gbar_D03_797573 | T/A | 0.08 | 6.03 | 6.43 | - | - | - | - | Gbar_D03_839089 | A/G | 0.08 | 6.71 | 6.88 | - | - | - | - |
| Gbar_D03_798678 | G/T | 0.08 | - | 6.08 | - | - | - | - | Gbar_D03_839863 | T/C | 0.08 | 6.61 | 7.10 | - | - | - | - |
| Gbar_D03_798962 | C/T | 0.08 | 6.67 | 6.84 | - | - | - | - | Gbar_D03_839876 | T/C | 0.08 | 6.79 | 7.30 | - | - | - | - |
| Gbar_D03_799134 | A/G | 0.08 | 6.63 | 7.07 | - | - | - | - | Gbar_D03_840601 | G/T | 0.08 | - | 6.49 | - | - | - | - |
| Gbar_D03_799247 | C/T | 0.08 | - | 6.25 | - | - | - | - | Gbar_D03_840765 | T/C | 0.08 | 6.24 | 6.64 | - | - | - | - |
| Gbar_D03_799255 | A/T | 0.08 | - | 6.44 | - | - | - | - | Gbar_D03_841312 | C/G | 0.08 | 6.78 | 7.30 | - | - | - | - |
| Gbar_D03_799256 | A/T | 0.08 | - | 6.44 | - | - | - | - | Gbar_D03_841994 | A/C | 0.08 | 6.50 | 6.91 | - | - | - | - |
| Gbar_D03_799319 | T/C | 0.08 | - | 6.12 | - | - | - | - | Gbar_D03_842586 | A/G | 0.08 | 6.63 | 6.96 | - | - | - | - |
| Gbar_D03_799394 | C/A | 0.08 | - | 6.48 | - | - | - | - | Gbar_D03_843572 | A/C | 0.08 | 6.14 | 6.81 | - | - | - | - |
| Gbar_D03_800106 | C/T | 0.08 | 7.09 | 7.43 | - | - | - | - | Gbar_D03_843648 | A/T | 0.08 | 6.39 | 6.80 | - | - | - | - |
| Gbar_D03_800241 | A/G | 0.08 | 6.69 | 7.05 | - | - | - | - | Gbar_D03_843867 | C/T | 0.08 | 6.78 | 7.24 | - | - | - | - |
| Gbar_D03_800458 | C/T | 0.08 | 6.87 | 7.16 | - | - | - | - | Gbar_D03_845453 | A/G | 0.09 | 6.55 | 6.72 | - | - | - | - |
| Gbar_D03_802124 | G/A | 0.08 | 6.64 | 7.06 | - | - | - | - | Gbar_D03_845600 | G/A | 0.09 | 6.31 | 7.04 | - | - | - | - |
| Gbar_D03_802482 | T/C | 0.08 | - | 6.01 | - | - | - | - | Gbar_D03_845689 | A/G | 0.08 | 6.89 | 7.21 | - | - | - | - |
| Gbar_D03_802651 | A/T | 0.08 | - | 6.03 | - | - | - | - | Gbar_D03_845829 | G/A | 0.08 | - | 6.47 | - | - | - | - |
| Gbar_D03_802954 | C/A | 0.08 | 6.85 | 7.49 | - | - | - | - | Gbar_D03_845864 | T/G | 0.08 | 6.28 | 6.85 | - | - | - | - |
| Gbar_D03_802966 | C/T | 0.08 | 6.64 | 7.34 | - | - | - | - | Gbar_D03_845887 | G/A | 0.09 | 6.96 | 7.37 | - | - | - | - |
| Gbar_D03_803002 | A/T | 0.08 | 6.22 | 6.61 | - | - | - | - | Gbar_D03_845918 | C/T | 0.08 | 6.92 | 7.38 | - | - | - | - |
| Gbar_D03_803219 | C/T | 0.08 | 6.90 | 7.36 | - | - | - | - | Gbar_D03_845927 | C/A | 0.08 | 6.92 | 6.95 | - | - | - | - |
| Gbar_D03_803493 | C/T | 0.08 | 6.44 | 6.52 | - | - | - | - | Gbar_D03_845978 | C/T | 0.08 | 6.67 | 6.71 | - | - | - | - |
| Gbar_D03_803800 | C/G | 0.08 | 6.64 | 7.15 | - | - | - | - | Gbar_D03_846044 | C/T | 0.08 | 6.95 | 6.95 | - | - | - | - |
| Gbar_D03_804931 | A/C | 0.08 | 6.34 | 6.93 | - | - | - | - | Gbar_D03_846113 | A/G | 0.08 | 7.68 | 7.97 | - | - | - | - |
| Gbar_D03_804990 | T/C | 0.08 | - | 6.45 | - | - | - | - | Gbar_D03_846244 | C/T | 0.08 | 6.83 | 7.14 | - | - | - | - |
| Gbar_D03_805316 | G/T | 0.08 | - | 6.28 | - | - | - | - | Gbar_D03_846411 | G/A | 0.08 | 6.88 | 7.46 | - | - | - | - |
| Gbar_D03_805693 | A/T | 0.08 | - | 6.09 | - | - | - | - | Gbar_D03_846776 | A/T | 0.08 | - | 6.78 | - | - | - | - |
| Gbar_D03_806228 | G/T | 0.08 | 6.73 | 6.83 | - | - | - | - | Gbar_D03_846928 | T/C | 0.08 | 6.46 | 6.59 | - | - | - | - |
| Gbar_D03_806237 | A/G | 0.08 | 6.75 | 6.86 | - | - | - | - | Gbar_D03_847147 | G/A | 0.08 | 6.54 | 7.18 | - | - | - | - |
| Gbar_D03_806328 | G/C | 0.08 | 6.33 | 7.26 | - | - | - | - | Gbar_D03_847189 | G/A | 0.08 | 6.42 | 6.72 | - | - | - | - |
| Gbar_D03_806361 | A/G | 0.08 | 6.16 | 7.05 | - | - | - | - | Gbar_D03_847202 | A/T | 0.08 | 6.43 | 6.78 | - | - | - | - |
| Gbar_D03_806465 | T/C | 0.08 | - | 6.40 | - | - | - | - | Gbar_D03_847223 | A/G | 0.08 | 6.14 | 6.52 | - | - | - | - |
| Gbar_D03_806529 | A/G | 0.08 | 6.52 | 6.72 | - | - | - | - | Gbar_D03_847355 | T/G | 0.08 | 6.91 | 7.47 | - | - | - | - |
| Gbar_D03_806539 | T/C | 0.08 | - | 6.26 | - | - | - | - | Gbar_D03_847401 | G/A | 0.08 | 6.77 | 6.95 | - | - | - | - |
| Gbar_D03_806589 | T/C | 0.08 | - | 6.10 | - | - | - | - | Gbar_D03_847995 | G/A | 0.08 | 6.02 | 6.12 | - | - | - | - |
| Gbar_D03_807754 | T/A | 0.08 | 7.08 | 7.51 | - | - | - | - | Gbar_D03_848275 | T/C | 0.07 | 6.20 | 6.06 | - | - | - | - |
| Gbar_D03_807990 | C/T | 0.08 | 7.08 | 7.34 | - | - | - | - | Gbar_D03_848436 | T/A | 0.08 | - | 6.07 | - | - | - | - |
| Gbar_D03_808150 | A/C | 0.08 | 6.31 | 6.76 | - | - | - | - | Gbar_D03_848499 | C/T | 0.08 | 6.11 | 6.52 | - | - | - | - |
| Gbar_D03_808265 | C/G | 0.08 | 6.00 | 6.19 | - | - | - | - | Gbar_D03_848643 | A/C | 0.08 | 7.26 | 7.67 | - | - | - | 6.12 |
| Gbar_D03_808550 | T/G | 0.08 | 7.15 | 7.41 | - | - | - | - | Gbar_D03_848661 | C/T | 0.08 | 7.35 | 7.94 | - | - | - | - |
| Gbar_D03_809610 | C/T | 0.08 | 6.73 | 7.07 | - | - | - | - | Gbar_D03_849103 | C/A | 0.08 | - | 6.00 | - | - | - | - |
| Gbar_D03_809907 | T/A | 0.08 | 7.61 | 7.96 | - | - | 6.03 | 6.30 | Gbar_D03_849352 | A/G | 0.08 | 6.26 | 6.63 | - | - | - | - |
| Gbar_D03_809936 | T/C | 0.08 | 7.29 | 7.58 | - | - | - | 6.15 | Gbar_D03_849388 | G/A | 0.08 | 6.44 | 6.67 | - | - | - | - |
| Gbar_D03_810081 | C/T | 0.08 | - | 6.39 | - | - | - | - | Gbar_D03_849439 | A/G | 0.08 | 6.51 | 6.61 | - | - | - | - |
| Gbar_D03_810116 | C/T | 0.08 | 6.30 | 6.55 | - | - | - | - | Gbar_D03_849633 | C/T | 0.08 | 6.11 | 6.33 | - | - | - | - |
| Gbar_D03_810150 | G/A | 0.08 | 6.59 | 6.74 | - | - | - | - | Gbar_D03_850056 | C/A | 0.08 | 6.60 | 7.35 | - | - | - | - |
| Gbar_D03_810613 | A/G | 0.08 | 7.41 | 8.07 | - | - | - | 6.02 | Gbar_D03_850089 | C/G | 0.08 | - | 6.33 | - | - | - | - |
| Gbar_D03_810680 | G/A | 0.07 | 6.26 | 6.57 | - | - | - | - | Gbar_D03_850208 | C/T | 0.08 | 6.93 | 7.38 | - | - | - | - |
| Gbar_D03_811064 | A/T | 0.08 | 6.32 | 6.60 | - | - | - | - | Gbar_D03_850677 | C/A | 0.08 | 6.13 | 6.60 | - | - | - | - |
| Gbar_D03_811336 | T/C | 0.08 | 6.78 | 7.33 | - | - | - | - | Gbar_D03_850678 | T/C | 0.08 | - | 6.51 | - | - | - | - |
| Gbar_D03_811591 | T/C | 0.08 | 6.55 | 7.04 | - | - | - | - | Gbar_D03_851878 | T/C | 0.08 | 6.49 | 7.16 | - | - | - | - |
| Gbar_D03_811659 | C/T | 0.08 | 6.31 | 6.85 | - | - | - | - | Gbar_D03_852191 | C/T | 0.08 | 6.59 | 7.27 | - | - | - | - |
| Gbar_D03_811778 | A/C | 0.08 | 6.91 | 7.19 | - | - | - | - | Gbar_D03_853726 | A/G | 0.08 | - | 6.99 | - | - | - | - |
| Gbar_D03_812251 | C/G | 0.08 | 7.15 | 7.02 | - | - | - | - | Gbar_D03_855664 | G/A | 0.08 | 6.48 | 6.84 | - | - | - | - |
| Gbar_D03_813482 | C/T | 0.08 | 6.98 | 7.39 | - | - | - | - | Gbar_D03_855672 | C/T | 0.08 | - | 6.18 | - | - | - | - |
| Gbar_D03_813572 | T/G | 0.08 | 6.54 | 7.06 | - | - | - | - | Gbar_D03_856654 | C/A | 0.08 | 6.31 | 6.61 | - | - | - | - |
| Gbar_D03_813580 | A/C | 0.07 | 6.37 | 6.74 | - | - | - | - | Gbar_D03_857269 | C/T | 0.08 | 6.51 | 6.83 | - | - | - | - |
| Gbar_D03_813730 | T/A | 0.08 | 6.82 | 7.10 | - | - | - | - | Gbar_D03_857625 | T/C | 0.08 | - | 6.25 | - | - | - | - |
| Gbar_D03_813783 | T/C | 0.08 | 6.49 | 6.78 | - | - | - | - | Gbar_D03_857695 | T/C | 0.08 | - | 6.16 | - | - | - | - |
| Gbar_D03_813832 | A/G | 0.08 | - | 6.28 | - | - | - | - | Gbar_D03_857949 | A/G | 0.08 | 6.33 | 6.64 | - | - | - | - |
| Gbar_D03_814065 | A/T | 0.08 | - | 6.31 | - | - | - | - | Gbar_D03_858238 | T/C | 0.08 | 6.50 | 6.84 | - | - | - | - |
| Gbar_D03_815481 | A/G | 0.08 | 6.29 | 6.92 | - | - | - | - | Gbar_D03_858711 | C/T | 0.08 | 6.67 | 7.06 | - | - | - | - |
| Gbar_D03_815663 | C/T | 0.08 | - | 6.03 | - | - | - | - | Gbar_D03_859170 | G/A | 0.08 | 7.12 | 7.08 | - | - | - | - |
| Gbar_D03_815732 | C/T | 0.08 | 6.43 | 6.96 | - | - | - | - | Gbar_D03_859805 | G/A | 0.08 | - | 6.49 | - | - | - | - |
| Gbar_D03_816340 | G/A | 0.08 | 7.02 | 7.52 | - | - | - | - | Gbar_D03_859825 | C/T | 0.08 | - | 6.95 | - | - | - | - |
| Gbar_D03_817303 | G/A | 0.09 | 6.70 | 7.14 | - | - | - | - | Gbar_D03_860521 | A/G | 0.08 | 6.52 | 6.93 | - | - | - | - |
| Gbar_D03_817476 | G/A | 0.08 | 6.17 | 6.47 | - | - | - | - | Gbar_D03_860559 | G/A | 0.08 | 6.06 | 6.39 | - | - | - | - |
| Gbar_D03_818461 | T/C | 0.08 | 6.67 | 7.22 | - | - | - | - | Gbar_D03_860585 | C/T | 0.08 | 6.28 | 6.78 | - | - | - | - |
| Gbar_D03_818955 | C/A | 0.08 | 6.07 | 6.37 | - | - | - | - | Gbar_D03_861068 | T/C | 0.08 | 6.59 | 6.70 | - | - | - | - |
| Gbar_D03_819316 | C/G | 0.08 | 6.91 | 7.44 | - | - | - | - | Gbar_D03_861112 | A/G | 0.08 | 6.83 | 7.51 | - | - | - | - |
| Gbar_D03_819566 | C/T | 0.08 | 6.33 | 6.88 | - | - | - | - | Gbar_D03_861183 | T/C | 0.08 | 6.80 | 7.35 | - | - | - | - |
| Gbar_D03_820090 | A/C | 0.07 | 6.17 | 6.26 | - | - | - | - | Gbar_D03_861307 | G/A | 0.08 | 6.18 | 6.73 | - | - | - | - |
| Gbar_D03_821496 | T/C | 0.08 | 6.65 | 7.23 | - | - | - | - | Gbar_D03_861348 | C/T | 0.08 | 6.24 | 6.96 | - | - | - | - |
| Gbar_D03_821544 | C/T | 0.08 | 6.49 | 6.74 | - | - | - | - | Gbar_D03_861362 | A/G | 0.08 | 6.48 | 6.87 | - | - | - | - |
| Gbar_D03_822027 | A/T | 0.08 | 7.10 | 7.53 | - | - | - | 6.05 | Gbar_D03_861829 | G/T | 0.08 | 6.01 | 6.29 | - | - | - | - |
| Gbar_D03_822378 | A/C | 0.08 | - | 6.15 | - | - | - | - | Gbar_D03_861889 | A/G | 0.08 | 6.39 | 6.93 | - | - | - | - |
| Gbar_D03_822389 | G/A | 0.08 | - | 6.15 | - | - | - | - | Gbar_D03_862149 | C/G | 0.08 | - | 6.41 | - | - | - | - |
| Gbar_D03_823477 | G/T | 0.08 | 6.86 | 6.90 | - | - | - | - | Gbar_D03_863216 | A/G | 0.08 | 6.79 | 7.05 | - | - | - | - |
| Gbar_D03_825160 | G/T | 0.08 | 6.91 | 7.50 | - | - | - | - | Gbar_D03_863260 | A/T | 0.08 | - | 6.09 | - | - | - | - |
| Gbar_D03_825670 | A/G | 0.08 | 6.64 | 6.77 | - | - | - | - | Gbar_D03_863288 | T/A | 0.08 | 6.77 | 6.97 | - | - | - | - |
| Gbar_D03_826341 | T/C | 0.07 | 6.37 | 6.72 | - | - | - | - | Gbar_D03_863380 | A/G | 0.08 | 6.74 | 6.79 | - | - | - | - |
| Gbar_D03_826386 | A/T | 0.07 | 6.35 | 6.79 | - | - | - | - | Gbar_D03_863504 | G/T | 0.08 | - | 6.21 | - | - | - | - |
| Gbar_D03_826631 | G/A | 0.08 | 7.92 | 8.32 | - | - | - | 6.09 | Gbar_D03_864223 | T/C | 0.08 | 6.18 | 6.43 | - | - | - | - |
| Gbar_D03_826777 | C/T | 0.08 | 7.14 | 7.59 | - | - | - | - | Gbar_D03_864909 | A/G | 0.08 | 6.69 | 7.06 | - | - | - | - |
| Gbar_D03_827276 | G/A | 0.08 | - | 6.81 | - | - | - | - | Gbar_D03_865179 | G/A | 0.08 | 6.50 | 6.82 | - | - | - | - |
| Gbar_D03_829019 | A/G | 0.08 | 6.60 | 7.05 | - | - | - | - | Gbar_D03_865347 | T/C | 0.08 | 6.89 | 7.39 | - | - | - | - |
| Gbar_D03_829425 | A/T | 0.08 | 6.58 | 7.06 | - | - | - | - | Gbar_D03_866738 | C/A | 0.08 | 6.51 | 6.53 | - | - | - | - |
| Gbar_D03_830950 | A/G | 0.08 | - | 6.00 | - | - | - | - | Gbar_D03_866739 | G/T | 0.08 | 6.45 | 6.46 | - | - | - | - |
| Gbar_D03_831211 | T/C | 0.08 | - | 6.15 | - | - | - | - | Gbar_D03_867612 | T/A | 0.08 | 6.75 | 7.44 | - | - | - | - |
| Gbar_D03_831772 | A/G | 0.07 | 6.09 | 6.60 | - | - | - | - | Gbar_D03_868257 | G/A | 0.08 | - | 6.46 | - | - | - | - |
| Gbar_D03_831844 | C/T | 0.08 | - | 6.40 | - | - | - | - | Gbar_D03_868367 | G/A | 0.08 | 6.76 | 7.37 | - | - | - | - |
| Gbar_D03_831944 | T/A | 0.08 | 6.80 | 7.18 | - | - | - | - | Gbar_D03_868494 | G/A | 0.08 | - | 6.28 | - | - | - | - |
| Gbar_D03_832044 | G/A | 0.08 | 6.97 | 7.01 | - | - | - | - | Gbar_D03_868547 | T/C | 0.08 | 6.43 | 6.94 | - | - | - | - |
| Gbar_D03_832128 | T/C | 0.08 | 6.54 | 7.26 | - | - | - | - | Gbar_D03_869436 | C/A | 0.08 | 6.83 | 6.92 | - | - | - | - |
| Gbar_D03_832209 | G/A | 0.08 | 6.81 | 7.26 | - | - | - | - | Gbar_D03_870721 | G/T | 0.08 | 6.77 | 7.26 | - | - | - | - |
| Gbar_D03_832789 | C/G | 0.08 | 7.02 | 7.41 | - | - | - | - | Gbar_D03_871316 | C/T | 0.08 | - | 6.55 | - | - | - | - |
| Gbar_D03_833775 | T/C | 0.07 | 6.07 | 6.44 | - | - | - | - | Gbar_D03_872949 | C/T | 0.08 | 6.68 | 7.26 | - | - | - | - |
| Gbar_D03_833918 | T/C | 0.08 | 6.46 | 6.98 | - | - | - | - | Gbar_D03_874004 | T/C | 0.07 | - | 6.02 | - | - | - | - |
| Gbar_D03_834213 | G/A | 0.08 | 6.30 | 7.11 | - | - | - | - | Gbar_D03_875334 | T/A | 0.08 | 6.74 | 6.79 | - | - | - | - |
| Gbar_D03_834357 | G/A | 0.08 | 6.93 | 7.09 | - | - | - | - | Gbar_D03_876178 | A/G | 0.08 | 6.45 | 6.96 | - | - | - | - |
| Gbar_D03_834385 | G/A | 0.08 | 6.35 | 6.41 | - | - | - | - | Gbar_D03_876290 | G/T | 0.08 | 6.05 | 6.66 | - | - | - | - |
| Gbar_D03_834806 | T/C | 0.08 | 7.22 | 7.57 | - | - | - | - | Gbar_D03_877058 | A/G | 0.09 | - | 6.17 | - | - | - | - |
| Gbar_D03_835978 | A/G | 0.08 | 6.77 | 7.33 | - | - | - | - | Gbar_D03_877654 | G/A | 0.08 | 6.76 | 6.96 | - | - | - | - |

**Table S3.** Summary of the total associated SNPs identified through GWAS for *FW* resistance in Sea Island cotton. (Continued)

| SNP | Ref/Alt | MAF | -log (P-value) in different environments | | | | | | SNP | Ref/Alt | MAF | -log (P-value) in different environments | | | | | |
| --- | --- | --- | --- | --- | --- | --- | --- | --- | --- | --- | --- | --- | --- | --- | --- | --- | --- |
|  |  |  | 2015 | 2016 | 2018 | 2019 | Mean | BLUP |  |  |  | 2015 | 2016 | 2018 | 2019 | Mean | BLUP |
| Gbar_D03_880446 | C/G | 0.08 | 6.71 | 7.14 | - | - | - | - | Gbar_D03_910027 | A/T | 0.08 | - | 6.21 | - | - | - | - |
| Gbar_D03_881676 | G/A | 0.08 | 6.86 | 7.47 | - | - | - | - | Gbar_D03_910165 | T/C | 0.08 | 6.59 | 6.98 | - | - | - | - |
| Gbar_D03_882444 | T/A | 0.08 | 6.66 | 6.87 | - | - | - | - | Gbar_D03_911317 | C/A | 0.08 | 6.47 | 7.00 | - | - | - | - |
| Gbar_D03_882817 | T/A | 0.08 | 6.92 | 7.15 | - | - | - | - | Gbar_D03_911427 | G/A | 0.08 | 6.64 | 7.12 | - | - | - | - |
| Gbar_D03_882889 | G/A | 0.08 | - | 6.36 | - | - | - | - | Gbar_D03_913182 | C/T | 0.08 | 6.82 | 7.48 | - | - | - | - |
| Gbar_D03_883127 | G/A | 0.08 | - | 6.52 | - | - | - | - | Gbar_D03_913474 | T/G | 0.08 | - | 6.08 | - | - | - | - |
| Gbar_D03_883704 | C/G | 0.08 | 6.15 | 6.18 | - | - | - | - | Gbar_D03_913749 | G/A | 0.08 | 6.08 | 6.40 | - | - | - | - |
| Gbar_D03_883984 | A/T | 0.08 | - | 6.24 | - | - | - | - | Gbar_D03_913869 | G/A | 0.08 | 6.17 | 6.22 | - | - | - | - |
| Gbar_D03_884142 | A/G | 0.08 | - | 6.61 | - | - | - | - | Gbar_D03_914283 | G/C | 0.08 | 6.10 | 6.79 | - | - | - | - |
| Gbar_D03_884147 | A/T | 0.08 | 6.39 | 7.05 | - | - | - | - | Gbar_D03_915438 | C/G | 0.08 | 6.63 | 7.07 | - | - | - | - |
| Gbar_D03_884158 | A/T | 0.08 | 6.31 | 6.89 | - | - | - | - | Gbar_D03_915814 | T/C | 0.07 | - | 6.12 | - | - | - | - |
| Gbar_D03_884404 | A/C | 0.07 | 6.39 | 6.54 | - | - | - | - | Gbar_D03_916489 | T/G | 0.08 | 6.07 | 6.61 | - | - | - | - |
| Gbar_D03_884492 | A/G | 0.08 | 6.51 | 7.03 | - | - | - | - | Gbar_D03_917666 | C/T | 0.09 | - | 6.24 | - | - | - | - |
| Gbar_D03_884660 | A/C | 0.08 | 6.02 | 6.19 | - | - | - | - | Gbar_D03_918390 | G/A | 0.08 | 7.03 | 7.12 | - | - | - | - |
| Gbar_D03_885798 | A/T | 0.08 | 6.36 | 7.06 | - | - | - | - | Gbar_D03_918454 | A/G | 0.08 | 6.98 | 7.63 | - | - | - | - |
| Gbar_D03_885841 | C/A | 0.08 | 6.12 | 6.54 | - | - | - | - | Gbar_D03_919295 | G/T | 0.08 | 6.21 | 6.31 | - | - | - | - |
| Gbar_D03_886146 | C/T | 0.08 | - | 6.01 | - | - | - | - | Gbar_D03_919316 | A/T | 0.08 | 6.33 | 6.72 | - | - | - | - |
| Gbar_D03_886165 | G/A | 0.08 | - | 6.49 | - | - | - | - | Gbar_D03_919375 | A/G | 0.07 | - | 6.24 | - | - | - | - |
| Gbar_D03_886259 | C/T | 0.08 | 6.51 | 6.73 | - | - | - | - | Gbar_D03_919378 | T/C | 0.08 | - | 6.43 | - | - | - | - |
| Gbar_D03_886524 | C/A | 0.08 | 6.27 | 7.07 | - | - | - | - | Gbar_D03_919408 | C/T | 0.08 | - | 7.12 | - | - | - | - |
| Gbar_D03_886537 | C/T | 0.08 | 6.24 | 6.82 | - | - | - | - | Gbar_D03_919480 | T/G | 0.08 | 6.21 | 7.33 | - | - | - | - |
| Gbar_D03_887470 | G/A | 0.08 | - | 6.10 | - | - | - | - | Gbar_D03_919504 | G/A | 0.08 | 6.30 | 6.76 | - | - | - | - |
| Gbar_D03_887508 | T/C | 0.08 | - | 6.19 | - | - | - | - | Gbar_D03_920104 | A/G | 0.08 | 7.30 | 7.57 | - | - | - | - |
| Gbar_D03_887891 | A/G | 0.08 | 6.03 | 6.43 | - | - | - | - | Gbar_D03_920484 | C/T | 0.08 | - | 6.24 | - | - | - | - |
| Gbar_D03_888250 | T/C | 0.08 | 7.21 | 7.66 | - | - | - | - | Gbar_D03_922129 | T/C | 0.08 | 6.43 | 6.79 | - | - | - | - |
| Gbar_D03_888540 | A/G | 0.08 | - | 6.56 | - | - | - | - | Gbar_D03_923381 | C/T | 0.08 | 6.27 | 6.34 | - | - | - | - |
| Gbar_D03_888570 | T/C | 0.08 | 6.09 | 6.65 | - | - | - | - | Gbar_D03_925438 | T/A | 0.08 | 6.25 | 6.87 | - | - | - | - |
| Gbar_D03_889027 | C/T | 0.08 | - | 6.45 | - | - | - | - | Gbar_D03_925463 | C/T | 0.08 | 6.51 | 7.28 | - | - | - | - |
| Gbar_D03_889762 | G/A | 0.08 | 6.15 | 6.83 | - | - | - | - | Gbar_D03_925948 | C/T | 0.08 | 6.20 | 6.79 | - | - | - | - |
| Gbar_D03_889853 | G/A | 0.08 | 6.67 | 7.25 | - | - | - | - | Gbar_D03_926451 | G/A | 0.08 | 6.11 | 6.02 | - | - | - | - |
| Gbar_D03_890328 | G/A | 0.08 | 6.85 | 7.45 | - | - | - | - | Gbar_D03_926550 | A/G | 0.08 | 6.49 | 6.98 | - | - | - | - |
| Gbar_D03_891244 | C/G | 0.09 | 6.13 | 6.19 | - | - | - | - | Gbar_D03_927253 | A/T | 0.08 | - | 6.34 | - | - | - | - |
| Gbar_D03_891425 | A/T | 0.08 | 6.40 | 7.07 | - | - | - | - | Gbar_D03_927586 | A/G | 0.08 | - | 6.06 | - | - | - | - |
| Gbar_D03_891465 | T/C | 0.08 | 6.70 | 6.92 | - | - | - | - | Gbar_D03_927832 | C/T | 0.08 | - | 6.46 | - | - | - | - |
| Gbar_D03_891531 | C/T | 0.08 | - | 6.28 | - | - | - | - | Gbar_D03_927886 | C/T | 0.08 | 6.05 | 6.69 | - | - | - | - |
| Gbar_D03_891608 | G/A | 0.08 | - | 6.65 | - | - | - | - | Gbar_D03_928268 | A/C | 0.08 | 6.15 | 7.13 | - | - | - | - |
| Gbar_D03_891771 | A/C | 0.08 | 6.60 | 6.58 | - | - | - | - | Gbar_D03_928586 | T/A | 0.08 | 6.54 | 7.00 | - | - | - | - |
| Gbar_D03_891846 | A/G | 0.08 | 6.02 | 6.76 | - | - | - | - | Gbar_D03_928931 | A/T | 0.08 | 6.39 | 6.78 | - | - | - | - |
| Gbar_D03_892558 | A/G | 0.08 | 6.57 | 6.92 | - | - | - | - | Gbar_D03_928963 | T/A | 0.08 | 6.88 | 7.02 | - | - | - | - |
| Gbar_D03_892638 | A/G | 0.08 | 6.67 | 7.08 | - | - | - | - | Gbar_D03_929263 | A/C | 0.08 | 6.44 | 6.94 | - | - | - | - |
| Gbar_D03_892644 | G/A | 0.08 | 6.68 | 6.85 | - | - | - | - | Gbar_D03_929354 | C/T | 0.08 | 6.19 | 6.28 | - | - | - | - |
| Gbar_D03_892753 | G/A | 0.08 | - | 6.05 | - | - | - | - | Gbar_D03_929439 | A/G | 0.08 | 6.85 | 6.86 | - | - | - | - |
| Gbar_D03_892901 | T/C | 0.08 | 6.03 | 6.43 | - | - | - | - | Gbar_D03_929567 | T/C | 0.08 | 6.33 | 6.61 | - | - | - | - |
| Gbar_D03_892950 | G/A | 0.08 | - | 6.03 | - | - | - | - | Gbar_D03_929578 | C/T | 0.08 | 6.16 | 6.53 | - | - | - | - |
| Gbar_D03_893090 | T/C | 0.08 | 6.35 | 6.43 | - | - | - | - | Gbar_D03_929895 | A/G | 0.08 | 6.59 | 7.20 | - | - | - | - |
| Gbar_D03_893103 | C/T | 0.08 | 6.18 | 6.68 | - | - | - | - | Gbar_D03_929903 | C/T | 0.08 | 6.61 | 7.30 | - | - | - | - |
| Gbar_D03_893132 | T/A | 0.08 | - | 6.46 | - | - | - | - | Gbar_D03_929941 | T/C | 0.08 | 6.57 | 7.36 | - | - | - | - |
| Gbar_D03_893264 | A/G | 0.08 | 6.32 | 6.75 | - | - | - | - | Gbar_D03_930105 | T/A | 0.08 | 6.72 | 7.22 | - | - | - | - |
| Gbar_D03_893355 | C/G | 0.08 | 6.42 | 6.95 | - | - | - | - | Gbar_D03_930482 | T/A | 0.08 | 6.33 | 6.69 | - | - | - | - |
| Gbar_D03_893458 | A/G | 0.08 | 6.64 | 7.07 | - | - | - | - | Gbar_D03_930562 | G/A | 0.08 | 6.33 | 6.61 | - | - | - | - |
| Gbar_D03_893670 | T/A | 0.08 | 6.41 | 6.90 | - | - | - | - | Gbar_D03_931362 | C/T | 0.08 | - | 6.07 | - | - | - | - |
| Gbar_D03_895012 | T/A | 0.08 | 6.65 | 7.14 | - | - | - | - | Gbar_D03_931881 | T/A | 0.08 | - | 6.27 | - | - | - | - |
| Gbar_D03_895774 | T/A | 0.08 | 6.28 | 6.67 | - | - | - | - | Gbar_D03_932359 | G/T | 0.08 | 6.99 | 7.01 | - | - | - | - |
| Gbar_D03_895885 | C/T | 0.08 | 6.07 | 6.65 | - | - | - | - | Gbar_D03_932811 | T/C | 0.08 | 6.80 | 7.39 | - | - | - | - |
| Gbar_D03_895969 | A/G | 0.08 | 6.29 | 6.77 | - | - | - | - | Gbar_D03_932910 | A/G | 0.08 | 6.36 | 6.57 | - | - | - | - |
| Gbar_D03_896192 | G/A | 0.08 | 6.57 | 7.14 | - | - | - | - | Gbar_D03_933036 | A/T | 0.08 | 6.43 | 6.88 | - | - | - | - |
| Gbar_D03_896279 | C/T | 0.08 | 6.09 | 6.41 | - | - | - | - | Gbar_D03_933312 | T/C | 0.08 | - | 6.06 | - | - | - | - |
| Gbar_D03_896764 | A/T | 0.08 | 6.58 | 6.99 | - | - | - | - | Gbar_D03_933325 | A/G | 0.08 | - | 6.15 | - | - | - | - |
| Gbar_D03_897819 | A/C | 0.08 | 6.83 | 7.13 | - | - | - | - | Gbar_D03_934354 | G/C | 0.09 | - | 6.16 | - | - | - | - |
| Gbar_D03_897840 | C/G | 0.08 | - | 6.37 | - | - | - | - | Gbar_D03_934504 | C/T | 0.08 | 6.12 | 7.02 | - | - | - | - |
| Gbar_D03_897899 | G/C | 0.08 | 6.20 | 6.90 | - | - | - | - | Gbar_D03_934684 | C/T | 0.08 | - | 6.62 | - | - | - | - |
| Gbar_D03_897901 | C/T | 0.08 | 6.22 | 6.89 | - | - | - | - | Gbar_D03_934831 | A/T | 0.08 | 6.31 | 7.02 | - | - | - | - |
| Gbar_D03_898193 | G/A | 0.08 | 6.62 | 7.26 | - | - | - | - | Gbar_D03_935005 | T/C | 0.08 | 6.31 | 6.66 | - | - | - | - |
| Gbar_D03_898245 | G/A | 0.08 | 6.38 | 7.17 | - | - | - | - | Gbar_D03_935178 | C/T | 0.08 | 6.04 | 6.97 | - | - | - | - |
| Gbar_D03_898356 | T/C | 0.08 | - | 6.33 | - | - | - | - | Gbar_D03_935248 | G/A | 0.08 | 7.21 | 7.21 | - | - | - | - |
| Gbar_D03_898377 | G/A | 0.08 | - | 6.34 | - | - | - | - | Gbar_D03_935584 | T/C | 0.08 | 6.76 | 7.09 | - | - | - | - |
| Gbar_D03_898709 | A/G | 0.08 | 6.49 | 6.75 | - | - | - | - | Gbar_D03_936037 | A/T | 0.08 | 6.10 | 6.12 | - | - | - | - |
| Gbar_D03_899069 | T/C | 0.08 | 6.40 | 6.50 | - | - | - | - | Gbar_D03_936082 | T/A | 0.08 | 6.20 | 6.36 | - | - | - | - |
| Gbar_D03_899482 | A/G | 0.08 | - | 6.16 | - | - | - | - | Gbar_D03_936812 | A/G | 0.08 | 6.67 | 7.09 | - | - | - | - |
| Gbar_D03_900042 | T/C | 0.08 | - | 6.75 | - | - | - | - | Gbar_D03_937095 | T/A | 0.08 | 6.13 | 7.02 | - | - | - | - |
| Gbar_D03_900147 | C/T | 0.08 | 6.17 | 6.85 | - | - | - | - | Gbar_D03_937309 | G/T | 0.07 | 6.48 | 6.45 | - | - | - | - |
| Gbar_D03_900804 | T/C | 0.08 | 6.68 | 7.15 | - | - | - | - | Gbar_D03_937576 | T/C | 0.08 | 6.20 | 6.59 | - | - | - | - |
| Gbar_D03_901197 | G/A | 0.07 | 6.30 | 6.34 | - | - | - | - | Gbar_D03_937673 | A/T | 0.08 | 6.93 | 7.33 | - | - | - | - |
| Gbar_D03_901272 | G/A | 0.08 | 6.60 | 7.05 | - | - | - | - | Gbar_D03_937692 | G/A | 0.08 | 7.00 | 7.40 | - | - | - | - |
| Gbar_D03_901276 | G/T | 0.08 | 6.76 | 6.99 | - | - | - | - | Gbar_D03_938152 | T/C | 0.08 | - | 6.04 | - | - | - | - |
| Gbar_D03_901536 | C/A | 0.08 | 6.87 | 6.67 | - | - | - | - | Gbar_D03_938167 | A/G | 0.08 | 6.04 | 6.10 | - | - | - | - |
| Gbar_D03_902288 | G/C | 0.08 | 6.44 | 6.90 | - | - | - | - | Gbar_D03_938670 | T/C | 0.08 | - | 6.19 | - | - | - | - |
| Gbar_D03_902828 | T/C | 0.08 | - | 6.50 | - | - | - | - | Gbar_D03_939095 | C/T | 0.08 | 6.86 | 7.46 | - | - | - | - |
| Gbar_D03_903181 | G/A | 0.08 | - | 6.22 | - | - | - | - | Gbar_D03_939478 | G/A | 0.08 | 6.70 | 7.03 | - | - | - | - |
| Gbar_D03_903930 | T/A | 0.08 | 7.07 | 7.66 | - | - | - | - | Gbar_D03_940347 | A/T | 0.08 | 6.48 | 6.85 | - | - | - | - |
| Gbar_D03_903935 | G/A | 0.08 | 6.93 | 7.60 | - | - | - | - | Gbar_D03_940981 | T/G | 0.08 | 6.19 | 6.30 | - | - | - | - |
| Gbar_D03_904409 | G/T | 0.08 | 6.40 | 6.90 | - | - | - | - | Gbar_D03_942540 | A/G | 0.08 | 6.89 | 7.14 | - | - | - | - |
| Gbar_D03_906027 | G/C | 0.08 | 6.76 | 6.78 | - | - | - | - | Gbar_D03_942558 | C/T | 0.08 | 7.09 | 7.51 | - | - | - | - |
| Gbar_D03_906494 | T/C | 0.08 | 6.79 | 7.37 | - | - | - | - | Gbar_D03_942661 | G/A | 0.08 | 6.52 | 7.08 | - | - | - | - |
| Gbar_D03_906561 | A/C | 0.08 | 6.34 | 6.72 | - | - | - | - | Gbar_D03_942735 | A/C | 0.08 | 6.70 | 7.32 | - | - | - | - |
| Gbar_D03_906646 | A/T | 0.08 | 7.13 | 7.18 | - | - | - | - | Gbar_D03_942849 | C/A | 0.08 | 6.76 | 7.19 | - | - | - | - |
| Gbar_D03_906765 | T/G | 0.08 | 6.89 | 7.28 | - | - | - | - | Gbar_D03_942868 | A/G | 0.08 | 6.02 | 6.36 | - | - | - | - |
| Gbar_D03_907447 | A/G | 0.08 | - | 6.75 | - | - | - | - | Gbar_D03_953927 | G/A | 0.08 | 6.95 | 6.80 | - | - | - | - |
| Gbar_D03_907819 | G/T | 0.08 | 6.68 | 7.30 | - | - | - | - | Gbar_D03_959041 | A/G | 0.08 | 6.46 | 7.26 | - | - | - | - |
| Gbar_D03_907826 | C/T | 0.08 | 6.60 | 7.08 | - | - | - | - | Gbar_D03_968233 | A/T | 0.08 | 6.58 | 6.57 | - | - | - | - |
| Gbar_D03_907859 | G/A | 0.08 | 6.53 | 6.97 | - | - | - | - | Gbar_D03_970200 | A/C | 0.08 | 6.09 | - | - | - | - | - |
| Gbar_D03_908044 | A/T | 0.08 | 6.49 | 6.69 | - | - | - | - | Gbar_D03_987798 | G/A | 0.09 | - | 6.08 | - | - | - | - |
| Gbar_D03_908268 | A/G | 0.08 | 6.75 | 7.07 | - | - | - | - | Gbar_D03_987802 | T/G | 0.09 | 6.15 | 6.42 | - | - | - | - |
| Gbar_D03_908768 | A/C | 0.08 | 6.12 | 6.55 | - | - | - | - | Gbar_D03_993066 | T/C | 0.13 | - | - | - | 6.15 | 6.25 | 6.44 |
| Gbar_D03_908812 | T/C | 0.08 | - | 6.39 | - | - | - | - | Gbar_D03_995976 | T/C | 0.13 | - | - | - | 6.05 | - | 6.06 |
| Gbar_D03_909011 | G/C | 0.08 | 6.31 | 6.92 | - | - | - | - | Gbar_D05_54270545 | T/A | 0.06 | - | - | 6.32 | - | - | - |
| Gbar_D03_909073 | C/T | 0.08 | 6.85 | 7.31 | - | - | - | - | Gbar_D06_44882448 | C/A | 0.17 | - | - | - | 6.19 | - | 6.03 |
| Gbar_D03_909136 | G/T | 0.08 | - | 6.54 | - | - | - | - | Gbar_D12_5382642 | T/G | 0.11 | - | 6.17 | - | - | - | - |

**Table S4.** Distribution of the SNP number on 26 chromosomes of Sea Island cotton.

| Chromosome | 2015 | 2016 | 2018 | 2019 | Mean | BLUP |
| --- | --- | --- | --- | --- | --- | --- |
| A01 | 0 | 0 | 0 | 0 | 0 | 0 |
| A02 | 0 | 0 | 0 | 1 | 0 | 1 |
| A03 | 0 | 0 | 0 | 0 | 0 | 0 |
| A04 | 0 | 0 | 0 | 0 | 0 | 0 |
| A05 | 0 | 0 | 0 | 0 | 0 | 0 |
| A06 | 0 | 0 | 0 | 0 | 0 | 0 |
| A07 | 0 | 0 | 0 | 0 | 1 | 1 |
| A08 | 0 | 0 | 0 | 0 | 0 | 0 |
| A09 | 0 | 0 | 0 | 0 | 0 | 0 |
| A10 | 0 | 0 | 0 | 0 | 0 | 0 |
| A11 | 0 | 0 | 0 | 3 | 8 | 3 |
| A12 | 0 | 0 | 0 | 0 | 0 | 0 |
| A13 | 0 | 0 | 0 | 1 | 1 | 1 |
| D01 | 0 | 0 | 0 | 0 | 0 | 0 |
| D02 | 0 | 0 | 0 | 0 | 0 | 0 |
| D03 | 1513 | 2128 | 169 | 236 | 199 | 256 |
| D04 | 0 | 0 | 0 | 0 | 0 | 0 |
| D05 | 0 | 0 | 1 | 0 | 0 | 0 |
| D06 | 0 | 0 | 0 | 1 | 0 | 1 |
| D07 | 0 | 0 | 0 | 0 | 0 | 0 |
| D08 | 0 | 0 | 0 | 0 | 0 | 0 |
| D09 | 0 | 0 | 0 | 0 | 0 | 0 |
| D10 | 0 | 0 | 0 | 0 | 0 | 0 |
| D11 | 0 | 0 | 0 | 0 | 0 | 0 |
| D12 | 0 | 1 | 0 | 0 | 0 | 0 |
| D13 | 0 | 0 | 0 | 0 | 0 | 0 |
| Total No. | 1513 | 2129 | 170 | 242 | 209 | 263 |

**Table S5.** Information of the core SNPs on D03 chromosome identified in this study.

| SNP | Position (bp) | | Reference | Alternate | MAF | -log (*P*-value) in different environments | | | | | |
| --- | --- | --- | --- | --- | --- | --- | --- | --- | --- | --- | --- |
|  |  |  |  |  |  | 2015 | 2016 | 2018 | 2019 | Mean | BLUP |
| Gbar_D03_1068053 | | 1,068,053 | A | G | 0.15 | 6.22 | 6.45 | 6.82 | 8.86 | 8.30 | 8.63 |
| Gbar_D03_1075812 | | 1,075,812 | A | G | 0.15 | 6.01 | 6.34 | 6.49 | 8.75 | 8.29 | 8.59 |
| Gbar_D03_1092095 | | 1,092,095 | G | C | 0.16 | 6.30 | - | 6.27 | 9.02 | 8.57 | 8.86 |
| Gbar_D03_1105012 | | 1,105,012 | G | T | 0.16 | 6.55 | 7.70 | 6.64 | 8.16 | 8.14 | 8.40 |
| Gbar_D03_1107259 | | 1,107,259 | A | C | 0.16 | 6.20 | 6.78 | 6.79 | 8.42 | 8.07 | 8.37 |
| Gbar_D03_1110567 | | 1,110,567 | A | G | 0.21 | 6.99 | 8.11 | 10.00 | 13.12 | 11.01 | 11.57 |
| Gbar_D03_1159232 | | 1,159,232 | A | G | 0.16 | 6.23 | 6.66 | 7.06 | 8.52 | 7.97 | 8.29 |
| Gbar_D03_1343889 | | 1,343,889 | A | G | 0.16 | - | - | 7.33 | 10.04 | 8.93 | 9.31 |
| Gbar_D03_1629970 | | 1,629,970 | C | T | 0.18 | - | 6.94 | 8.64 | 11.52 | 10.10 | 10.52 |
| Gbar_D03_1664579 | | 1,664,579 | T | C | 0.18 | - | - | 8.13 | 10.44 | 8.89 | 9.27 |
| Gbar_D03_1672718 | | 1,672,718 | G | A | 0.19 | - | 6.09 | 7.88 | 10.24 | 8.86 | 9.23 |
| Gbar_D03_1676638 | | 1,676,638 | A | C | 0.19 | - | - | 7.82 | 10.45 | 9.06 | 9.42 |
| Gbar_D03_2215669 | | 2,215,669 | A | T | 0.17 | 6.51 | 6.94 | 7.89 | 9.01 | 8.42 | 8.77 |
| Gbar_D03_2224222 | | 2,224,222 | A | G | 0.20 | - | 6.30 | 7.06 | 8.90 | 8.40 | 8.72 |


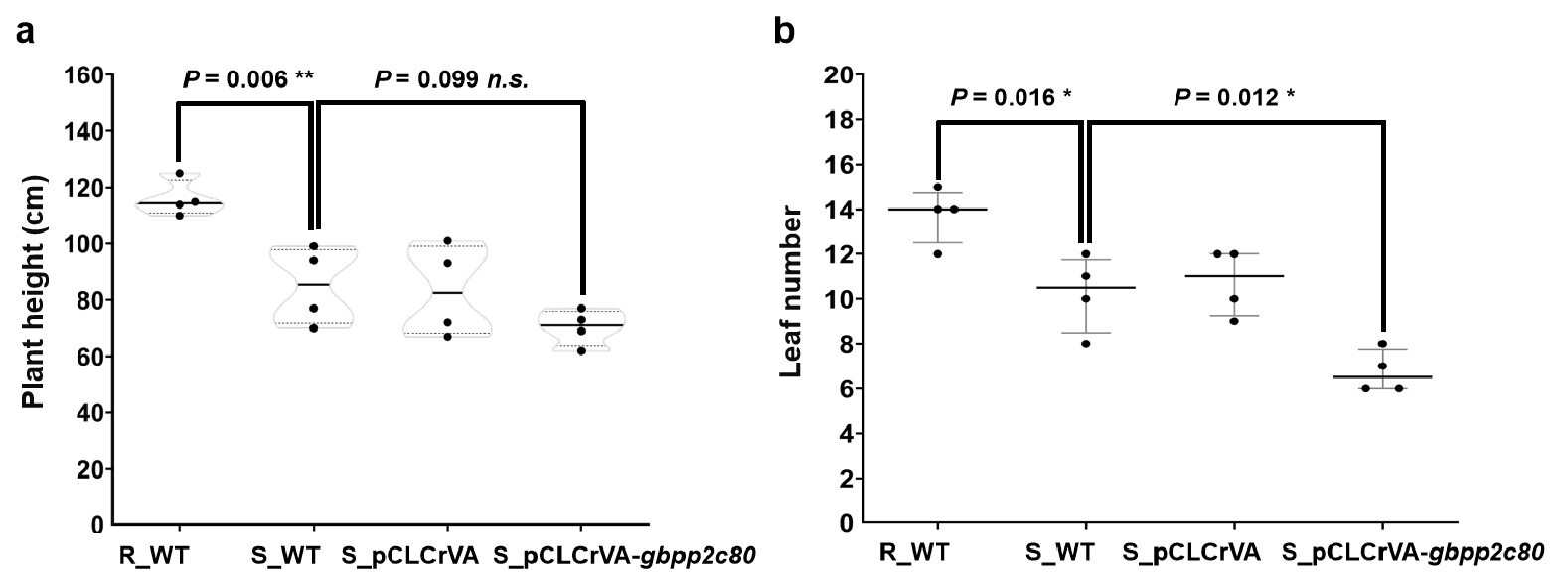


**Figure S2.** *GbPP2C80* affects the balance between *FW* resistance and plant growth in Sea Island cotton. (a) Plant height (cm) of *GbPP2C80*-silenced Sea Island cotton individuals and the controls. R: *FW*-resistant variety, T10-280. S: *FW*-susceptible variety, Ⅱ15-3464. WT: wild type. S_pCLCrVA: *FW*-susceptible variety containing pCLCrVA empty vector. S_pCLCrVA-*gbpp2c80*: *GbPP2C80*-silenced *FW*-susceptible lines. *n* = 4 individuals. (b) Leaf number of *GbPP2C80*-silenced Sea Island cotton individuals and the controls. *n* = 4 individuals.The significant level (*P*) was calculated by two-tailed *t*-test. *, **, and *n.s.* represent *P* < 0.05, *P* < 0.01, and not significant, respectively.


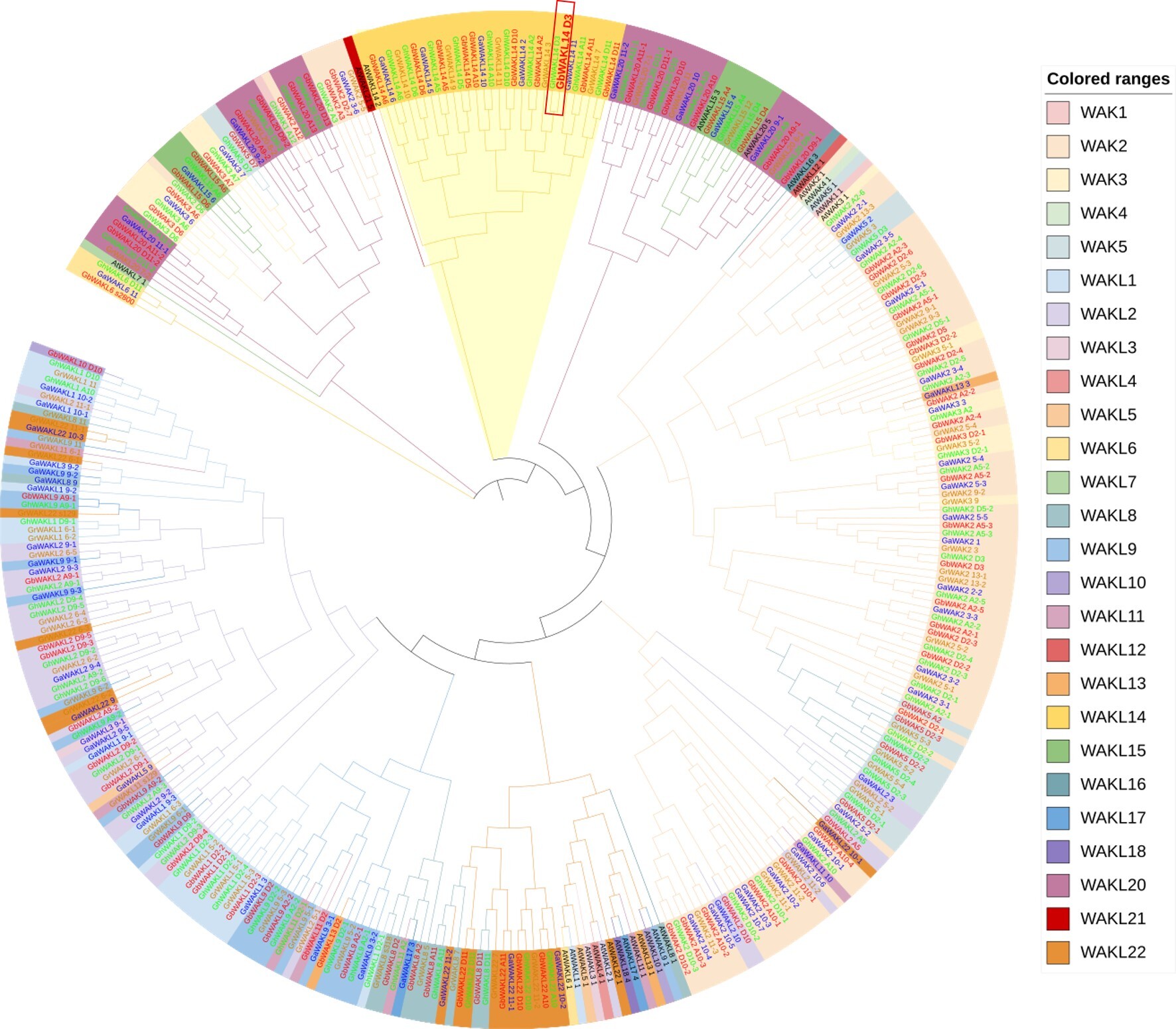


**Figure S3.** The phylogenetic tree built with WAK(L)s proteins in *G.barbadense* (Gb), *G. hirsutum* (Gh), *G. raimondii* (Gr), *G. arboreum* (Ga), and *A. thaliana* (At). The tree was built in software MEGA7 ^[77]^ by using the maximum likelihood method, and the bootstrap was set to 1000 replicates. The image was beautified in online software iTol (https://itol.embl.de/). Based on 26 *Arabidopsis* WAK(L) genes (*WAK1-WAK5*, *WAKL1-WAKL22*, no *WAKL19*), the genes in cotton were divided into 26 types and labeled in 26 colors. The genes were named as "species abbreviation (i.e., Gb) + gene anotation (i.e., WAKL14) + chromosome number (i.e., D03) or + copy number (i.e., -3)". Unlike the other types of genes, the *WAKL14*-type genes were relatively conserved and clustered, highlighted by a yellowish triangle. The *FW* resistance gene, *GbWAKL14_D03* (in *G. barbadense*), and its homology, *GhWAKL14_D03* (in *G. hirsutum*) have the closest relationship (marked in a red box), both of which originated from *GrWAKL14_3* (*in G. raimondii*).


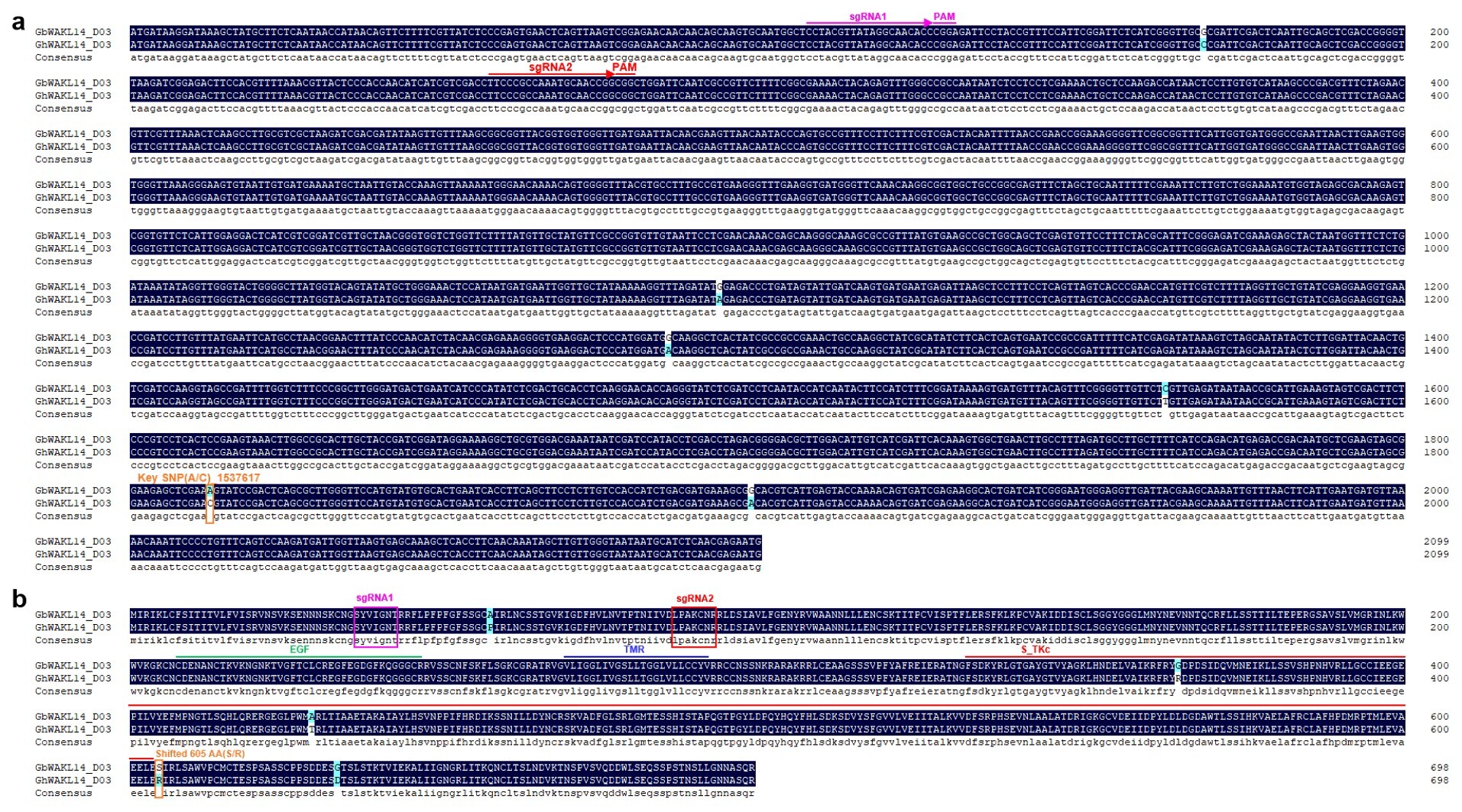


**Figure S4.** The alignments of CDS and protein sequences of *GbWAKL14* (D03) in Sea Island cotton and its homology *GhWAKL14* (D03) in upland cotton. (a) The alignments of CDS sequences of *GbWAKL14* (D03) and *GhWAKL14* (D03). The locations of the sgRNA1 with PAM, sgRNA2 with PAM, and the key SNP (A/C_1537617) were highlighted with pink line, red line, and orange box, respectively. (b) The alignments of protein sequences of *GbWAKL14* (D03) and *GhWAKL14* (D03). The locations of the sgRNA1 with PAM, sgRNA2 with PAM, and the key amino acid (S/R_605AA) were highlighted with pink, red, and orange boxes, respectively. The domains, EGF (Epidermal growth factor-like domain), TMR (Transmembrane region), S_TKc (Serine/Threonine protein kinases, catalytic domain) were marked with green, blue, and red lines.


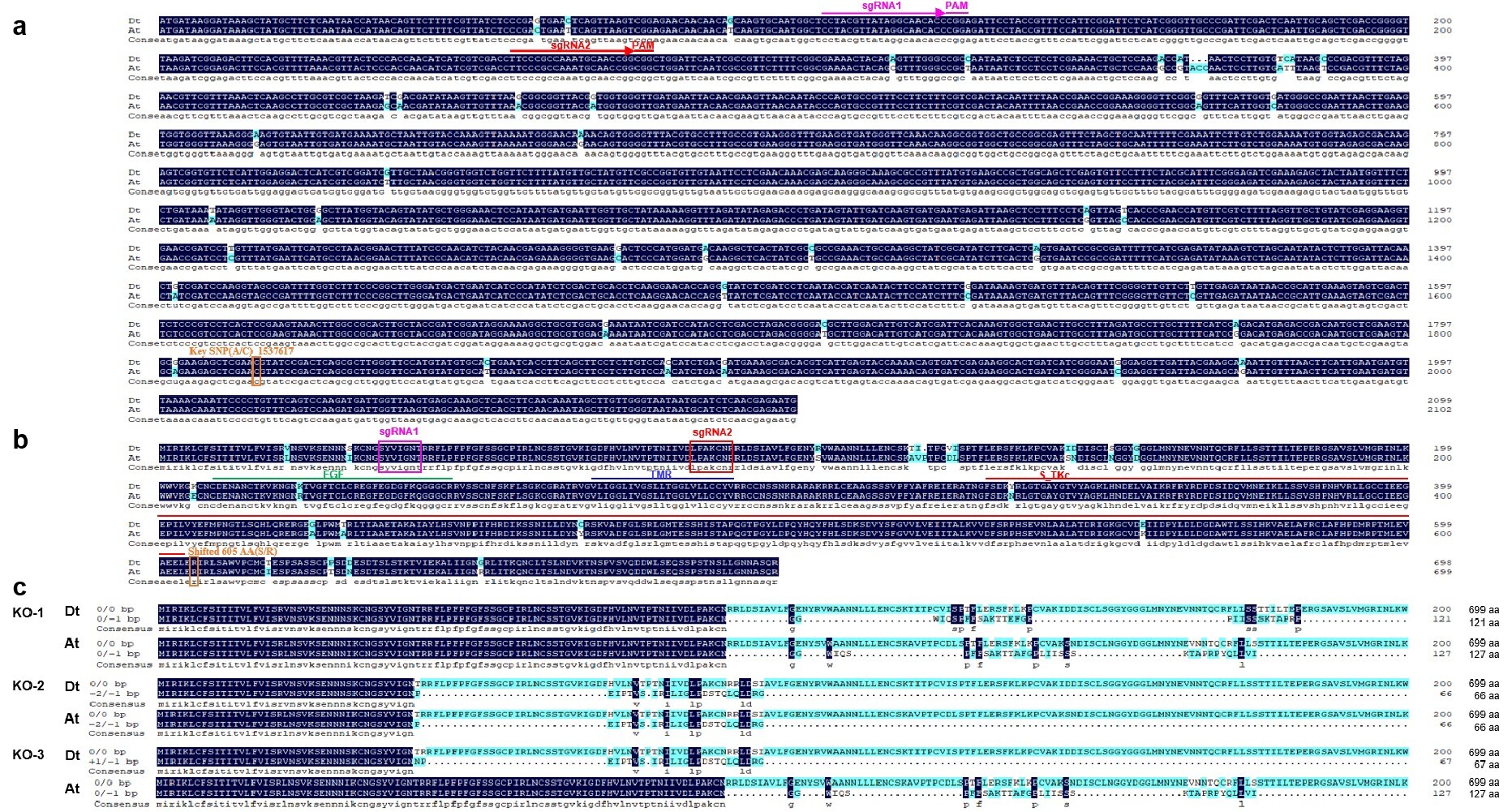


**Figure S5.** The locations of the sgRNAs and the editing effects in the Dt and At homologoues of *GbWAKL14*.(a) The locations of two sgRNAs and the key SNP in CDS sequences of *GbWAKL14* (Dt) and its homologue in At. (b) The locations of two sgRNAs and the key amino acid in protein sequences of *GbWAKL14* (Dt) and its homologue in At. (c) The mutations in three knock-out lines caused the premature termination of protein translation.


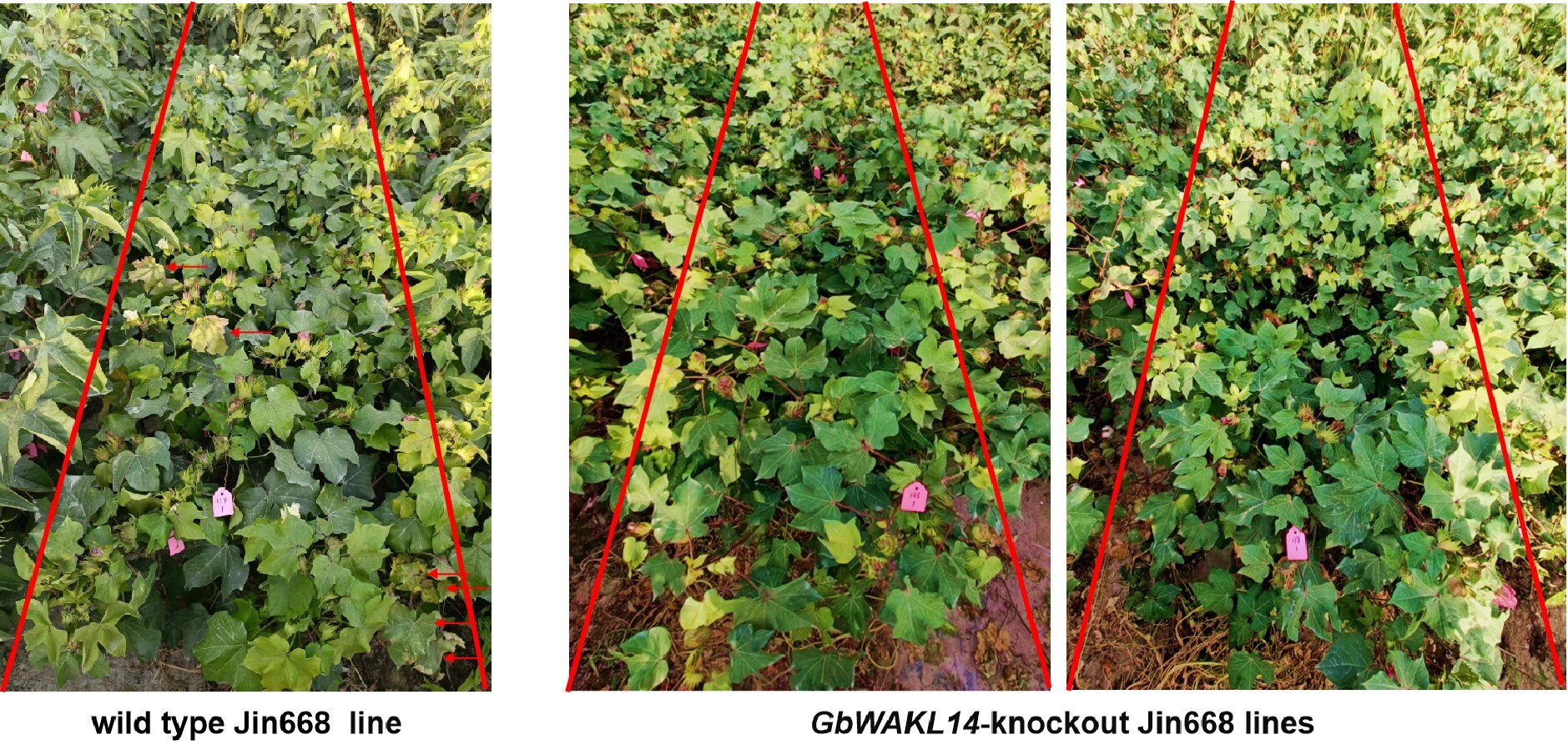


**Figure S6.** Wilt disease phenotypes of wild type and *GbWAKL14*-knockout Jin668 lines in the field. The red arrows show the *VW* diseased leaves. The light green leaves were not caused by the wilt disease but by the strong light.


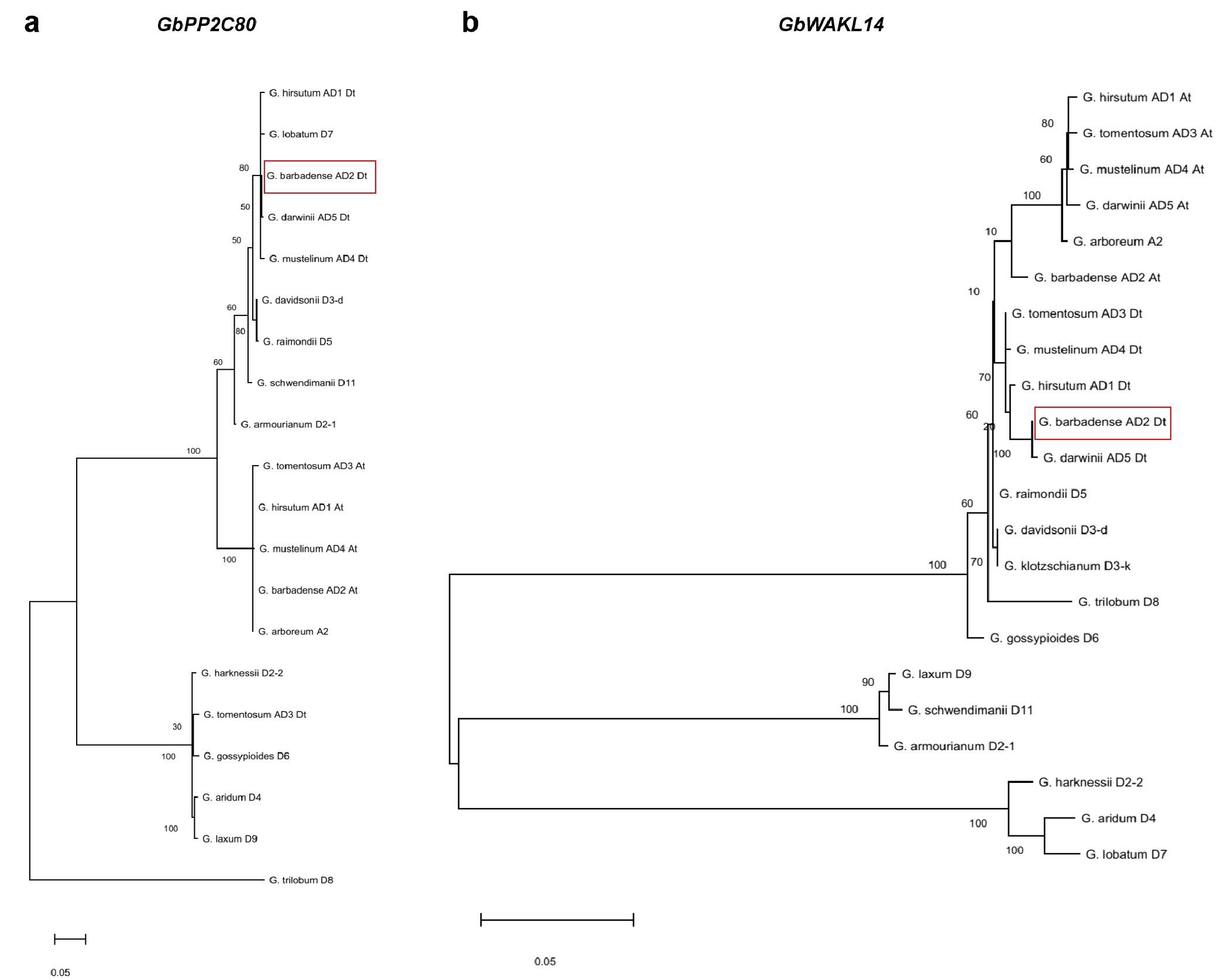


**Figure S7.** *GbPP2C80* and *GbWAKL14* are derived from the D genome ancestral cotton species. (a) Evolutionary tree of *GbPP2C80*in A_2_, D genome and AD genome tetraploid cotton species. (b) Evolutionary tree of *GbWAKL14* in A_2_, D genome and AD genome tetraploid cotton species. The labeled "G. barbadense AD2 Dt" corresponds to the candidate genes, *GbPP2C80* and *GbWAKL14*, in this study.


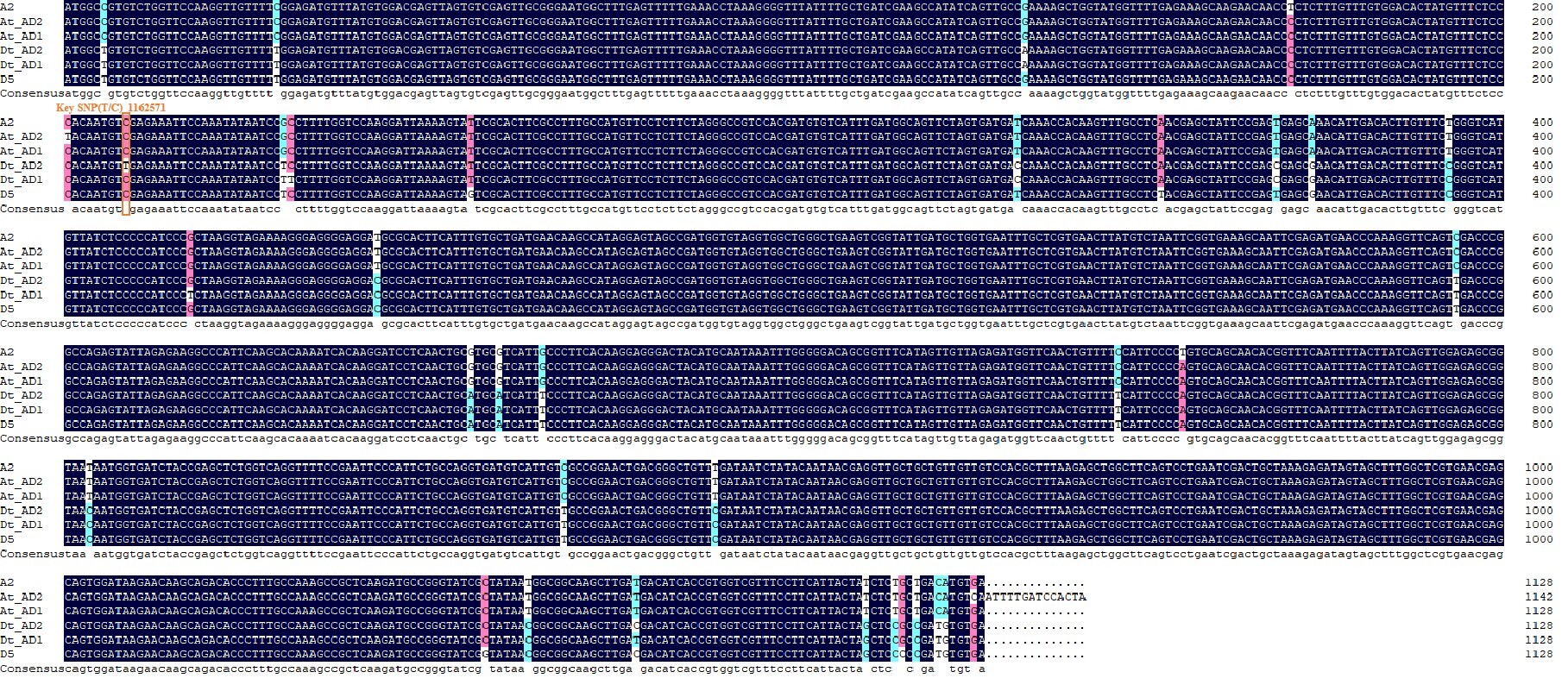


**Figure S8.** Multiple alignment of *GbPP2C80* in AD_2_ and its homologues in diploid ancestral cotton species, A_2_ and D_5_, and the tetraploid cultivated cotton species AD_1_. The orange box indicates the location of key non-synonymous SNP.


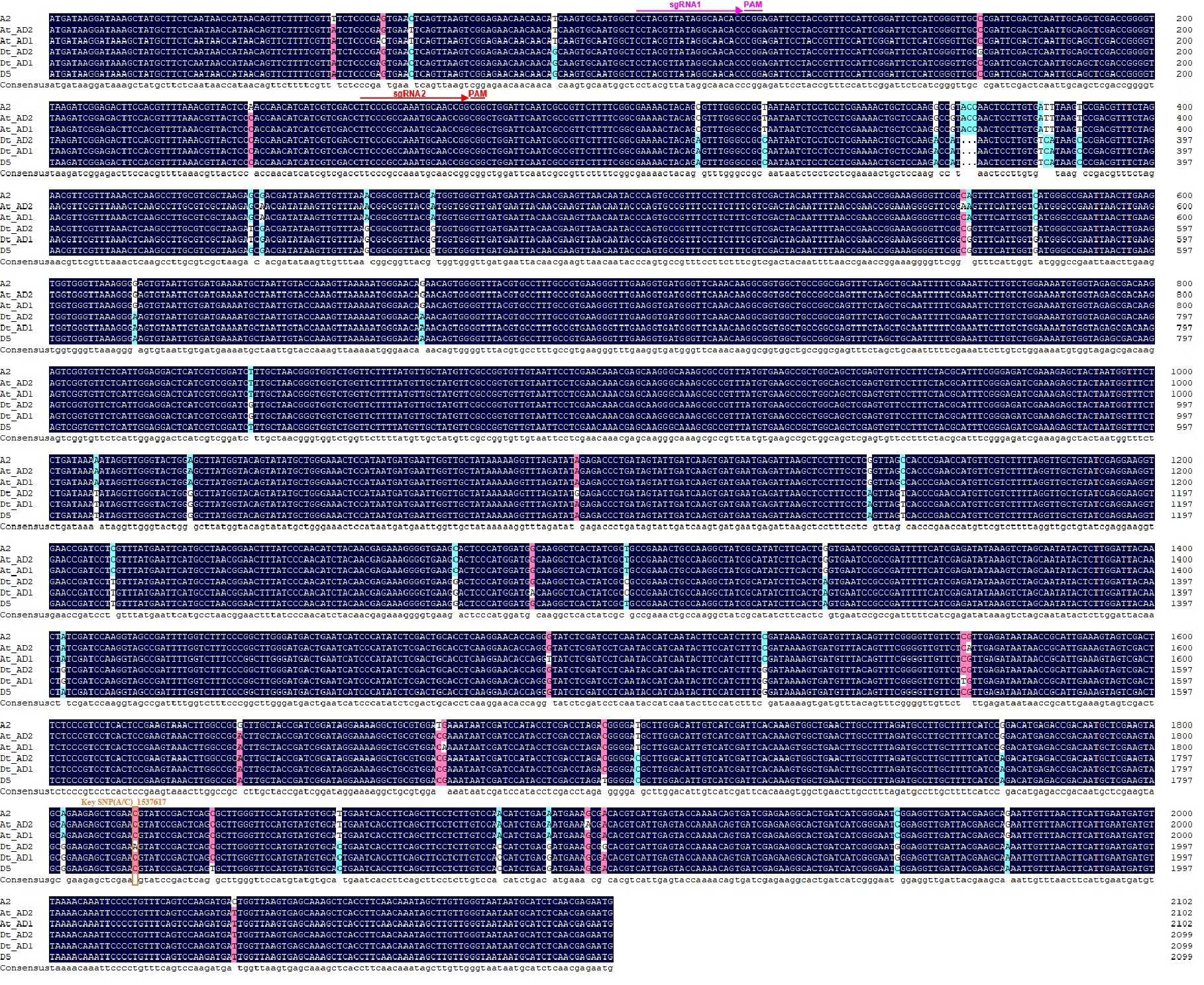


**Figure S9.** Multiple alignment of *GbWAKL14* in AD_2_ and its homologues in diploid ancestral cotton species, A_2_ and D_5_, and tetraploid cultivated cotton species, AD_1_. The orange box indicated the location of key non-synonymous SNP.


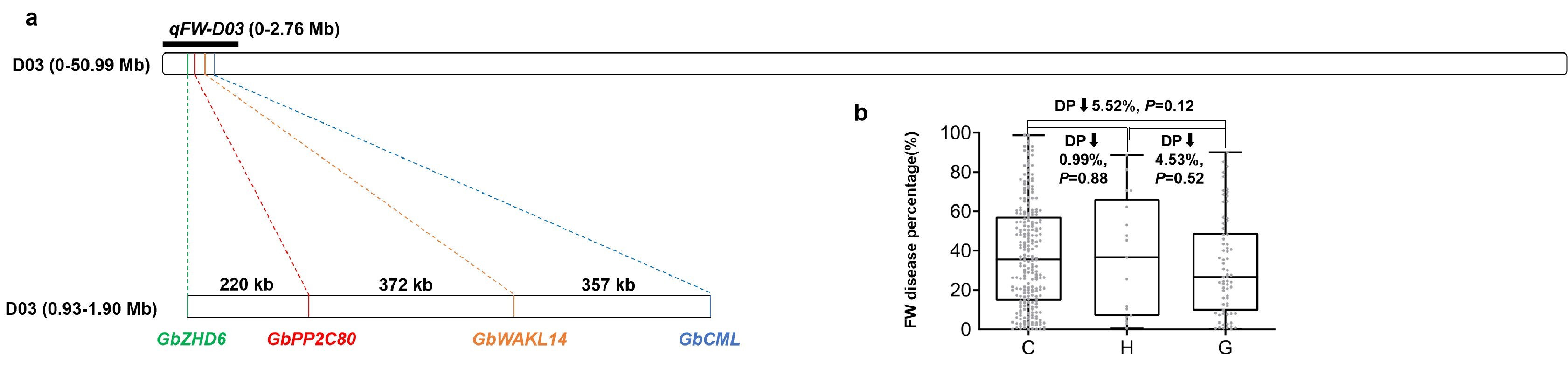


**Figure S10.** *FW* resistance genes on the chromosome D03 in Sea Island cotton. (a) The location of *FW*-resistance QTL and genes on D03 chromosome. (b) The effects of different haplotypes of *GbCML* on *FW* disease percentage in our Sea Island cotton population.


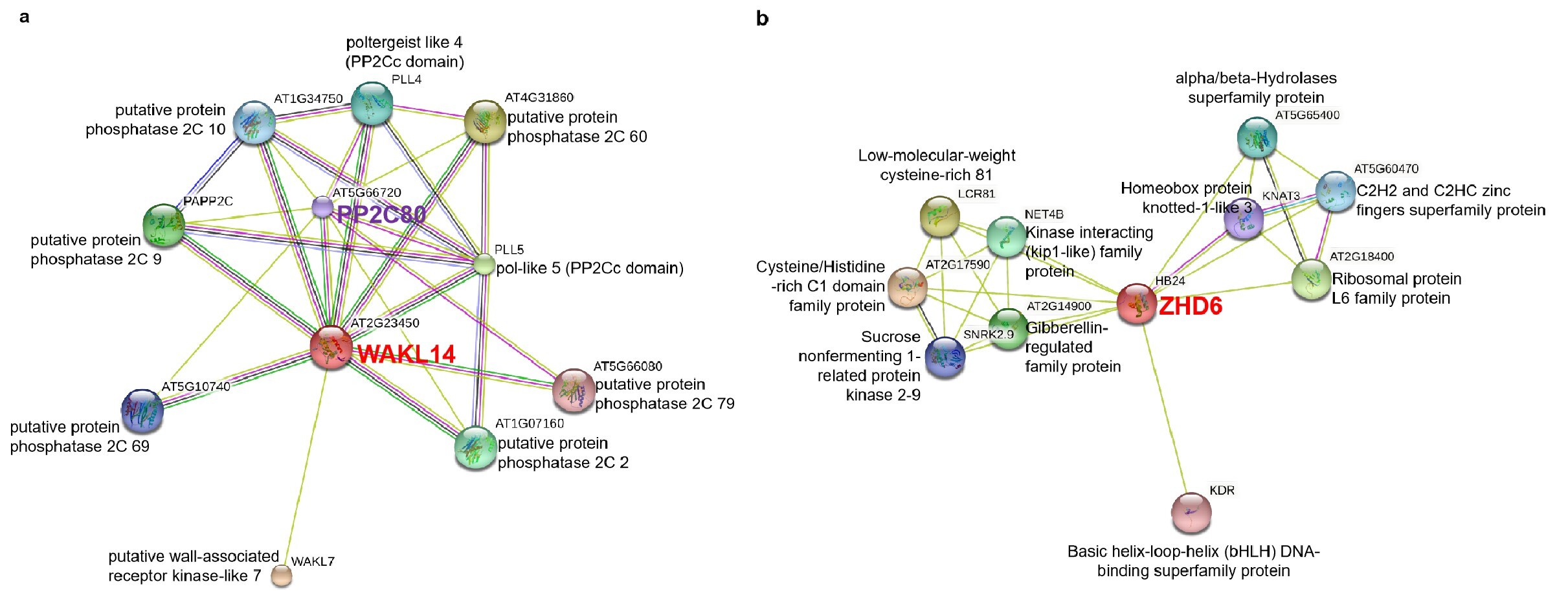


**Figure S11.** Predicted interaction proteins with WAKL14 and ZHD6 in *A. thaliana*. (a) The predicted interacting proteins with WAKL14 in *A. thaliana.* The annotations of each protein include the name (or protein ID) and the description of its function in *A. thaliana.* (b) The predicted interacting proteins with ZHD6. These two networks were predicted by software STITCH (http://stitch.embl.de/).


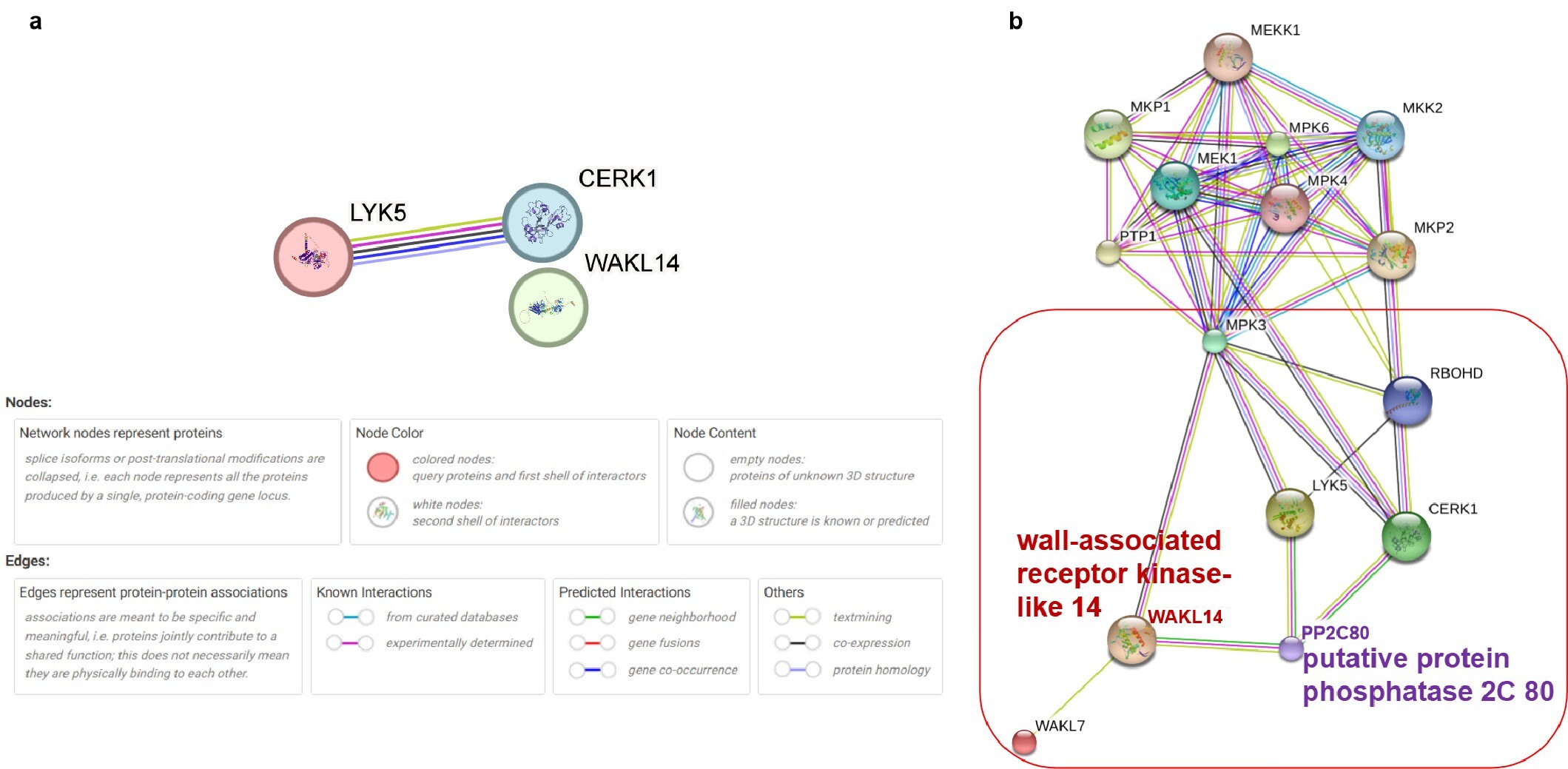


**Figure S12.** Interaction predictions for WAKL14, PP2C80, and other immune-related proteins in *A. thaliana*. (a) Interaction prediction of WAKL14, LYK5 and CERK1. The prediction software was STRING (https://cn.string-db.org/). (b) Interaction prediction of WAKL14, PP2C80, receptor kinases (LYK5, CERK1, and WAKL7), MAPK cascade proteins, ROS production protein (RBOHD). The prediction software was STITCH (http://stitch.embl.de/).

**Table S6.** Primers used in this study.

| **Gene** | **Vector** | **Purpose** | **Forward primer** | **Reverse primer** | |
| --- | --- | --- | --- | --- | --- |
| *GbPP2C80* | pCLCrVA | VIGS | GGACTAGTTGTCATTTGATGGCAGTTCT | CCTTAATTAATCACCATTGTTACCGCTCT | |
| *GbPP2C80* | p2301 | Overexpression | TCTAGAACTATGGCTGTGTCTGGTTCC | GGTACCGGGCCTCATACCTAAGTCCATC | |
| *GbWAKL14* | p2301 | Overexpression | GGGGTACCGTTATTAATTGCATTGCGGC | CGGACTAGTATGTATGATTTTTTACAAAGGTCTCA | |
| *GbWAKL14* | pRGEB32-GhU6.9 | sgRNA1 detection | GGAGTGAGTACGGTGTGCCGAGTGAACTCAGTTAAGTCGG | GAGTTGGATGCTGGATGGTGCAATTGAGTCGAATCGG | |
| *GbWAKL14* | pRGEB32-GhU6.9 | sgRNA2 detection | GGAGTGAGTACGGTGTGCCCGATTCGACTCAATTGCA | GAGTTGGATGCTGGATGGAAGGAGTTATGGTCTTGGAGCA | |
| *GbWAKL14* | pRGEB32-GhU6.9 | NPT*Ⅱ* detection | ACTGGGCACAACAGACAATCG | GCATCAGCCATGATGGATACTTT | |
| *GbWAKL14* | pRGEB32-GhU6.9 | Cas 9 detection | TTCAAGGTGCTGGGCAACAC | CTCAGGATGTCGCTCAGCAG | |
| *GbPP2C80* | p35S::GFP | GFP | GGTACCATGGCTGTGTCTGGTTCCA | TCTAGACACATCGGCGGAGCTAGT | |
| *GbWAKL14* | p35S::GFP | GFP | GGTACCATGATAAGGATAAAGCTATGCTTC | CCCGGGTTCTCGTTGAGATGCATTATTAC | |
| *GbPP2C80* | pCambia 1300-YFPN | BiFC assay | GGTACCATGGCTGTGTCTGGTTCCA | ACTAGTCACATCGGCGGAGCTAGT | |
| *GbPP2C80* | pCambia 1300-YFPC | BiFC assay | GGTACCATGGCTGTGTCTGGTTCCA | ACTAGTCACATCGGCGGAGCTAGT | |
| *GbWAKL14* | pCambia 1300-YFPN | BiFC assay | GGTACCATGATAAGGATAAAGCTATGCTTC | ACTAGTTTCTCGTTGAGATGCATTATTAC | |
| *GbMPK3* | pCambia 1300-YFPN | BiFC assay | GGATCCATGGCTGACGTCGCTCCG | GTCGACAGCATAAGTTGGATTCAGAGCCA | |
| *GbMPK3* | pCambia 1300-YFPC | BiFC assay | GGATCCATGGCTGACGTCGCTCCG | GTCGACAGCATAAGTTGGATTCAGAGCCA | |
| *GbRbohD* | pCambia 1300-YFPC | BiFC assay | GAATTCATGAAGAATGAAGATTGGAGAGGA | GTCGACAAAGTTCTCTTTGTGAAAATCAAACT | |
| *GbPP2C80* | pGBKT7 | Y2H | ATGGAGGCCGAATTCATGGCTGTGTCTGGTTCCA | GCAGGTCGACGGATCCCACATCGGCGGAGCTAGT | |
| *GbWAKL14* | pGADT7 | Y2H | ATGGAGGCCAGTGAATTCATGATAAGGATAAAGCTATGCTTC | CTCGAGCTCGATGGATCCTTCTCGTTGAGATGCATTATTAC | |
| *GbUBQ7* |  | qRT-PCR | GAAGGCATTCCACCTGACCAAC | CTTGACCTTCTTCTTCTTGTGCTTG | |
| *GbPP2C80* |  | qRT-PCR | TCAGTTGACCCGGCCAGAGTAT | TGTTGCTGCACTGGGGAATGAA | |
| *GbWAKL14* |  | qRT-PCR | CTACAGAGTTTGGGCCGCCAAT | CTACAGAGTTTGGGCCGCCAAT | |
| *GbMPK3* |  | qRT-PCR | TCTGCCCCGAACCGTTTTCTTT | TCAGAGCCAAGGCCTCTTGGTA | |
| *GbRbohD* |  | qRT-PCR | TTGGGGTAGCCCTACACGGAAT | GGAAGATTGAGCTTGTTGCGCC | |
| *GbUBQ7* |  | qRT-PCR | GAAGGCATTCCACCTGACCAAC | CTTGACCTTCTTCTTCTTGTGCTTG | |
| *FOV* |  | qRT-PCR | CCACTGTGAGTACTCTCCTCG | CCCAGGCGTACTTGAAGGAAC | |
| *GbPP2C80* |  | S marker | TATTTTTTCAGCTGTTCGAGTTTTA | TTTCAAGGTTCCTATTCTTTGTTCA | |
| *GbPP2C80* |  | R marker | GGTTGTTCGAGTTTTGTCTTATTATTA | | TTTCAAGGTTCCTATTCTTTGTTCA |
| *GbWAKL14* |  | S/R marker | TATTGTAACCATTGATTGAATCACA | TGCGTAAAAAAAAAAAAACTAAAA | |

**Table S7.** Information of 20 extreme susceptible/resistant Sea Island cotton varieties.

| **Catergory** | **Code** | **Name** | **Gene haplotype** | | **Disease percentage (%)** | | | | |
| --- | --- | --- | --- | --- | --- | --- | --- | --- | --- |
|  |  |  | ***GbPP2C80*** | ***GbWAKL14*** | **Korla, 2015** | **Korla, 2016** | **Korla, 2018** | **Korla, 2019** | **Mean value** |
| Susceptible | S1 | II15-3464 | T | A | NA | NA | NA | 98.78 | 98.78 |
|  | S2 | Su7871-и | T | A | NA | NA | NA | 98.75 | 98.75 |
|  | S3 | II15-3493 | T | A | NA | NA | NA | 95.70 | 95.70 |
|  | S4 | 3761 | T | A | NA | NA | NA | 94.29 | 94.29 |
|  | S5 | YJ005499 | T | A | NA | NA | NA | 93.10 | 93.10 |
|  | S6 | II15-3460 | T | A | NA | NA | NA | 93.01 | 93.01 |
|  | S7 | XKK213 | T | A | NA | NA | NA | 91.04 | 91.04 |
|  | S8 | YL2 | T | A | 88.10 | 76.19 | 89.85 | 90.02 | 86.04 |
|  | S9 | JB91-44 | T | A | 76.67 | 85.40 | 85.27 | 86.16 | 83.37 |
|  | S10 | AK785-3 | T | A | 80.00 | 69.23 | 91.05 | 90.98 | 82.82 |
| Resistant | R1 | T10-280 | C | C | 0.00 | 0.00 | 0.00 | 0.00 | 0.00 |
|  | R2 | XH42 | C | C | 0.72 | 0.00 | 0.00 | 0.00 | 0.18 |
|  | R3 | XH49 | C | C | 0.74 | 0.00 | 0.00 | 0.00 | 0.18 |
|  | R4 | XH51 | C | C | 0.76 | 0.00 | 0.00 | 0.00 | 0.19 |
|  | R5 | XH43 | C | C | 0.00 | 0.00 | 0.00 | 1.14 | 0.28 |
|  | R6 | XH38 | C | C | 1.43 | 0.00 | 0.00 | 0.00 | 0.36 |
|  | R7 | XH56 | C | C | 1.56 | 0.00 | 0.00 | 0.00 | 0.39 |
|  | R8 | XH46 | C | C | 2.17 | 0.00 | 0.00 | 0.00 | 0.54 |
|  | R9 | XH48 | C | C | 2.34 | 0.00 | 0.00 | 0.00 | 0.59 |
|  | R10 | XH57 | C | C | 3.08 | 0.00 | 0.00 | 0.00 | 0.77 |

**Table S8.** Primers used for CRISPR/Cas9 recombinant vector construction.

| **Target Name** | **Target sequence (5’-3’)** |
| --- | --- |
| pRGEB32-7s | AAGCATCAGATGggcaAACAAAGCACCAGTGGTCTAG |
| inf pRGEB32-7s | AAGCATCAGATGGGCAAACAAA |
| GbWAKL14-sgRNA1as | GGTGTTGCCTATAACGTAGGTGCACCAGCCGGGAAT |
| GbWAKL14-sgRNA1s | CCTACGTTATAGGCAACACCGTTTTAGAGCTAGAAATA |
| GbWAKL14-sgRNA2as | CCGGTTGCATTTGGCGGGAATGCACCAGCCGGGAAT |
| GbWAKL14-inf-sgRNA2as | TTCTAGCTCTAAAACCCGGTTGCATTTGGCGGGAA |
